# Defluorinative Cyclization of Enamides with Fluoroalkyl Halides Through Two Vicinal C(sp^3^)─F Bonds Functionalization

**DOI:** 10.1002/advs.202404738

**Published:** 2024-12-31

**Authors:** Yu‐Lan Chen, Wei Han, Yuan‐Yuan Ren, Mengtao Ma, Danhua Ge, Zhi‐Liang Shen, Kai Guo, Xue‐Qiang Chu

**Affiliations:** ^1^ Technical Institute of Fluorochemistry School of Chemistry and Molecular Engineering Nanjing Tech University Nanjing 211816 China; ^2^ Department of Chemistry and Materials Science College of Science Nanjing Forestry University Nanjing 210037 China; ^3^ College of Biotechnology and Pharmaceutical Engineering Nanjing Tech University Nanjing 211816 China

**Keywords:** 1,2‐difluoroalkenyl group, C─F bond functionalization, defluorinative cyclization, fluoroalkyl halides, oxazole

## Abstract

Introducing distinctive functional groups to expand the structural diversity and improve the intrinsic properties of parent molecules has been an essential pursuit in organic chemistry. By using perfluoroalkyl halide (PFAH) as a nontraditional, readily available, ideal 1,2‐difluoroalkenyl coupling partner, a defluorinative cyclization reaction of enamides for the construction of fluoroalkenyl oxazoles is first developed. The selective and controllable two‐fold cleavage of vicinal C(sp^3^)─F bonds in PFAH not only enables the introduction of a specific 1,2‐difluoroalkenyl moiety with ease but also results in the functionalization of two C(sp^2^)─H bonds of enamides without the need for metal catalyst, photocatalyst, oxidant, or light. The method can be applied to the late‐stage modification of complex molecules, synthesis of biological‐relevant oxazole analoges, and scale‐up synthesis, which all further highlight the real‐world utility of this protocol. Mechanistic studies reveal that the reaction possibly proceeds through a radical perfluoroalkylation, consecutive C─F bond heterolytic cleavage, and cyclization process. In addition, the in situ formed perfluoroalkyl radical which may also serve as an essential hydrogen abstractor.

## Graphical abstract

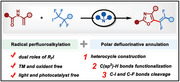

## 1 Introduction

**Figure 1:**
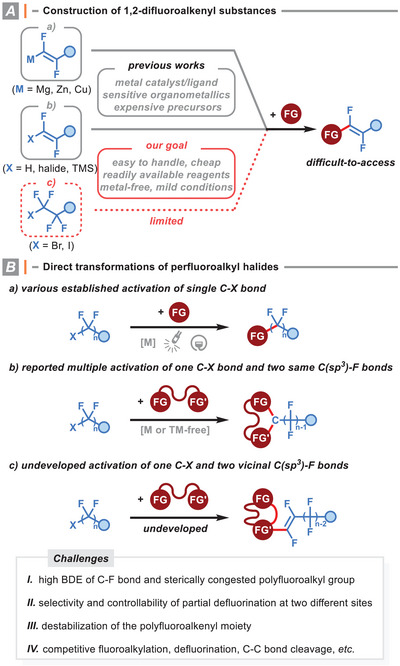
Construction of 1,2‐difluoroalkenyl unit and the present reaction manifolds of perfluoroalkyl halide (PFAH).

Significant physicochemical enhancements in terms of lipophilicity, solubility, metabolic stability, and bioavailability conferred by modification of target molecules with fluorine atoms have garnered widespread recognition in fields ranging from pharmaceuticals,^[^ ^1^ ^]^ and agrochemicals^[^ ^2^ ^]^ to materials science.^[^ ^3^ ^]^ In particular, fluoroalkenes demonstrate their superiority over the non‐fluorinated counterparts, which are commonly found in fluoropolymers, liquid crystalline materials, and biologically active agents (for selected reviews see ref. [4]). Nevertheless, unique 1,2‐difluoroalkenyl structures are limited in accessibility because of the dearth of straightforward and broadly applicable strategies to synthesize 1,2‐difluoroalkenes from commodity feedstocks.^[^ ^5^ ^]^ Most research attention has been focused on coupling reactions by employing prefunctionalized fluoroalkenyl precursors (for selected reviews see ref. [6]) and/or highly reactive fluorinated organometallic reagents (e.g., organolithium, magnesium, zinc, aluminum, and copper reagents)^[^ ^7^ ^]^ (**Figure** **1**A, a,b). Evidently, there are substantial drawbacks associated with them, including multistep manipulation, the need for toxic/sensitive reagents, the use of expensive metal catalysts/ligands, moisture exclusion, harsh reaction conditions, and the lack of efficiency. Moreover, the high basicity and nucleophilicity of organometallic reagents often lead to poor chemoselectivity and low compatibility with functional groups, thereby hampering their practical applications in organic synthesis.

Perfluoroalkyl halides R_f_X (PFAHs, X = Br, I) are commercially available, low‐toxic, cost‐effective, stable, and easy‐to‐handle (liquid or solid) chemicals.^[^ ^8^ ^]^ They have shown great promise for the production of various fluorinated molecules.(for selected examples, see ref. [9]. Our reaction hypothesis is based on the selective and controllable transformation of two vicinal C(sp^3^)─F bonds in PFAH, which renders it a versatile and ideal 1,2‐difluoroalkenyl building block (Figure 1A, c). If successful, the aforementioned shortcomings related to the specialized 1,2‐difluoroalkenyl motif installation would be effectively addressed.^[^ ^6^, ^7^ ^]^ However, the state‐of‐the‐art synthesis of organofluorides by utilizing PTAHs as substrates is largely limited to the functionalization of either one or three carbon─halogen bonds at the terminal carbon atom (Figure 1B, a,b).^[^ ^8^ ^]^(for selected reviews see refs. [9, 10]) One sporadic example, reported by Chen and co‐workers, only restricted to the straightforward radical difluoroalkenylation to specific porphyrins.^[^ ^11^ ^]^ Very recently, the groups of Nishimot/Yasuda^[^ ^12a–c^ ^]^ and Liu^[^ ^12d^ ^]^ independently achieved photo‐ or Ni‐catalyzed selective single or dual C(sp^3^)─F bond activation of the CF_2_ unit in perfluoroalkyl compounds (1‐2F@1C). Despite these considerable advancements, ready methods for the activation of multiple C─F bonds on different carbon atoms of perfluoroalkyl groups are still scarce (2F@2C). On the other hand, our hypothetical reaction mode faces other serious issues (Figure 1B,c), including 1) difficulties in the activation of the robust C(sp^3^)─F bonds in perfluoroalkyl groups which are intrinsic less reactive and sterically congested;^[^ ^13^, ^14^ ^]^ 2) occurrence of non‐site‐selective transformations and undesired multi‐activation of C─F and other C─C bonds;^[^ ^15^ ^]^ 3) the competing interference of the generated fluoroalkenyl moiety due to the harsh reaction conditions and the highly reactive reagents that are generally needed in the reactions.^[^ ^16^ ^]^

### 1.1 Reaction Design and Optimization

**Figure 2:**
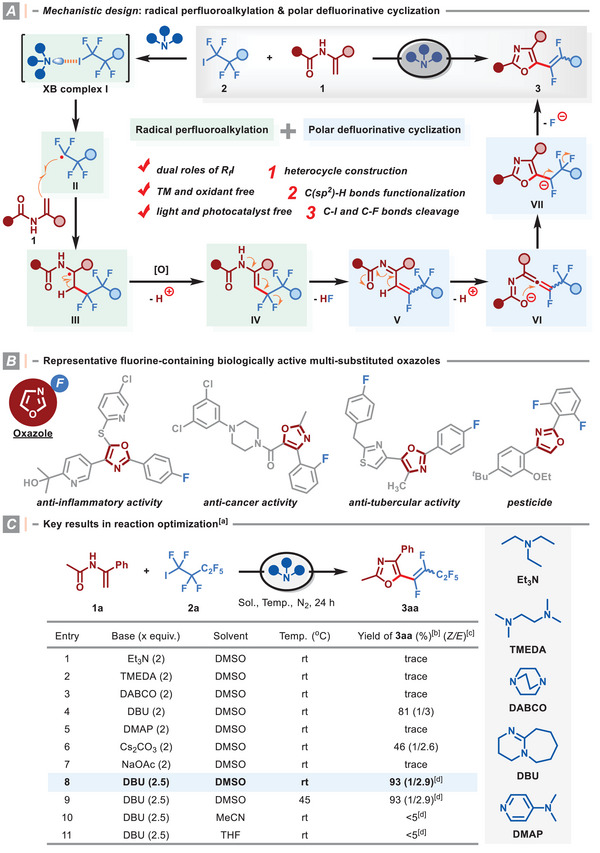
Mechanistic design, representative fluorine‐containing biologically active oxazoles, and key results in the reaction optimization. ^[a]^ Reaction conditions: **1a** (0.3 mmol), **2a** (0.6 mmol), and base (0.6–0.75 mmol) in solvent (2 mL) at rt‐45 °C under N_2_ for 24 h. ^[b]^ Isolated yields. ^[c]^ Ratio of Z/E in parentheses was determined by ^19^F NMR analysis. ^[d]^ **2a** (0.9 mmol) was used.

We envisioned a possible relay involving radical perfluoroalkylation and downstream polar defluorination process using terminal enamide **1** and PFAH **2** as substrates (**Figure** **2**A). When an amine is present, it initially interacts with PFAH to form an encounter complex **I** via halogen bonding (XB).(for selected examples, see ref. [17]).Subsequently, an electrophilic perfluoroalkyl radical **II** is formed from the collapse of XB adduct through intramolecular electron transfer (ET), (for selected examples, see ref. [18]) and it can be readily intercepted by enamide **1** to deliver an α‐amino radical **III**. Then, the oxidation of the reactive radical intermediate **III** by an excess of perfluoroalkyl radicals or other oxidative species, such as the radical cation from DBU,^[^ ^18c^ ^]^ produced the perfluoroalkylated enamide **IV**, which undergoes rapid polar defluorination under basic conditions, leading to the fluoroallene species **VI**. Further intramolecular *O*‐nucleophilic cyclization and ensuing β‐F elimination at an adjacent carbon site forge the desired 1,2‐difluoroalkyl product 3. (for selected examples of redox‐neutral defluorination, see ref. [19]).It is worth noting that PFAH might serve as a dual‐role reagent in this system—a perfluoroalkyl radical precursor through C─X bond cleavage and a hydrogen abstractor for the oxidation of the reactive α‐amino radical **III**. (for selected examples, see ref. [20]).Moreover, consecutive two vicinal C(sp^3^)─F bonds cleavage of PFAHs not only provides a conceptually novel approach for the access to privileged 1,2‐difluoroalkyl oxazole derivatives (Figure 2B) (For selected example and reviews, see ref. [21]) but also results in metal‐/oxidative‐/light‐/photocatalyst‐free two C(sp^2^)─H bonds functionalization of enamides.^[^ ^22^ ^]^

Herein we describe the successful merger of radical perfluoroalkylation and twofold C(sp^3^)─F bond functionalization for the 1,2‐difluoroalkenylation of enamides. Initially, *N*‐(1‐phenylvinyl)acetamide (**1a**) and perfluorobutyl iodide (**2a**) were selected as benchmark substrates to evaluate various base promoters (Figure 2C, entries 1–7). In the presence of DBU, we observed that the desired reaction proceeded smoothly to deliver the oxazole **3aa** in 81% yield with a *Z*/*E* ratio of 1/3 (entry 4). The use of Cs_2_CO_3_ as a base gave the product in 46% yield (entry 6), while other organic or inorganic bases, including Et_3_N, TMEDA, DABCO, DMAP, and NaOAc, resulted in poor yields (entries 1–3, 5, and 7). Moreover, increasing the amounts of **2a** and DBU could further enhance the reaction yield to 93% with a *Z*/*E* ratio of 1/2.9 (entry 8). Varying the reaction temperature from 25 to 45 °C did not influence the reaction performance (entry 9). However, the reaction conducted in MeCN or THF showed low efficiency (entries 10–11). These results could be attributed to the strongly polar DMSO, which is beneficial for the formation of perfluoroalkyl radicals.^[^ ^23^ ^]^

### 1.2 Substrate Scope Studies

**Scheme 1:**
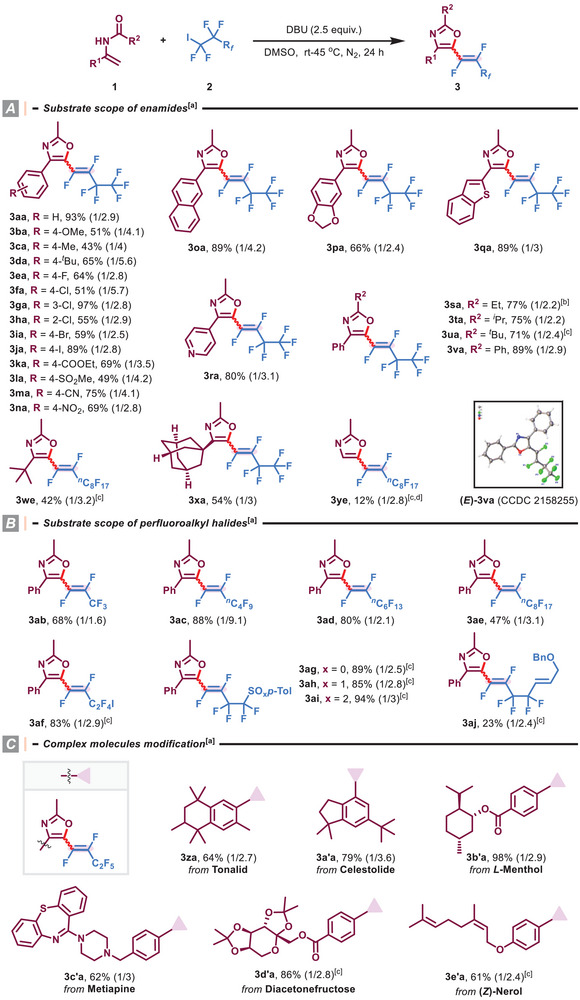
Substrate scope of various enamides and perfluoroalkyl halides. ^[a]^ Standard reaction conditions; isolated yield; ratio of Z/E in parentheses was determined by 19F NMR analysis. ^[b]^ 40 W blue LEDs was used. ^[c]^ 45 °C. ^[d]^ **1y** (0.9 mmol) and **2e** (0.3 mmol) were used.

With the optimized reaction conditions in hand, the substrate scopes with respect to enamides **1** and fluoroalkyl halides **2** were studied (**Scheme** **1**). As shown in Scheme 1A, a variety of oxazole derivatives containing electronically or sterically varied substituents (R) on the aryl moiety were smoothly synthesized in acceptable yields with moderate *Z*/*E* selectivity (**3aa‐3na**). Useful functional groups, such as methoxy, halogens, ethoxycarbonyl, sulfonyl, cyano, and nitro were compatible with this mild reaction, thus providing great flexibility for further synthetic elaboration. Moreover, the reaction efficiency was not impeded by *ortho*‐ or *meta*‐chloro substituents on the phenyl ring (97% and 55% yield, respectively, **3ga** and **3ha**). It is worth mentioning that the transformations of fused aromatic and heterocyclic *N*‐vinylacetamides {e.g., naphthalene, benzo[*d*][1,3]dioxole, benzo[*b*]thiophene, pyridine} successfully occurred (**3oa‐3ra**). Replacing the *N*‐substitution pattern (COR^2^) with other acyl groups, including propionyl, isobutyryl, pivalyl, and benzoyl, had no negative influence on the reaction outcomes (**3sa‐3va**). The related structure of the major product (*E*)−**3va** was characterized by X‐ray crystallographic analysis.^[^ ^24^ ^]^ Besides (hetero)aryl enamides, aliphatic substrates derived from sterically congested ketones including 3,3‐dimethylbutan‐2‐one and adamantan‐1‐yl)ethan‐1‐one were also competent reaction partners, delivering products **3we** and **3xa** in 42% and 54% yields, respectively. Particularly noteworthy is the formation of product **3ye** with an unsubstituted *C*4‐position, which remains intact despite the presence of excessive perfluorodecyl iodide.

Subsequently, fluoroalkyl halides possessing length‐varied perfluroalkyl chains (C3‐C10) were found to smoothly react with *N*‐(1‐phenylvinyl)acetamide (**1a**), furnishing the corresponding products **3ab‐3ae** in 47–88% yields (Scheme 1B). When synthetically valuable handles, such as iodine (**3af**), sulfoether (**3ag**), sulfoxide (**3ah**), sulphone (**3ai**), and alkene (**3aj**), were preinstalled on the side of the fluoroalkyl group, no significant loss in efficiencies and selectivities was witnessed. Typically, these privileged fluorovinyl azacycles are otherwise challenging to obtain through traditional coupling methods, exemplifying the versatility and practicality of this protocol.^[^ ^21^ ^]^

Our mild process also permitted late‐stage modification of more complex molecules, as demonstrated by transformations of Tonalid (**3za**), Celestolide (**3a'a**), *L*‐Menthol (**3b'a**), Metiapine (**3c'a**), Diacetonefructose (**3d'a**), and (*Z*)‐Nerol (**3e'a**) derivatives (Scheme 1C).

### 1.3 Synthetic Applications

**Scheme 2:**
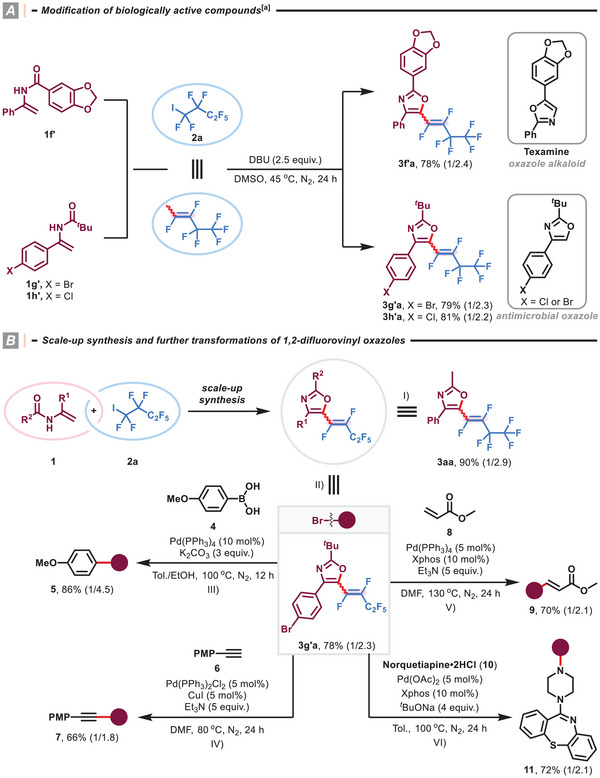
Synthetic applications. I) Scale‐up synthesis of product **3aa**. II) Scale‐up synthesis of product **3g'a**. III) Suzuki coupling reaction of product **3g'a** with 4‐methoxyphenylboronic acid (**4**). IV) Sonogashira coupling reaction of product **3g'a** with 4‐ethynylanisole (**6**). V) Heck reaction of product **3g'a** with methyl acrylate (**8**). VI) Buchwald‐Hartwig coupling reaction of product **3g'a** with norquetiapine∙2HCl (**10**). ^[a]^ Standard reaction conditions; isolated yield; ratio of Z/E in parentheses was determined by ^19^F NMR analysis.

The power of the present defluorinative cyclization is illustrated by introducing fluoroalkenyl moiety on biologically active‐relevant compounds (**Scheme** **2**A). The diaryloxazole alkaloid texamine and 2‐(*tert*‐butyl)−4‐(4‐halophenyl)oxazoles were found to be associated with antimycobaterial and antimicrobial activities (For selected examples, see ref. [25], and similar fluoroalkenylated analogues **3f'a‐3h'a** could be prepared in good yields utilizing the present methodology. Furthermore, comparable yields were obtained for the gram‐scale preparation of products **3aa** (90% yield, 1.89 g) and **3g'a** (78% yield, 1.73 g), revealing the scalability of the reaction (Scheme 2B, I,II). When the obtained product **3g'a** was decorated with bromide substituent, it became feasible to introduce more diverse structural features. Valuable motifs, such as arene (Scheme 2B, III; 86% yield, *Z*/*E* = 1/4.5; **5**), alkyne (Scheme 2B, IV; 66% yield, *Z*/*E* = 1/1.8; **7**), acrylate (Scheme 2B, V; 70% yield, *Z*/*E* = 1/2.1; **9**), and Norquetiapine (Scheme 2B, VI; 72% yield, *Z*/*E* = 1/2.1; **11**), could be facilely integrated with oxazole core, and the structure and the configuration of the fluorovinyl group remained unreacted in different Pd‐catalyzed cross‐coupling reactions Figure [Supplementary-material advs8947-supitem-0001].

### 1.4 Mechanistic Investigations

**Scheme 3:**
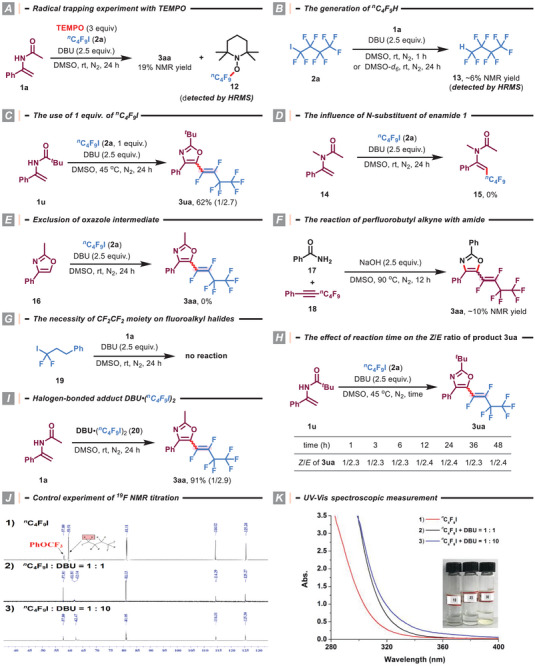
Mechanistic studies.

To gain more insights into the reaction mechanism, several experiments were conducted (**Scheme** **3**). First, 2,2,6,6‐tetramethylpiperidinooxy (TEMPO) was added to the model reaction to confirm whether a radical pathway was involved or not (Scheme 3A). As expected, the yield of product **3aa** was dramatically reduced, and TEMPO‐trapped *^n^* C_4_F_9_ radical adduct **12** was detected. This probe experiment demonstrated that the reaction proceeds via a radical process.^[^ ^18^ ^]^ Second, the generation of *^n^* C_4_F_9_H was observed in the system, supporting the hydrogen abstracting function of *^n^* C_4_F_9_ radical (Scheme 3B).^[^ ^20^ ^]^ Meanwhile, the low NMR yield of *^n^* C_4_F_9_H also suggested the presence of other hydrogen abstractors or oxidative species. Third, the fact that the product **3** **ua** could be obtained in 62% yield when 1 equiv. of perfluorobutyl iodide was used further supported the above speculation (Scheme 3C).^[^ ^23^ ^]^ Fourth, the use of *N*‐methyl enamide **14** resulted in no detectable product under standard reaction conditions, thereby suggesting the necessity of the NH pattern on the enamide for successful transformation (Scheme 3D). We excluded the possibility of the product being generated from the direct fluoroalkenylation of oxazole because the exposure of presynthesized oxazole **16** to the standard reaction conditions failed to give the desired product **3aa** (Scheme 3E).^[^ ^11^ ^]^ This experiment also indicated that an intermolecular perfluoroalkylation event might occur before intramolecular heterocyclization. Furthermore, product **3aa** was obtained with ≈10% NMR yield from the reaction of benzamide (**17**) with (perfluorohex‐1‐yn‐1‐yl)benzene (**18**) in the presence of NaOH as the base (Scheme 3F). The production of **3aa** is probably ascribed to the hydroamination^[^ ^26^ ^]^ of perfluoroalkyl alkyne to generate a key perfluoroalkylated enamide (also see Figure 2, IV), which subsequently undergoes consecutive defluorination to afford the corresponding product, thereby reinforcing the aforementioned conclusions in Scheme 3E. Moreover, the use of (3,3‐difluoro‐3‐iodopropyl)benzene (**19**) as the fluoroalkyl halide substrate did not yield any products, indicating the necessity of the CF_2_CF_2_ moiety for the successful transformation (Scheme 3G). As expected, the reaction time almost had no influence on the reaction stereoselectivity, as the *Z*/*E* ratio of product **3** **ua** did not change with reaction times ranging from 1 to 48 h (Scheme 3H). In addition, when a solution of DBU•(*^n^* C_4_F_9_I)_2_ (**20**) and acetamide **1a** was performed at room temperature for 24 h, 91% yield of product **3aa** was isolated (Scheme 3I). This result strongly demonstrates the crucial role played by the DBU•(*^n^* C_4_F_9_I)_2_ complex in this defluorinative cyclization.^[^ ^18^ ^]^ The existence of the proposed halogen bonding interaction between DBU and *^n^* C_4_F_9_I was further verified by the ^19^F NMR titration experiment and UV–vis spectroscopic measurement.^[^ ^27^ ^]^ It was found that the resonance corresponding to the F of the CF_2_I group shifted to the upfield region when DBU was added to the DMSO‐*d*_6_ solution of *^n^* C_4_F_9_I (Scheme 3J). In addition, we also observed a redshift of absorption and color change when *^n^* C_4_F_9_I and DBU were added in DMSO, indicating the formation of a halogen bonding adduct (Scheme 3K).^[^ ^17^, ^28^ ^]^

**Scheme 4:**
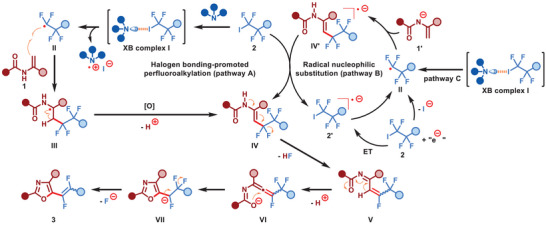
Proposed mechanism.

Based on the conducted control experiments and a literature survey, we propose two plausible mechanisms for the defluorinative cyclization of enamides with fluoroalkyl halides (**Scheme** **4**).^[^ ^17^, ^18^, ^23^ ^]^ One possible mechanism involves halogen bonding‐promoted radical perfluoroalkylation and a downstream polar defluorination process, which is consistent with the description in Figure 2A (pathway A).^[^ ^17^, ^18^ ^]^ However, based on the mechanistic studies, an alternative pathway consisting of a base‐promoted radical substitution for the formation of the key intermediate of perfluoroalkylated enamide **IV** cannot be excluded (pathway B).^[^ ^23^ ^]^ In the initiation step, the perfluoroalkyl radical anion **2′** could be generated by electron transfer, which easily decomposes to form the perfluoroalkyl radical **II** and iodine anion. Further interaction of the perfluoroalkyl radical **II** with the nucleophilic enamide anion **1′** under basic conditions gives the radical anion **IV’**, which, by lectron transfer with another molecule of perfluoroalkyl halide **2**, forms the enamide **IV** and regenerates the perfluoroalkyl radical anion **2′**, thus continuing the propagation cycle. In this cycle, DBU only serves as a base. Subsequently, the defluorination of enamide **IV** occurs to generate a fluoroallene species **VI**, which rapidly undergoes intramolecular *O*‐nucleophilic cyclization followed by β‐F elimination at an adjacent carbon site to form the desired 1,2‐difluoroalkyl product **3**. Additionally, the initial perfluoroalkyl radical **II** could also originate from the collapse of XB complex **I**, which then participates in the propagation cycle (pathway C).

In summary, we have developed an unprecedented defluorinative cyclization of enamides with fluoroalkyl halides under mild reaction conditions. A series of fluoroalkenyl oxazoles could be synthesized via an XB adduct‐induced and/or a base‐promoted radical perfluoroalkylation and sequential consecutive defluorination. Impressively, the selective and controllable two‐fold cleavage of vicinal C(sp^3^)─F bonds in fluoroalkyl halides not only permits the installation of a 1,2‐difluoroalkenyl moiety with ease but also results in the functionalization of two C(sp^2^)─H bonds of enamides without the need for metal catalyst, photocatalyst, oxidant, or light. The approach also features broad substrate scope, good functional group tolerance, excellent scalability, and useful synthetic applications. The existence of the XB complex was investigated by the ^19^F NMR titration experiment and UV–vis spectroscopic measurement. Given the increasing importance of heterocycle skeletons containing fluorinated moiety, this method provides a modular and reliable platform for accessing specific fluorinated heterocycles of medicinal and biological interest. Further research based on the functionalization of two vicinal C(sp^3^)─F bonds on fluoroalkyl halides is ongoing in our laboratory.

## Conflict of Interest

The authors declare no conflict of interest.

## Supporting information

Supporting Information

Supporting cif

## Data Availability

The data that support the findings of this study are available in the supplementary material of this article.
