# Supplementary material for: Defluorinative Cyclization of Enamides with Fluoroalkyl Halides Through Two Vicinal C(sp3)─F Bonds Functionalization
Source: Adv Sci (Weinh). 2024 Dec 31;12(8):2404738. doi: 10.1002/advs.202404738 (PMC11848561; doi:10.1002/advs.202404738)

ADVANCED SCIENCE
Open Access

Supporting Information

for *Adv. Sci.*, DOI 10.1002/advs.202404738

Defluorinative Cyclization of Enamides with Fluoroalkyl Halides Through Two Vicinal C(sp^3)—F Bonds Functionalization

Yu-Lan Chen, Wei Han, Yuan-Yuan Ren, Mengtao Ma, Danhua Ge, Zhi-Liang Shen, Kai Guo* and Xue-Qiang Chu**

Supporting Information

Defluorinative Cyclization of Enamides with Perfluoroalkyl Halides through Two Vicinal C(sp^3)-F Bonds Functionalization

Yu-Lan Chen,[a] Wei Han,[a] Yuan-Yuan Ren,[a] Mengtao Ma,[c] Danhua Ge,[a] Zhi-Liang Shen,*[,a] Kai Guo,*[,b] and Xue-Qiang Chu*[,a]

[a] *Technical Institute of Fluorochemistry, School of Chemistry and Molecular Engineering, Nanjing Tech University, Nanjing 211816, China. E-mails: ias_zlshen@njtech.edu.cn; xueqiangchu@njtech.edu.cn.*

[b] *College of Biotechnology and Pharmaceutical Engineering, Nanjing Tech University, Nanjing 211816, China. E-mail: guok@njtech.edu.cn.*

[c] *Jiangsu Provincial Key Lab for the Chemistry and Utilization of Agro-Forest Biomass, College of Chemical Engineering, Nanjing Forestry University, Nanjing 210037, China.*

Table of Contents

1. General information page S2
2. General procedures for the synthesis of 1,2-difluoroalkyl oxazoles page S2
3. Scale-up synthesis of product page S2
4. Further transformations of products page S3
5. Mechanistic studies page S5
6. Optimization of reaction conditions page S11
7. Characterization data for products page S12
8. The X-ray crystal structure of product **3va** page S29
9. References page S30
10. ^{1}H, ^{19}F, and ^{13}C NMR spectra of products page S31

General information

Unless otherwise stated, all reagents were purchased from commercial suppliers and used without further purification. All reactions were carried out under N_2 atmosphere using undistilled solvent. Melting points were recorded on an electrothermal digital melting point apparatus. ^{1}H, ^{19}F, and ^{13}C NMR spectra were recorded in $CDCl_3$ on Bruker Avance or Joel 400 MHz spectrometers. NMR splitting patterns are designated as singlet (s), doublet (d), triplet (t), quartet (q), multiplet (m), *etc*. The chemical shifts (δ) are reported in ppm and coupling constants (J) in Hz. High resolution mass spectrometry (HRMS) data were obtained on a Waters LC-TOF mass spectrometer (Xevo G2-XS QTof) using electrospray ionization (ESI) in positive or negative mode. A suitable crystal was selected and recorded on a XtaLAB AFC12 (RINC): Kappa single diffractometer. Column chromatography was generally performed on silica gel (300-400 mesh) and reactions were monitored by thin layer chromatography (TLC) using UV light to visualize the course of the reactions.

General procedures for the synthesis of 1,2-difluoroalkyl oxazoles

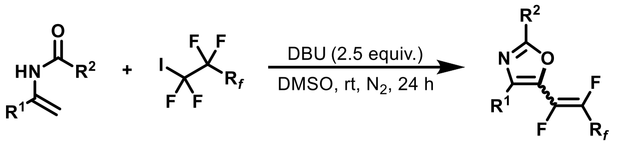

A solution of enamide[1] (0.3 mmol, 1 equiv., **1**), polyfluoroalkyl iodide (0.9 mmol, 3 equiv., **2**), and 1,8-diazabicyclo[5,4,0]undec-7-ene (114.2 mg, 0.75 mmol, 2.5 equiv., DBU) in DMSO (2.0 mL) was stirred at room temperature under N_2 atmosphere for 24 h. The reaction was then quenched by saturated NH_4Cl solution (20 mL) and extracted with EtOAc (20 mL x 3). The organic layer was washed with saturated brine twice, dried over $MgSO_4$, filtered, and concentrated under reduced pressure. The crude product was purified by flash column chromatography (300-400 mesh) using petroleum ether/ethyl acetate (100/1 ~ 10/1) as eluent to afford the pure products **3**.

Scale-up synthesis of product

1) Scale-up synthesis of product 3aa

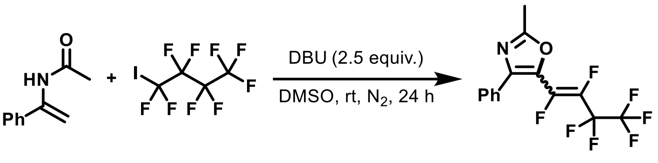

A solution of *N*-(1-phenylvinyl)acetamide (1.0 g, 6.2 mmol, 1 equiv., **1a**), perfluorobutyl iodide (6.4 g, 18.6 mmol, 3 equiv., **2a**), and 1,8-diazabicyclo[5,4,0]undec-7-ene (2.4 g, 15.5 mmol, 2.5 equiv., DBU) in DMSO (40 mL) was stirred at room temperature under N_2 atmosphere for 24 h. The reaction was then quenched by saturated NH_4Cl solution (70 mL) and extracted with EtOAc (70 mL x 3). The organic layer was washed with saturated brine twice, dried over $MgSO_4$, filtered, and concentrated under reduced pressure. The crude product was purified by flash column chromatography (300-400 mesh) using petroleum ether/ethyl acetate (100/1) as eluent to afford the pure product **3aa** (1.89 g, 90% yield).

2) Scale-up synthesis of product 3g'a

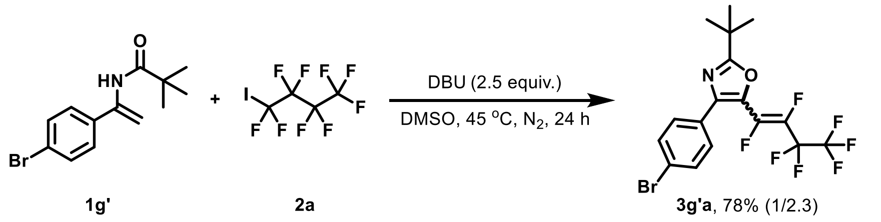

A solution of *N*-(1-(4-bromophenyl)vinyl)pivalamide (1.4 g, 4.8 mmol, 1 equiv., **1g'**), perfluorobutyl iodide (5.0 g, 14.4 mmol, 3 equiv., **2a**), and 1,8-diazabicyclo[5,4,0]undec-7-ene (1.8 g, 12 mmol, 2.5 equiv., DBU) in DMSO (32 mL) was stirred at 45 °C under N_2 atmosphere for 24 h. The reaction was then quenched by saturated NH_4Cl solution (50 mL) and extracted with EtOAc (50 mL x 3). The organic layer was washed with saturated brine twice, dried over $MgSO_4$, filtered, and concentrated under reduced pressure. The crude product was purified by flash column chromatography (300-400 mesh) using petroleum ether/ethyl acetate (50/1) as eluent to afford the pure product **3g'a** (1.73 g, 78% yield).

Further transformations of products

1) Suzuki coupling reaction of product 3g'a with 4-methoxyphenylboronic acid (4)

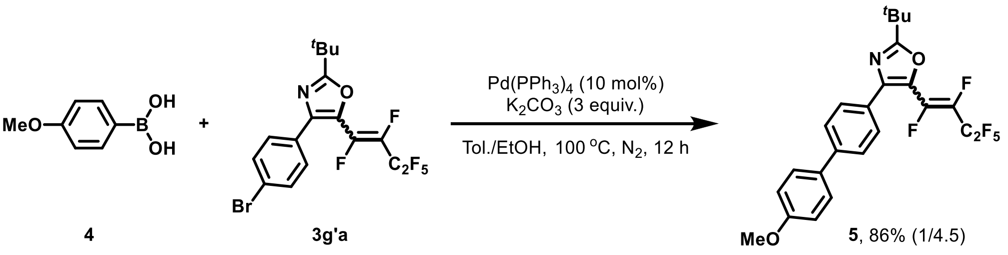

A solution of 4-(4-bromophenyl)-2-(*tert*-butyl)-5-(perfluorobut-1-en-1-yl)oxazole (138.1 mg, 0.3 mmol, 1 equiv., **3g'a**), 4-methoxyphenylboronic acid (91.2 mg, 0.6 mmol, 2 equiv., **4**), $Pd(PPh_3)_4$ (34.7 mg, 0.03 mmol, 0.1 equiv.), and K_2CO_3 (124.4 mg, 0.9 mmol, 3 equiv.) in toluene/EtOH (v/v = 1/1, 2 mL) was stirred at 100 °C under N_2 atmosphere for 12 h. The reaction was then quenched by saturated NH_4Cl solution (20 mL) and extracted with EtOAc (20 mL x 3). The organic layer was washed with saturated brine twice, dried over $MgSO_4$, and concentrated under reduced pressure. The crude product was purified by flash silica gel column chromatography (300-400 mesh) using petroleum ether/ethyl acetate (100/1) as eluent to afford the pure product **5** (125.5 mg, 86% yield).

2) Sonogashira coupling reaction of product 3g'a with 4-ethynylanisole (6)

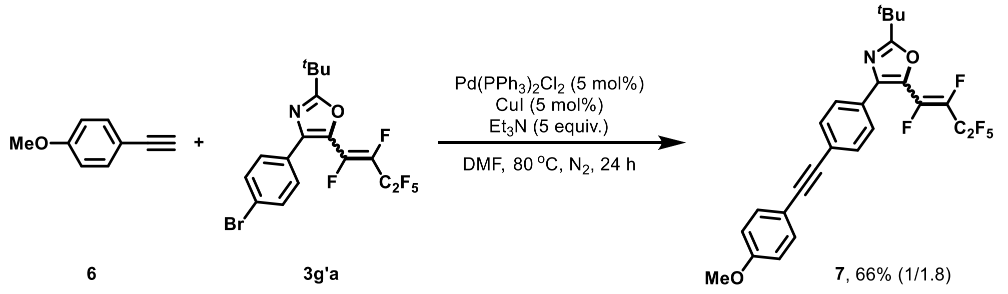

A solution of 4-(4-bromophenyl)-2-(*tert*-butyl)-5-(perfluorobut-1-en-1-yl)oxazole (138.1 mg, 0.3

mmol, 1 equiv., **3g'a**), 4-ethynylanisole (79.3 mg, 0.6 mmol, 2 equiv., **6**), $Pd(PPh_3)_2Cl_2$ (10.5 mg, 0.015 mmol, 0.05 equiv.), CuI (2.9 mg, 0.015 mmol, 0.05 equiv.), and Et_3N (151.8 mg, 1.5 mmol, 5 equiv.) in DMF (2 mL) was stirred at 80 °C under N_2 atmosphere for 24 h. The reaction was then quenched by saturated NH_4Cl solution (20 mL) and extracted with EtOAc (20 mL x 3). The organic layer was washed with saturated brine twice, dried over $MgSO_4$, and concentrated under reduced pressure. The crude product was purified by flash silica gel column chromatography (300-400 mesh) using petroleum ether/ethyl acetate (100/1) as eluent to afford the pure product **7** (101.1 mg, 66% yield).

3) Heck reaction of product 3g'a with methyl acrylate (8)

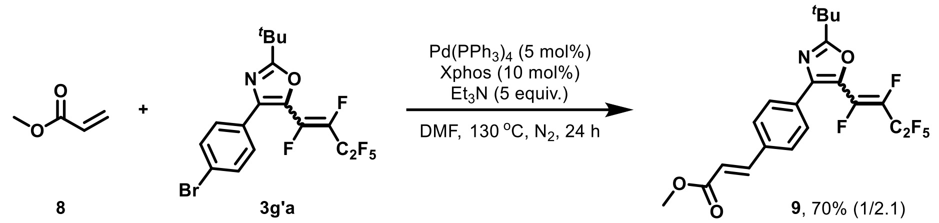

A solution of 4-(4-bromophenyl)-2-(*tert*-butyl)-5-(perfluorobut-1-en-1-yl)oxazole (138.1 mg, 0.3 mmol, 1 equiv., **3g'a**), methyl acrylate (51.7 mg, 0.9 mmol, 3 equiv., **8**), $Pd(PPh_3)_4$ (17.3 mg, 0.015 mmol, 0.05 equiv.), Xphos (14.3 mg, 0.03 mmol, 0.1 equiv.), and Et_3N (151.8 mg, 1.5 mmol, 5 equiv.) in DMF (2 mL) was stirred at 130 °C under N_2 atmosphere for 24 h. The reaction was then quenched by saturated NH_4Cl solution (20 mL) and extracted with EtOAc (20 mL x 3). The organic layer was washed with saturated brine twice, dried over $MgSO_4$, and concentrated under reduced pressure. The crude product was purified by flash silica gel column chromatography (300-400 mesh) using petroleum ether/ethyl acetate (50/1) as eluent to afford the pure product **9** (98.3 mg, 70% yield).

4) Buchwald-Hartwig coupling reaction of product 3g'a with norquetiapine·2HCl (10)

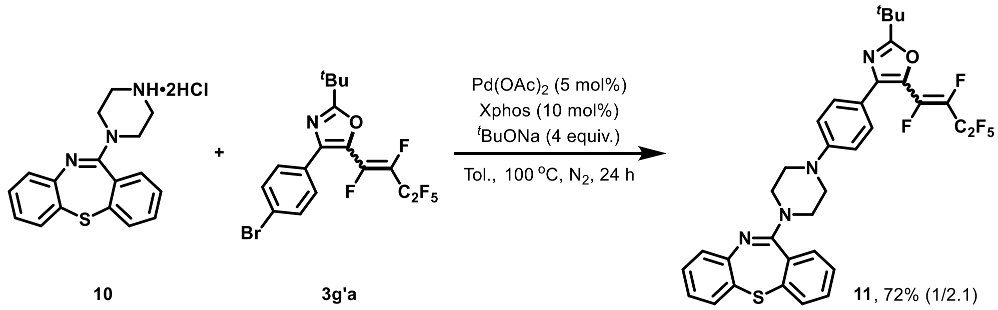

A solution of 4-(4-bromophenyl)-2-(*tert*-butyl)-5-(perfluorobut-1-en-1-yl)oxazole (138.1 mg, 0.3 mmol, 1 equiv., **3g'a**), norquetiapine·2HCl (221 mg, 0.6 mmol, 2 equiv., **10**), $Pd(OAc)_2$ (3.4 mg, 0.015 mmol, 0.05 equiv.), Xphos (14.3 mg, 0.03 mmol, 0.1 equiv.), and ^{t}BuONa (115.3 mg, 1.2 mmol, 4 equiv.) in toluene (2 mL) was stirred at 100 °C under N_2 atmosphere for 24 h. The reaction was then quenched by saturated NH_4Cl solution (20 mL) and extracted with EtOAc (20 mL x 3). The organic layer was washed with saturated brine twice, dried over $MgSO_4$, and concentrated under reduced pressure. The crude product was purified by flash silica gel column chromatography (300-400 mesh) using petroleum ether/ethyl acetate (20/1) as eluent to afford the pure product **11** (144.9 mg, 72% yield).

Mechanistic studies

1) Radical trapping experiment with 2,2,6,6-tetramethylpiperidin-1-oxyl (TEMPO)

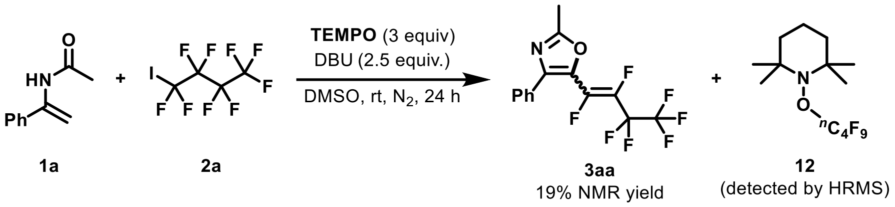

A solution of *N*-(1-phenylvinyl)acetamide (48.4 mg, 0.3 mmol, 1 equiv., **1a**), perfluorobutyl iodide (311.3 mg, 0.9 mmol, 3 equiv., **2a**), 1,8-diazabicyclo[5,4,0]undec-7-ene (114.2 mg, 0.75 mmol, 2.5 equiv., DBU), and 2,2,6,6-tetramethylpiperidin-1-oxyl (140.6 mg, 0.9 mmol, 3 equiv., TEMPO) in DMSO (2 mL) was stirred at room temperature under N_2 atmosphere for 24 h. Then the reaction mixture was passed through a short pad of Celite followed by rinse with MeCN. A sample was taken from the filtrate and was directly analyzed by HRMS. The reaction was then quenched by saturated NH_4Cl solution (20 mL) and extracted with EtOAc (20 mL x 3). The organic extract was dried over Na_2SO_4, filtered, and concentrated under reduced pressure. The residue was directly analyzed by NMR analysis. 19% NMR yield of 5-(perfluorobut-1-en-1-yl)-2,4-diphenyloxazole (**3aa**) was determined by ^{19}F NMR analysis of the residue using 1-fluoro-4-methoxybenzene (0.3 mmol) as an internal standard.

The TEMPO-adduct **12** was detected by HRMS analysis:

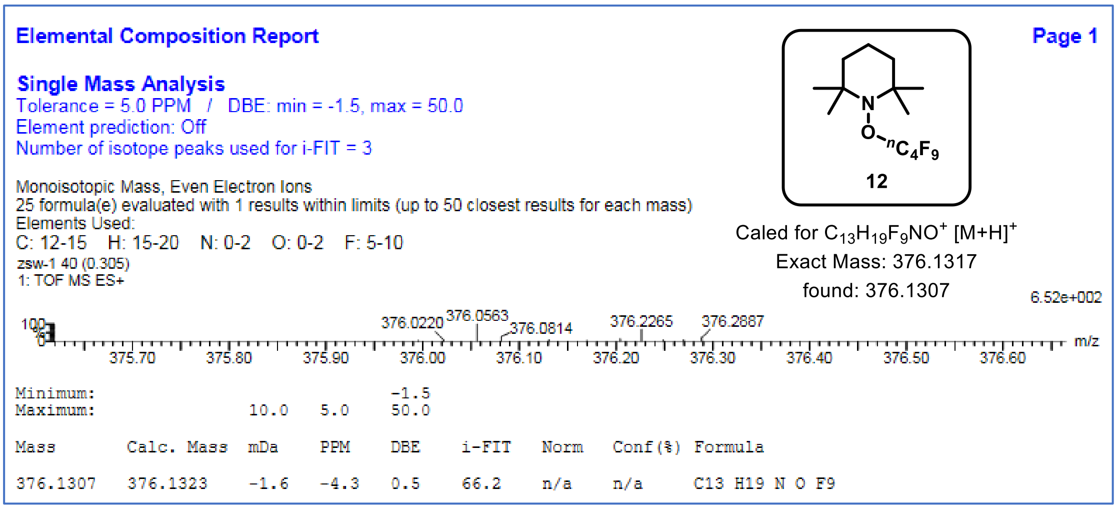
Elemental Composition Report — Page 1

Single Mass Analysis
Tolerance = 5.0 PPM / DBE: min = -1.5, max = 50.0
Element prediction: Off
Number of isotope peaks used for i-FIT = 3

Monoisotopic Mass, Even Electron Ions
25 formula(e) evaluated with 1 results within limits (up to 50 closest results for each mass)
Elements Used:
C: 12-15 H: 15-20 N: 0-2 O: 0-2 F: 5-10
zsw-1 40 (0.305)
1: TOF MS ES+

12
Calcd for $C_{13}H_{19}F_9NO^+$ [M+H]$^+$
Exact Mass: 376.1317
found: 376.1307

Minimum:				-1.5				
Maximum:		10.0	5.0	50.0				
Mass	Calc. Mass	mDa	PPM	DBE	i-FIT	Norm	Conf(%)	Formula
376.1307	376.1323	-1.6	-4.3	0.5	66.2	n/a	n/a	C13 H19 N O F9

Theses results suggested that $^{n}C_4F_9$ radical might be involved in the defluorinative cyclization process.

2) The generation of $^{n}C_4F_9H$

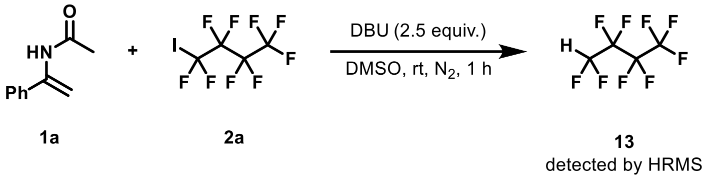

A solution of *N*-(1-phenylvinyl)acetamide (48.4 mg, 0.3 mmol, 1 equiv., **1a**), perfluorobutyl iodide (311.3 mg, 0.9 mmol, 3 equiv., **2a**), and 1,8-diazabicyclo[5,4,0]undec-7-ene (114.2 mg, 0.75 mmol,

2.5 equiv., DBU) in DMSO (2 mL) was stirred at room temperature under N_2 atmosphere for 1 h. Then the reaction mixture was passed through a short pad of Celite followed by rinse with MeCN. A sample was taken from the filtrate and was directly analyzed by HRMS.

The $^{n}C_4F_9H$ (**13**)[2] was detected by HRMS analysis:

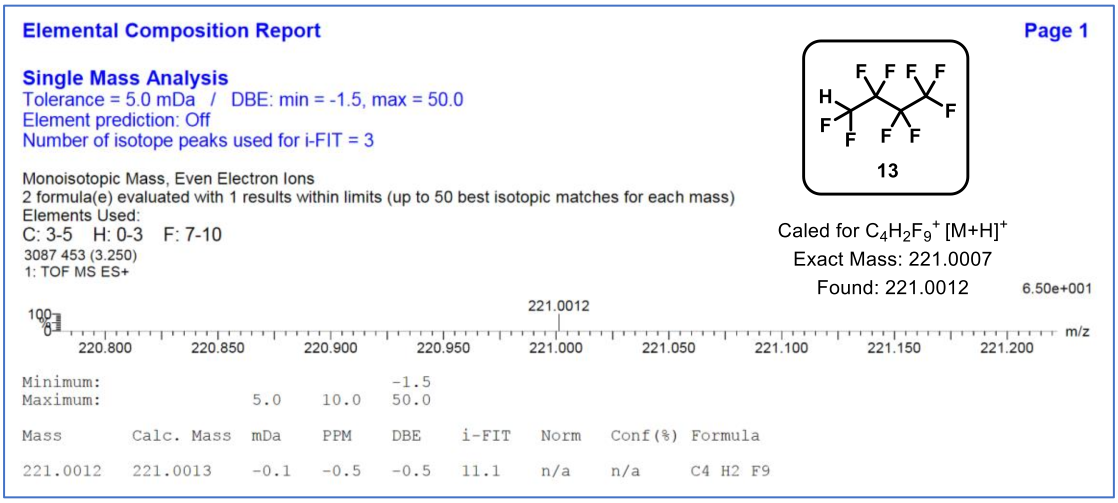
Elemental Composition Report

Page 1

Single Mass Analysis
Tolerance = 5.0 mDa / DBE: min = -1.5, max = 50.0
Element prediction: Off
Number of isotope peaks used for i-FIT = 3

Monoisotopic Mass, Even Electron Ions
2 formula(e) evaluated with 1 results within limits (up to 50 best isotopic matches for each mass)
Elements Used:
C: 3-5 H: 0-3 F: 7-10
3087 453 (3.250)
1: TOF MS ES+

Caled for $C_4H_2F_9^+$ [M+H]$^+$
Exact Mass: 221.0007
Found: 221.0012

Minimum:				-1.5				
Maximum:		5.0	10.0	50.0				
Mass	Calc. Mass	mDa	PPM	DBE	i-FIT	Norm	Conf(%)	Formula
221.0012	221.0013	-0.1	-0.5	-0.5	11.1	n/a	n/a	C4 H2 F9

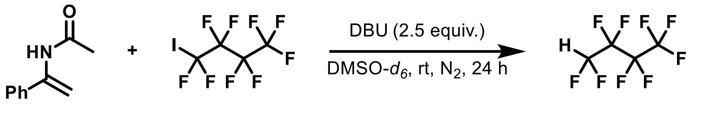

1a **2a** **13**, ~6% NMR yield

A solution of *N*-(1-phenylvinyl)acetamide (48.4 mg, 0.3 mmol, 1 equiv., **1a**), perfluorobutyl iodide (311.3 mg, 0.9 mmol, 3 equiv., **2a**), 4-fluoroanisole (37.8 mg, 0.3 mmol, 1 equiv.), and 1,8-diazabicyclo[5,4,0]undec-7-ene (114.2 mg, 0.75 mmol, 2.5 equiv., DBU) in DMSO-d_6 (2 mL) was stirred at room temperature under N_2 atmosphere for 24 h. ~6% NMR yield of $^{n}C_4F_9H$ (**13**) was determined by ^{19}F NMR analysis of the residue using 1-fluoro-4-methoxybenzene (0.3 mmol) as an internal standard.

^{19}F NMR analysis of the residue:

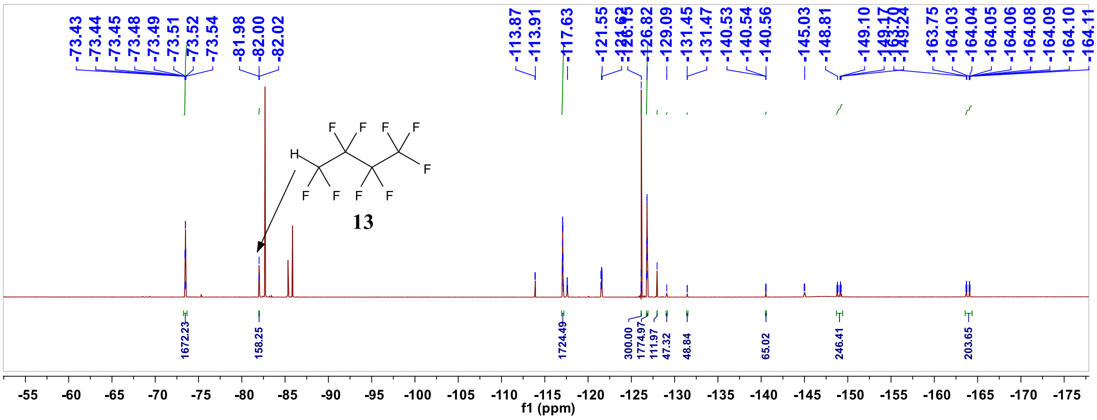

These results suggested that 1) $^{n}C_4F_9$ radical might be involved in the defluorinative cyclization process; 2) $^{n}C_4F_9$ radical might serve as a hydrogen abstractor; 3) other species might also serve as a hydrogen abstractor or oxidant.

3) The use of 1 equiv. of $^nC_4F_9I$

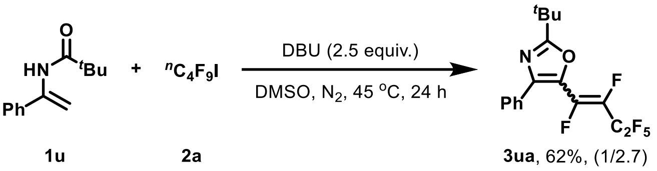

A solution of *N*-(1-phenylvinyl)pivalamide (61.0 mg, 0.3 mmol, 1 equiv., **1u**), polyfluoroalkyl iodide (103.8 mg, 0.3 mmol, 1 equiv., **2a**), and 1,8-diazabicyclo[5,4,0]undec-7-ene (114.2 mg, 0.75 mmol, 2.5 equiv., DBU) in DMSO (2.0 mL) was stirred at 45 °C under N_2 atmosphere for 24 h. The reaction was then quenched by saturated NH_4Cl solution (20 mL) and extracted with EtOAc (20 mL x 3). The organic layer was washed with saturated brine twice, dried over $MgSO_4$, filtered, and concentrated under reduced pressure. The crude product was purified by flash silica gel column chromatography (300-400 mesh) using petroleum ether/ethyl acetate (50/1) as eluent to afford the pure product **3ua** (70.5 mg, 62% yield).

This result suggested that an alternative base-promoted $S_{RN}1$ pathway might be involved; 2) other species might also serve as a hydrogen abstractor or oxidant.

4) The influence of *N*-substituent of enamide 1

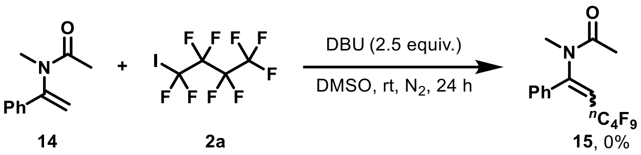

A solution of *N*-methyl-*N*-(1-phenylvinyl)acetamide[3] (52.6 mg, 0.3 mmol, 1 equiv., **14**), perfluorobutyl iodide (311.3 mg, 0.9 mmol, 3 equiv., **2a**), and 1,8-diazabicyclo[5,4,0]undec-7-ene (114.2 mg, 0.75 mmol, 2.5 equiv., DBU) in DMSO (2 mL) was stirred at room temperature under N_2 atmosphere for 24 h. No desired product **15** was obtained.

This result suggested NH moiety on enamide 1 is essential for the initial perfluoroalkylation.

5) Exclusion of oxazole intermediate 16

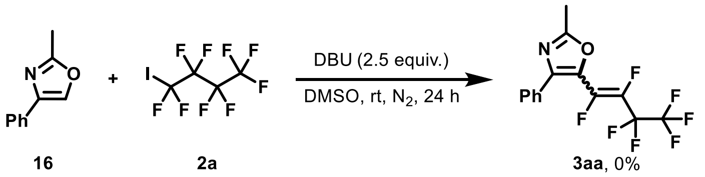

A solution of 2-methyl-4-phenyloxazole[4] (47.8 mg, 0.3 mmol, 1 equiv., **16**), perfluorobutyl iodide (311.3 mg, 0.9 mmol, 3 equiv., **2a**), and 1,8-diazabicyclo[5,4,0]undec-7-ene (114.2 mg, 0.75 mmol, 2.5 equiv., DBU) in DMSO (2 mL) was stirred at room temperature under N_2 atmosphere for 24 h. No desired product **3aa** was obtained.

This result suggested that oxazole 14 is not a reaction intermediate.

6) The reaction of perfluorobutyl alkyne with amide

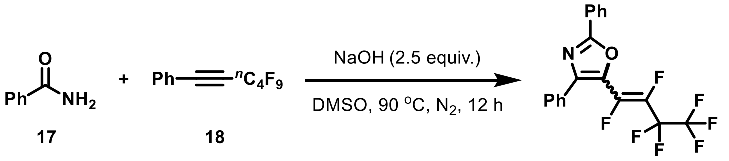

17 18

3aa, ~10% NMR yield

A solution of benzamide (36.3 mg, 0.3 mmol, 1 equiv., **17**), (perfluorohex-1-yn-1-yl)benzene[5] (144.1 mg, 0.45 mmol, 1.5 equiv., **18**), and NaOH (30 mg, 0.75 mmol, 2.5 equiv.) in DMSO (2 mL) was stirred at 90 °C under N_2 atmosphere for 12 h. The vial was then cooled to room temperature and the reaction mixture was quenched by saturated NH_4Cl solution (20 mL) followed by extraction with EtOAc (20 mL x 3). The organic layer was washed with saturated brine twice, dried over $MgSO_4$, and concentrated under reduced pressure. The residue was directly analyzed by NMR analysis. ~10% NMR yield of 5-(perfluorobut-1-en-1-yl)-2,4-diphenyloxazole (**3aa**) was determined by ^{19}F NMR analysis of the residue using 1-fluoro-4-methoxybenzene (0.3 mmol) as an internal standard.

The suggested result is that the perfluoroalkylation event occurs before the defluorinative cyclization.

7) The necessity of CF_2CF_2 moiety on the fluoroalkyl halides

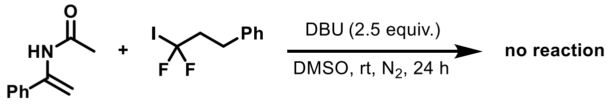

1a **19**

A solution of *N*-(1-phenylvinyl)acetamide (48.4 mg, 0.3 mmol, 1 equiv., **1a**), (3,3-difluoro-3-iodopropyl)benzene (253.9 mg, 0.9 mmol, 3 equiv., **19**[6]), and 1,8-diazabicyclo[5,4,0]undec-7-ene (114.2 mg, 0.75 mmol, 2.5 equiv., DBU) in DMSO (2 mL) was stirred at room temperature under N_2 atmosphere for 24 h. No desired product was obtained.

This result suggested that CF_2CF_2 moiety on the fluoroalkyl halides is necessary for the successful defluorinative transformation.

8) The effect of reaction time on the *Z*/*E* ratio of product 3ua

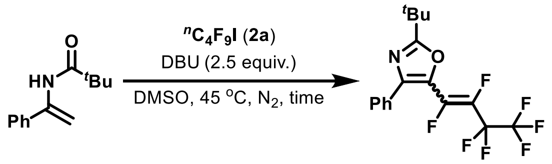

1u **3ua**

time (h)	1	3	6	12	24	36	48
Z/*E* of **3ua**	1/2.3	1/2.3	1/2.3	1/2.4	1/2.4	1/2.3	1/2.4

A solution of *N*-(1-phenylvinyl)pivalamide (61.0 mg, 0.3 mmol, 1 equiv., **1u**), polyfluoroalkyl iodide (311.3 mg, 0.9 mmol, 3 equiv., **2a**), and 1,8-diazabicyclo[5,4,0]undec-7-ene (114.2 mg, 0.75 mmol, 2.5 equiv., DBU) in DMSO (2.0 mL) was stirred at 45 °C under N_2 atmosphere for 1-48 h. The reaction was then quenched by saturated NH_4Cl solution (20 mL) and extracted with EtOAc (20 mL x 3). The organic layer was washed with saturated brine twice, dried over $MgSO_4$, filtered, and concentrated under reduced pressure. The ratio of *Z*/*E* was determined by ^{19}F NMR analysis of the residue using 1-fluoro-4-methoxybenzene (0.3 mmol) as an internal standard.

This result suggested that the reaction time almost has no influence on the reaction

stereoselectivity.

9) Halogen-bonded adduct DBU·(^{n}C$_4$F$_9$I)$_2$ as the reagent

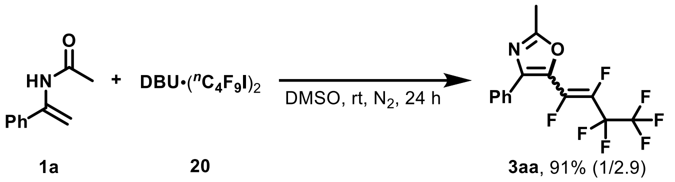

A solution of *N*-(1-phenylvinyl)acetamide (48.4 mg, 0.3 mmol, 1 equiv., **1a**) and DBU·(^{n}C$_4$F$_9$I)$_2$ (633.1 mg, 0.75 mmol, 2.5 equiv., **20**) in DMSO (2 mL) was stirred at room temperature under N_2 atmosphere for 24 h. The reaction was then quenched by saturated NH_4Cl solution (20 mL) and extracted with EtOAc (20 mL x 3). The organic layer was washed with saturated brine twice, dried over $MgSO_4$, filtered, and concentrated under reduced pressure. The crude product was purified by flash column chromatography (300-400 mesh) using petroleum ether/ethyl acetate (100/1) as eluent to afford the pure product **3aa** (93 mg, 91% yield).

This result suggested that the reaction might start with the in situ formation of DBU·(^{n}C$_4$F$_9$I)$_2$ adduct.

10) ^{19}F NMR titration experiment of DBU with ^{n}C$_4$F$_9$I[7]

^{19}F NMR spectra of four samples of mixtures of ^{n}C$_4$F$_9$I and DBU in DMSO-d_6 were recorded at 298 K. The total volume of the mixture was 0.6 mL, and the amount of ^{n}C$_4$F$_9$I was kept constant at 0.02 mmol (3.3 µL) while that of DBU was varied from 0 to 0.2 mmol (the molar ratios of ^{n}C$_4$F$_9$I : DBU were 0:0, 1:1, and 1:10). $PhOCF_3$ (δ = -57.8) was used as internal standard.

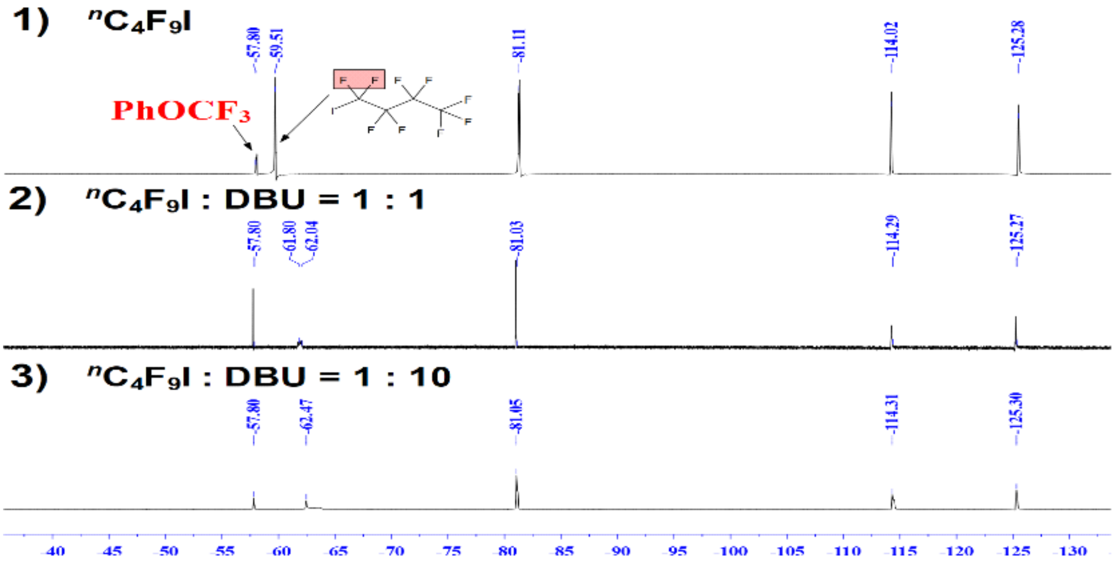

The ^{19}F NMR signal of CF$_2$I moiety shifted upfield when the amount of DBU increased, indicating the formation of halogen bonding adduct.

11) UV-Vis spectroscopic measurement[8]

UV-Vis absorption of different compound combinations. Each component was mixed in a ratio that reflects the actual reaction setup. The concentrations are 10-fold lower compared to the actual reaction setup.

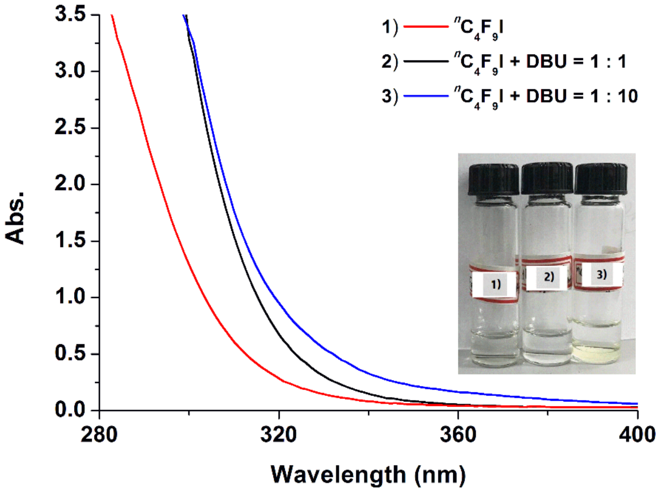

A red shift of absorption and color change were observed when ${}^{n}C_4F_9I$ and DBU were combined in DMSO, indicating the formation of halogen bonding adduct between ${}^{n}C_4F_9I$ and DBU.

Optimization of reaction conditions

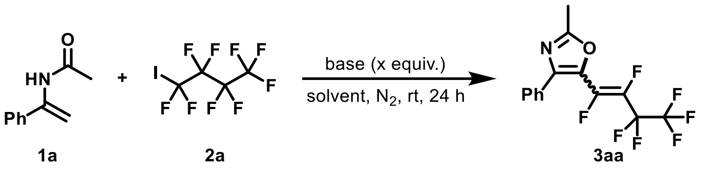

Entry	Base (x equiv.)	Solvent	Yield of **3aa** (%)[b]
1	Cs_2CO_3 (2)	MeCN	trace
2	Cs_2CO_3 (2)	DCE	trace
3	Cs_2CO_3 (2)	DME	trace
4	Cs_2CO_3 (2)	THF	trace
5	Cs_2CO_3 (2)	1,4-dioxane	trace
6	Cs_2CO_3 (2)	DMSO	46 (1/2.6)[c]
7	Cs_2CO_3 (2)	toluene	trace
8	Cs_2CO_3 (2)	EtOH	trace
9	Cs_2CO_3 (2)	EtOAc	trace
10	Cs_2CO_3 (2)	DMF	24 (1/1.5)[c]
11	Cs_2CO_3 (2)	DMA	17 (1/2.5)[c]
12	Cs_2CO_3 (2)	NMP	32 (1/2)[c]
13	Cs_2CO_3 (2)	^{t}BuOH	trace
14	Cs_2CO_3 (2)	cyclohexane	trace
15	K_3PO_4 (2.5)	DMSO	16 (1/6)[c]
16	$NaHCO_3$ (2.5)	DMSO	trace
17	NaOAc (2.5)	DMSO	trace
18	K_2CO_3 (2.5)	DMSO	31 (1/3)[c]
19	DMAP (2)	DMSO	trace
20	Et_3N (2)	DMSO	trace
21	PMDETA (2)	DMSO	trace
22	TMEDA (2)	DMSO	trace
23	DABCO (2)	DMSO	trace
24	DBU (2)	DMSO	81 (1/3)[c]
25	NaOH (2)	DMSO	0
26	Quinine (2)	DMSO	trace
**27**	**DBU** (2.5)	**DMSO**	**93[d] (1/2.9)[c]**
28	DBU (2.5)	DMSO	93[d,e] (1/2.9)[c]
29	DBU (2.5)	MeCN	<5[d]
30	DBU (2.5)	THF	<5[d]

[a] Reaction conditions: **1a** (0.3 mmol), **2a** (0.6 mmol), and base (0.6-0.75 mmol) in solvent (2 mL) at room temperature under N_2 for 24 h. [b] Isolated yields. [c] Ratio of *Z*/*E* in parentheses was determined by ^{19}F NMR analysis. [d] **2a** (0.9 mmol) was used. [e] At 45 °C.

Characterization data for products

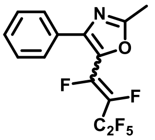

2-Methyl-5-(perfluorobut-1-en-1-yl)-4-phenyloxazole (3aa):
Yield = 93% (95 mg, *Z*/*E* = 1/2.9). Light yellow oil.
IR (KBr): ν = 2959, 1464, 1218, 804 cm^{-1}.
^{1}H NMR (400 MHz, $CDCl_3$): δ = 7.73–7.61 (m, 2H), 7.49–7.35 (m, 3H), 2.62–2.51 (m, 3H) ppm.
^{19}F NMR (376 MHz, $CDCl_3$) of (*E*)-isomer: δ = -84.19 (tq, *J* = 5.8, 2.9 Hz, 3F), -120.06 (ddq, *J* = 21.7, 12.3, 2.5 Hz, 2F), -145.86 – -146.78 (m, 1F), -160.04 (dtt, *J* = 137.3, 13.7, 4.3 Hz, 1F) ppm; **(*Z*)-isomer:** δ = -83.48 (dt, *J* = 6.5, 2.9 Hz, 3F), -109.95 (d, *J* = 14.4 Hz, 1F), -119.72 (dt, *J* = 16.6, 3.3 Hz, 2F), -140.96 (qd, *J* = 15.2, 13.7 Hz, 1F) ppm.
^{13}C NMR (100 MHz, $CDCl_3$) of (*E*)-isomer: δ = 164.0 (d, *J* = 2.7 Hz), 147.4–144.9 (m, 1C), 144.3 (d, *J* = 2.8 Hz), 129.7, 129.6–129.4 (m, 1C), 129.0, 128.7, 127.97, 127.95, 14.2 ppm; carbons corresponding to the C_2F_5 group cannot be identified due to C-F coupling.
HRMS (m/z): calcd for $C_{14}H_9F_7NO$ $[M+H]^+$ 340.0567, found: 340.0573.

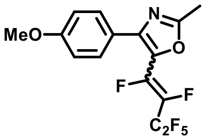

4-(4-Methoxyphenyl)-2-methyl-5-(perfluorobut-1-en-1-yl)oxazole (3ba):
Yield = 51% (57 mg, *Z*/*E* = 1/4.1). Light yellow oil.
IR (KBr): ν = 2963, 1578, 1219, 1033, 837 cm^{-1}.
^{1}H NMR (400 MHz, $CDCl_3$): δ = 7.66–7.56 (m, 2H), 6.97–6.92 (m, 2H), 3.84 (s, 3H), 2.58–2.52 (m, 3H) ppm.
^{19}F NMR (376 MHz, $CDCl_3$) of (*E*)-isomer: δ = -84.15 – -84.33 (m, 3F), -119.84 – -120.23 (m, 2F), -146.03 – -147.13 (m, 1F), -160.54 (dddt, *J* = 137.4, 13.7, 9.2, 4.7 Hz, 1F) ppm; **(*Z*)-isomer:** δ = -83.54 (dt, *J* = 6.2, 2.9 Hz, 3F), -110.08 (dd, *J* = 15.1, 2.5 Hz, 1F), -119.71 (dq, *J* = 16.3, 2.7 Hz, 2F), -141.47 (dq, *J* = 16.0, 8.2 Hz, 1F) ppm.
^{13}C NMR (100 MHz, $CDCl_3$) of (*E*)-isomer: δ = 163.8 (d, *J* = 2.7 Hz), 160.7, 147.5–146.7 (m, 1C), 144.1 (d, *J* = 3.3 Hz), 131.5–130.5 (m, 1C), 129.4, 129.3, 122.5, 114.2, 55.4, 14.2 ppm; carbons corresponding to the C_2F_5 group cannot be identified due to C-F coupling.
HRMS (m/z): calcd for $C_{15}H_{11}F_7NO_2$ $[M+H]^+$ 370.0673, found: 370.0678.

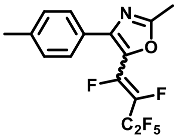

2-Methyl-5-(perfluorobut-1-en-1-yl)-4-(*p*-tolyl)oxazole (3ca):
Yield = 43% (46 mg, *Z*/*E* = 1/4). Light yellow oil.
IR (KBr): ν = 2929, 1705, 1328, 1041, 824, 741 cm^{-1}.
^{1}H NMR (400 MHz, $CDCl_3$): δ = 7.56–7.51 (m, 2H), 7.26–7.21 (m, 2H), 2.58 (s, 3H), 2.39 (s, 3H) ppm.
^{19}F NMR (376 MHz, $CDCl_3$) of (*E*)-isomer: δ = -84.22 (ddd, *J* = 6.8, 4.9, 3.2 Hz, 3F), -120.09 (ddq, *J* = 22.5, 13.2, 3.0 Hz, 2F), -146.30 (dddt, *J* = 137.6, 25.7, 20.6, 5.0 Hz, 1F), -160.16 (ddtd, *J* = 137.9, 17.9, 8.9, 4.4 Hz, 1F) ppm; **(*Z*)-isomer:** δ = -83.50 – -83.55 (m, 3F), -110.06 – -110.15 (m, 1F), -119.74 (dt, *J* = 16.4, 2.7 Hz, 2F), -141.21 – -141.42 (m, 1F) ppm.
^{13}C NMR (100 MHz, $CDCl_3$) of (*E*)-isomer: δ = 163.9 (d, *J* = 2.5 Hz), 147.4–146.6 (m, 1C), 144.3 (d, *J* = 3.0 Hz), 139.8, 132.1–131.0 (m, 1C), 129.5, 127.77, 127.75, 127.1, 21.5, 14.2 ppm; carbons corresponding to the C_2F_5 group cannot be identified due to C-F coupling.
HRMS (m/z): calcd for $C_{15}H_{11}F_7NO$ $[M+H]^+$ 354.0723, found: 354.0729.

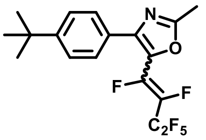

4-(4-(*tert*-Butyl)phenyl)-2-methyl-5-(perfluorobut-1-en-1-yl)oxazole (3da):

Yield = 65% (77 mg, *Z*/*E* = 1/5.6). Light yellow oil.

IR (KBr): ν = 2965, 1709, 1582, 1219, 923, 842 cm^{-1}.

^{1}H NMR (400 MHz, $CDCl_3$): δ = 7.67–7.57 (m, 2H), 7.48–7.43 (m, 2H), 2.60–2.54 (m, 3H), 1.38–1.33 (m, 9H) ppm.

^{19}F NMR (376 MHz, $CDCl_3$) of (*E*)-isomer: δ = -84.20 (tt, *J* = 4.8, 2.6 Hz, 3F), -120.02 (ddq, *J* = 25.9, 13.4, 3.0 Hz, 2F), -146.28 (dtd, *J* = 137.5, 25.7, 4.5 Hz, 1F), -159.95 – -160.87 (m, 1F) ppm; **(*Z*)-isomer:** δ = -83.47 (dq, *J* = 5.6, 2.8 Hz, 3F), -110.13 – -110.28 (m, 1F), -119.74 (dt, *J* = 16.6, 2.7 Hz, 2F), -141.43 (ddddd, *J* = 14.6, 12.0, 10.0, 7.4, 2.9 Hz, 1F) ppm.

^{13}C NMR (100 MHz, $CDCl_3$) of (*E*)-isomer: δ = 163.8 (d, *J* = 2.5 Hz), 152.9, 144.6–144.0 (m, 1C), 127.7, 127.6, 127.0, 126.9–126.7 (m, 1C), 126.0, 125.7, 34.9, 31.3, 14.2 ppm; carbons corresponding to the C_2F_5 group cannot be identified due to C-F coupling.

HRMS (m/z): calcd for $C_{18}H_{17}F_7NO$ $[M+H]^+$ 396.1193, found: 396.1195.

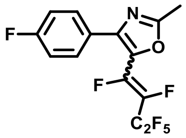

4-(4-Fluorophenyl)-2-methyl-5-(perfluorobut-1-en-1-yl)oxazole (3ea):

Yield = 64% (68.4 mg, *Z*/*E* = 1/2.8). Light yellow oil.

IR (KBr): ν = 2918, 1735, 1223, 1040, 886 cm^{-1}.

^{1}H NMR (400 MHz, $CDCl_3$): δ = 7.71–7.59 (m, 2H), 7.16–7.08 (m, 2H), 2.59–2.53 (m, 3H) ppm.

^{19}F NMR (376 MHz, $CDCl_3$) of (*E*)-isomer: δ = -84.15 – -84.48 (m, 3F), -110.91 – -110.98 (m, 1F), -120.04 – -120.31 (m, 2F), -147.07 (dddd, *J* = 137.6, 31.4, 20.7, 5.3 Hz, 1F), -160.01 (dddt, *J* = 137.6, 13.1, 8.6, 4.4 Hz, 1F) ppm; **(*Z*)-isomer:** δ = -83.61 (dd, *J* = 7.0, 3.4 Hz, 3F), -110.51 (d, *J* = 14.1 Hz, 1F), -110.86 – -110.91 (m, 1F), -119.54 – -119.89 (m, 2F), -140.44 – -141.20 (m, 1F) ppm.

^{13}C NMR (100 MHz, $CDCl_3$) of (*E*)-isomer: δ = 164.4 (d, *J* = 68.9 Hz), 163.2 (d, *J* = 178.5 Hz), 147.5–145.8 (m, 1C), 143.3 (d, *J* = 3.1 Hz), 132.4–131.2 (m, 1C), 123.0 (d, *J* = 3.1 Hz), 129.9 (d, *J* = 3.0 Hz), 126.2 (d, *J* = 3.4 Hz), 115.9 (d, *J* = 22.0 Hz), 14.1 ppm; carbons corresponding to the C_2F_5 group cannot be identified due to C-F coupling.

HRMS (m/z): calcd for $C_{14}H_8F_8NO$ $[M+H]^+$ 358.0473, found: 358.0480.

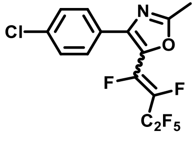

4-(4-Chlorophenyl)-2-methyl-5-(perfluorobut-1-en-1-yl)oxazole (3fa):

Yield = 51% (56.8 mg, *Z*/*E* = 1/5.7). Light yellow oil.

IR (KBr): ν = 2927, 1587, 1410, 1222, 982, 802 cm^{-1}.

^{1}H NMR (400 MHz, $CDCl_3$): δ = 7.66–7.55 (m, 2H), 7.43–7.37 (m, 2H), 2.59–2.53 (m, 3H) ppm.

^{19}F NMR (376 MHz, $CDCl_3$) of (*E*)-isomer: δ = -84.24 (t, *J* = 4.1 Hz, 3F), -120.00 – -120.29 (m, 2F), -146.93 (dddd, *J* = 137.6, 31.4, 20.6, 5.4 Hz, 1F), -159.58 (dtt, *J* = 137.7, 13.5, 4.6 Hz, 1F) ppm; **(*Z*)-isomer:** δ = -83.48 – -83.62 (m, 3F), -110.71 (d, *J* = 14.1 Hz, 1F), -119.48 – -119.85 (m, 2F), -140.27 – -140.64 (m, 1F) ppm.

^{13}C NMR (100 MHz, $CDCl_3$) of (*E*)-isomer: δ = 164.1 (d, *J* = 2.5 Hz), 147.3–145.8 (m, 1C), 143.1 (d, *J* = 3.2 Hz), 135.7, 132.6–131.7 (m, 1C), 129.23, 129.20, 129.0 (2C), 14.1 ppm; carbons corresponding to the C_2F_5 group cannot be identified due to C-F coupling.

HRMS (m/z): calcd for $C_{14}H_8ClF_7NO$ $[M+H]^+$ 374.0177, found: 374.0183.

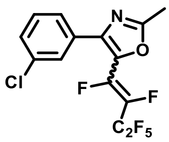

4-(3-Chlorophenyl)-2-methyl-5-(perfluorobut-1-en-1-yl)oxazole (3ga):
Yield = 97% (109 mg, *Z*/*E* = 1/2.8). Light yellow oil.
IR (KBr): ν = 2928, 1707, 1217, 984, 764 cm^{-1}.
^{1}H NMR (400 MHz, CDCl$_3$): δ = 7.53–7.43 (m, 2H), 7.39–7.30 (m, 2H), 2.64–2.55 (m, 3H) ppm.
^{19}F NMR (376 MHz, CDCl$_3$) of (*E*)-isomer: δ = -84.31 – -84.41 (m, 3F), -120.03 – -120.23 (m, 2F), -150.73 – -151.37 (m, 1F), -162.65 (dddt, *J* = 135.5, 13.3, 8.8, 4.4 Hz, 1F) ppm; **(*Z*)-isomer:** δ = -83.27 – -83.56 (m, 3F), -116.14 (d, *J* = 11.5 Hz, 1F), -119.23 (dd, *J* = 16.4, 2.2 Hz, 2F), -144.87 (ttd, *J* = 16.6, 8.0, 3.7 Hz, 1F) ppm.
^{13}C NMR (100 MHz, CDCl$_3$) of (*E*)-isomer: δ = 163.8 (d, *J* = 3.2 Hz), 141.0–140.9 (m, 1C), 133.4–133.3 (m, 1C), 131.2, 130.83, 130.76, 130.3, 130.0, 129.7, 127.1, 126.9, 14.2 ppm; carbons corresponding to the C_2F_5 group cannot be identified due to C-F coupling.
HRMS (m/z): calcd for $C_{14}H_8ClF_7NO$ [M+H]$^+$ 374.0177, found: 374.0186.

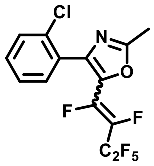

4-(2-Chlorophenyl)-2-methyl-5-(perfluorobut-1-en-1-yl)oxazole (3ha):
Yield = 55% (62.1 mg, *Z*/*E* = 1/2.9). Light yellow oil.
IR (KBr): ν = 2909, 1725, 1578, 1221, 1039, 632 cm^{-1}.
^{1}H NMR (400 MHz, CDCl$_3$): δ = 7.51–7.44 (m, 2H), 7.38–7.30 (m, 2H), 2.63–2.56 (m, 3H) ppm.
^{19}F NMR (376 MHz, CDCl$_3$) of (*E*)-isomer: δ = -84.36 (t, *J* = 4.3 Hz, 3F), -119.85 – -120.60 (m, 2F), -150.64 – -151.71 (m, 1F), -162.11 – -163.07 (m, 1F) ppm; **(*Z*)-isomer:** δ = -83.44 (dd, *J* = 6.6, 4.0 Hz, 3F), -116.14 (d, *J* = 11.6 Hz, 1F), -119.23 (d, *J* = 17.9 Hz, 2F), -144.52 – -145.52 (m, 1F) ppm.
^{13}C NMR (100 MHz, CDCl$_3$) of (*E*)-isomer: δ = 163.84, 163.79 (d, *J* = 2.7 Hz), 147.6–141.7 (m, 1C), 141.0, 138.7–134.4 (m, 1C), 133.4, 131.2, 130.8, 130.0, 129.7, 126.9, 14.2 ppm; carbons corresponding to the C_2F_5 group cannot be identified due to C-F coupling.
HRMS (m/z): calcd for $C_{14}H_8ClF_7NO$ [M+H]$^+$ 374.0177, found: 374.0182.

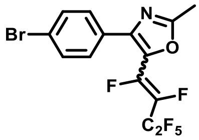

4-(4-Bromophenyl)-2-methyl-5-(perfluorobut-1-en-1-yl)oxazole (3ia):
Yield = 59% (73.5 mg, *Z*/*E* = 1/2.5). White solid.
IR (KBr): ν = 2962, 1727, 1407, 832, 723 cm^{-1}.
^{1}H NMR (400 MHz, CDCl$_3$): δ = 7.58–7.48 (m, 4H), 2.59–2.52 (m, 3H) ppm.
^{19}F NMR (376 MHz, CDCl$_3$) of (*E*)-isomer: δ = -84.12 – -84.47 (m, 3F), -120.06 – -120.27 (m, 2F), -146.84 (dtd, *J* = 137.8, 25.9, 5.1 Hz, 1F), -158.90 – -159.81 (m, 1F) ppm; **(*Z*)-isomer:** δ = -83.60 (d, *J* = 8.3 Hz, 3F), -110.81 (d, *J* = 13.5 Hz, 1F), -119.75 (d, *J* = 16.6 Hz, 2F), -140.49 (ddt, *J* = 22.4, 14.9, 8.0 Hz, 1F) ppm.
^{13}C NMR (100 MHz, CDCl$_3$) of (*E*)-isomer: δ = 164.1 (d, *J* = 2.6 Hz), 147.2–144.2 (m, 1C), 143.1 (d, *J* = 3.3 Hz), 132.0, 129.5, 129.4, 128.60, 128.58, 124.0, 14.1 ppm; carbons corresponding to the C_2F_5 group cannot be identified due to C-F coupling.
HRMS (m/z): calcd for $C_{14}H_8BrF_7NO$ [M+H]$^+$ 417.9672, found: 417.9675.

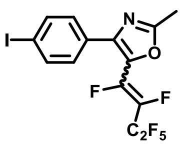

4-(4-Iodophenyl)-2-methyl-5-(perfluorobut-1-en-1-yl)oxazole (3ja):
Yield = 89% (124 mg, *Z*/*E* = 1/2.8). Light yellow oil.
IR (KBr): ν = 2929, 1689, 1584, 980, 828, 720 cm^{-1}.
^{1}H NMR (400 MHz, $CDCl_3$): δ = 7.80–7.74 (m, 2H), 7.45–7.34 (m, 2H), 2.58–2.53 (m, 3H) ppm.
^{19}F NMR (376 MHz, $CDCl_3$) of (*E*)-isomer: δ = -84.17 – -84.27 (m, 3F), -120.13 (ddq, *J* = 26.3, 13.6, 3.3 Hz, 2F), -146.83 (dddt, *J* = 138.0, 26.3, 21.0, 5.6 Hz, 1F), -159.42 (ddtd, *J* = 137.8, 18.4, 9.4, 4.7 Hz, 1F) ppm; **(*Z*)-isomer:** δ = -83.38 – -83.70 (m, 3F), -110.48 – -110.92 (m, 1F), -119.62 – -119.82 (m, 2F), -140.39 (ddq, *J* = 21.8, 9.7, 7.2 Hz, 1F) ppm.
^{13}C NMR (100 MHz, $CDCl_3$) of (*E*)-isomer: δ = 164.1 (d, *J* = 2.6 Hz), 144.5–143.8 (m, 1C), 143.2 (d, *J* = 3.0 Hz), 138.8–138.3 (m, 1C), 138.2, 137.9, 129.50, 129.48, 95.9, 14.2 ppm; carbons corresponding to the C_2F_5 group cannot be identified due to C-F coupling.
HRMS (m/z): calcd for $C_{14}H_8F_7INO$ $[M+H]^+$ 465.9533, found: 465.9539.

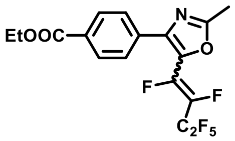

Ethyl 4-(2-methyl-5-(perfluorobut-1-en-1-yl)oxazol-4-yl)benzoate (3ka):
Yield = 69% (85 mg, *Z*/*E* = 1/3.5). White solid.
IR (KBr): ν = 2928, 1719, 1412, 864, 616 cm^{-1}.
^{1}H NMR (400 MHz, $CDCl_3$): δ = 8.16–8.05 (m, 2H), 7.80–7.66 (m, 2H), 4.44–4.33 (m, 2H), 2.62–2.53 (m, 3H), 1.44–1.34 (m, 3H) ppm.
^{19}F NMR (376 MHz, $CDCl_3$) of (*E*)-isomer: δ = -84.08 – -84.32 (m, 3F), -120.17 (ddd, *J* = 23.2, 11.8, 6.9 Hz, 2F), -146.02 – -146.78 (m, 1F), -158.58 – -159.31 (m, 1F) ppm; **(*Z*)-isomer:** δ = -83.45 – -83.66 (m, 3F), -110.72 (d, *J* = 12.1 Hz, 1F), -119.73 (d, *J* = 15.9 Hz, 2F), -140.11 (ddt, *J* = 21.5, 17.4, 7.7 Hz, 1F) ppm.
^{13}C NMR (100 MHz, $CDCl_3$) of (*E*)-isomer: δ = 166.1, 164.2 (d, *J* = 2.4 Hz), 146.9–143.0 (m, 1C), 134.1, 133.6–132.2 (m, 1C), 131.3, 130.2, 129.9, 127.82, 127.80, 61.3, 14.4, 14.2 ppm; carbons corresponding to the C_2F_5 group cannot be identified due to C-F coupling.
HRMS (m/z): calcd for $C_{17}H_{13}F_7NO_3$ $[M+H]^+$ 412.0778, found: 412.0782.

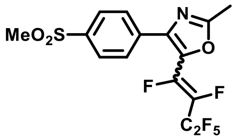

2-Methyl-4-(4-(methylsulfonyl)phenyl)-5-(perfluorobut-1-en-1-yl)oxazole (3la):
Yield = 49% (61 mg, *Z*/*E* = 1/4.2). White solid.
IR (KBr): ν = 3018, 2923, 1717, 1585, 1410, 776, 557 cm^{-1}.
^{1}H NMR (400 MHz, $CDCl_3$): δ = 8.05–7.97 (m, 2H), 7.93–7.81 (m, 2H), 3.08 (s, 3H), 2.63–2.56 (m, 3H) ppm.
^{19}F NMR (376 MHz, $CDCl_3$) of (*E*)-isomer: δ = -84.13 (tt, *J* = 5.2, 3.2 Hz, 3F), -120.16 (ddq, *J* = 25.7, 13.0, 3.0 Hz, 2F), -146.89 – -147.53 (m, 1F), -158.51 (dddt, *J* = 137.6, 13.0, 8.7, 4.5 Hz, 1F) ppm; **(*Z*)-isomer:** δ = -83.46 (dt, *J* = 6.4, 2.7 Hz, 3F), -111.39 – -111.49 (m, 1F), -119.69 (dq, *J* = 16.5, 2.6 Hz, 2F), -139.33 (dddt, *J* = 16.4, 12.9, 9.2, 7.0 Hz, 1F) ppm.
^{13}C NMR (100 MHz, $CDCl_3$) of (*E*)-isomer: δ = 164.5 (d, *J* = 2.5 Hz), 147.3–143.5 (m, 1C), 142.0 (d, *J* = 3.1 Hz), 141.1, 135.4, 134.8–133.2 (m, 1C), 128.9, 128.2, 127.8, 44.6, 14.2 ppm; carbons corresponding to the C_2F_5 group cannot be identified due to C-F coupling.
HRMS (m/z): calcd for $C_{15}H_{11}F_7NO_3S$ $[M+H]^+$ 418.0342, found: 418.0345.

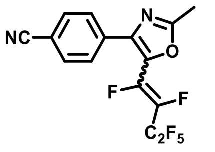

4-(2-Methyl-5-(perfluorobut-1-en-1-yl)oxazol-4-yl)benzonitrile (3ma):
Yield = 75% (82 mg, *Z*/*E* = 1/4.1). White solid.
IR (KBr): ν = 2918, 1719, 1577, 1041, 745 cm^{-1}.
^{1}H NMR (400 MHz, CDCl$_3$): δ = 7.85–7.75 (m, 1H), 7.75–7.69 (m, 3H), 2.62–2.56 (m, 3H) ppm.
^{19}F NMR (376 MHz, CDCl$_3$) of (*E*)-isomer: δ = -84.16 (dq, *J* = 7.9, 3.3 Hz, 3F), -120.19 (ddq, *J* = 25.3, 12.5, 2.8 Hz, 2F), -146.63 – -147.80 (m, 1F), -158.48 (ddtd, *J* = 137.6, 17.3, 8.6, 4.1 Hz, 1F) ppm; **(*Z*)-isomer:** δ = -83.51 (dt, *J* = 6.3, 2.7 Hz, 3F), -111.43 (d, *J* = 12.8 Hz, 1F), -119.73 (dt, *J* = 16.4, 2.5 Hz, 2F), -139.32 (dddd, *J* = 23.1, 13.1, 9.5, 6.9 Hz, 1F) ppm.
^{13}C NMR (100 MHz, CDCl$_3$) of (*E*)-isomer: δ = 164.4 (d, *J* = 2.5 Hz), 146.8–142.8 (m, 1C), 142.1 (d, *J* = 3.0 Hz), 134.4, 133.9–133.0 (m, 1C), 132.5, 128.54, 128.51, 118.5, 113.1, 14.2 ppm; carbons corresponding to the C_2F_5 group cannot be identified due to C-F coupling.
HRMS (m/z): calcd for $C_{15}H_8F_7N_2O$ [M+H]$^+$ 365.0519, found: 365.0527.

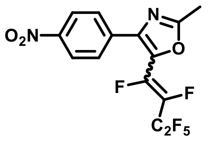

2-Methyl-4-(4-nitrophenyl)-5-(perfluorobut-1-en-1-yl)oxazole (3na):
Yield = 69% (80 mg, *Z*/*E* = 1/2.8). White solid.
IR (KBr): ν = 2931, 1727, 1516, 1222, 981 cm^{-1}.
^{1}H NMR (400 MHz, CDCl$_3$): δ = 8.35–8.25 (m, 2H), 7.91–7.77 (m, 2H), 2.64–2.56 (m, 3H) ppm.
^{19}F NMR (376 MHz, CDCl$_3$) of (*E*)-isomer: δ = -84.08 – -84.20 (m, 3F), -120.14 – -120.30 (m, 2F), -146.88 – -147.65 (m, 1F), -158.20 (dddt, *J* = 137.8, 13.1, 8.8, 4.4 Hz, 1F) ppm; **(*Z*)-isomer:** δ = -83.45 – -83.53 (m, 3F), -111.57 (d, *J* = 12.6 Hz, 1F), -119.73 (d, *J* = 16.8 Hz, 2F), -138.94 – -139.15 (m, 1F) ppm.
^{13}C NMR (100 MHz, CDCl$_3$) of (*E*)-isomer: δ = 164.5 (d, *J* = 2.6 Hz), 148.2, 144.0–138.7 (m, 1C), 136.2, 135.6–135.5 (m, 1C), 128.9, 128.8, 124.3, 124.0, 14.2 ppm; carbons corresponding to the C_2F_5 group cannot be identified due to C-F coupling.
HRMS (m/z): calcd for $C_{14}H_8F_7N_2O_3$ [M+H]$^+$ 385.0418, found: 385.0421.

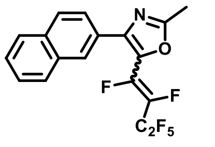

2-Methyl-4-(naphthalen-2-yl)-5-(perfluorobut-1-en-1-yl)oxazole (3oa):
Yield = 89% (104 mg, *Z*/*E* = 1/4.2). Light yellow oil.
IR (KBr): ν = 2931, 1614, 1220, 1032, 981 cm^{-1}.
^{1}H NMR (400 MHz, CDCl$_3$): δ = 8.30–8.15 (m, 1H), 7.95–7.82 (m, 3H), 7.77–7.68 (m, 1H), 7.57–7.48 (m, 2H), 2.66–2.57 (m, 3H) ppm.
^{19}F NMR (376 MHz, CDCl$_3$) of (*E*)-isomer: δ = -84.02 – -84.13 (m, 3F), -120.07 (ddq, *J* = 25.5, 13.3, 2.9 Hz, 2F), -145.44 (dddt, *J* = 138.0, 25.3, 19.5, 5.4 Hz, 1F), -159.18 (ddtd, *J* = 137.6, 16.9, 8.9, 3.9 Hz, 1F) ppm; **(*Z*)-isomer:** δ = -83.44 (dt, *J* = 6.1, 2.6 Hz, 3F), -109.87 (d, *J* = 14.4 Hz, 1F), -119.63 (dd, *J* = 16.4, 2.6 Hz, 2F), -140.72 (tq, *J* = 15.9, 7.9 Hz, 1F) ppm.
^{13}C NMR (100 MHz, CDCl$_3$) of (*E*)-isomer: δ = 164.1 (d, *J* = 2.3 Hz), 147.7–144.4 (m, 1C), 144.2 (d, *J* = 2.5 Hz), 133.7, 133.2, 128.7, 128.6, 127.9, 127.8, 127.7, 127.2, 126.8, 124.8, 124.7, 124.0–123.9 (m, 1C), 14.3 ppm; carbons corresponding to the C_2F_5 group cannot be identified due to C-F coupling.
HRMS (m/z): calcd for $C_{18}H_{11}F_7NO$ [M+H]$^+$ 390.0723, found: 390.0727.

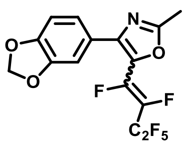

4-(Benzo[*d*][1,3]dioxol-5-yl)-2-methyl-5-(perfluorobut-1-en-1-yl)oxazole (3pa):

Yield = 66% (76 mg, *Z*/*E* = 1/2.4). Light yellow oil.

IR (KBr): ν = 2915, 1719, 1580, 1039, 931 cm^{-1}.

^{1}H NMR (400 MHz, CDCl$_3$): δ = 7.23–7.10 (m, 2H), 6.88–6.82 (m, 1H), 6.03–5.99 (m, 2H), 2.57–2.51 (m, 3H) ppm.

^{19}F NMR (376 MHz, CDCl$_3$) of (*E*)-isomer: δ = -84.25 (t, *J* = 4.5 Hz, 3F), -120.01 – -120.21 (m, 2F), -145.52 – -147.13 (m, 1F), -160.07 (dddt, *J* = 137.7, 13.1, 8.7, 4.4 Hz, 1F) ppm; **(*Z*)-isomer:** δ = -83.51 – -83.60 (m, 3F), -110.00 (dd, *J* = 14.7, 2.7 Hz, 1F), -119.43 – -119.87 (m, 2F), -141.04 (ddt, *J* = 23.7, 16.1, 7.7 Hz, 1F) ppm.

^{13}C NMR (100 MHz, CDCl$_3$) of (*E*)-isomer: δ = 163.8 (d, *J* = 2.6 Hz), 148.8, 148.1, 147.5–144.1 (m, 1C), 144.0 (d, *J* = 3.0 Hz), 131.6–130.8 (m, 1C), 123.9, 122.3 (d, *J* = 3.0 Hz), 108.8, 108.6, 108.1 (d, *J* = 3.0 Hz), 101.6, 14.1 ppm; carbons corresponding to the C_2F_5 group cannot be identified due to C-F coupling.

HRMS (m/z): calcd for $C_{15}H_9F_7NO_3$ [M+H]$^+$ 384.0465, found: 384.0475.

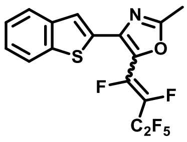

4-(Benzo[*b*]thiophen-2-yl)-2-methyl-5-(perfluorobut-1-en-1-yl)oxazole (3qa):

Yield = 89% (106 mg, *Z*/*E* = 1/3). White solid.

IR (KBr): ν = 3010, 1708, 1577, 1441, 1123, 897 cm^{-1}.

^{1}H NMR (400 MHz, CDCl$_3$): δ = 7.90–7.77 (m, 2H), 7.72–7.63 (m, 1H), 7.42–7.34 (m, 2H), 2.61–2.56 (m, 3H) ppm.

^{19}F NMR (376 MHz, CDCl$_3$) of (*E*)-isomer: δ = -84.06 (ddd, *J* = 7.9, 5.2, 3.4 Hz, 3F), -119.90 (ddq, *J* = 25.9, 13.2, 3.1 Hz, 2F), -146.59 – -147.50 (m, 1F), -159.18 (dddt, *J* = 137.0, 13.2, 8.8, 4.5 Hz, 1F) ppm; **(*Z*)-isomer:** δ = -83.42 (dt, *J* = 6.2, 2.7 Hz, 3F), -112.00 – -112.11 (m, 1F), -119.65 (dq, *J* = 16.5, 2.6 Hz, 2F), -139.56 – -139.78 (m, 1F) ppm.

^{13}C NMR (100 MHz, CDCl$_3$) of (*E*)-isomer: δ = 163.9 (d, *J* = 2.6 Hz), 146.4–143.8 (m, 1C), 140.5 (d, *J* = 1.5 Hz), 139.9, 132.0, 131.9–131.2 (m, 1C), 125.6, 124.9, 124.73, 124.66, 124.5, 122.5, 122.4, 14.1 ppm; carbons corresponding to the C_2F_5 group cannot be identified due to C-F coupling.

HRMS (m/z): calcd for $C_{16}H_9F_7NOS$ [M+H]$^+$ 396.0288, found: 396.0290.

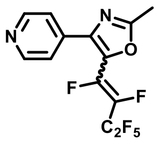

2-Methyl-5-(perfluorobut-1-en-1-yl)-4-(pyridin-4-yl)oxazole (3ra):

Yield = 80% (82 mg, *Z*/*E* = 1/3.1). Light yellow oil.

IR (KBr): ν = 2928, 1705, 1574, 1124, 814, 709 cm^{-1}.

^{1}H NMR (400 MHz, CDCl$_3$): δ = 8.70–8.62 (m, 2H), 7.59–7.47 (m, 2H), 2.59–2.52 (m, 3H) ppm.

^{19}F NMR (376 MHz, CDCl$_3$) of (*E*)-isomer: δ = -84.39 (t, *J* = 4.1 Hz, 3F), -120.45 (ddd, *J* = 25.9, 13.0, 3.5 Hz, 2F), -146.64 – -147.42 (m, 1F), -158.42 (dddt, *J* = 137.8, 17.6, 13.2, 4.7 Hz, 1F) ppm; **(*Z*)-isomer:** δ = -83.73 (d, *J* = 7.0 Hz, 3F), -111.69 (d, *J* = 12.4 Hz, 1F), -119.98 (d, *J* = 16.0 Hz, 2F), -139.32 – -139.73 (m, 1F) ppm.

^{13}C NMR (100 MHz, CDCl$_3$) of (*E*)-isomer: δ = 164.4 (d, *J* = 2.4 Hz), 150.4, 146.8–142.6 (m, 1C), 141.4 (d, *J* = 3.3 Hz), 137.4, 134.4–133.4 (m, 1C), 122.0, 121.9, 14.1 ppm; carbons corresponding to the C_2F_5 group cannot be identified due to C-F coupling.

HRMS (m/z): calcd for $C_{13}H_8F_7N_2O$ [M+H]$^+$ 341.0519, found: 341.0528.

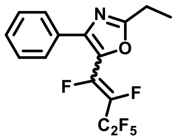

2-Ethyl-5-(perfluorobut-1-en-1-yl)-4-phenyloxazole (3sa):
Yield = 77% (81.7 mg, *Z*/*E* = 1/2.2). Light yellow oil.
IR (KBr): ν = 2949, 1446, 1234, 804 cm^{-1}.
^{1}H NMR (400 MHz, CDCl$_3$): δ = 7.74–7.63 (m, 2H), 7.47–7.37 (m, 3H), 2.96–2.84 (m, 2H), 1.47–1.36 (m, 3H) ppm.
^{19}F NMR (376 MHz, CDCl$_3$) of (*E*)-isomer: δ = -84.22 (dd, *J* = 9.8, 6.0 Hz, 3F), -119.99 – -120.19 (m, 2F), -146.10 – -146.87 (m, 1F), -159.78 – -160.58 (m, 1F) ppm; **(*Z*)-isomer:** δ = -83.58 (d, *J* = 8.5 Hz, 3F), -110.62 (d, *J* = 14.3 Hz, 1F), -119.82 (d, *J* = 16.1 Hz, 2F), -141.37 (ddq, *J* = 23.7, 16.2, 7.5 Hz, 1F) ppm.
^{13}C NMR (100 MHz, CDCl$_3$) of (*E*)-isomer: δ = 168.2 (d, *J* = 2.7 Hz), 147.4–144.5 (m, 1C), 144.1 (d, *J* = 3.2 Hz), 132.4–131.3 (m, 1C), 129.6, 129.0, 128.7, 127.98, 127.95, 22.0, 11.1 ppm; carbons corresponding to the C_2F_5 group cannot be identified due to C-F coupling.
HRMS (m/z): calcd for $C_{15}H_{11}F_7NO$ [M+H]$^+$ 354.0723, found: 354.0729.

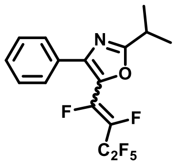

2-Isopropyl-5-(perfluorobut-1-en-1-yl)-4-phenyloxazole (3ta):
Yield = 75% (83.1 mg, *Z*/*E* = 1/2.2). Light blue oil.
IR (KBr): ν = 2981, 1566, 1328, 1220, 1181, 983, 694 cm^{-1}.
^{1}H NMR (400 MHz, CDCl$_3$): δ = 7.76–7.64 (m, 2H), 7.48–7.37 (m, 3H), 3.25–3.13 (m, 1H), 1.46–1.39 (m, 6H) ppm.
^{19}F NMR (376 MHz, CDCl$_3$) of (*E*)-isomer: δ = -84.22 (t, *J* = 5.2 Hz, 3F), -120.06 (dd, *J* = 26.2, 14.2 Hz, 2F), -146.32 – -147.18 (m, 1F), -160.06 – -160.80 (m, 1F) ppm; **(*Z*)-isomer:** δ = -83.61 (d, *J* = 8.1 Hz, 3F), -111.12 (d, *J* = 13.7 Hz, 1F), -119.90 (d, *J* = 16.5 Hz, 2F), -141.48 – -141.83 (m, 1F) ppm.
^{13}C NMR (100 MHz, CDCl$_3$) of (*E*)-isomer: δ = 171.4 (d, *J* = 2.0 Hz), 147.4–144.7 (m, 1C), 144.0 (d, *J* = 3.2 Hz), 132.0–131.1 (m, 1C), 129.5, 129.0, 128.7, 128.04, 128.02, 28.8, 20.3 ppm; carbons corresponding to the C_2F_5 group cannot be identified due to C-F coupling.
HRMS (m/z): calcd for $C_{16}H_{13}F_7NO$ [M+H]$^+$ 368.0880, found: 368.0873.

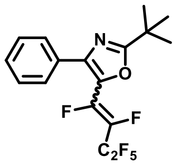

2-(*tert*-Butyl)-5-(perfluorobut-1-en-1-yl)-4-phenyloxazole (3ua):
Yield = 71% (81.6 mg, *Z*/*E* = 1/2.4). Yellow oil.
IR (KBr): ν = 2979, 1561, 1328, 1221, 1180, 694 cm^{-1}.
^{1}H NMR (400 MHz, CDCl$_3$): δ = 7.79–7.66 (m, 2H), 7.49–7.37 (m, 3H), 1.51–1.44 (m, 9H) ppm.
^{19}F NMR (376 MHz, CDCl$_3$) of (*E*)-isomer: δ = -84.05 – -84.34 (m, 3F), -119.99 – -120.16 (m, 2F), -146.68 – -147.33 (m, 1F), -160.53 – -161.06 (m, 1F) ppm; **(*Z*)-isomer:** δ = -83.67 (d, *J* = 6.8 Hz, 3F), -111.66 (d, *J* = 14.2 Hz, 1F), -119.91 – -119.98 (m, 2F), -141.88 – -142.27 (m, 1F) ppm.
^{13}C NMR (100 MHz, CDCl$_3$) of (*E*)-isomer: δ = 173.7 (d, *J* = 2.7 Hz), 147.5–144.6 (m, 1C), 144.0 (d, *J* = 3.4 Hz), 131.9–131.3 (m, 1C), 129.5, 129.0, 128.7, 128.14, 128.11, 34.3, 28.5 ppm; carbons corresponding to the C_2F_5 group cannot be identified due to C-F coupling.
HRMS (m/z): calcd for $C_{17}H_{15}F_7NO$ [M+H]$^+$ 382.1036, found: 382.1031.

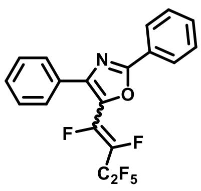

5-(Perfluorobut-1-en-1-yl)-2,4-diphenyloxazole (3va):
Yield = 89% (107 mg, *Z*/*E* = 1/2.9). White solid.
IR (KBr): ν = 2929, 1577, 1442, 1057, 834 cm^{-1}.
^{1}H NMR (400 MHz, CDCl$_3$): δ = 8.20–8.12 (m, 2H), 7.86–7.72 (m, 2H), 7.59–7.44 (m, 6H) ppm.
^{19}F NMR (376 MHz, CDCl$_3$) of (*E*)-isomer: δ = -84.05 – -84.14 (m, 3F), -119.84 – -119.99 (m, 2F), -147.35 – -148.14 (m, 1F), -160.01 (dddt, *J* = 137.1, 13.3, 8.9, 4.5 Hz, 1F) ppm; **(*Z*)-isomer:** δ = -83.41 – -83.46 (m, 3F), -111.71 (d, *J* = 14.2 Hz, 1F), -119.57 (dd, *J* = 16.4, 2.4 Hz, 2F), -140.98 – -141.19 (m, 1F) ppm.
^{13}C NMR (100 MHz, CDCl$_3$) of (*E*)-isomer: δ = 163.4 (d, *J* = 3.0 Hz), 145.7–145.2 (m, 1C), 131.9, 130.1, 129.8, 129.1, 129.0, 128.8, 128.23, 128.20, 127.3, 127.2, 126.2 ppm; carbons corresponding to the C_2F_5 group cannot be identified due to C-F coupling.
HRMS (m/z): calcd for $C_{19}H_{11}F_7NO$ [M+H]$^+$ 402.0723, found: 402.0729.

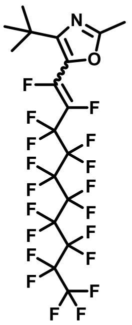

4-(*tert*-Butyl)-2-methyl-5-(perfluorodec-1-en-1-yl)oxazole (3we):
Yield = 42% (77.9 mg, *Z*/*E* = 1/3.2). Yellow oil.
IR (KBr): ν = 2976, 1583, 1368, 1246, 1152, 669 cm^{-1}.
^{1}H NMR (400 MHz, CDCl$_3$): δ = 2.48–2.41 (m, 3H), 1.31–1.26 (m, 9H) ppm.
^{19}F NMR (376 MHz, CDCl$_3$): δ = -80.91 (s, 0.9F), -80.92 – -80.98 (m, 3F), -99.32 (d, *J* = 13.4 Hz, 0.3F), -116.07 (s, 0.6F), -117.14 – -117.44 (m, 2F), -121.79 – -121.97 (m, 2.4F), -121.99 – -122.20 (m, 6F), -122.77 (s, 0.6F), -122.81 – -122.98 (m, 2F), -123.35 – -123.57 (m, 2F), -126.17 – -126.25 (m, 0.6F), -126.26 – -126.36 (m, 2F), -127.21 – -127.86 (m, 1F), -140.30 – -140.59 (m, 0.3F), -155.13 – -155.65 (m, 1F) ppm.
^{13}C NMR (100 MHz, CDCl$_3$) of (*E*)-isomer: δ = 162.8, 154.2, 147.6–144.1 (m, 1C), 130.7–125.3 (m, 1C), 119.6–115.0 (m, 1C), 32.4, 29.1 (d, *J* = 2.9 Hz), 14.1 ppm; carbons corresponding to the C_8F_{17} group cannot be identified due to C-F coupling.
HRMS (m/z): calcd for $C_{18}H_{13}F_{19}NO$ [M+H]$^+$ 620.0688, found: 620.0690.

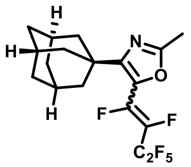

4-((3*r*,5*r*,7*r*)-Adamantan-1-yl)-2-methyl-5-(perfluorobut-1-en-1-yl)oxazole (3xa):
Yield = 54% (64 mg, *Z*/*E* = 1/3). Light yellow oil.
IR (KBr): ν = 2909, 1726, 1223, 799, 745 cm^{-1}.
^{1}H NMR (400 MHz, CDCl$_3$): δ = 2.55–2.41 (m, 3H), 2.04 (s, 3H), 1.92 (s, 6H), 1.75 (s, 6H) ppm.
^{19}F NMR (376 MHz, CDCl$_3$) of (*E*)-isomer: δ = -84.19 (tt, *J* = 4.8, 3.1 Hz, 3F), -120.48 (ddq, *J* = 25.0, 13.4, 3.1 Hz, 2F), -125.29 – -126.22 (m, 1F), -155.60 (dddt, *J* = 146.8, 13.2, 8.8, 4.4 Hz, 1F) ppm; **(*Z*)-isomer:** δ = -83.16 (dt, *J* = 7.7, 2.7 Hz, 3F), -98.11 – -98.22 (m, 1F), -119.39 (dq, *J* = 16.8, 2.6 Hz, 2F), -141.13 (ddt, *J* = 24.1, 16.1, 8.1 Hz, 1F) ppm.
^{13}C NMR (100 MHz, CDCl$_3$) of (*E*)-isomer: δ = 163.2–162.8 (m, 1C), 154.2–153.9 (m, 1C), 147.1–146.6 (m, 1C), 144.6–143.9 (m, 1C), 130.3–129.3 (m, 1C), 40.84, 40.81, 36.6, 34.5, 28.3, 14.2 ppm; carbons corresponding to the C_2F_5 group cannot be identified due to C-F coupling.
HRMS (m/z): calcd for $C_{18}H_{19}F_7NO$ [M+H]$^+$ 398.1349, found: 398.1355.

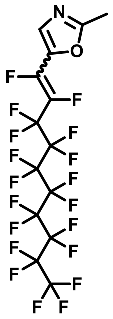

2-Methyl-5-(perfluorodec-1-en-1-yl)oxazole (3ye):
Yield = 12% (20.3 mg, *Z*/*E* = 1/2.8). Yellow oil.
IR (KBr): ν = 1703, 1557, 1213, 1149, 953, 667 cm^{-1}.
^{1}H NMR (400 MHz, CDCl$_3$): δ = 7.43–7.30 (m, 1H), 2.57–2.51 (m, 3H) ppm.
^{19}F NMR (376 MHz, CDCl$_3$): δ = -80.95 (s, 1.2F), -80.97 – -81.03 (m, 3F), -114.84 (dt, *J* = 17.9, 9.1 Hz, 0.8F), -117.04 (dt, *J* = 26.8, 13.2 Hz, 2F), -121.04 (d, *J* = 8.0 Hz, 0.4F), -121.84 – -122.01 (m, 2.4F), -122.04 – -122.32 (m, 6F), -122.54 (td, *J* = 12.6, 6.6 Hz, 0.8F), -122.85 (s, 0.8F), -122.87 – -123.02 (m, 2F), -123.69 (dq, *J* = 14.2, 7.3 Hz, 2F), -126.30 (s, 0.8F), -126.33 – -126.48 (m, 2F), -145.74 – -146.03 (m, 0.4F), -156.29 – -156.93 (m, 1F), -163.85 (dt, *J* = 132.7, 13.6 Hz, 1F) ppm.
^{13}C NMR (100 MHz, CDCl$_3$) of (*E*)-isomer: δ = 164.4, 147.3–143.8 (m, 1C), 139.6–137.6 (m, 1C), 131.1, 131.0, 14.1 ppm; carbons corresponding to the C_8F_{17} group cannot be identified due to C-F coupling.
HRMS (m/z): calcd for $C_{14}H_5F_{19}NO$ [M+H]$^+$ 564.0062, found: 564.0067.

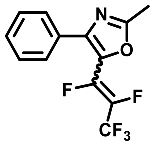

2-Methyl-5-(perfluoroprop-1-en-1-yl)-4-phenyloxazole (3ab):
Yield = 68% (59 mg, *Z*/*E* = 1/1.6). Light yellow oil.
IR (KBr): ν = 2930, 1718, 1151, 903, 694 cm^{-1}.
^{1}H NMR (400 MHz, CDCl$_3$): δ = 7.73–7.63 (m, 2H), 7.47–7.37 (m, 3H), 2.62–2.53 (m, 3H) ppm.
^{19}F NMR (376 MHz, CDCl$_3$) of (*E*)-isomer: δ = -67.31 (dd, *J* = 21.3, 11.5 Hz, 3F), -147.90 (dq, *J* = 137.1, 21.4 Hz, 1F), -162.07 (dq, *J* = 137.0, 11.5 Hz, 1F) ppm; **(*Z*)-isomer:** δ = -68.25 (dd, *J* = 13.0, 7.3 Hz, 3F), -116.88 (dt, *J* = 14.3, 7.2 Hz, 1F), -143.62 – -143.83 (m, 1F) ppm.
^{13}C NMR (100 MHz, CDCl$_3$) of (*E*)-isomer: δ = 163.9 (d, *J* = 2.6 Hz), 145.2–143.9 (m, 1C), 133.0–131.0 (m, 1C), 129.6, 129.0, 128.8, 127.94, 127.91, 127.1, 121.2–118.4 (m, 1C), 14.2 ppm.
HRMS (m/z): calcd for $C_{13}H_9F_5NO$ [M+H]$^+$ 290.0599, found: 290.0608.

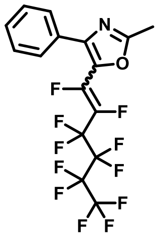

2-Methyl-5-(perfluorohex-1-en-1-yl)-4-phenyloxazole (3ac):
Yield = 88% (116 mg, *Z*/*E* = 1/9.1). Light yellow oil.
IR (KBr): ν = 2931, 1701, 1239, 882, 738 cm^{-1}.
^{1}H NMR (400 MHz, CDCl$_3$): δ = 7.73–7.60 (m, 2H), 7.48–7.36 (m, 3H), 2.65–2.52 (m, 3H) ppm.
^{19}F NMR (376 MHz, CDCl$_3$) of (*E*)-isomer: δ = -80.93 (t, *J* = 9.7 Hz, 3F), -117.01 (dq, *J* = 25.6, 12.7 Hz, 2F), -124.14 – -124.35 (m, 2F), -126.20 – -126.35 (m, 2F), -145.73 – -146.55 (m, 1F), -158.34 – -159.26 (m, 1F) ppm; **(*Z*)-isomer:** δ = -80.90 (s, 3F), -109.55 (d, *J* = 13.3 Hz, 1F), -116.38 (q, *J* = 14.2 Hz, 2F), -123.10 – -123.29 (m, 2F), -126.39 (dt, *J* = 12.3, 5.1 Hz, 2F), -139.75 – -140.34 (m, 1F) ppm.
^{13}C NMR (100 MHz, CDCl$_3$) of (*E*)-isomer: δ = 164.0 (d, *J* = 2.5 Hz), 147.3–144.6 (m, 1C), 144.3 (d, *J* = 3.2 Hz), 132.4–131.5 (m, 1C), 129.6, 129.0, 128.7, 128.0, 127.9, 14.2 ppm; carbons corresponding to the C_4F_9 group cannot be identified due to C-F coupling.
HRMS (m/z): calcd for $C_{16}H_9F_{11}NO$ [M+H]$^+$ 440.0503, found: 440.0508.

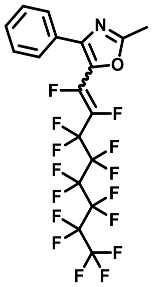

2-Methyl-5-(perfluorooct-1-en-1-yl)-4-phenyloxazole (3ad):

Yield = 80% (130 mg, *Z*/*E* = 1/2.1). Light yellow oil.

IR (KBr): ν = 2914, 1574, 1213, 1151, 901 cm^{-1}.

^{1}H NMR (400 MHz, $CDCl_3$): δ = 7.75–7.60 (m, 2H), 7.49–7.36 (m, 3H), 2.62–2.52 (m, 3H) ppm.

^{19}F NMR (376 MHz, $CDCl_3$) of (*E*)-isomer: δ = -80.69 – -80.82 (m, 3F), -116.81 (dq, *J* = 25.9, 13.7 Hz, 2F), -121.97 – -122.18 (m, 2F), -122.80 (s, 2F), -123.15 – -123.53 (m, 2F), -126.14 – -126.29 (m, 2F), -145.65 – -146.39 (m, 1F), -158.37 – -159.10 (m, 1F) ppm; **(*Z*)-isomer:** δ = -80.82 – -80.91 (m, 3F), -109.45 (d, *J* = 13.3 Hz, 1F), -116.16 (q, *J* = 14.4 Hz, 2F), -122.22 – -122.45 (m, 4F), -122.65 – -122.79 (m, 2F), -126.06 – -126.14 (m, 2F), -139.72 – -140.03 (m, 1F) ppm.

^{13}C NMR (100 MHz, $CDCl_3$) of (*E*)-isomer: δ = 164.0 (d, *J* = 2.5 Hz), 147.7–144.6 (m, 1C), 144.3 (d, *J* = 2.8 Hz), 132.4–131.7 (m, 1C), 129.7, 129.0, 128.7, 127.98, 127.95, 14.2 ppm; carbons corresponding to the C_6F_{13} group cannot be identified due to C-F coupling.

HRMS (m/z): calcd for $C_{18}H_9F_{15}NO$ $[M+H]^+$ 540.0439, found: 540.0444.

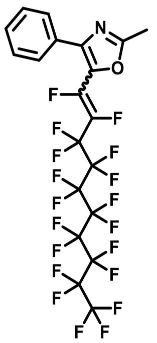

2-Methyl-5-(perfluorodec-1-en-1-yl)-4-phenyloxazole (3ae):

Yield = 47% (90.9 mg, *Z*/*E* = 1/3.1). White solid.

IR (KBr): ν = 2967, 1705, 1242, 775 cm^{-1}.

^{1}H NMR (400 MHz, $CDCl_3$): δ = 7.78–7.59 (m, 2H), 7.51–7.33 (m, 3H), 2.65–2.48 (m, 3H) ppm.

^{19}F NMR (376 MHz, $CDCl_3$) of (*E*)-isomer: δ = -80.93 (d, *J* = 10.6 Hz, 3F), -116.89 (d, *J* = 25.7 Hz, 2F), -121.97 (s, 6F), -122.80 (s, 2F), -123.38 (s, 2F), -126.24 (s, 2F), -146.25 (dt, *J* = 137.3, 26.8 Hz, 1F), -158.76 (dd, *J* = 137.3, 14.2 Hz, 1F) ppm; **(*Z*)-isomer:** δ = -80.73 – -80.87 (m, 3F), -109.33 – -109.71 (m, 1F), -116.23 (s, 2F), -121.72 – -121.91 (m, 6F), -122.36 (s, 2F), -122.63 – -122.75 (m, 2F), -126.07 – -126.20 (m, 2F), -139.98 (s, 1F) ppm.

^{13}C NMR (100 MHz, $CDCl_3$) of (*E*)-isomer: δ = 164.0, 147.7–144.5 (m, 1C), 144.3, 132.4–130.2 (m, 1C), 129.6 (2C), 128.7, 128.0 (2C), 14.1 ppm; carbons corresponding to the C_8F_{17} group cannot be identified due to C-F coupling.

HRMS (m/z): calcd for $C_{20}H_9F_{19}NO$ $[M+H]^+$ 640.0375, found: 640.0381.

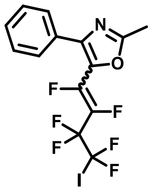

5-(1,2,3,3,4,4-Hexafluoro-4-iodobut-1-en-1-yl)-2-methyl-4-phenyloxazole (3af):

Yield = 83% (110.9 mg, *Z*/*E* = 1/2.9). White solid.

IR (KBr): ν = 1688, 1572, 1309, 1189, 1067, 774 cm^{-1}.

^{1}H NMR (400 MHz, $CDCl_3$): δ = 7.74–7.63 (m, 2H), 7.47–7.37 (m, 3H), 2.59–2.54 (m, 3H) ppm.

^{19}F NMR (376 MHz, $CDCl_3$) of (*E*)-isomer: δ = -60.98 – -61.17 (m, 2F), -110.38 – -110.69 (m, 2F), -145.37 (dtt, *J* = 137.3, 25.4, 6.1 Hz, 1F), -155.92 – -157.26 (m, 1F) ppm; **(*Z*)-isomer:** δ = -59.98 (q, *J* = 8.7 Hz, 2F), -109.37 (d, *J* = 13.6 Hz, 1F), -109.54 (dt, *J* = 16.1, 8.5 Hz, 2F), -137.76 – -138.15 (m, 1F) ppm.

^{13}C NMR (100 MHz, $CDCl_3$) of (*E*)-isomer: δ = 163.8 (d, *J* = 2.5 Hz), 147.1–144.2 (m, 1C),

143.9 (d, J = 3.2 Hz), 132.5–131.8 (m, 1C), 129.5, 129.0, 128.7, 127.91, 127.89, 14.2 ppm; carbons corresponding to the C_2F_4I group cannot be identified due to C-F coupling.
HRMS (m/z): calcd for $C_{14}H_9F_6INO$ $[M+H]^+$ 447.9628, found: 447.9623.

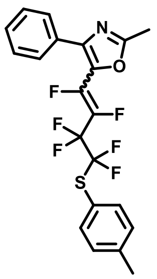

5-(1,2,3,3,4,4-Hexafluoro-4-(*p*-tolylthio)but-1-en-1-yl)-2-methyl-4-phenyloxazole (3ag):
Yield = 89% (118.8 mg, *Z*/*E* = 1/2.5). White solid.
IR (KBr): ν = 1689, 1310, 1187, 1076, 777, 507 cm^{-1}.
^{1}H NMR (400 MHz, CDCl$_3$): δ = 7.79–7.68 (m, 2H), 7.60–7.55 (m, 1H), 7.47–7.36 (m, 4H), 7.25–7.15 (m, 2H), 2.59–2.53 (m, 3H), 2.40–2.36 (m, 3H) ppm.

^{19}F NMR (376 MHz, CDCl$_3$) of (*E*)-isomer: δ = -88.87 (q, J = 6.4 Hz, 2F), -114.37 – -114.66 (m, 2F), -146.02 (dtt, J = 137.9, 25.6, 6.2 Hz, 1F), -155.41 – -155.93 (m, 1F) ppm; **(*Z*)-isomer:** δ = -87.83 (q, J = 7.7 Hz, 2F), -111.79 (d, J = 14.6 Hz, 1F), -113.82 (dt, J = 16.2, 7.5 Hz, 2F), -137.35 (dtd, J = 25.2, 16.4, 8.9 Hz, 1F) ppm.
^{13}C NMR (100 MHz, CDCl$_3$) of (*E*)-isomer: δ = 163.6 (d, J = 2.3 Hz), 146.6–143.7 (m, 1C), 143.5 (d, J = 3.2 Hz), 141.4, 137.3, 132.7–132.1 (m, 1C), 130.3, 130.2, 130.1, 129.4, 128.9, 128.7, 127.84, 127.82, 119.7 (t, J = 2.7 Hz), 21.4, 14.1 ppm.
HRMS (m/z): calcd for $C_{21}H_{16}F_6NOS$ $[M+H]^+$ 444.0851, found: 444.0848.

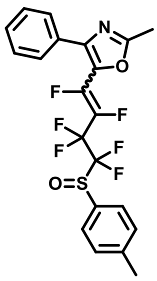

5-(1,2,3,3,4,4-Hexafluoro-4-(*p*-tolylsulfinyl)but-1-en-1-yl)-2-methyl-4-phenyloxazole (3ah):
Yield = 85% (117.8 mg, *Z*/*E* = 1/2.8). Yellow oil.
IR (KBr): ν = 1709, 1575, 1235, 1181, 918, 781 cm^{-1}.
^{1}H NMR (400 MHz, CDCl$_3$): δ = 7.74–7.55 (m, 4H), 7.46–7.32 (m, 5H), 2.57–2.52 (m, 3H), 2.46–2.41 (m, 3H) ppm.

^{19}F NMR (376 MHz, CDCl$_3$) of (*E*)-isomer: δ = -112.41 – -113.50 (m, 1F), -114.83 – -116.16 (m, 2F), -124.07 (q, J = 6.0 Hz, 0.5F), -124.70 (q, J = 6.9 Hz, 0.5F), -146.46 – -147.06 (m, 1F), -158.04 – -158.60 (m, 1F) ppm; **(*Z*)-isomer:** δ = -110.88 – -111.89 (m, 2F), -113.60 – -114.11 (m, 1F), -114.43 (ddd, J = 186.3, 15.8, 8.2 Hz, 1F), -123.60 (t, J = 7.7 Hz, 0.5F), -124.17 – -124.34 (m, 0.5F), -139.33 (ddt, J = 25.7, 17.4, 8.3 Hz, 1F) ppm.
^{13}C NMR (100 MHz, CDCl$_3$) of (*E*)-isomer: δ = 163.7 (d, J = 2.8 Hz), 147.0–146.0 (m, 1C), 144.7, 143.8 (d, J = 3.3 Hz), 132.5–132.1 (m, 1C), 132.0, 130.21, 130.15, 130.0, 129.4, 128.9, 128.6, 127.93, 127.90, 126.8, 21.7, 14.1 ppm.
HRMS (m/z): calcd for $C_{21}H_{16}F_6NO_2S$ $[M+H]^+$ 460.0800, found: 460.0803.

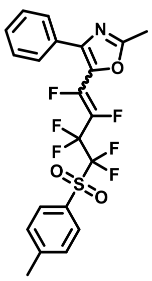

5-(1,2,3,3,4,4-Hexafluoro-4-tosylbut-1-en-1-yl)-2-methyl-4-phenyloxazole (3ai):

Yield = 94% (133.9 mg, *Z*/*E* = 1/3). Yellow oil.

IR (KBr): ν = 3068, 1595, 1364, 1190, 1119, 585 cm^{-1}.

^{1}H NMR (400 MHz, $CDCl_3$): δ = 7.94–7.76 (m, 2H), 7.75–7.65 (m, 2H), 7.46–7.35 (m, 5H), 2.58–2.51 (m, 3H), 2.49–2.44 (m, 3H) ppm.

^{19}F NMR (376 MHz, $CDCl_3$) of (*E*)-isomer: δ = -113.67 (q, *J* = 5.6 Hz, 2F), -113.96 – -114.14 (m, 2F), -146.34 (dtt, *J* = 137.0, 26.7, 7.0 Hz, 1F), -158.17 (dtd, *J* = 138.0, 13.9, 6.8 Hz, 1F) ppm; **(*Z*)-isomer:** δ = -110.90 (d, *J* = 13.7 Hz, 1F), -112.39 (d, *J* = 10.6 Hz, 2F), -113.42 (d, *J* = 17.7 Hz, 2F), -138.98 – -139.21 (m, 1F) ppm.

^{13}C NMR (100 MHz, $CDCl_3$) of (*E*)-isomer: δ = 163.8 (d, *J* = 2.5 Hz), 148.3, 147.0–144.2 (m, 1C), 143.9 (d, *J* = 3.1 Hz), 132.4–131.9 (m, 1C), 131.1, 131.0, 130.42, 130.35, 130.0, 129.5, 128.9, 128.6, 128.0, 127.9, 22.0, 14.1 ppm.

HRMS (m/z): calcd for $C_{21}H_{16}F_6NO_3S$ $[M+H]^+$ 476.0750, found: 476.0754.

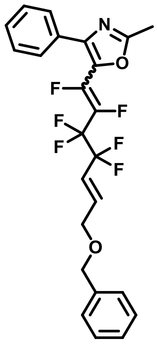

5-((5*E*)-7-(Benzyloxy)-1,2,3,3,4,4-hexafluorohepta-1,5-dien-1-yl)-2-methyl-4-phenyloxazole (3aj):

Yield = 23% (31.7 mg, *Z*/*E* = 1/2.4). Yellow oil.

IR (KBr): ν = 2858, 1574, 1232, 964, 696 cm^{-1}.

^{1}H NMR (400 MHz, $CDCl_3$): δ = 7.76–7.65 (m, 2H), 7.47–7.29 (m, 8H), 6.52–6.28 (m, 1H), 6.11–5.79 (m, 1H), 4.59–4.51 (m, 2H), 4.20–4.04 (m, 2H), 2.60–2.53 (m, 3H) ppm.

^{19}F NMR (376 MHz, $CDCl_3$) of (*E*)-isomer: δ = -112.78 – -112.96 (m, 2F), -117.78 (dd, *J* = 24.9, 12.5 Hz, 2F), -146.78 – -147.41 (m, 1F), -155.23 – -155.85 (m, 1F) ppm; **(*Z*)-isomer:** δ = -112.31 (d, *J* = 11.0 Hz, 2F), -112.64 (d, *J* = 14.8 Hz, 1F), -117.13 (d, *J* = 15.6 Hz, 2F), -136.75 – -137.07 (m, 1F) ppm.

^{13}C NMR (100 MHz, $CDCl_3$) of (*E*)-isomer: δ = 163.5 (d, *J* = 1.0 Hz), 146.1–143.5 (m, 1C), 143.2 (d, *J* = 3.0 Hz), 138.5, 138.4, 137.6, 132.6–132.2 (m, 1C), 129.4, 128.9, 128.7, 128.6, 128.0, 127.81, 127.77, 117.8, 117.6, 117.3, 72.9, 68.3, 14.2 ppm.

HRMS (m/z): calcd for $C_{24}H_{20}F_6NO_2$ $[M+H]^+$ 468.1393, found: 468.1391.

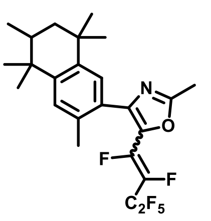

4-(3,5,5,6,8,8-Hexamethyl-5,6,7,8-tetrahydronaphthalen-2-yl)-2-methyl-5-(perfluorobut-1-en-1-yl)oxazole (3za):

Yield = 64% (91.3 mg, *Z*/*E* = 1/2.7). Yellow oil.

IR (KBr): ν = 2967, 1576, 1322, 1220, 1181, 863 cm^{-1}.

^{1}H NMR (400 MHz, $CDCl_3$): δ = 7.24–7.21 (m, 1H), 7.19–7.16 (m, 1H), 2.61–2.53 (m, 3H), 2.33–2.27 (m, 3H), 1.94–1.79 (m, 1H), 1.69–1.59 (m, 1H), 1.40–1.35 (m, 1H), 1.35–1.25 (m, 6H), 1.24–1.05 (m, 6H), 1.03–0.97 (m, 3H) ppm.

^{19}F NMR (376 MHz, $CDCl_3$) of (*E*)-isomer: δ = -84.43 (d, *J* = 3.6 Hz, 3F), -119.83 (dd, *J* = 25.9, 13.4 Hz, 2F), -150.10 – -150.76 (m, 1F), -163.53 – -164.10 (m, 1F) ppm; **(*Z*)-isomer:** δ = -83.50 (d, *J* = 7.6 Hz, 3F), -110.75 (d, *J* = 13.3 Hz, 1F), -119.53 (d, *J* = 16.5 Hz, 2F), -144.13 – -144.44 (m, 1F) ppm.

^{13}C NMR (100 MHz, $CDCl_3$) of (*E*)-isomer: δ = 163.4, 163.3, 147.5, 145.2 (d, J = 2.0 Hz), 144.9–144.2 (m, 1C), 142.2, 133.7, 133.6–131.9 (m, 1C), 129.1, 128.2, 126.8, 43.7, 37.8, 34.6, 34.1, 32.4, 31.9, 28.6, 25.0, 19.7, 16.9, 14.2 ppm; carbons corresponding to the C_2F_5 group cannot be identified due to C-F coupling.

HRMS (m/z): calcd for $C_{24}H_{27}F_7NO$ $[M+H]^+$ 478.1975, found: 478.1974.

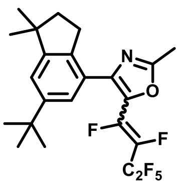

4-(6-(*tert*-Butyl)-1,1-dimethyl-2,3-dihydro-1*H*-inden-4-yl)-2-methyl-5-(perfluorobut-1-en-1-yl)oxazole (3a'a):

Yield = 79% (109.6 mg, *Z*/*E* = 1/3.6). Yellow oil.

IR (KBr): ν = 2958, 1578, 1332, 1221, 1005, 880 cm^{-1}.

^{1}H NMR (400 MHz, $CDCl_3$): δ = 7.27–7.23 (m, 1H), 7.22–7.19 (m, 1H), 2.96–2.83 (m, 2H), 2.59–2.54 (m, 3H), 1.95–1.89 (m, 2H), 1.32 (s, 9H), 1.29–1.25 (m, 6H) ppm.

^{19}F NMR (376 MHz, $CDCl_3$) of (*E*)-isomer: δ = -84.40 (s, 3F), -119.79 (dd, J = 25.5, 13.5 Hz, 2F), -148.58 – -149.27 (m, 1F), -162.37 – -162.99 (m, 1F) ppm; **(*Z*)-isomer:** δ = -83.53 (d, J = 7.6 Hz, 3F), -110.82 (d, J = 13.8 Hz, 1F), -119.59 (d, J = 17.0 Hz, 2F), -143.94 (ddq, J = 22.7, 15.2, 7.7 Hz, 1F) ppm.

^{13}C NMR (100 MHz, $CDCl_3$) of (*E*)-isomer: δ = 163.5 (d, J = 3.1 Hz), 153.3, 149.9, 148.0–146.0 (m, 1C), 145.0 (d, J = 3.8 Hz), 139.2, 133.3–132.7 (m, 1C), 125.7, 124.12, 124.10, 120.2, 44.2, 41.6, 34.8, 31.6, 29.0, 28.6, 14.2 ppm; carbons corresponding to the C_2F_5 group cannot be identified due to C-F coupling.

HRMS (m/z): calcd for $C_{23}H_{25}F_7NO$ $[M+H]^+$ 464.1819, found: 464.1817.

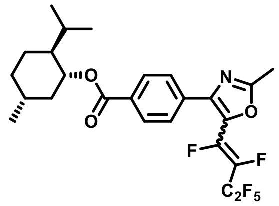

(1*R*,2*S*,5*R*)-2-Isopropyl-5-methylcyclohexyl 4-(2-methyl-5-(perfluorobut-1-en-1-yl)oxazol-4-yl)benzoate (3b'a):

Yield = 98% (153.1 mg, *Z*/*E* = 1/2.9). Yellow oil.

IR (KBr): ν = 2958, 1716, 1276, 1220, 983, 866, 726 cm^{-1}.

^{1}H NMR (400 MHz, $CDCl_3$): δ = 8.13–8.07 (m, 2H), 7.80–7.68 (m, 2H), 4.99–4.90 (m, 1H), 2.61–2.52 (m, 3H), 2.16–2.09 (m, 1H), 2.01–1.90 (m, 1H), 1.77–1.66 (m, 2H), 1.61–1.50 (m, 2H), 1.18–1.05 (m, 2H), 0.96–0.88 (m, 7H), 0.81–0.76 (m, 3H) ppm.

^{19}F NMR (376 MHz, $CDCl_3$) of (*E*)-isomer: δ = -84.19 – -84.31 (m, 3F), -120.15 – -120.36 (m, 2F), -146.78 (dtd, J = 138.5, 26.2, 5.8 Hz, 1F), -159.10 (ddt, J = 137.9, 17.4, 6.7 Hz, 1F) ppm; **(*Z*)-isomer:** δ = -83.60 (d, J = 7.6 Hz, 3F), -110.75 (d, J = 13.4 Hz, 1F), -119.84 (d, J = 16.4 Hz, 2F), -140.13 (dt, J = 14.5, 7.9 Hz, 1F) ppm.

^{13}C NMR (100 MHz, $CDCl_3$) of (*E*)-isomer: δ = 165.6, 164.2 (d, J = 2.4 Hz), 147.0–144.1 (m, 1C), 143.1 (d, J = 3.3 Hz), 134.0, 133.0–132.3 (m, 1C), 131.6, 129.9, 127.82, 127.79, 75.2, 47.3, 41.0, 34.4, 31.5, 26.5, 23.6, 22.1, 20.8, 16.5, 14.1 ppm; carbons corresponding to the C_2F_5 group cannot be identified due to C-F coupling.

HRMS (m/z): calcd for $C_{25}H_{27}F_7NO_3$ $[M+H]^+$ 522.1874, found: 522.1873.

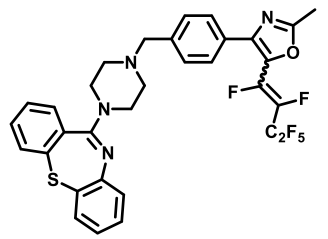

4-(4-((4-(Dibenzo[*b*,*f*][1,4]thiazepin-11-yl)piperazin-1-yl)methyl)phenyl)-2-methyl-5-(perfluorobut-1-en-1-yl)oxazole (3c'a): Yield = 62% (120 mg, *Z*/*E* = 1/3). Light yellow oil.

IR (KBr): ν = 2931, 1705, 1444, 1058, 678 cm^{-1}.

^{1}H NMR (400 MHz, CDCl$_3$): δ = 7.88 (d, *J* = 8.2 Hz, 0.5H), 7.58 (d, *J* = 8.2 Hz, 1.5H), 7.46 (d, *J* = 7.6 Hz, 1H), 7.42–7.34 (m, 3H), 7.29–7.21 (m, 3H), 7.15–7.09 (m, 1H), 7.04 (dd, *J* = 8.0, 1.5 Hz, 1H), 6.87–6.79 (m, 1H), 3.84–3.11 (m, 6H), 2.61–2.38 (m, 7H) ppm.

^{19}F NMR (376 MHz, CDCl$_3$) of (*E*)-isomer: δ = -84.13 (d, *J* = 4.6 Hz, 3F), -119.97 (dd, *J* = 25.4, 13.5 Hz, 2F), -146.26 (dtd, *J* = 138.2, 26.5, 5.8 Hz, 1F), -159.48 – -160.43 (m, 1F) ppm.

^{13}C NMR (100 MHz, CDCl$_3$) of (*E*)-isomer: δ = 197.9, 163.9 (d, *J* = 2.5 Hz), 160.9, 149.0, 147.2–143.2 (m, 1C), 140.0, 136.3, 134.2, 132.3, 132.2, 132.0–131.4 (m, 1C), 130.8, 129.5, 129.2, 129.1, 128.5, 128.3, 128.1, 127.9, 127.8, 125.4, 122.9, 62.7, 53.0, 26.8 (d, *J* = 29.4 Hz), 14.1 ppm; carbons corresponding to the C_2F_5 group cannot be identified due to C-F coupling.

HRMS (m/z): calcd for $C_{32}H_{26}F_7N_4OS$ [M+H]$^+$ 647.1710, found: 647.1715.

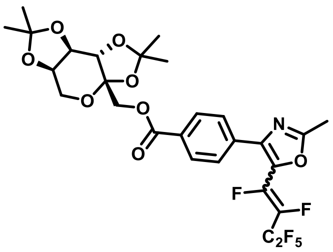

((3a*S*,5a*R*,8a*R*,8b*S*)-2,2,7,7-Tetramethyltetrahydro-3a*H*-bis([1,3]dioxolo)[4,5-*b*:4',5'-*d*]pyran-3a-yl)methyl 4-(2-methyl-5-(perfluorobut-1-en-1-yl)oxazol-4-yl)benzoate (3d'a):

Yield = 86% (161.2 mg, *Z*/*E* = 1/2.8). Yellow oil.

IR (KBr): ν = 2994, 1728, 1585, 1383, 1109, 982, 865, 726 cm^{-1}.

^{1}H NMR (400 MHz, CDCl$_3$): δ = 8.14–8.08 (m, 2H), 7.78–7.65 (m, 2H), 4.71–4.65 (m, 1H), 4.64–4.59 (m, 1H), 4.45–4.43 (m, 1H), 4.34–4.28 (m, 1H), 4.25–4.20 (m, 1H), 3.95–3.89 (m, 1H), 3.80–3.74 (m, 1H), 2.59–2.51 (m, 3H), 1.53–1.40 (m, 6H), 1.35–1.29 (m, 6H) ppm.

^{19}F NMR (376 MHz, CDCl$_3$) of (*E*)-isomer: δ = -84.25 (s, 3F), -120.27 (dd, *J* = 26.0, 12.9 Hz, 2F), -146.07 – -146.77 (m, 1F), -158.53 – -159.08 (m, 1F) ppm; **(*Z*)-isomer:** δ = -83.61 (d, *J* = 6.7 Hz, 3F), -110.76 (d, *J* = 13.2 Hz, 1F), -119.81 (d, *J* = 16.3 Hz, 2F), -140.07 (dq, *J* = 15.9, 7.6 Hz, 1F) ppm.

^{13}C NMR (100 MHz, CDCl$_3$) of (*E*)-isomer: δ = 165.4, 164.2 (d, *J* = 2.5 Hz), 146.8–144.0 (m, 1C), 142.9 (d, *J* = 3.1 Hz), 134.4, 133.2–132.3 (m, 1C), 130.7, 130.1, 127.81, 127.78, 109.2, 108.9, 101.7, 70.8, 70.6, 70.1, 65.5, 61.4, 26.5, 25.9, 25.5, 24.0, 14.0 ppm; carbons corresponding to the C_2F_5 group cannot be identified due to C-F coupling.

HRMS (m/z): calcd for $C_{27}H_{27}F_7NO_8$ [M+H]$^+$ 626.1619, found: 626.1624.

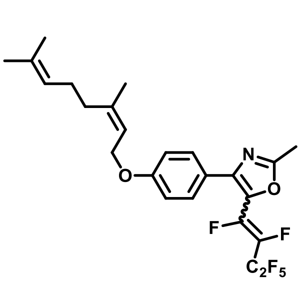

4-(4-(((*Z*)-3,7-Dimethylocta-2,6-dien-1-yl)oxy)phenyl)-2-methyl-5-(perfluorobut-1-en-1-yl)oxazole (3e'a):

Yield = 61% (89.6 mg, *Z*/*E* = 1/2.4). Yellow oil.

IR (KBr): ν = 2931, 1508, 1223, 1178, 980, 836, 744 cm^{-1}.

^{1}H NMR (400 MHz, CDCl$_3$): δ = 7.66–7.54 (m, 2H), 6.99–6.92 (m, 2H), 5.55–5.46 (m, 1H), 5.15–5.06 (m, 1H), 4.60–4.49 (m, 2H), 2.59–2.52 (m, 3H), 2.20–2.06 (m, 4H), 1.83–1.73 (m, 3H), 1.70–1.57 (m, 6H) ppm.

^{19}F NMR (376 MHz, CDCl$_3$) of (*E*)-isomer: δ = -84.05 – -84.37 (m, 3F), -120.04 (dd, J = 26.1, 12.8 Hz, 2F), -146.33 – -147.12 (m, 1F), -160.31 – -160.94 (m, 1F) ppm; **(*Z*)-isomer:** δ = -83.52 (d, J = 7.3 Hz, 3F), -110.07 (d, J = 15.3 Hz, 1F), -119.76 (d, J = 16.1 Hz, 2F), -141.30 – -141.62 (m, 1F) ppm.

^{13}C NMR (100 MHz, CDCl$_3$) of (*E*)-isomer: δ = 163.8 (d, J = 2.6 Hz), 160.0, 147.6–144.3 (m, 1C), 144.1 (d, J = 3.2 Hz), 142.2, 132.4, 131.3–130.5 (m, 1C), 129.30, 129.27, 123.7, 122.3, 120.1, 114.8, 64.7, 32.5, 26.7, 25.8, 23.6, 17.7, 14.1 ppm; carbons corresponding to the C_2F_5 group cannot be identified due to C-F coupling.

HRMS (m/z): calcd for $C_{24}H_{25}F_7NO_2$ $[M+H]^+$ 492.1768, found: 492.1762.

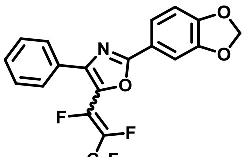

2-(Benzo[*d*][1,3]dioxol-5-yl)-5-(perfluorobut-1-en-1-yl)-4-phenyloxazole (3f'a):

Yield = 78% (104.7 mg, *Z*/*E* = 1/2.4). Yellow solid.

IR (KBr): ν = 2988, 2900, 1406, 1231, 1066 cm^{-1}.

^{1}H NMR (400 MHz, CDCl$_3$): δ = 7.69–7.51 (m, 3H), 7.44–7.39 (m, 1H), 7.35–7.27 (m, 3H), 6.78–6.73 (m, 1H), 5.90–5.86 (m, 2H) ppm.

^{19}F NMR (376 MHz, CDCl$_3$) of (*E*)-isomer: δ = -90.02 – -90.08 (m, 3F), -125.77 (dd, J = 25.5, 13.1 Hz, 2F), -153.88 (dtd, J = 136.9, 25.6, 5.5 Hz, 1F), -166.10 – -166.65 (m, 1F) ppm; **(*Z*)-isomer:** δ = -89.35 (d, J = 7.3 Hz, 3F), -117.64 (d, J = 15.5 Hz, 1F), -125.39 (d, J = 16.7 Hz, 2F), -147.31 (tq, J = 16.3, 8.2 Hz, 1F) ppm.

^{13}C NMR (100 MHz, CDCl$_3$) of (*E*)-isomer: δ = 163.1 (d, J = 2.7 Hz), 150.8, 148.4, 147.4–145.7 (m, 1C), 145.4 (d, J = 3.3 Hz), 132.4–131.0 (m, 1C), 130.2, 129.7, 128.7, 128.20, 128.17, 122.5, 120.2, 108.9, 107.3, 102.0 ppm; carbons corresponding to the C_2F_5 group cannot be identified due to C-F coupling.

HRMS (m/z): calcd for $C_{20}H_{11}F_7NO_3$ $[M+H]^+$ 446.0622, found: 446.0615.

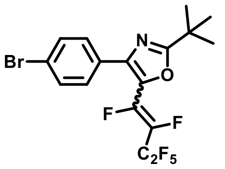

4-(4-Bromophenyl)-2-(*tert*-butyl)-5-(perfluorobut-1-en-1-yl)oxazole (3g'a):

Yield = 79% (108.8 mg, *Z*/*E* = 1/2.3). Yellow oil.

IR (KBr): ν = 2978, 1557, 1326, 1221, 983, 866 cm^{-1}.

^{1}H NMR (400 MHz, CDCl$_3$): δ = 7.64–7.57 (m, 1H), 7.57–7.52 (m, 3H), 1.48–1.42 (m, 9H) ppm.

^{19}F NMR (376 MHz, CDCl$_3$) of (*E*)-isomer: δ = -84.22 (t, J = 4.4 Hz, 3F), -119.97 – -120.11 (m, 2F), -147.45 (dtd, J = 137.0, 25.8, 5.3 Hz, 1F), -159.99 – -160.56 (m, 1F) ppm; **(*Z*)-isomer:** δ = -83.63 (d, J = 7.3 Hz, 3F), -112.31 (d, J = 13.8 Hz, 1F), -119.94 (d, J = 15.6 Hz, 2F), -141.36 (tt, J = 15.0, 7.4 Hz, 1F) ppm.

^{13}C NMR (100 MHz, CDCl$_3$) of (*E*)-isomer: δ = 173.8 (d, J = 2.7 Hz), 147.5–143.2 (m, 1C), 142.8 (d, J = 3.3 Hz), 140.7–134.8 (m, 1C), 131.9, 129.68, 129.65, 129.4, 123.9, 34.3, 28.5 ppm; carbons corresponding to the C_2F_5 group cannot be identified due to C-F coupling.

HRMS (m/z): calcd for $C_{17}H_{14}BrF_7NO$ $[M+H]^+$ 460.0142, found: 460.0139.

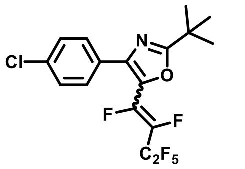

2-(*tert*-Butyl)-4-(4-chlorophenyl)-5-(perfluorobut-1-en-1-yl)oxazole (3h'a):

Yield = 81% (101.2 mg, *Z*/*E* = 1/2.2). Yellow oil.

IR (KBr): ν = 2979, 1559, 1325, 1221, 1094, 836 cm^{-1}.

^{1}H NMR (400 MHz, CDCl$_3$): δ = 7.72–7.57 (m, 2H), 7.44–7.34 (m, 2H), 1.48–1.39 (m, 9H) ppm.

^{19}F NMR (376 MHz, CDCl$_3$) of (*E*)-isomer: δ = -84.27 (d, *J* = 6.4 Hz, 3F), -120.12 (dd, *J* = 26.0, 12.8 Hz, 2F), -147.28 – -147.95 (m, 1F), -160.14 – -160.69 (m, 1F) ppm; **(*Z*)-isomer:** δ = -83.69 (d, *J* = 6.6 Hz, 3F), -112.32 (d, *J* = 14.1 Hz, 1F), -120.03 (d, *J* = 16.6 Hz, 2F), -141.36 – -141.55 (m, 1F) ppm.

^{13}C NMR (100 MHz, CDCl$_3$) of (*E*)-isomer: δ = 173.8 (d, *J* = 2.7 Hz), 147.4–143.1 (m, 1C), 142.8 (d, *J* = 3.4 Hz), 135.5, 132.2–130.2 (m, 1C), 129.5, 129.4, 129.0, 128.9, 34.3, 28.5 ppm; carbons corresponding to the C_2F_5 group cannot be identified due to C-F coupling.

HRMS (m/z): calcd for $C_{17}H_{14}ClF_7NO$ [M+H]$^+$ 416.0647, found: 416.0640.

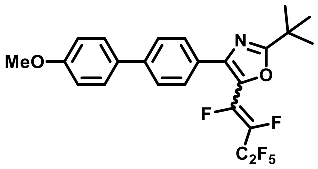

2-(*tert*-Butyl)-4-(4'-methoxy-[1,1'-biphenyl]-4-yl)-5-(perfluorobut-1-en-1-yl)oxazole (5):

Yield = 86% (125.5 mg, *Z*/*E* = 1/4.5). White solid.

IR (KBr): ν = 2983, 1497, 1331, 1217, 825, 725 cm^{-1}.

^{1}H NMR (400 MHz, CDCl$_3$): δ = 7.85–7.70 (m, 2H), 7.69–7.55 (m, 4H), 7.07–6.97 (m, 2H), 3.87 (s, 3H), 1.55–1.45 (m, 9H) ppm.

^{19}F NMR (376 MHz, CDCl$_3$) of (*E*)-isomer: δ = -84.04 – -84.30 (m, 3F), -119.88 – -120.02 (m, 2F), -146.79 – -147.49 (m, 1F), -160.53 – -161.09 (m, 1F) ppm; **(*Z*)-isomer:** δ = -83.53 (d, *J* = 6.9 Hz, 3F), -111.92 (d, *J* = 14.7 Hz, 1F), -119.87 (s, 2F), -142.07 (ddq, *J* = 23.5, 16.2, 7.5 Hz, 1F) ppm.

^{13}C NMR (100 MHz, CDCl$_3$) of (*E*)-isomer: δ = 173.6 (d, *J* = 2.7 Hz), 159.6, 147.7–144.0 (m, 1C), 143.7 (d, *J* = 3.2 Hz), 141.8, 132.9, 131.8–131.2 (m, 1C), 128.6, 128.52, 128.49, 128.3, 126.9, 114.4, 55.4, 34.3, 28.5 ppm; carbons corresponding to the C_2F_5 group cannot be identified due to C-F coupling.

HRMS (m/z): calcd for $C_{24}H_{21}F_7NO_2$ [M+H]$^+$ 488.1455, found: 488.1453.

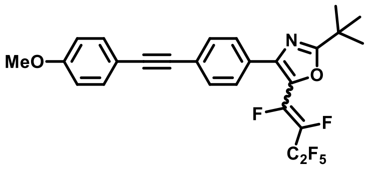

2-(*tert*-Butyl)-4-(4-((4-methoxyphenyl)ethynyl)phenyl)-5-(perfluorobut-1-en-1-yl)oxazole (7):

Yield = 66% (101.1 mg, *Z*/*E* = 1/1.8). Light yellow solid.

IR (KBr): ν = 2982, 2216, 1603, 1516, 1328, 1216, 834, 532 cm^{-1}.

^{1}H NMR (400 MHz, CDCl$_3$): δ = 7.77–7.65 (m, 2H), 7.61–7.56 (m, 2H), 7.53–7.48 (m, 2H), 6.92–6.87 (m, 2H), 3.83 (s, 3H), 1.50–1.43 (m, 9H) ppm.

^{19}F NMR (376 MHz, CDCl$_3$) of (*E*)-isomer: δ = -84.13 (t, *J* = 4.5 Hz, 3F), -119.90 – -120.06 (m, 2F), -146.45 – -147.06 (m, 1F), -160.09 (dddt, *J* = 137.3, 18.1, 13.5, 4.6 Hz, 1F) ppm; **(*Z*)-isomer:** δ = -83.57 (d, *J* = 6.5 Hz, 3F), -111.97 (d, *J* = 14.4 Hz, 1F), -119.82 – -119.90 (m, 2F), -141.44 – -141.65 (m, 1F) ppm.

^{13}C NMR (100 MHz, CDCl$_3$) of (*E*)-isomer: δ = 173.7 (d, *J* = 2.6 Hz), 159.9, 147.5–143.6 (m, 1C), 143.3 (d, *J* = 2.0 Hz), 133.3, 131.9, 131.7, 129.7, 129.2–129.0 (m, 1C), 127.98, 127.95, 124.8,

114.2, 91.2, 87.9, 55.4, 34.3, 28.5 ppm; carbons corresponding to the C_2F_5 group cannot be identified due to C-F coupling.
HRMS (m/z): calcd for $C_{26}H_{21}F_7NO_2$ $[M+H]^+$ 512.1455, found: 512.1453.

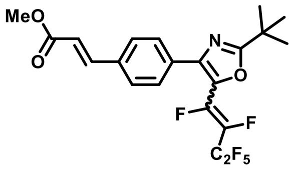

Methyl (2*E*)-3-(4-(2-(*tert*-butyl)-5-(perfluorobut-1-en-1-yl)oxazol-4-yl)phenyl)acrylate (9):
Yield = 70% (98.3 mg, *Z*/*E* = 1/2.1). Yellow oil.
IR (KBr): ν = 2975, 1717, 1635, 1328, 980, 842, 519 cm^{-1}.

^{1}H NMR (400 MHz, $CDCl_3$): δ = 7.79–7.75 (m, 1H), 7.72–7.66 (m, 2H), 7.60–7.55 (m, 2H), 6.51–6.45 (m, 1H), 3.82–3.79 (m, 3H), 1.47–1.41 (m, 9H) ppm.
^{19}F NMR (376 MHz, $CDCl_3$) of (*E*)-isomer: δ = -84.10 – -84.28 (m, 3F), -120.05 (ddd, *J* = 23.3, 12.5, 5.7 Hz, 2F), -146.81 – -147.52 (m, 1F), -159.85 – -160.42 (m, 1F) ppm; **(*Z*)-isomer:** δ = -83.65 (d, *J* = 6.6 Hz, 3F), -112.24 (dd, *J* = 14.0, 6.0 Hz, 1F), -119.96 (d, *J* = 13.9 Hz, 2F), -141.39 (dt, *J* = 21.0, 6.4 Hz, 1F) ppm.
^{13}C NMR (100 MHz, $CDCl_3$) of (*E*)-isomer: δ = 173.8 (d, *J* = 2.7 Hz), 167.4, 147.7–144.5 (m, 1C), 144.0, 143.0 (d, *J* = 3.2 Hz), 135.3, 132.1, 131. 6–131.3 (m, 1C), 128.6, 128.5, 128.3, 118.8, 51.9, 34.3, 28.4 ppm; carbons corresponding to the C_2F_5 group cannot be identified due to C-F coupling.
HRMS (m/z): calcd for $C_{21}H_{19}F_7NO_3$ $[M+H]^+$ 466.1248, found: 466.1246.

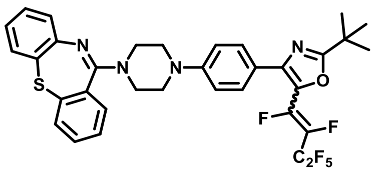

2-(*tert*-Butyl)-4-(4-(4-(dibenzo[*b*,*f*][1,4]thiazepin-11-yl)piperazin-1-yl)phenyl)-5-(perfluorobut-1-en-1-yl)oxazole (11):
Yield = 72% (144.9 mg, *Z*/*E* = 1/2.1). Yellow oil.
IR (KBr): ν = 2975, 2844, 1606, 1513, 1228, 1011, 761 cm^{-1}.
^{1}H NMR (400 MHz, $CDCl_3$): δ = 7.77–7.64 (m, 2H), 7.61–7.44 (m, 2H), 7.43–7.32 (m, 3H), 7.25–7.16 (m, 2H), 7.04–6.92 (m, 3H), 3.99–3.11 (m, 8H), 1.55–1.44 (m, 9H) ppm.
^{19}F NMR (376 MHz, $CDCl_3$) of (*E*)-isomer: δ = -84.09 (d, *J* = 6.0 Hz, 3F), -119.74 – -119.84 (m, 2F), -146.55 – -147.36 (m, 1F), -161.20 – -161.80 (m, 1F) ppm; **(*Z*)-isomer:** δ = -83.49 (d, *J* = 5.9 Hz, 3F), -111.66 (d, *J* = 15.6 Hz, 1F), -119.71 (d, *J* = 13.1 Hz, 2F), -142.84 (qd, *J* = 16.3, 8.2 Hz, 1F) ppm.
^{13}C NMR (100 MHz, $CDCl_3$) of (*E*)-isomer: δ = 173.3 (d, *J* = 2.8 Hz), 160.9, 151.7, 148.8, 148.0–144.1 (m, 1C), 143.9 (d, *J* = 3.3 Hz), 140.1, 134.1, 132.3, 131.1, 130.7–129.9 (m, 1C), 129.3, 129.11, 129.08, 128.5, 128.3, 128.1, 125.4, 123.2, 121.2, 115.7, 115.5, 48.3, 34.2, 28.5, 28.3 ppm; carbons corresponding to the C_2F_5 group cannot be identified due to C-F coupling.
HRMS (m/z): calcd for $C_{34}H_{30}F_7N_4OS$ $[M+H]^+$ 675.2023, found: 675.2022.

The X-ray crystal structure of product (*E*)-3va

(*E*)-5-(Perfluorobut-1-en-1-yl)-2,4-diphenyloxazole [(*E*)-3va; displacement ellipsoids are drawn at the 50% probability levels**]:**

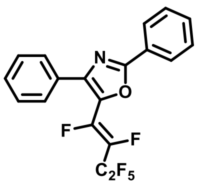

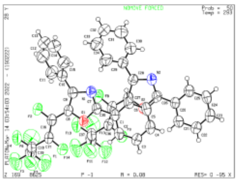

The single crystal was grown from the mixed solution of DCM/EtOAc/DMF (v/v/v = 10/3/1) by slowly evaporating the above solvents at room temperature.

Table S2. Crystal data and structure refinement for product **(*E*)-3va**.

Identification code	**(*E*)-3va**
Empirical formula	$C_{19}H_{10}F_7NO$
Formula weight	401.28
Temperature/K	293(2)
Crystal system	triclinic
Space group	P-1
a/Å	10.1707(12)
b/Å	10.8048(13)
c/Å	16.5700(18)
α/°	79.087(10)
β/°	76.566(10)
γ/°	85.923(10)
Volume/Å^3	1738.4(4)
Z	4
ρ_{calc}g/cm^3	1.533
μ/mm^{-1}	1.306
F(000)	808.0
Crystal size/mm^3	0.16 × 0.12 × 0.10
Radiation	Cu Kα (λ = 1.54184)
2Θ range for data collection/°	5.572 to 133.182
Index ranges	$-12 \le h \le 12$, $-12 \le k \le 12$, $-19 \le l \le 19$
Reflections collected	13418
Independent reflections	5899 [R_{int} = 0.0414, R_{sigma} = 0.0751]
Data/restraints/parameters	5899/0/505
Goodness-of-fit on F^2	0.977
Final R indexes [I>=2σ (I)]	R_1 = 0.0772, wR_2 = 0.2304
Final R indexes [all data]	R_1 = 0.1193, wR_2 = 0.2902
Largest diff. peak/hole / e Å^{-3}	0.42/-0.28

Crystal structure determination of product (*E*)-3va

Crystal Data for $C_{19}H_{10}F_7NO$ (M =401.28 g/mol): triclinic, space group P-1 (no. 2), a = 10.1707(12) Å, b = 10.8048(13) Å, c = 16.5700(18) Å, α = 79.087(10)°, β = 76.566(10)°, γ = 85.923(10)°, V = 1738.4(4) Å^3, Z = 4, T = 293(2) K, μ(Cu Kα) = 1.306 mm^{-1}, $Dcalc$ = 1.533 g/cm^3, 13418 reflections measured (5.572° ≤ 2Θ ≤ 133.182°), 5899 unique (R_{int} = 0.0414, R_{sigma} = 0.0751) which were used in all calculations. The final R_1 was 0.0772 (I > 2σ(I)) and wR_2 was 0.2902 (all data).

References

[1] Zhu, T.; Xie, S.; Rojsitthisak, P.; Wu, J. *Org. Biomol. Chem.* **2020**, *18*, 1504–1521.

[2] Hanack, M.; Ullmann, J. *J. Org. Chem.* **1989**, *54*, 1432–1435.

[3] Yu, W.; Chen, J.; Gao, K.; Liu, Z.; Zhang, Y. *Org. Lett.* **2014**, *16*, 4870–4873.

[4] Epple, R.; Cow, C.; Xie, Y.; Azimioara, M.; Russo, R.; Wang, X.; Wityak, J.; Karanewsky, D. S.; Tuntland, T.; Nguyêñ-Trên, V. T. B.; Ngo, C. C.; Huang, D.; Saez, E.; Spalding, T.; Gerken, A.; Iskandar, M.; Seidel, M.; Tian S.-S. *J. Med. Chem.* **2010**, *53*, 77–105.

[5] Li, J.; Liu, L.; Zheng, K.; Zheng, C.; Xiao, H.; Fan, S. *J. Org. Chem*. **2020**, *85*, 8723–8731.

[6] Zhao, H.-Y.; Zhou, M.; Zhang, X. *Org. Lett.* **2021**, *23,* 9106–9111.

[7] Pan, Z.; Fan, Z.; Lu, B.; Cheng, J. *Adv. Synth. Catal.* **2018**, *360*, 1761–1767.

[8] Sun, X.; Wang, W.; Li, Y.; Ma, J.; Yu, S. *Org. Lett.* **2016**, *18*, 4638–4641.

^{1}H, ^{19}F, and ^{13}C NMR spectra of products

^{1}H NMR spectra of the product **3aa** (400 MHz, $CDCl_3$)

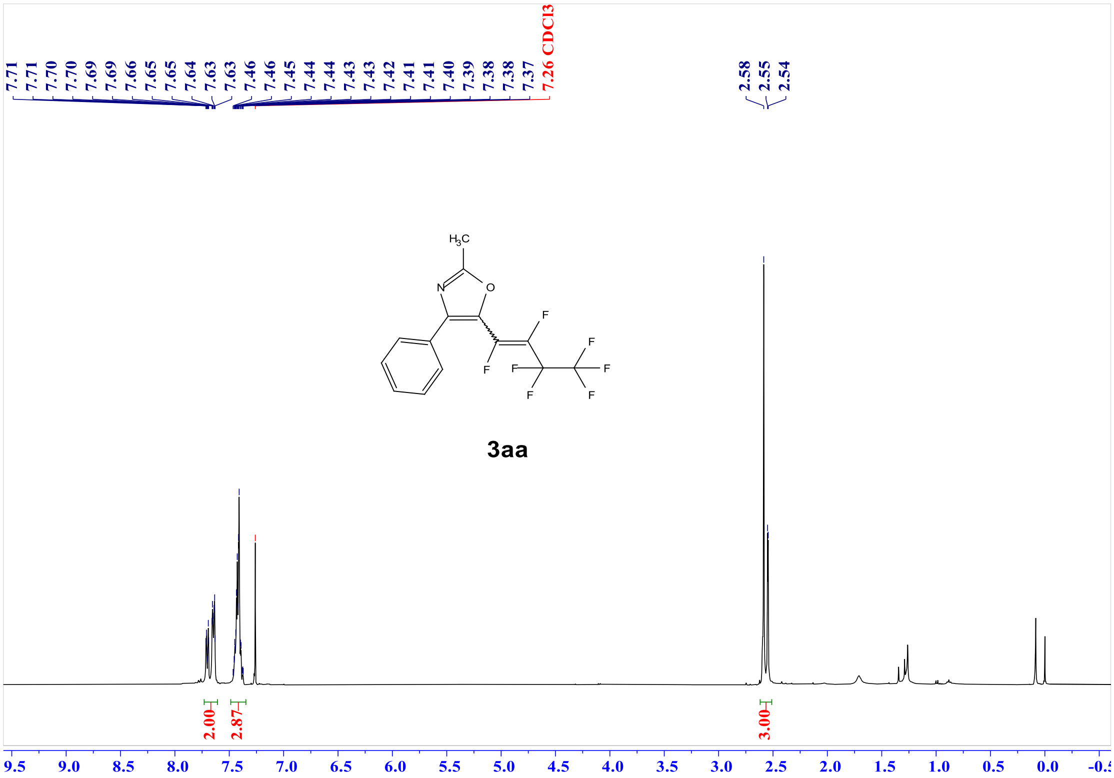

^{19}F NMR spectra of the product **3aa** (376 MHz, $CDCl_3$)

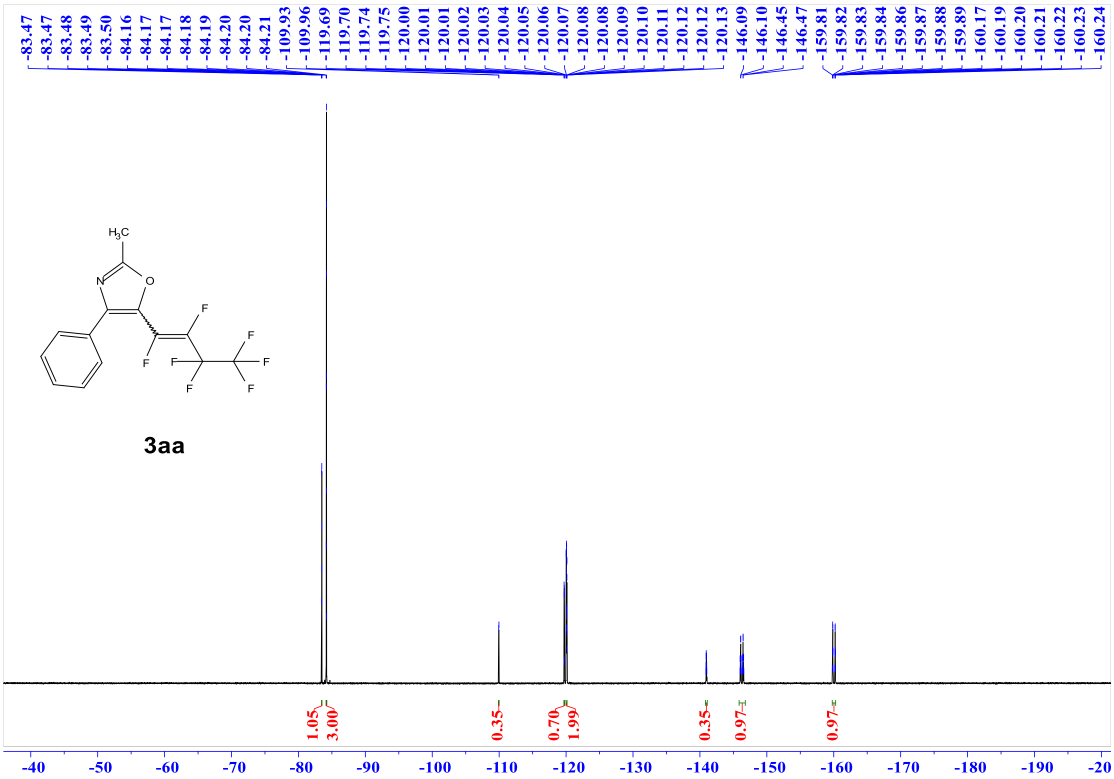

^{13}C NMR spectra of the product **3aa** (100 MHz, $CDCl_3$)

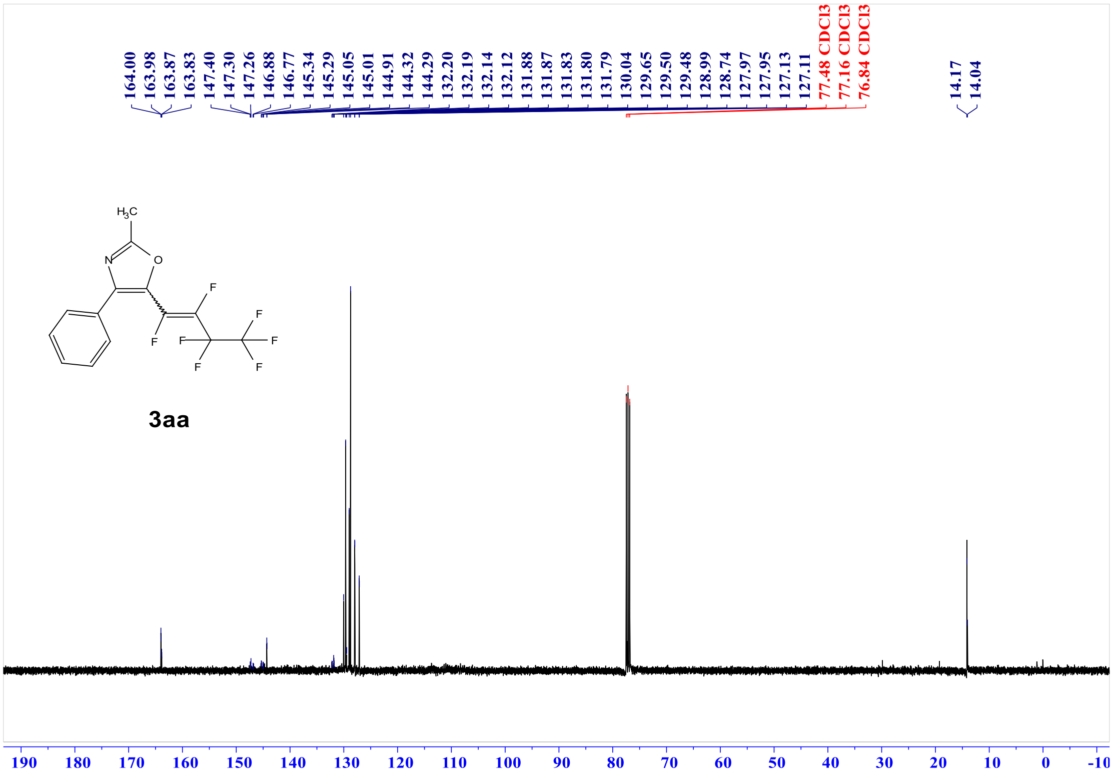

^{1}H NMR spectra of the product **3ba** (400 MHz, $CDCl_3$)

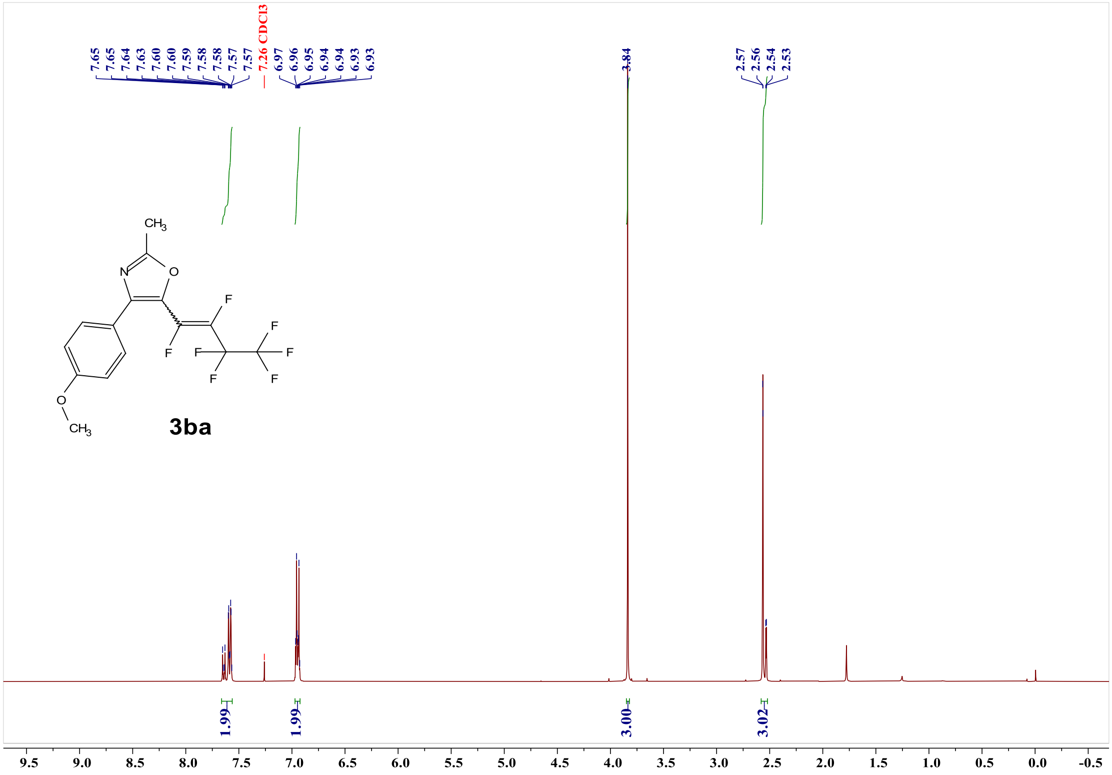

^{19}F NMR spectra of the product **3ba** (376 MHz, $CDCl_3$)

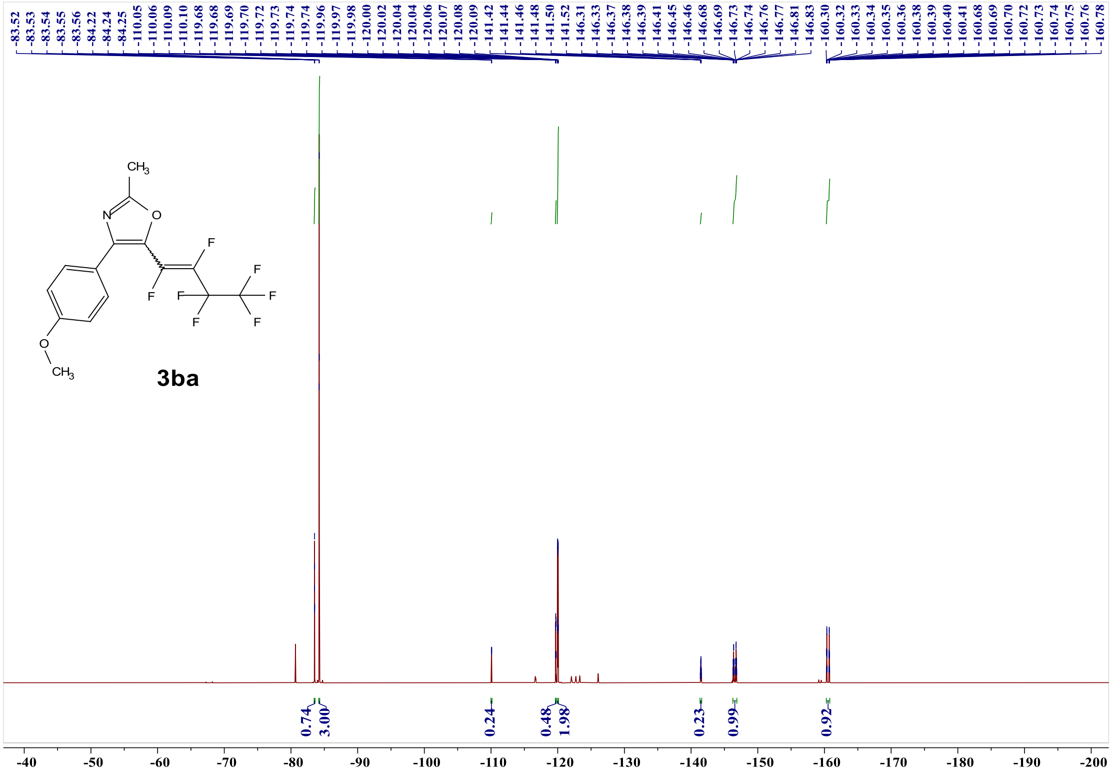

^{13}C NMR spectra of the product **3ba** (100 MHz, $CDCl_3$)

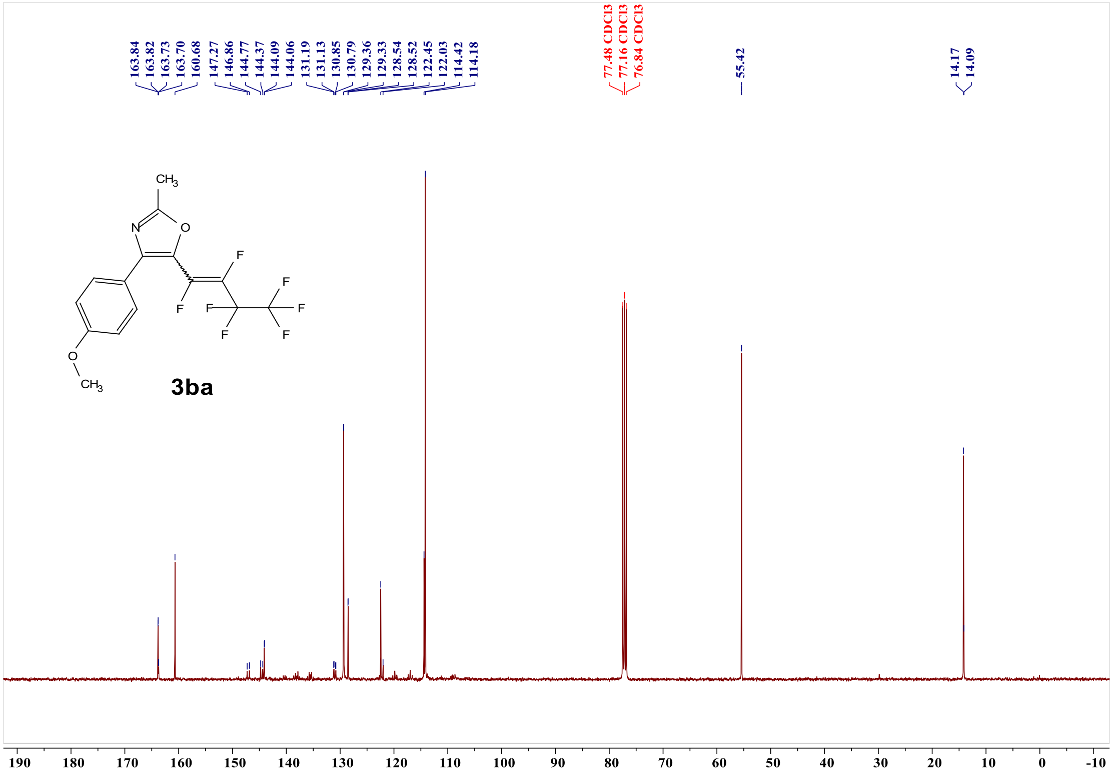

^{1}H NMR spectra of the product **3ca** (400 MHz, $CDCl_3$)

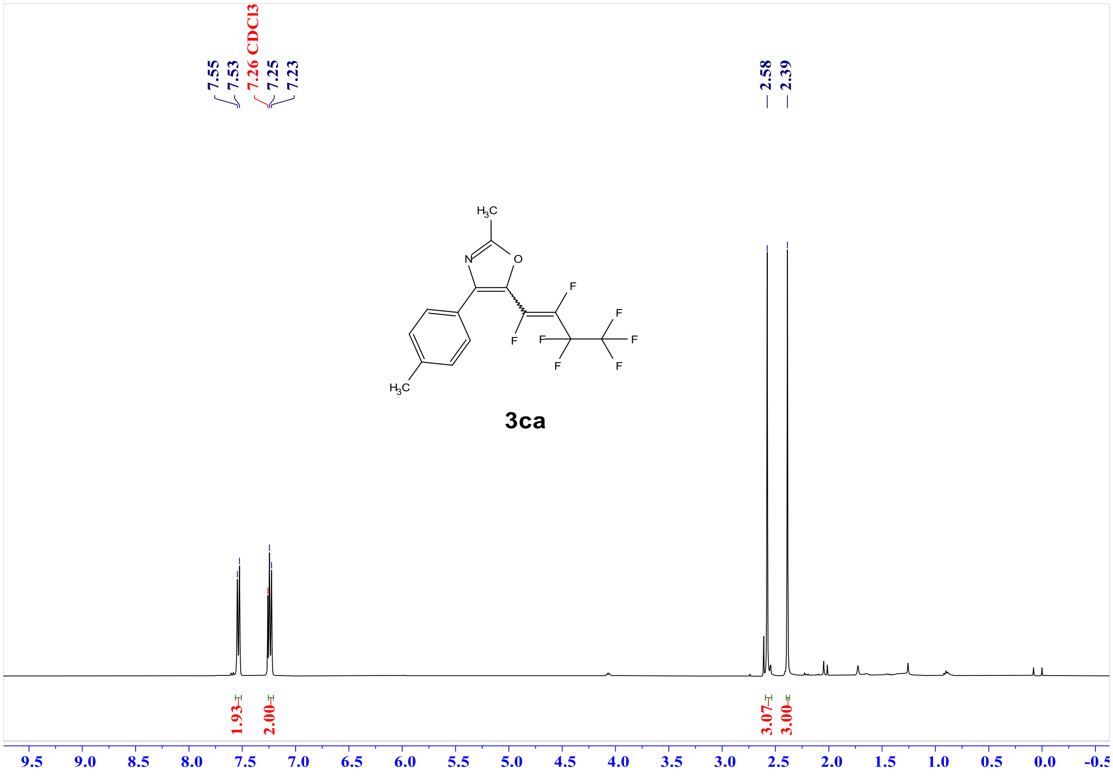

^{19}F NMR spectra of the product **3ca** (376 MHz, $CDCl_3$)

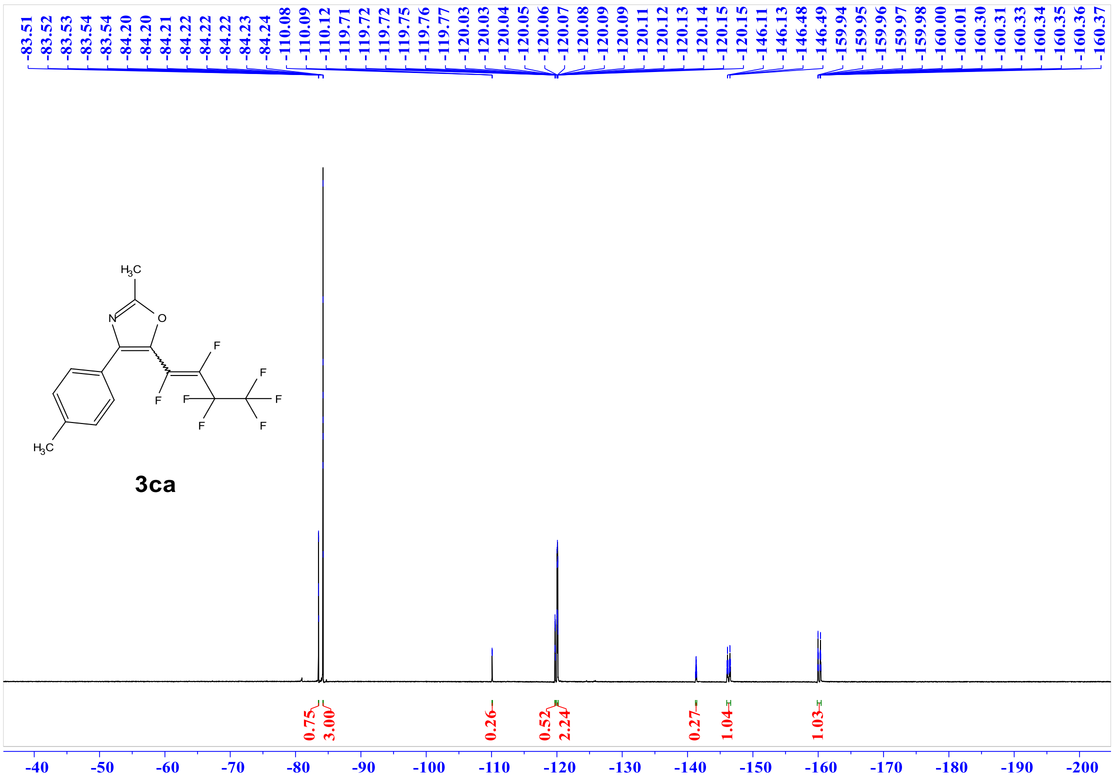

^{13}C NMR spectra of the product **3ca** (100 MHz, $CDCl_3$)

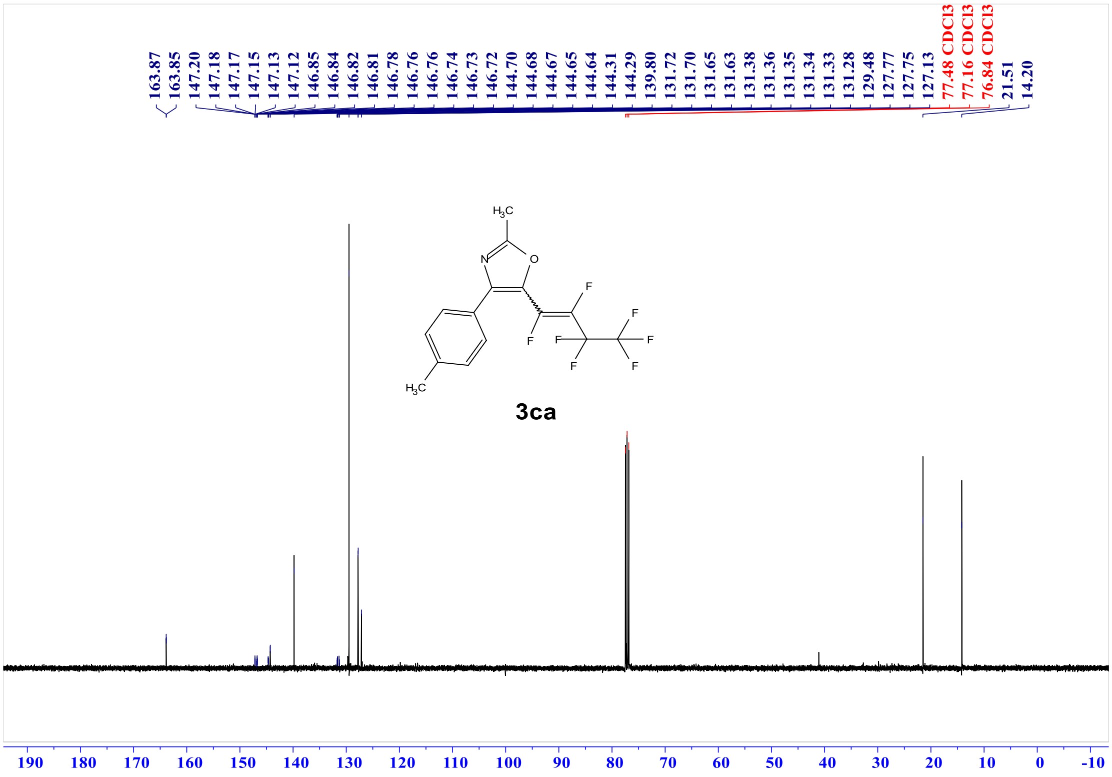

^{1}H NMR spectra of the product **3da** (400 MHz, $CDCl_3$)

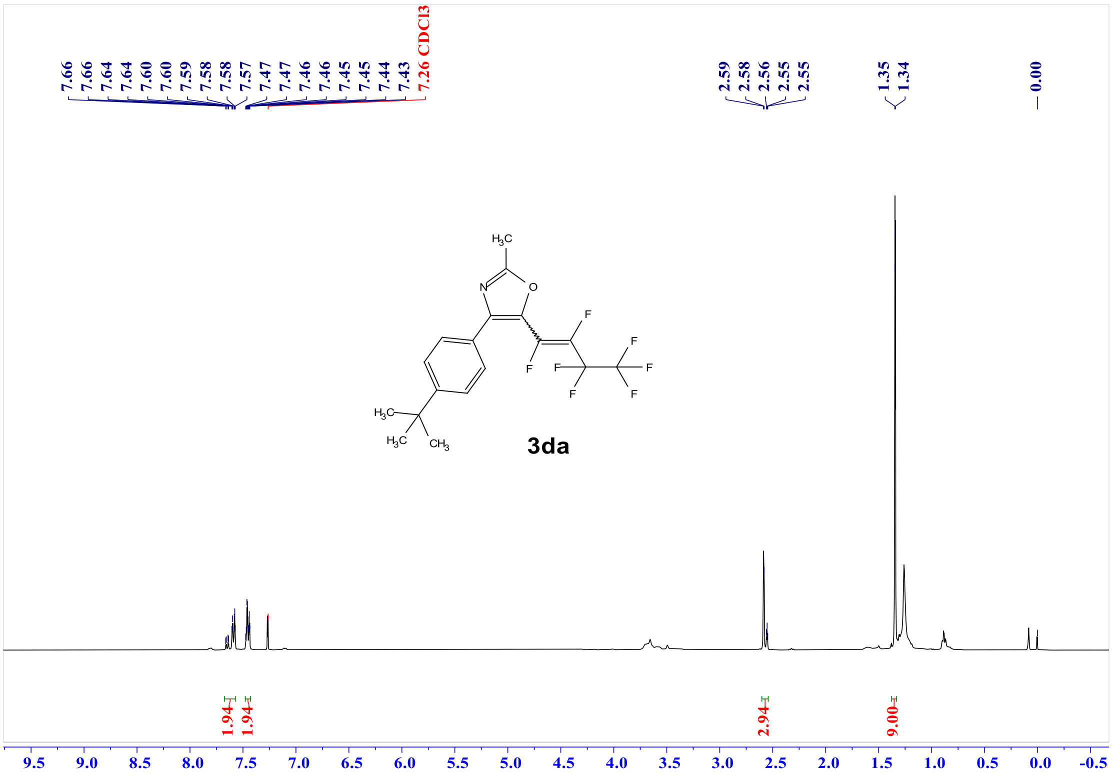

^{19}F NMR spectra of the product **3da** (376 MHz, $CDCl_3$)

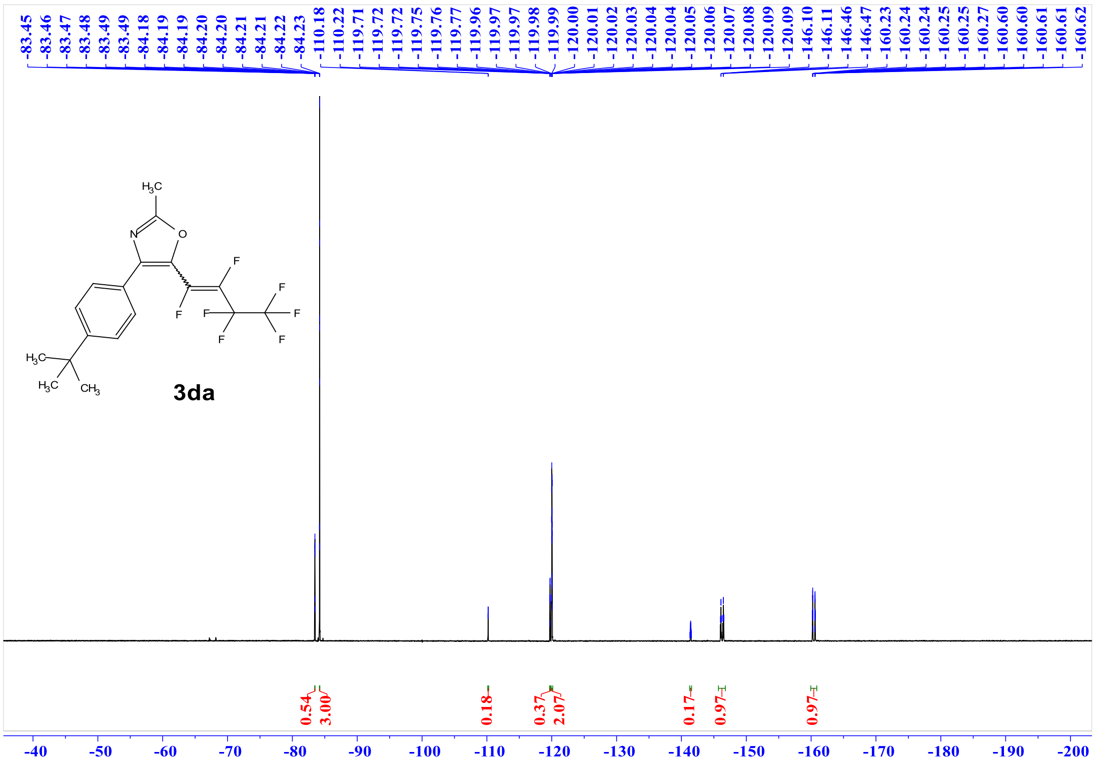

^{13}C NMR spectra of the product **3da** (100 MHz, $CDCl_3$)

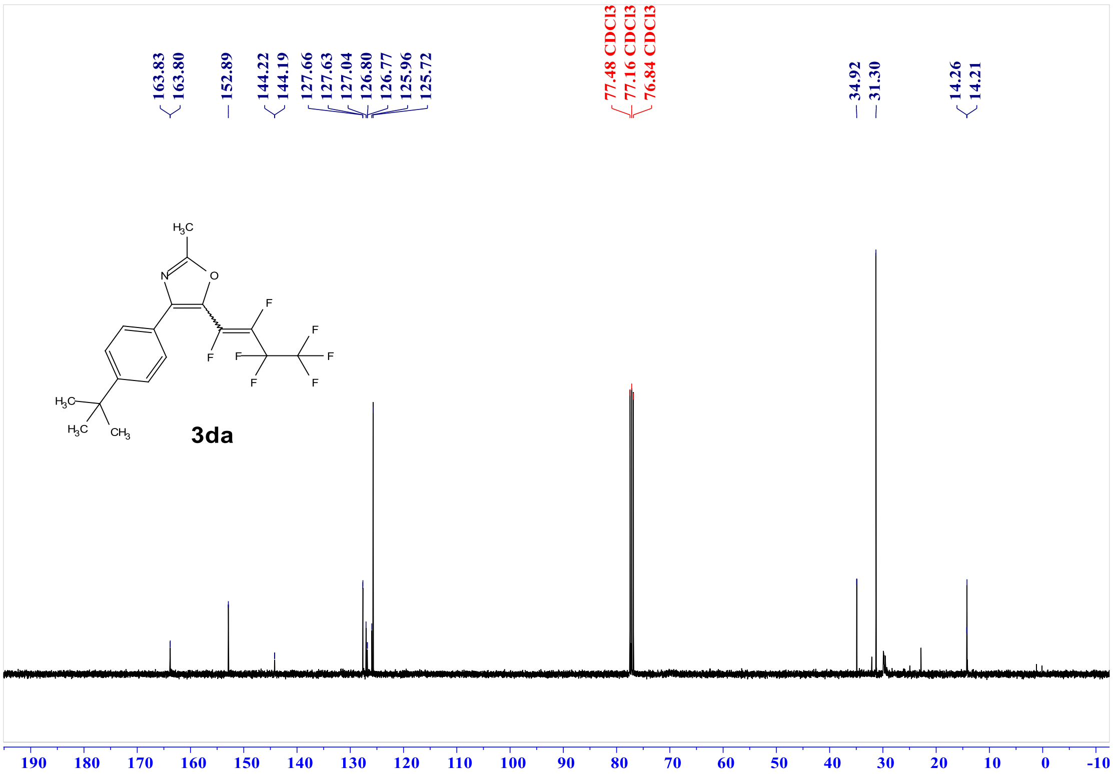

^1H NMR spectra of the product **3ea** (400 MHz, $CDCl_3$)

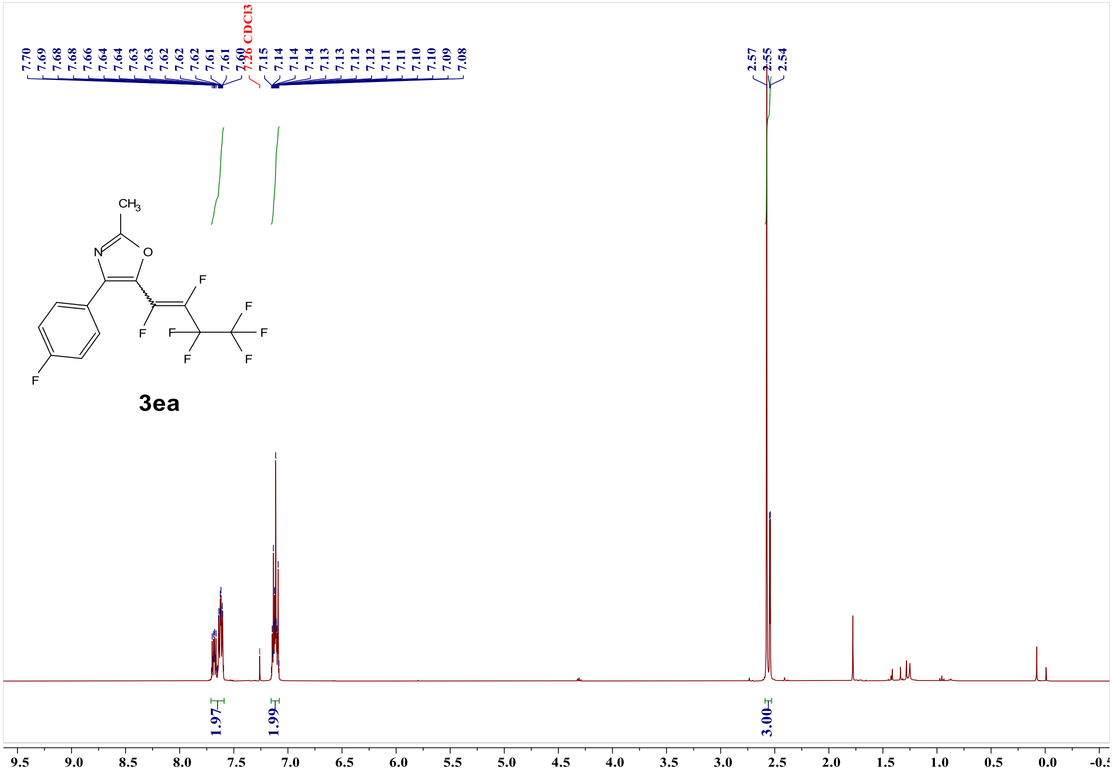

^{19}F NMR spectra of the product **3ea** (376 MHz, $CDCl_3$)

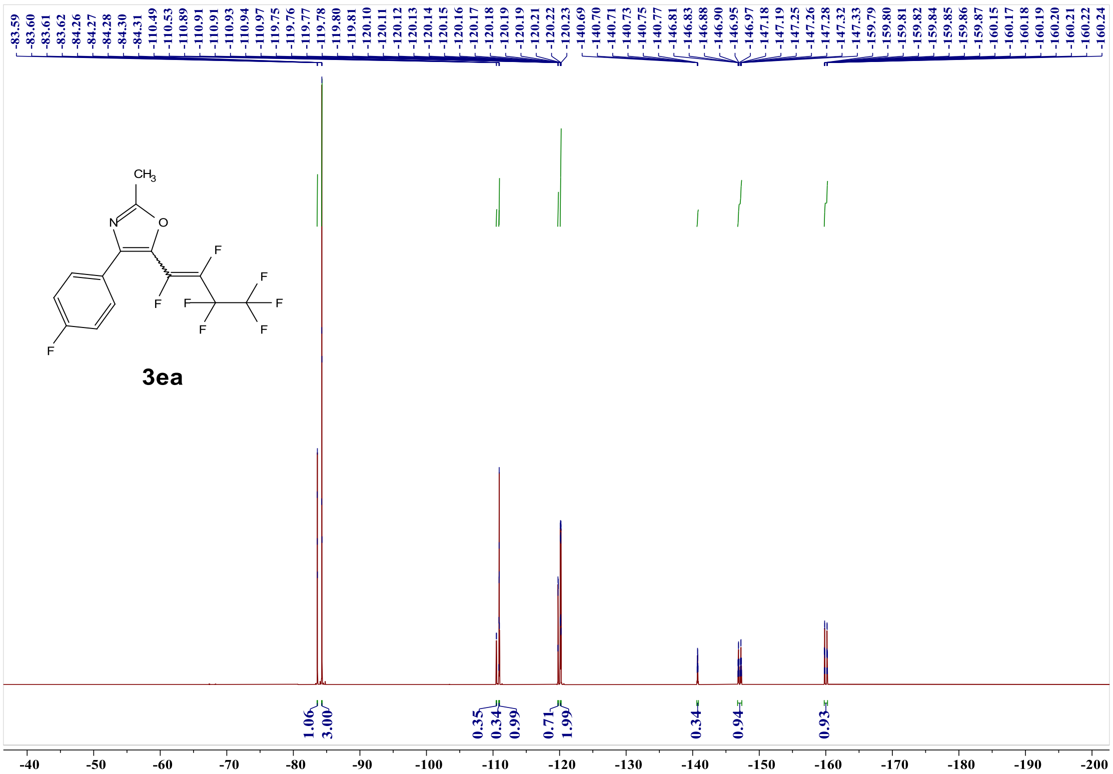

^{13}C NMR spectra of the product **3ea** (100 MHz, $CDCl_3$)

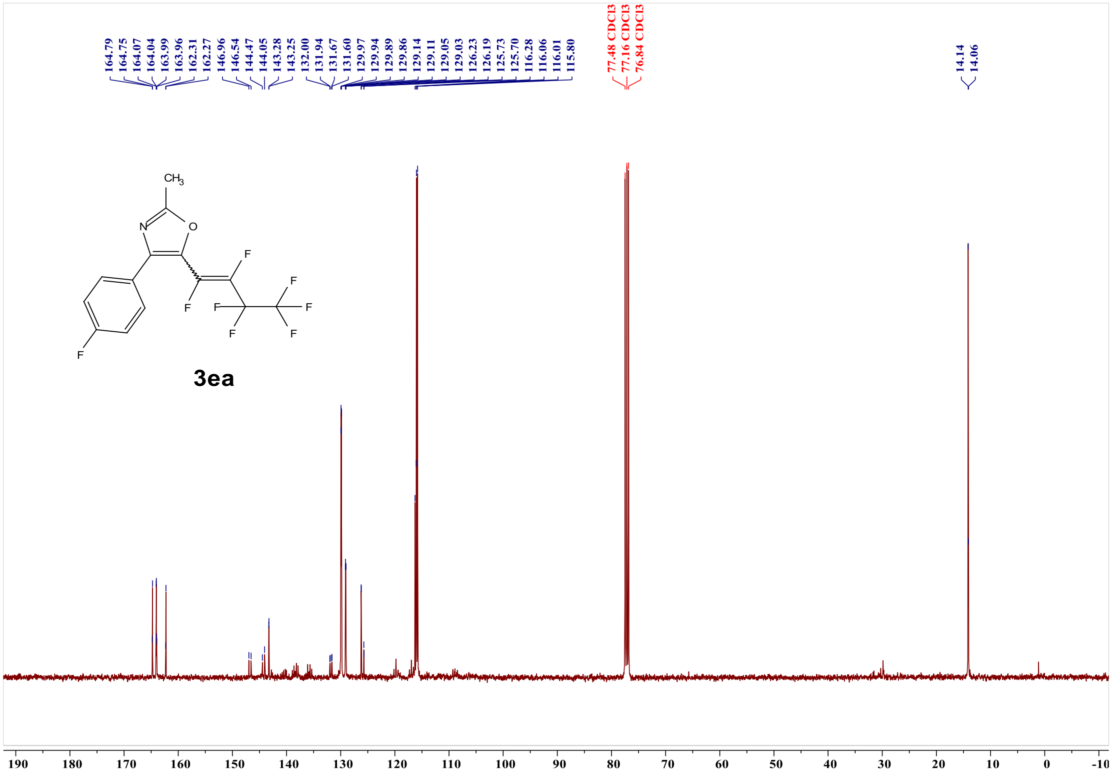

^{1}H NMR spectra of the product **3fa** (400 MHz, $CDCl_3$)

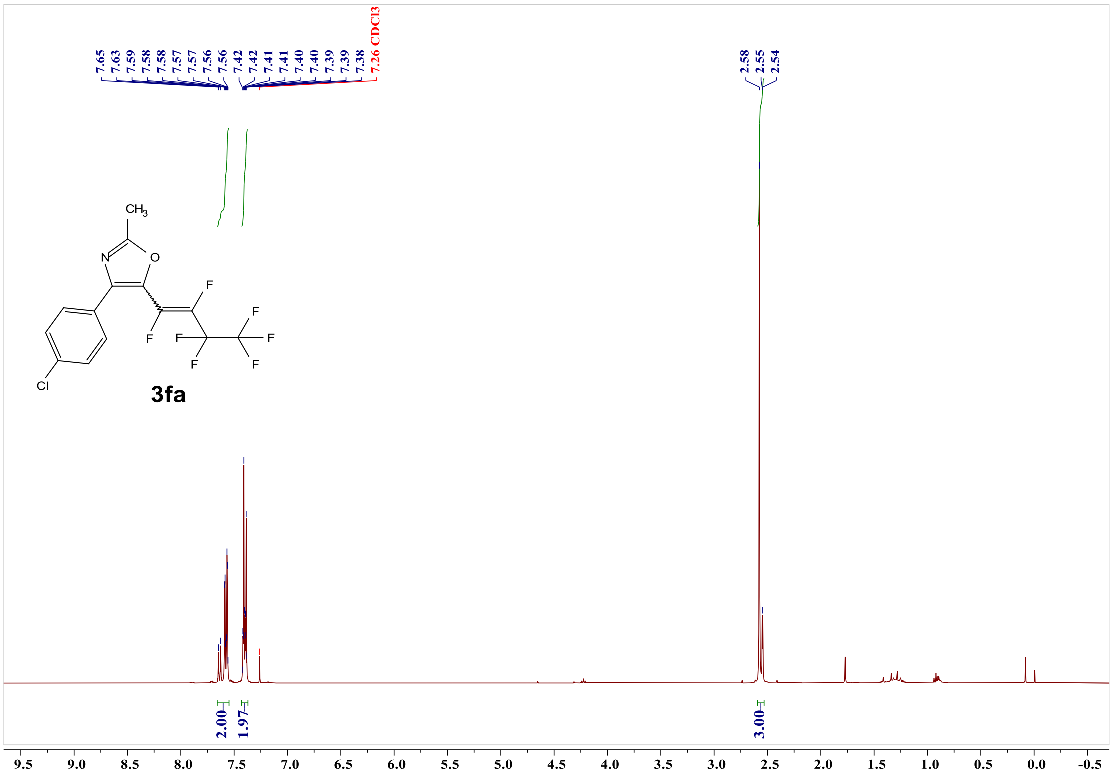

^{19}F NMR spectra of the product **3fa** (376 MHz, $CDCl_3$)

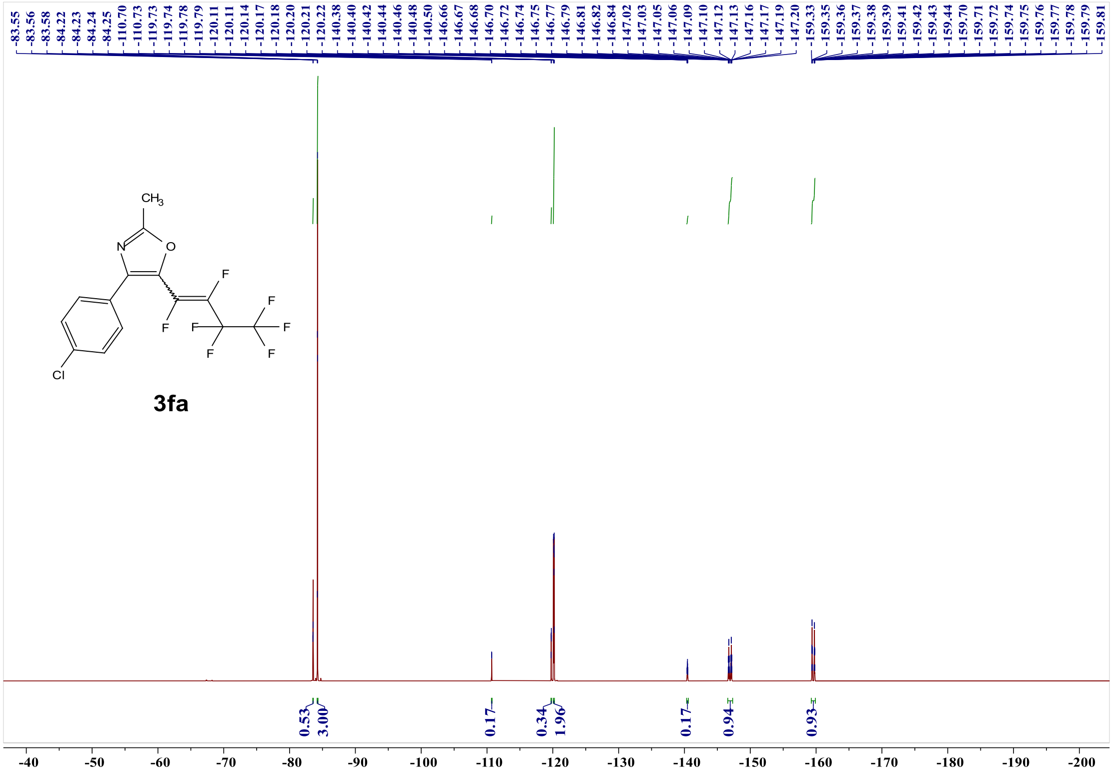

^{13}C NMR spectra of the product **3fa** (100 MHz, $CDCl_3$)

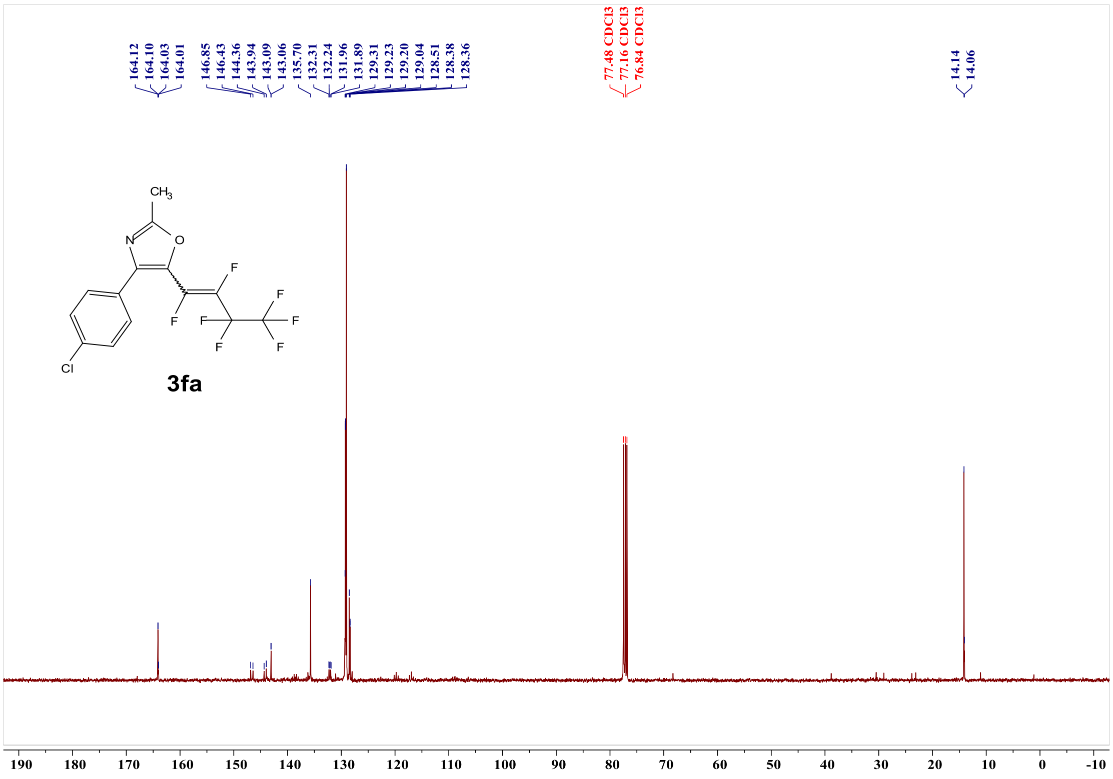

^{1}H NMR spectra of the product **3ga** (400 MHz, $CDCl_3$)

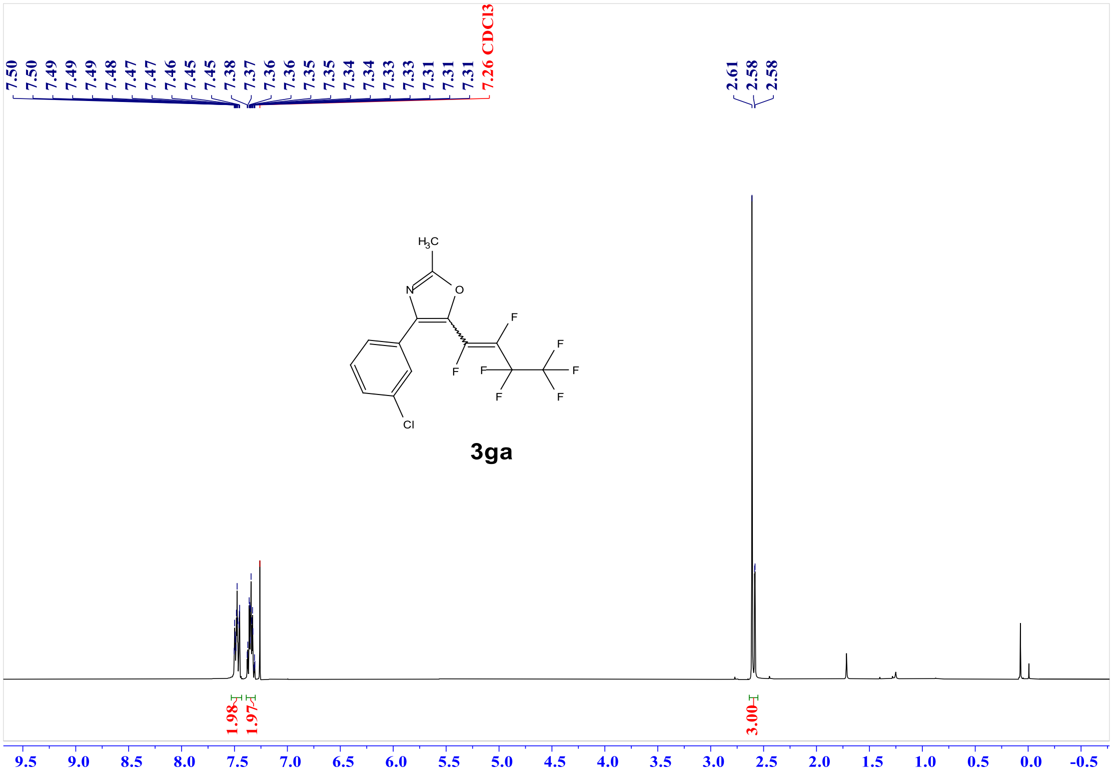

^{19}F NMR spectra of the product **3ga** (376 MHz, $CDCl_3$)

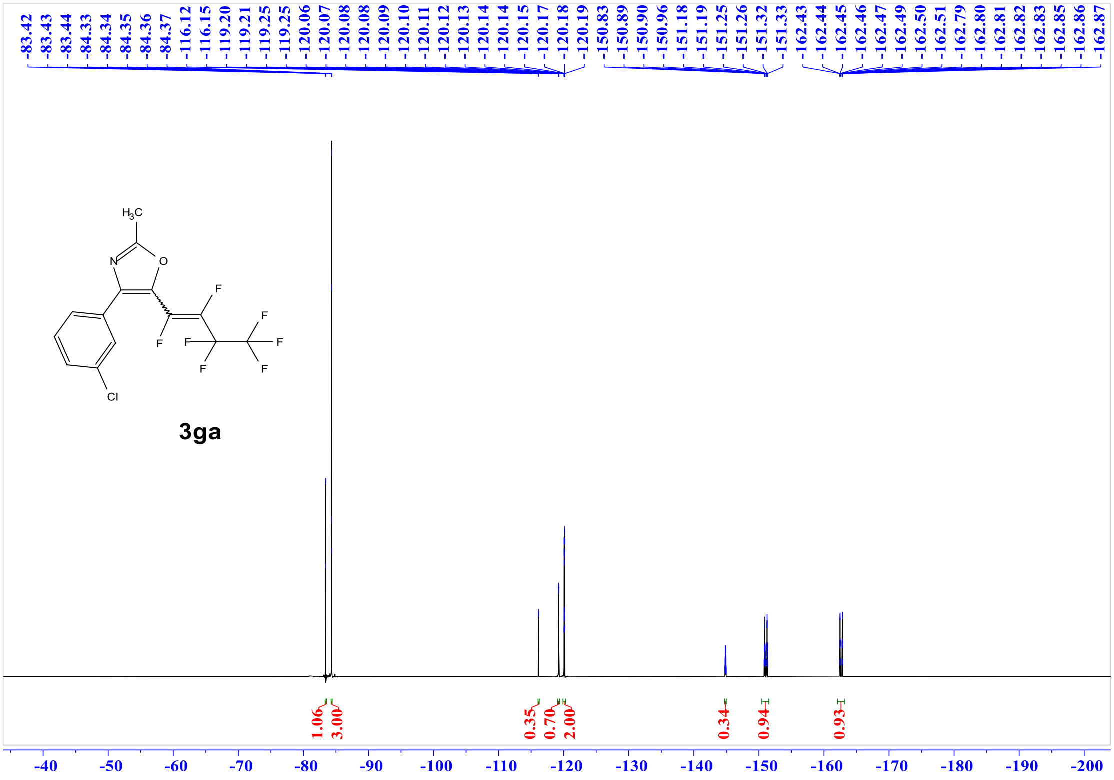

^{13}C NMR spectra of the product **3ga** (100 MHz, $CDCl_3$)

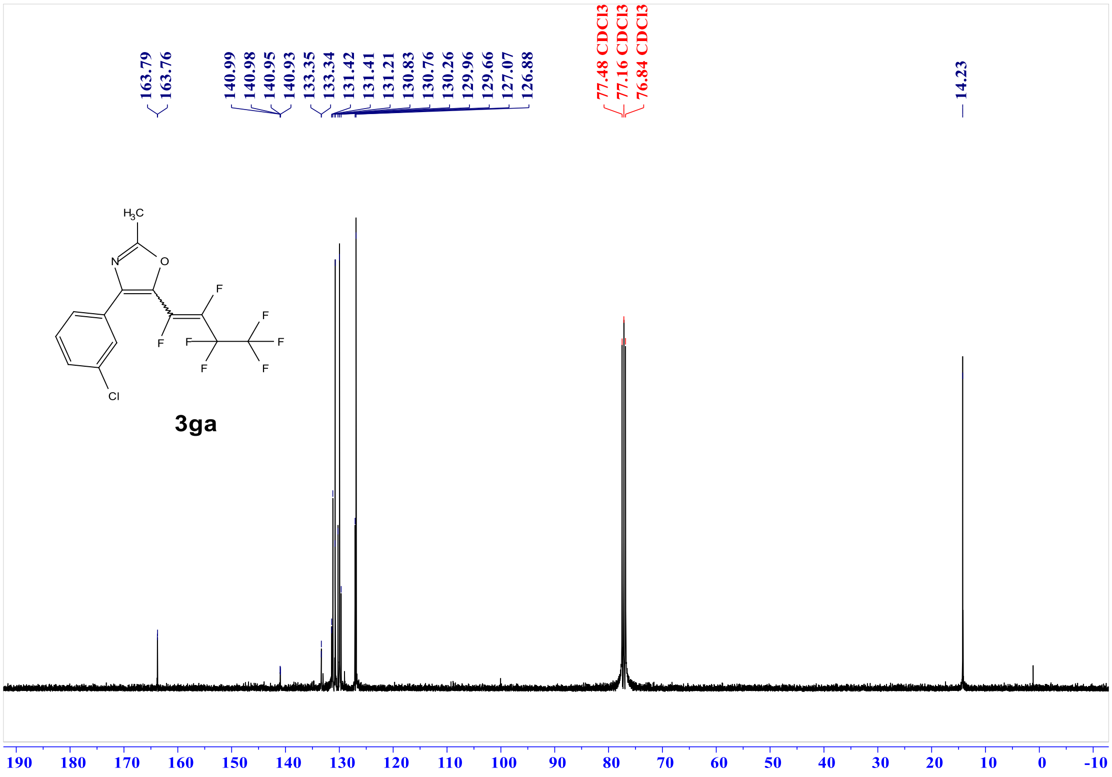

^{1}H NMR spectra of the product **3ha** (400 MHz, $CDCl_3$)

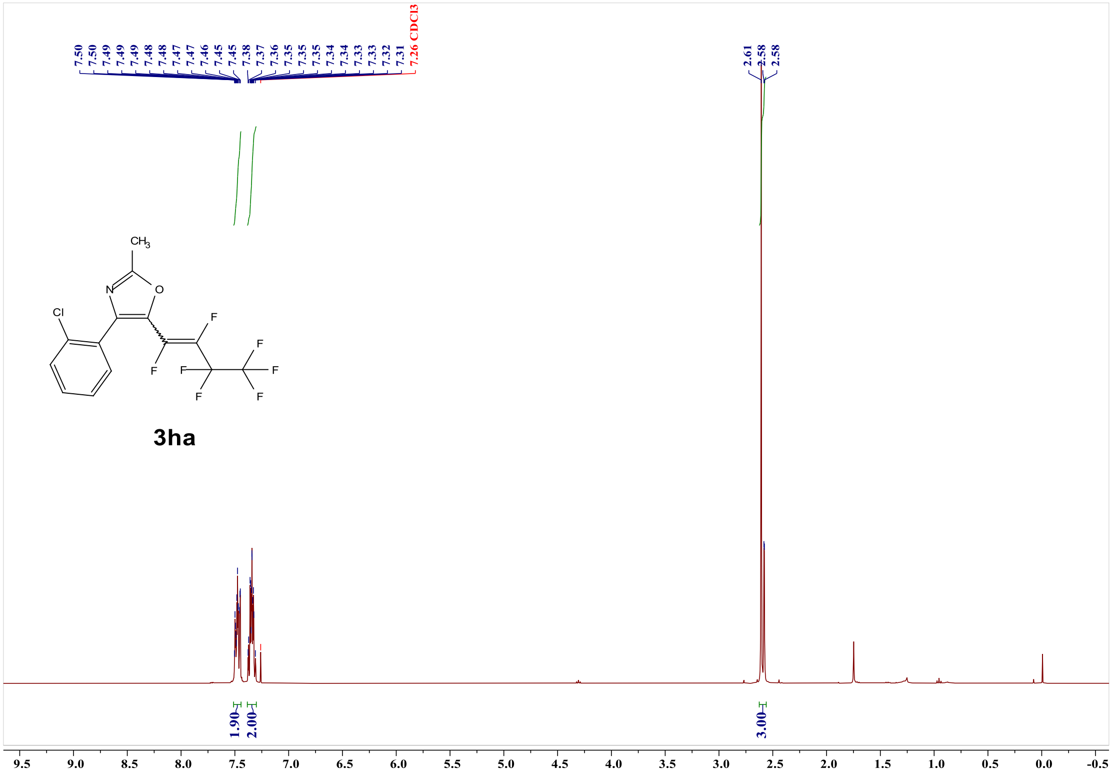

^{19}F NMR spectra of the product **3ha** (376 MHz, $CDCl_3$)

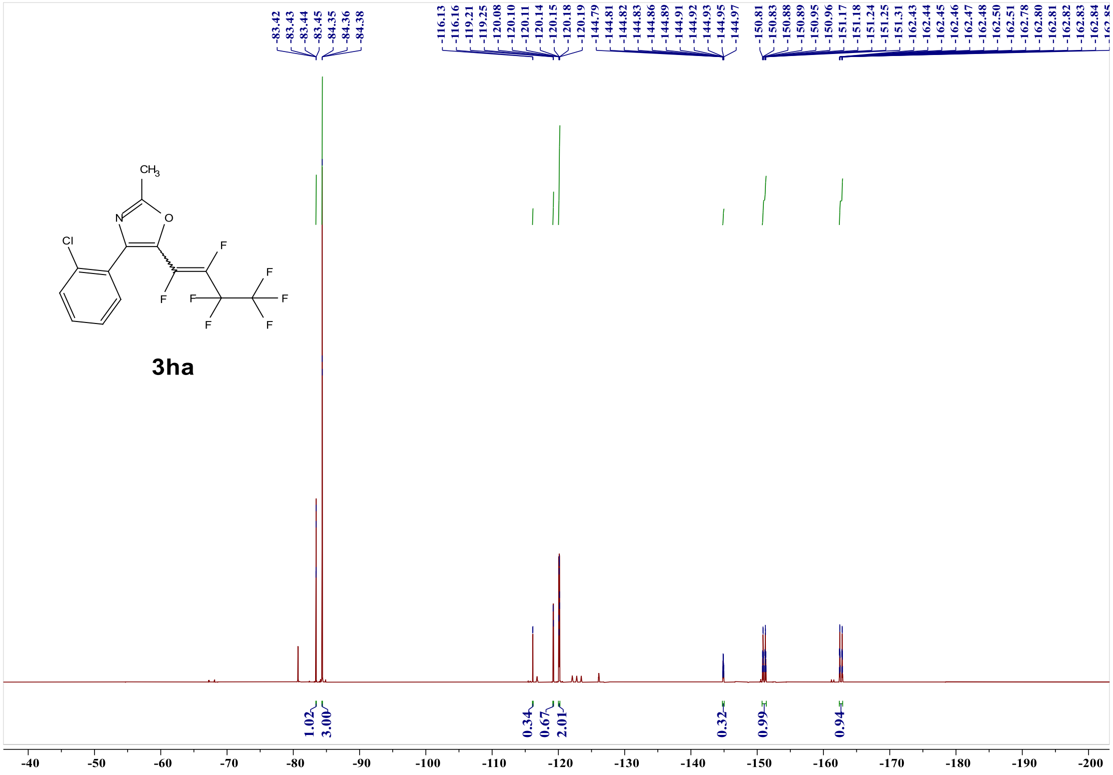

^{13}C NMR spectra of the product **3ha** (100 MHz, $CDCl_3$)

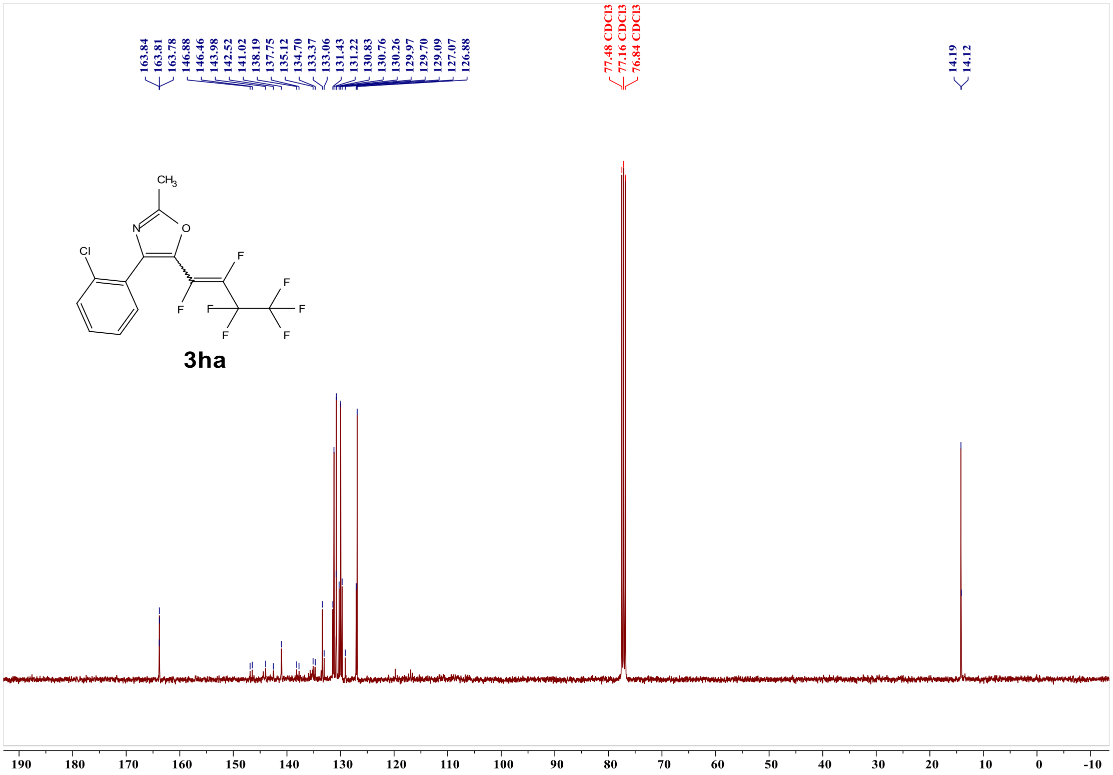

^{1}H NMR spectra of the product **3ia** (400 MHz, $CDCl_3$)

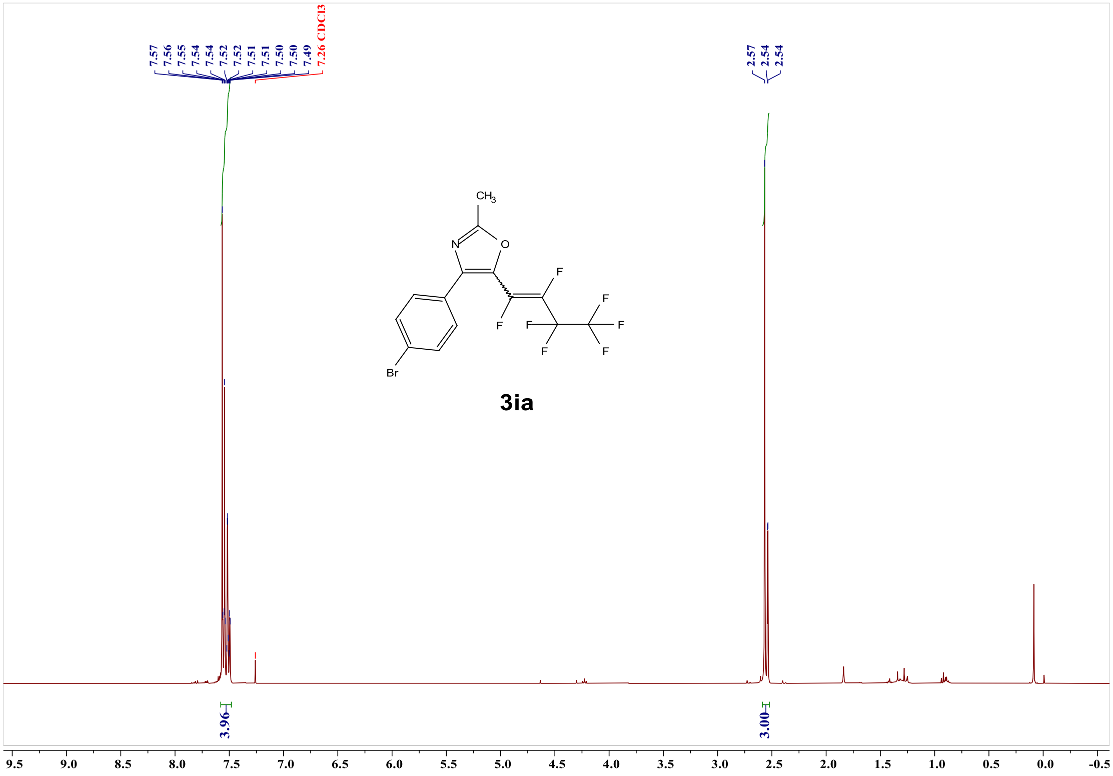

^{19}F NMR spectra of the product **3ia** (376 MHz, $CDCl_3$)

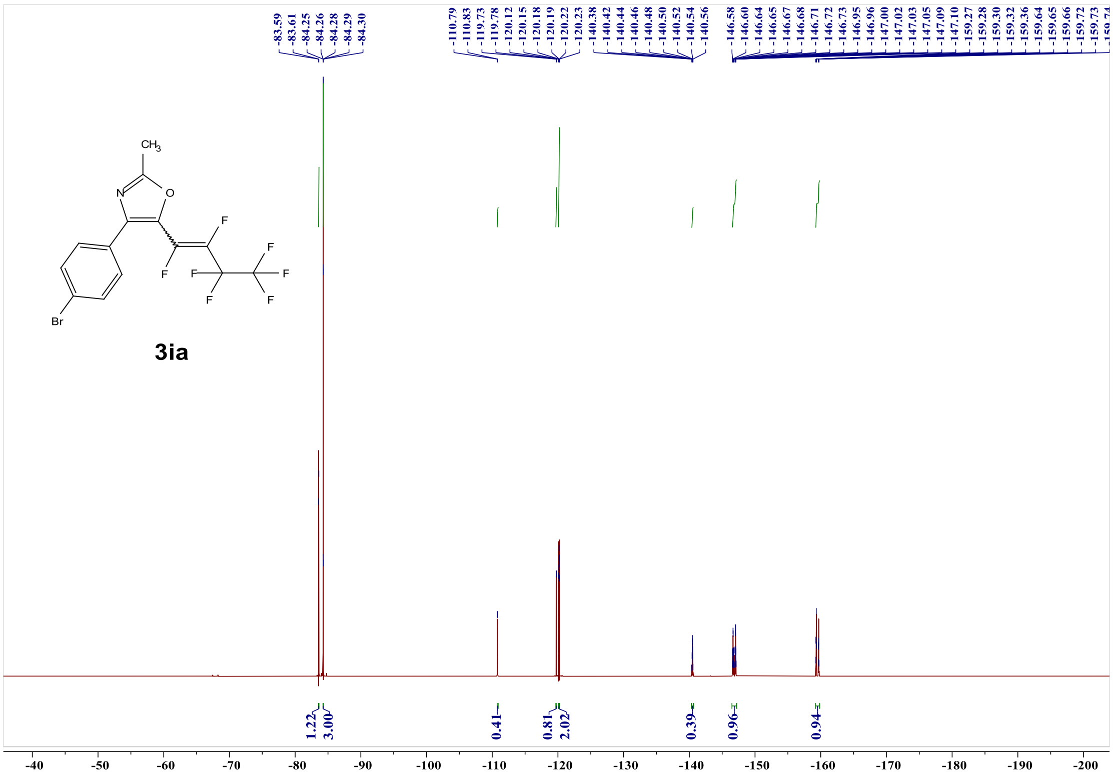

^{13}C NMR spectra of the product **3ia** (100 MHz, $CDCl_3$)

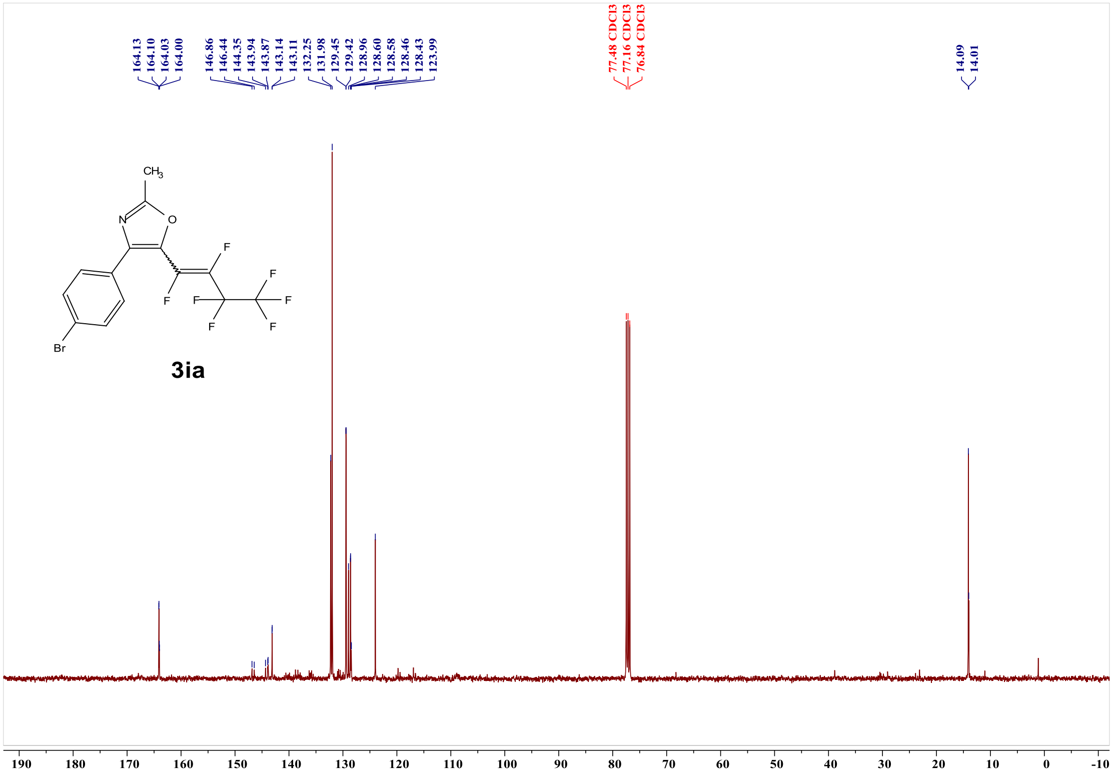

^{1}H NMR spectra of the product **3ja** (400 MHz, $CDCl_3$)

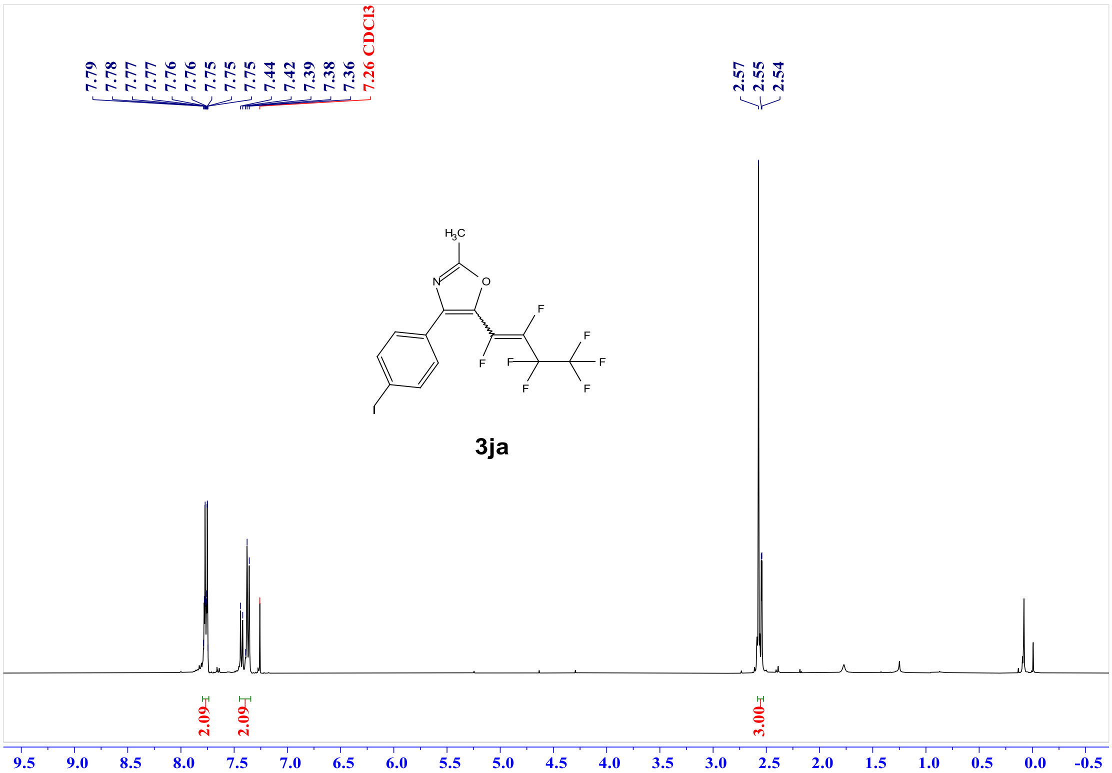

^{19}F NMR spectra of the product **3ja** (376 MHz, $CDCl_3$)

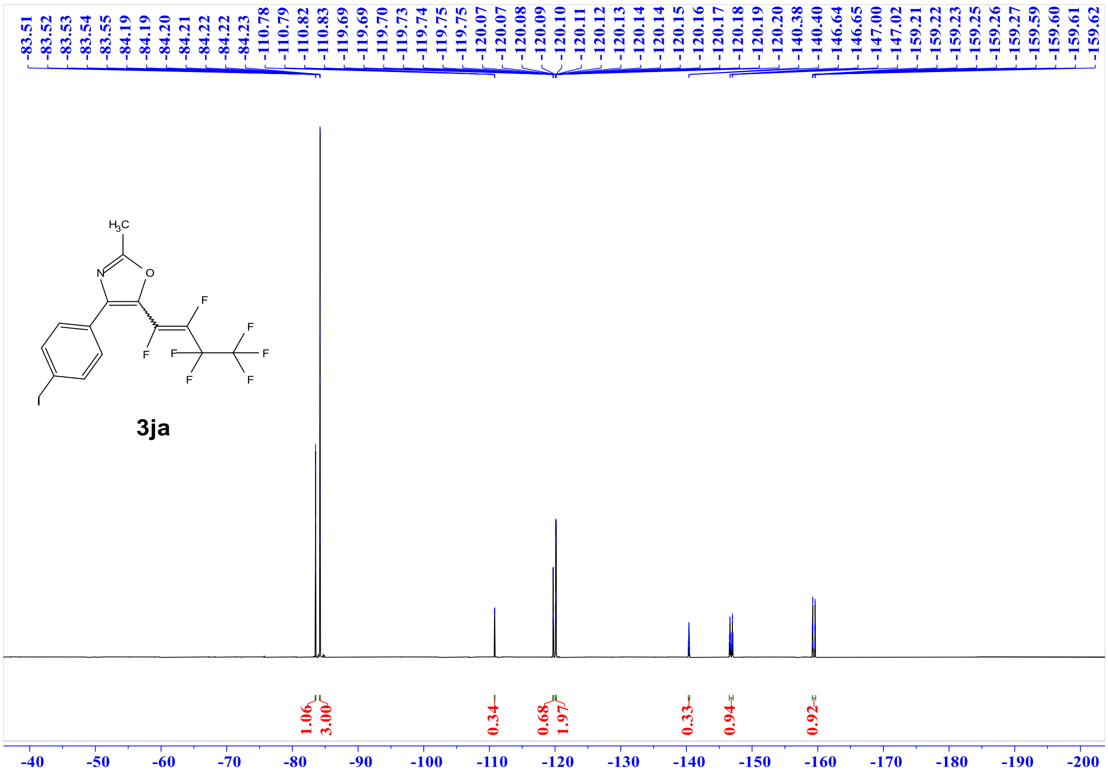

^{13}C NMR spectra of the product **3ja** (100 MHz, $CDCl_3$)

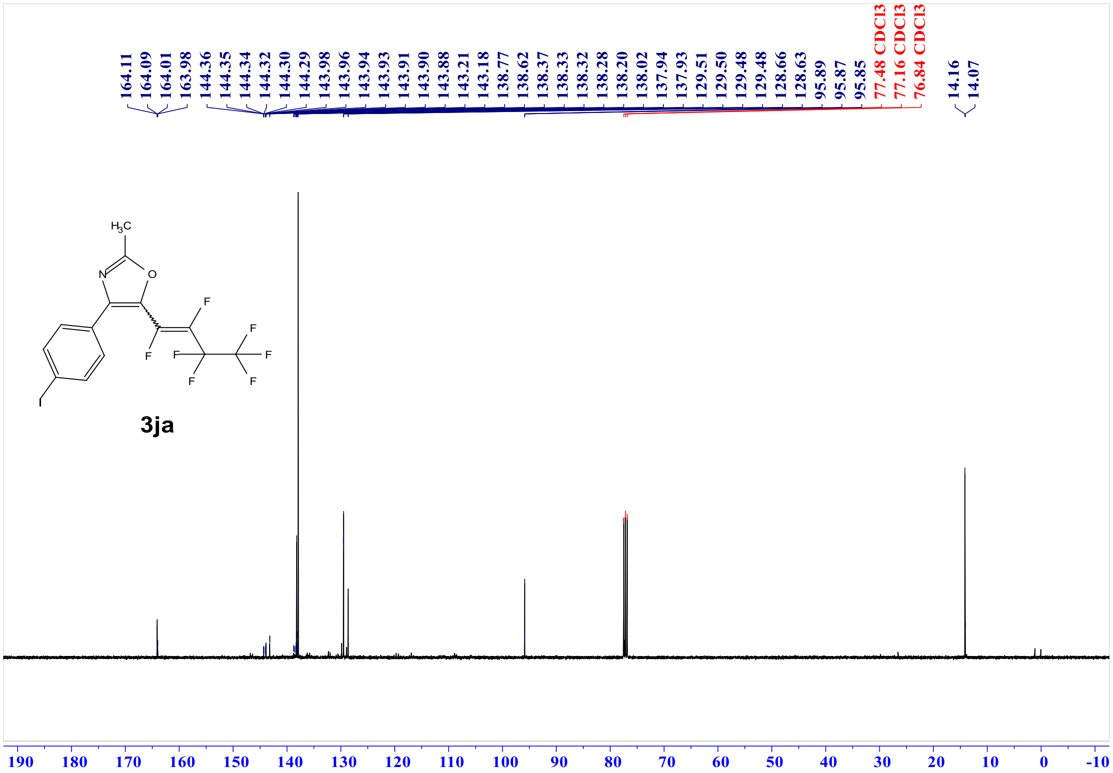

^{1}H NMR spectra of the product **3ka** (400 MHz, $CDCl_3$)

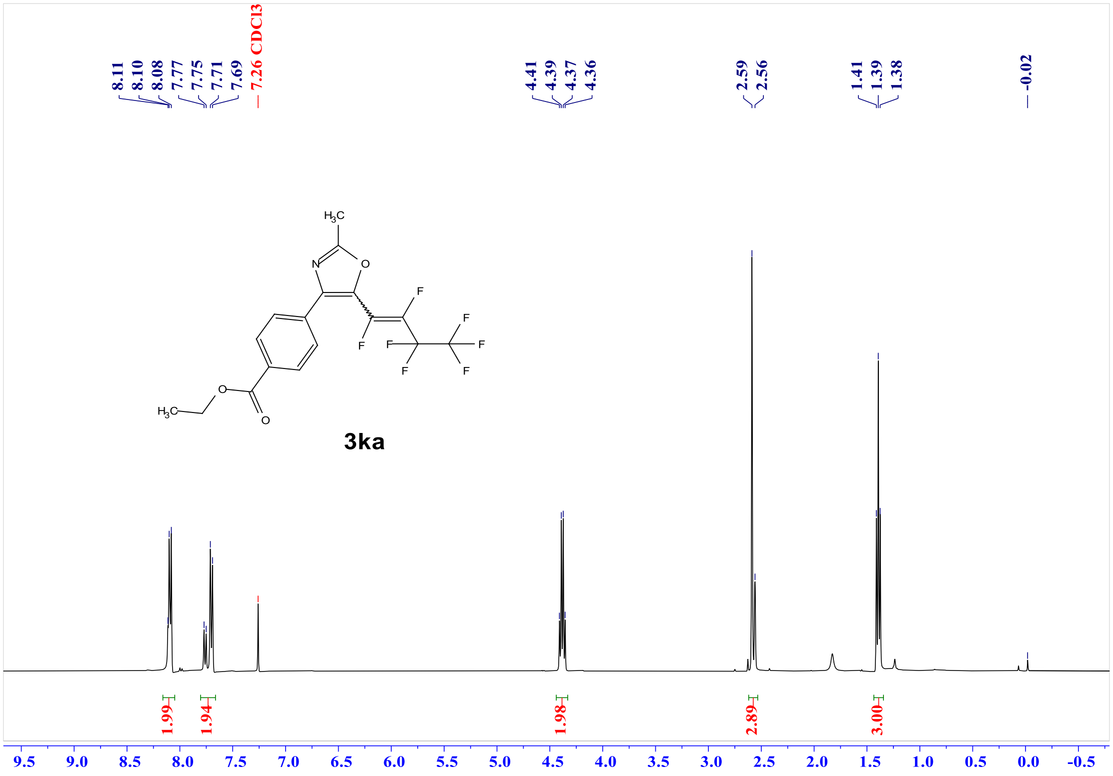

^{19}F NMR spectra of the product **3ka** (376 MHz, $CDCl_3$)

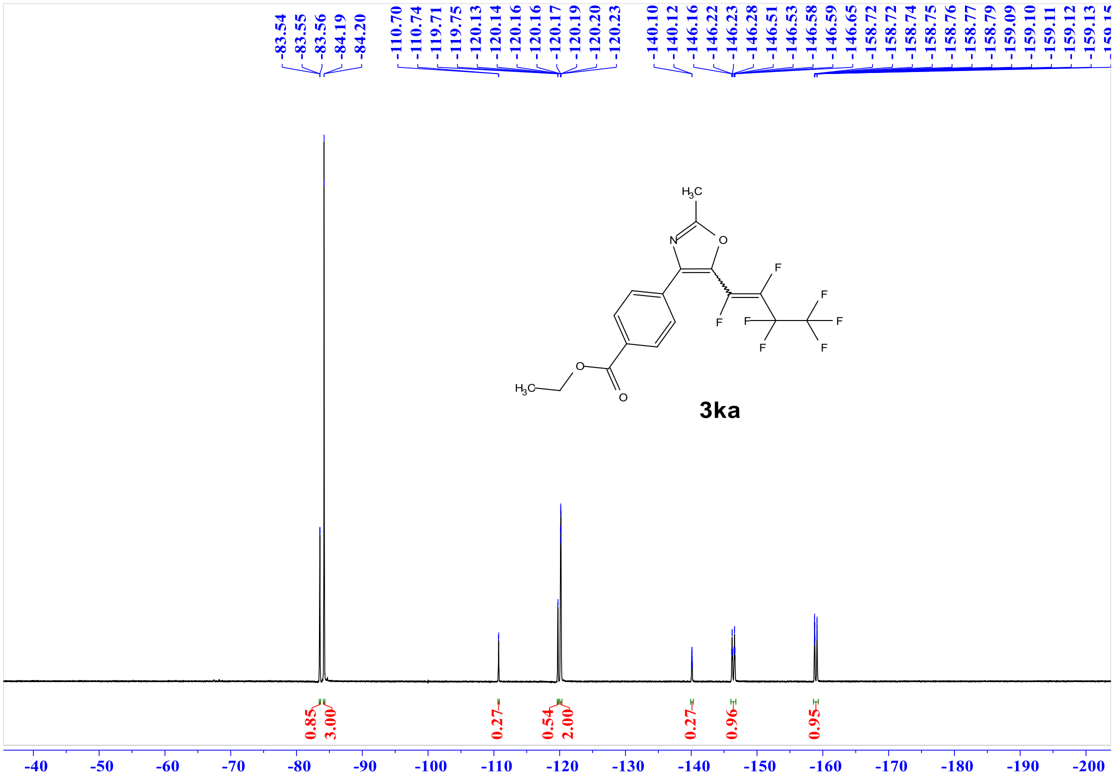

^{13}C NMR spectra of the product **3ka** (100 MHz, $CDCl_3$)

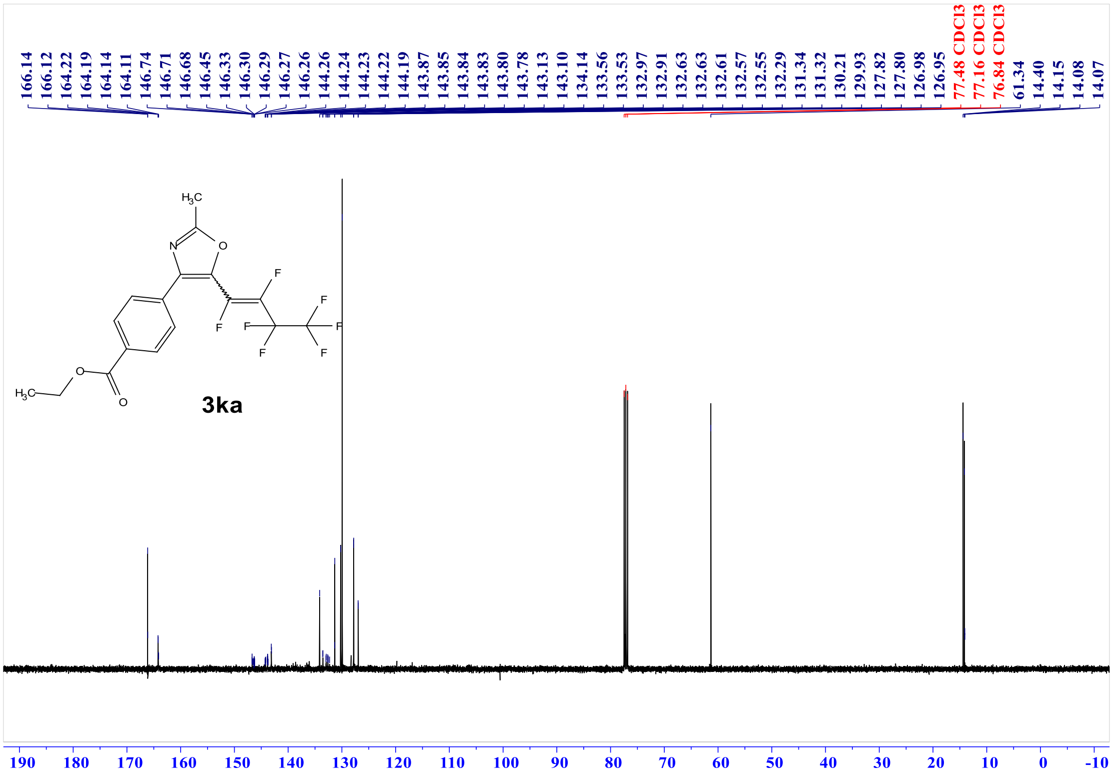

^{1}H NMR spectra of the product **3la** (400 MHz, $CDCl_3$)

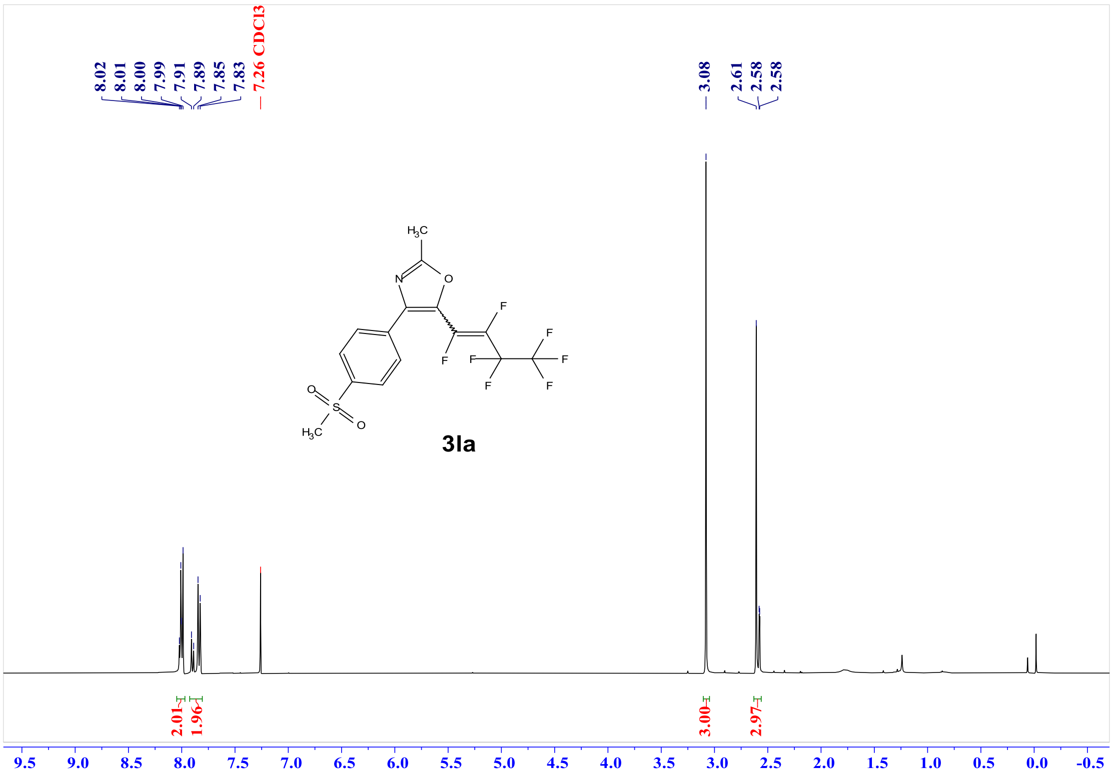

^{19}F NMR spectra of the product **3la** (376 MHz, $CDCl_3$)

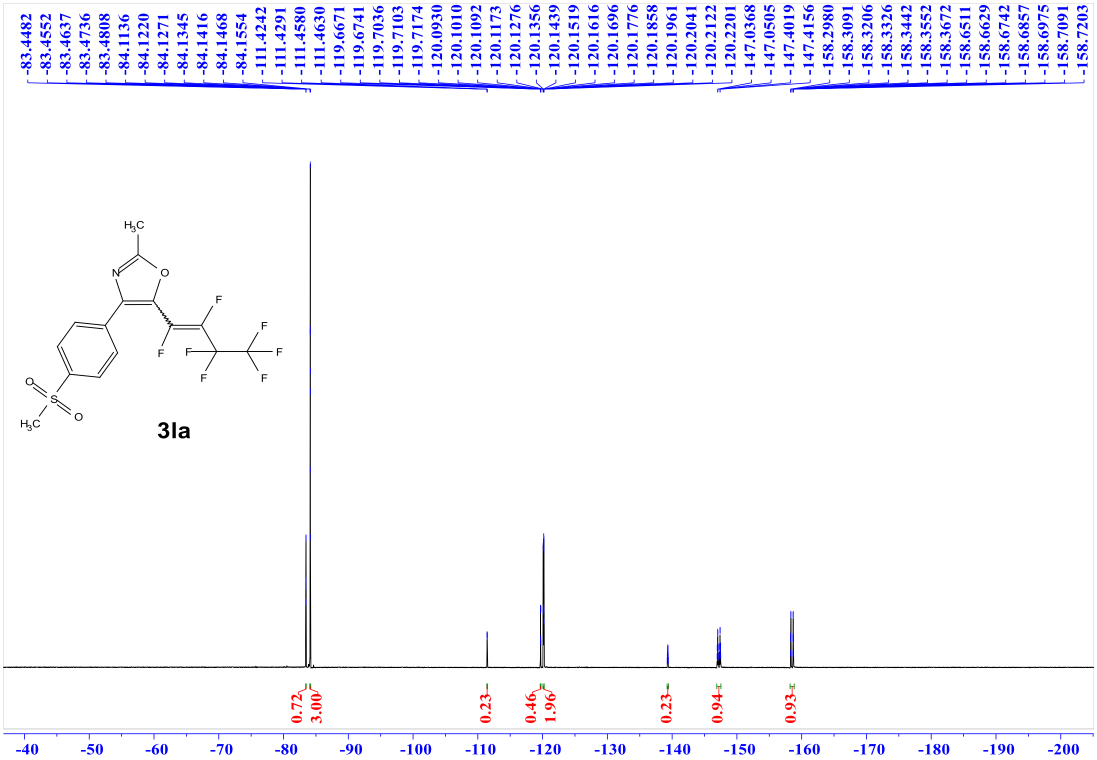

^{13}C NMR spectra of the product **3la** (100 MHz, $CDCl_3$)

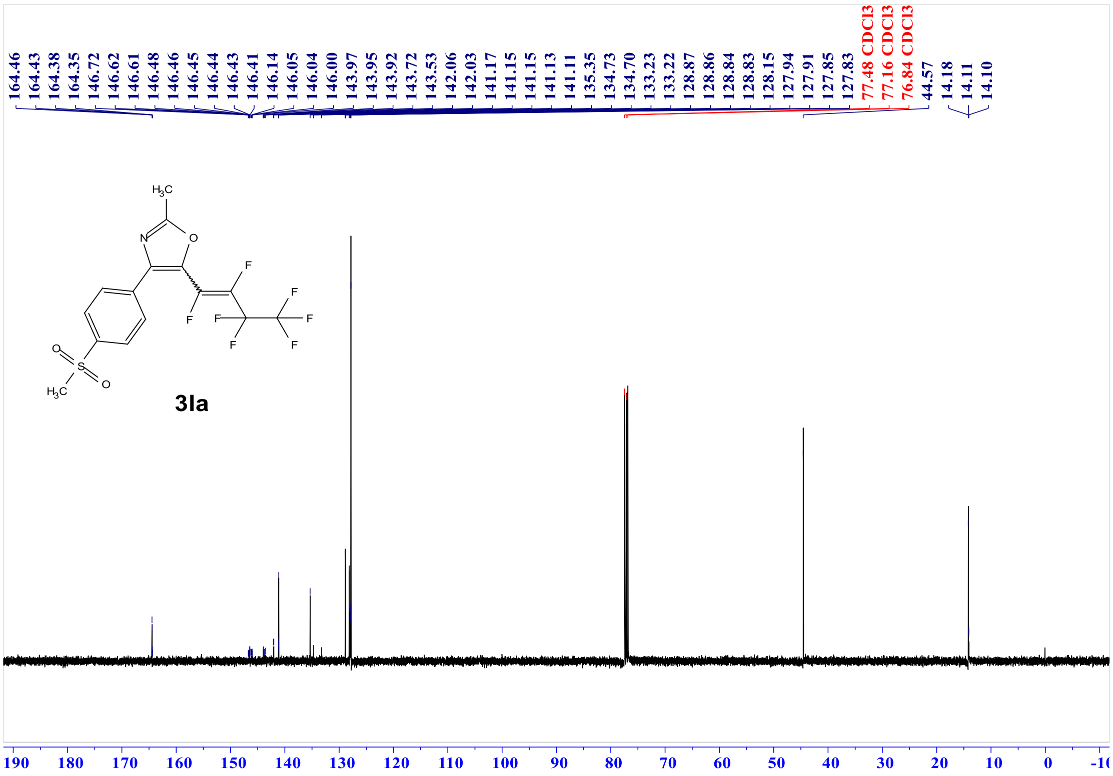

^{1}H NMR spectra of the product **3ma** (400 MHz, $CDCl_3$)

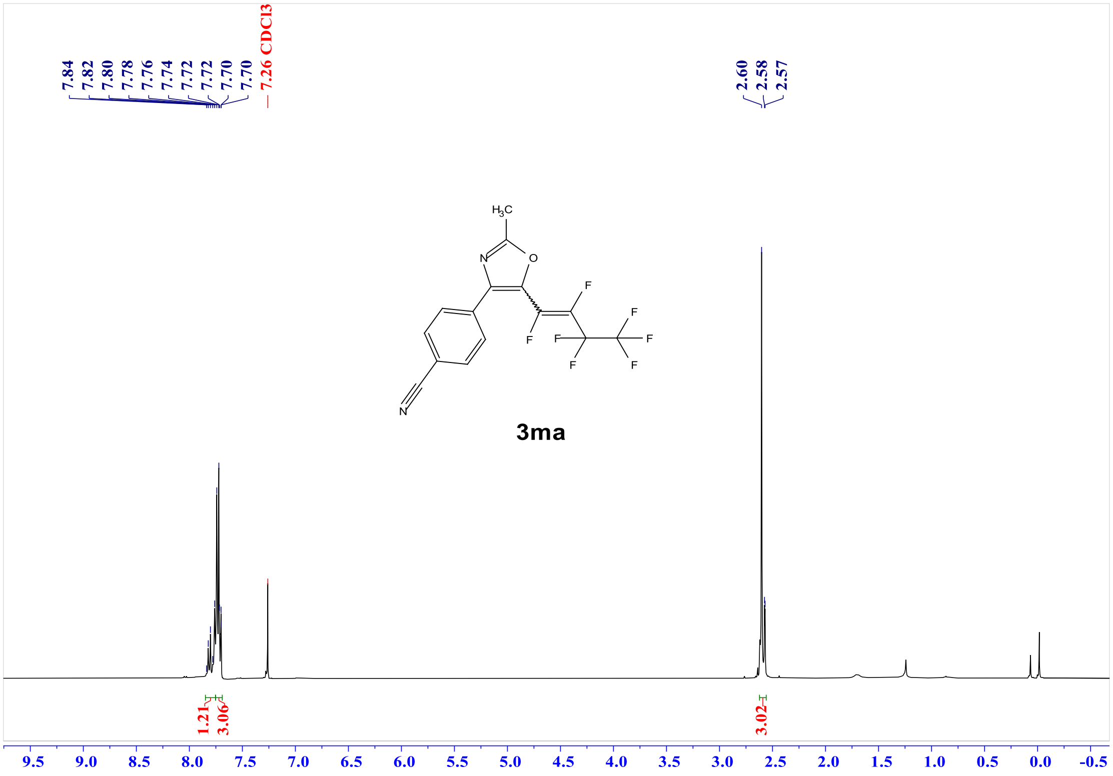

^{19}F NMR spectra of the product **3ma** (376 MHz, $CDCl_3$)

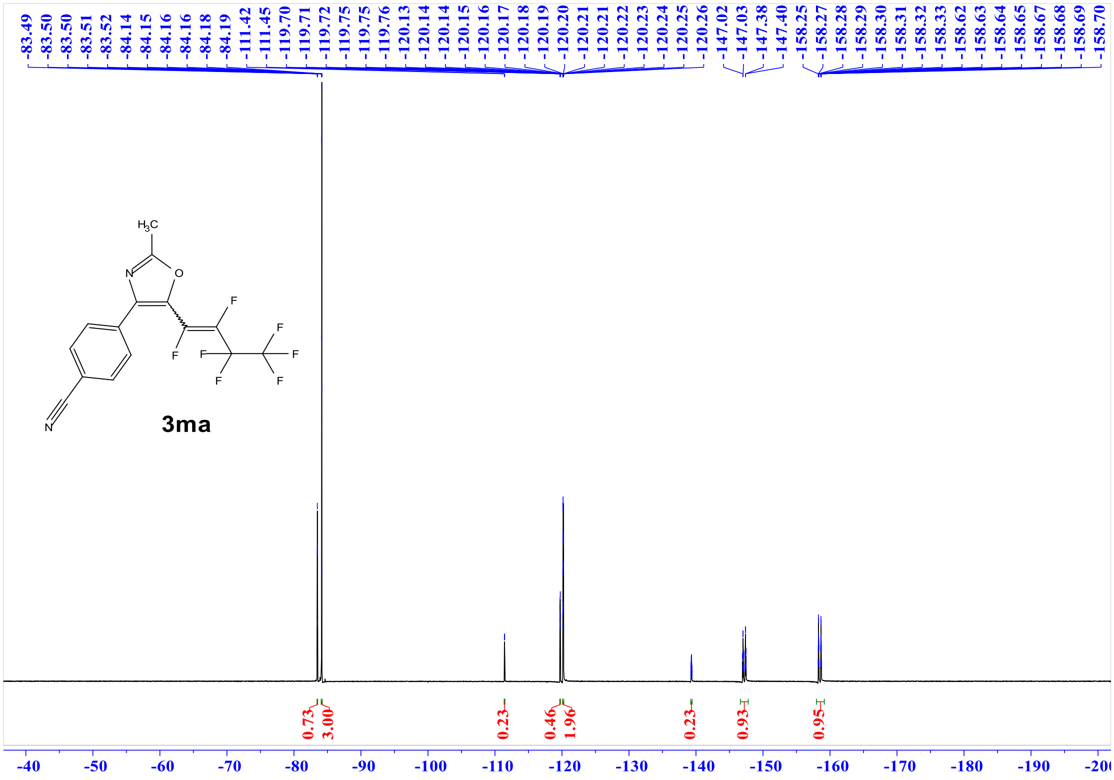

^{13}C NMR spectra of the product **3ma** (100 MHz, $CDCl_3$)

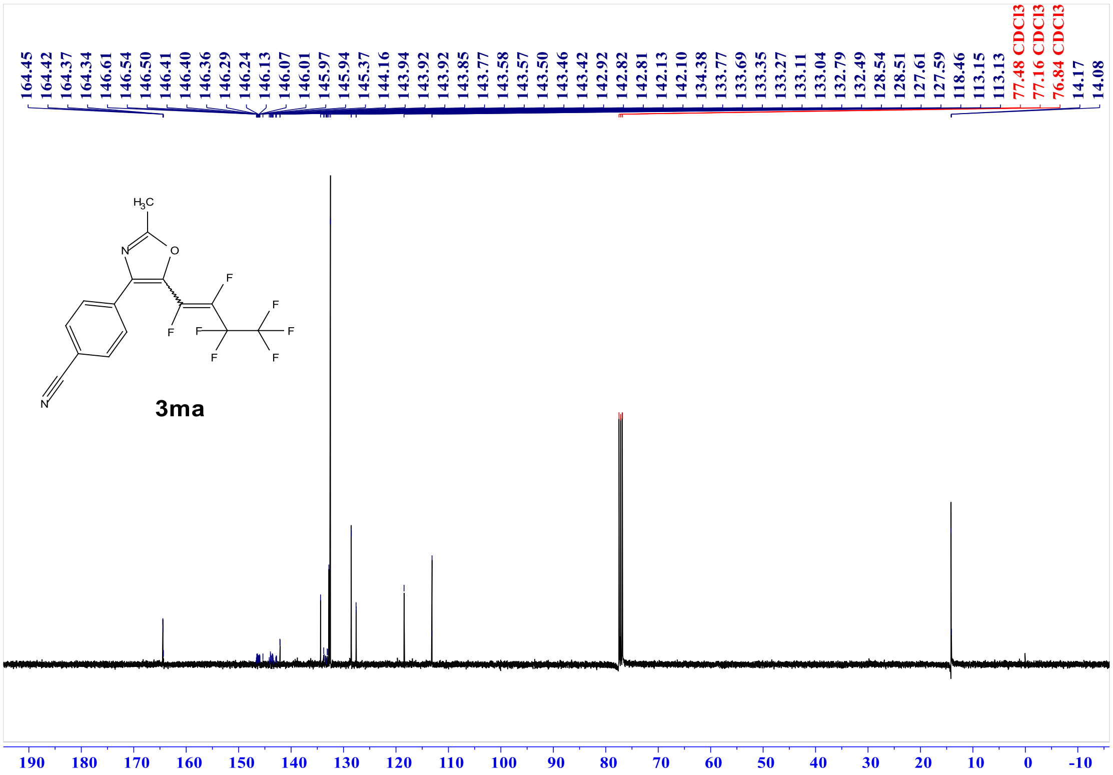

^{1}H NMR spectra of the product **3na** (400 MHz, $CDCl_3$)

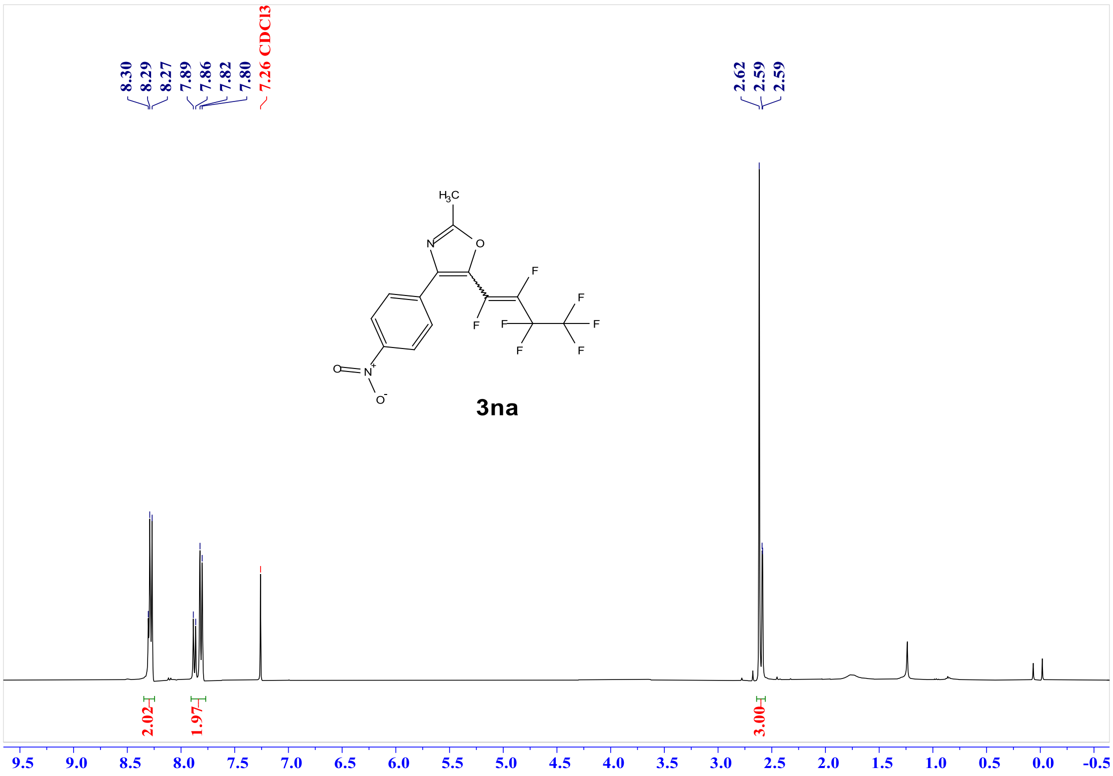

^{19}F NMR spectra of the product **3na** (376 MHz, $CDCl_3$)

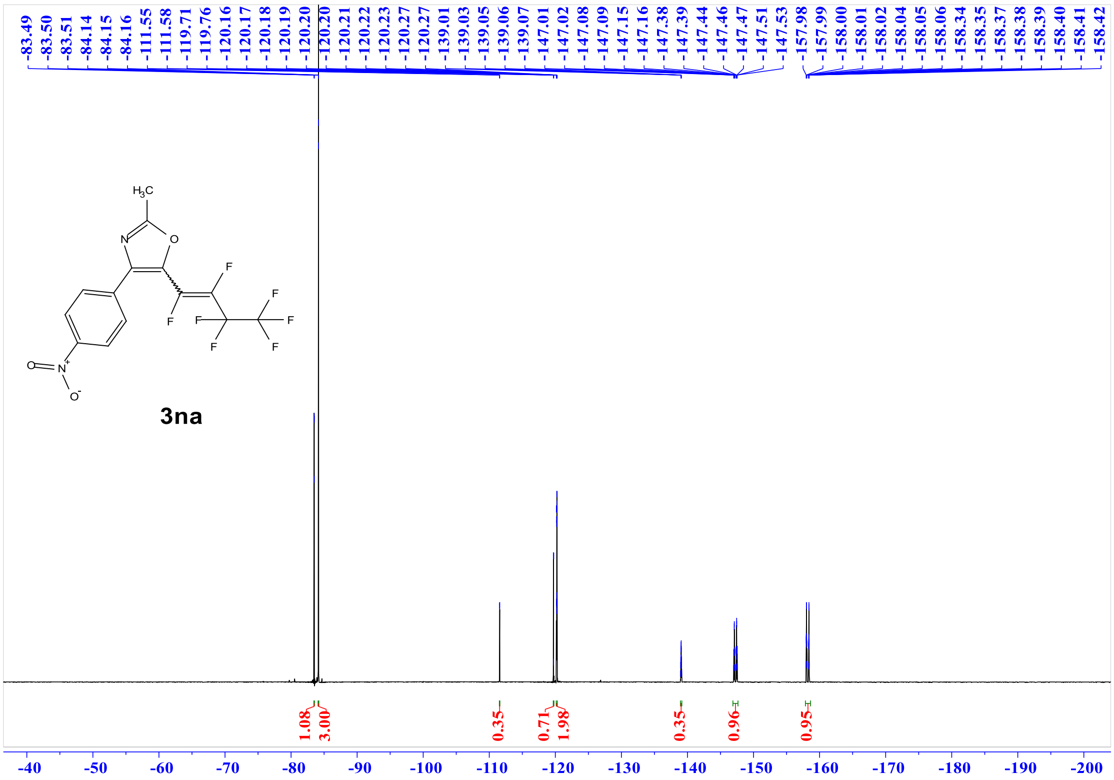

^{13}C NMR spectra of the product **3na** (100 MHz, $CDCl_3$)

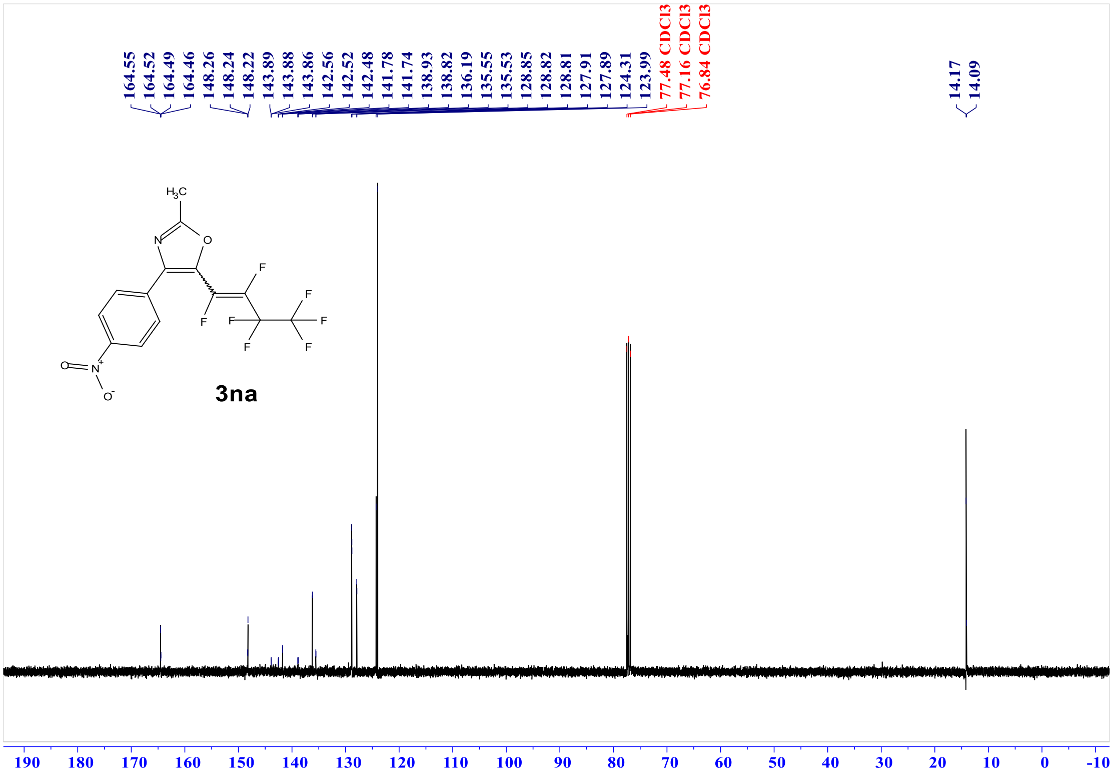

^{1}H NMR spectra of the product **3oa** (400 MHz, CDCl3)

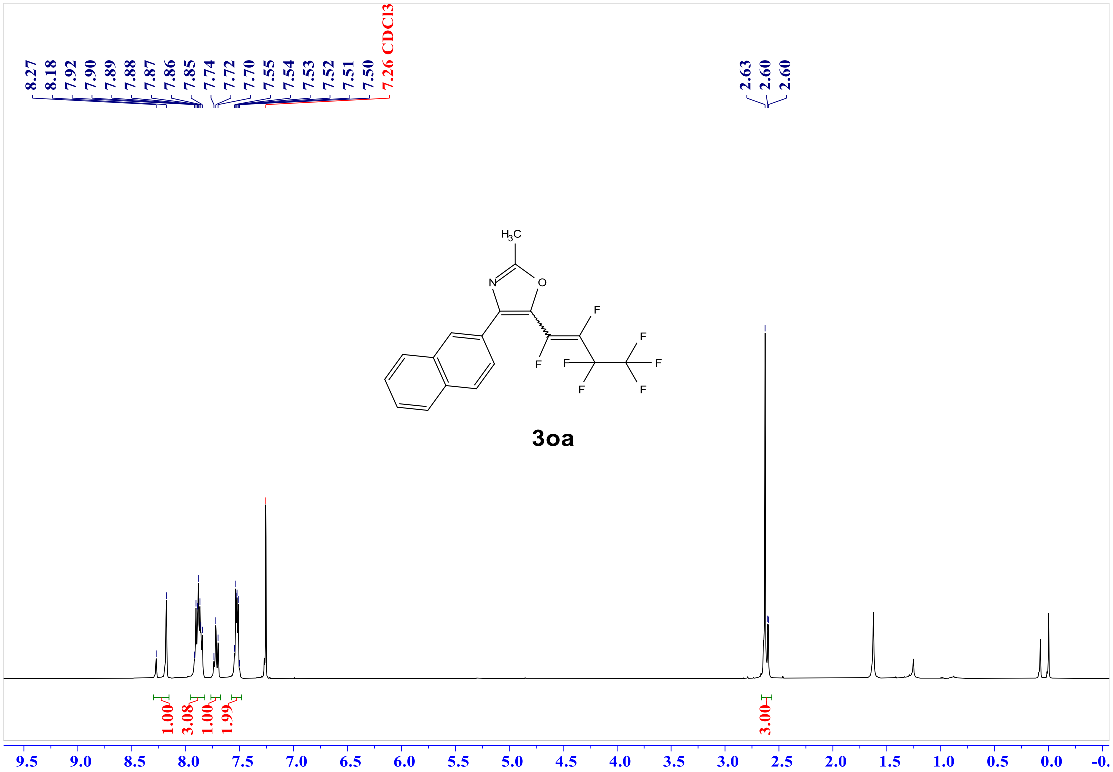

^{19}F NMR spectra of the product **3oa** (376 MHz, CDCl3)

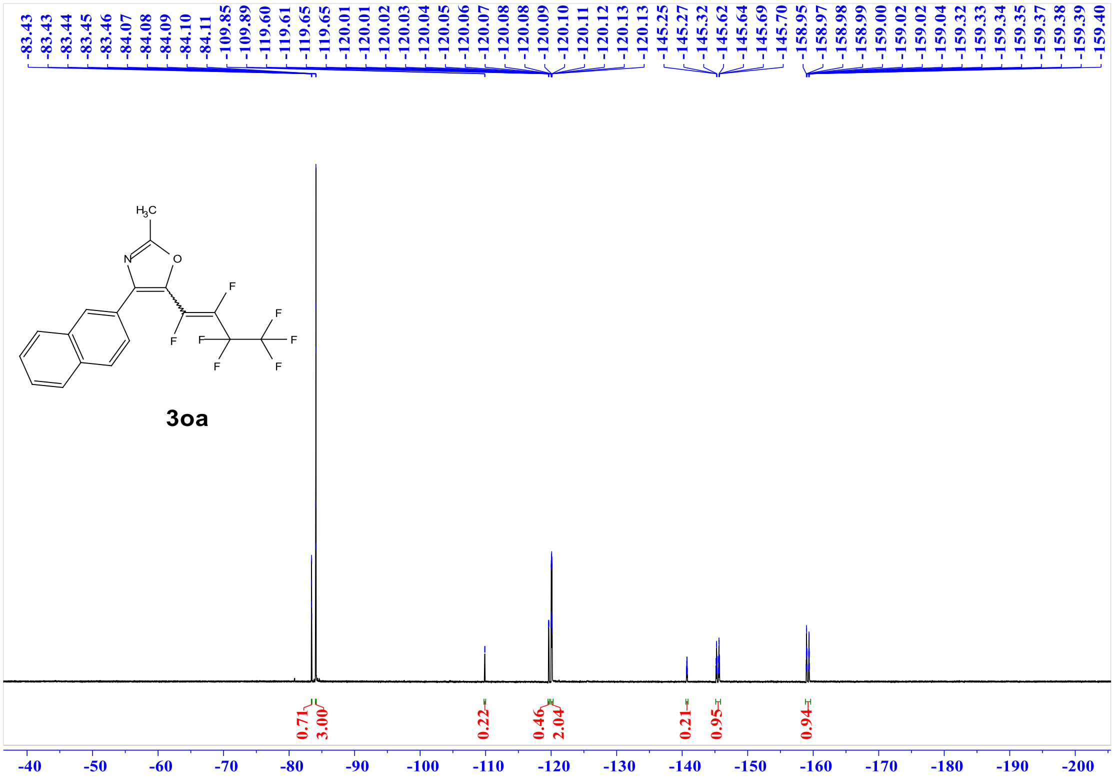

^{13}C NMR spectra of the product **3oa** (100 MHz, $CDCl_3$)

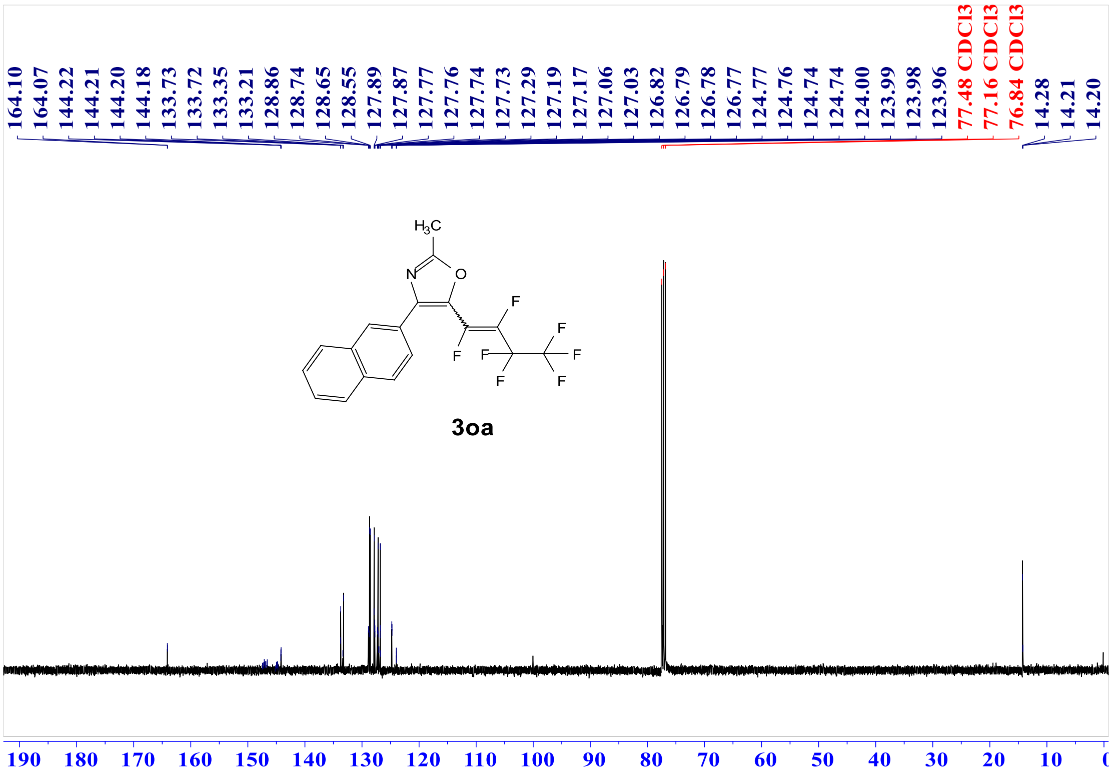

^{1}H NMR spectra of the product **3pa** (400 MHz, $CDCl_3$)

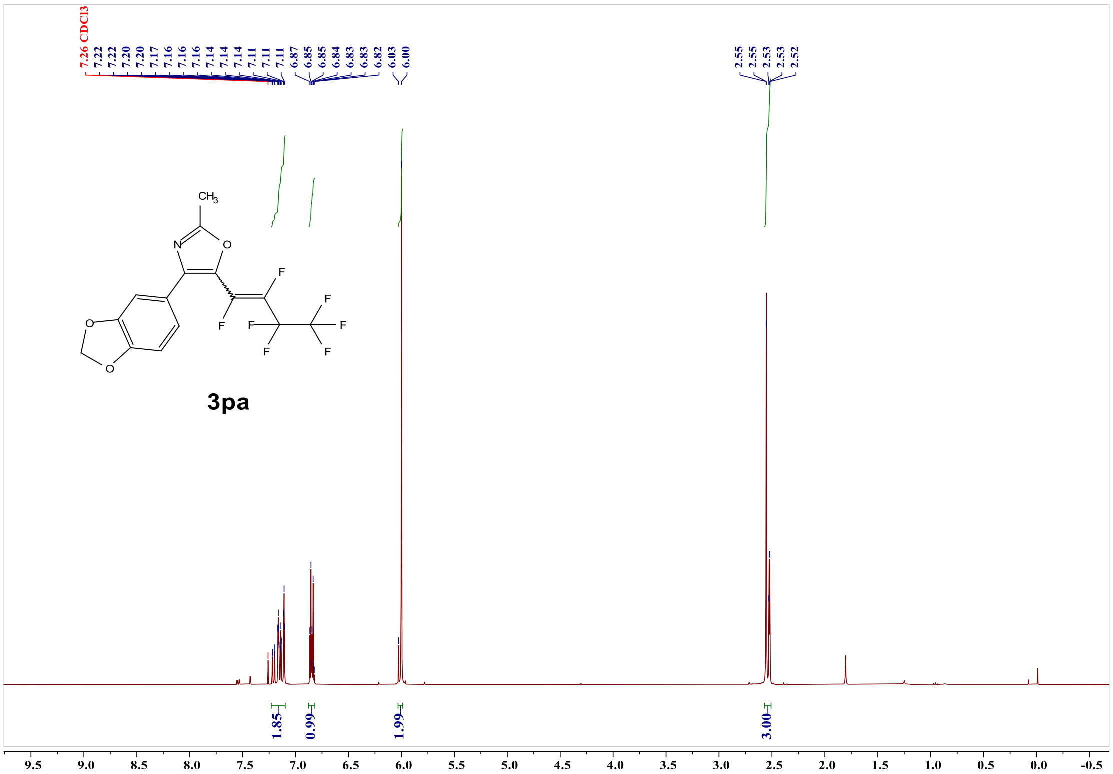

^{19}F NMR spectra of the product **3pa** (376 MHz, $CDCl_3$)

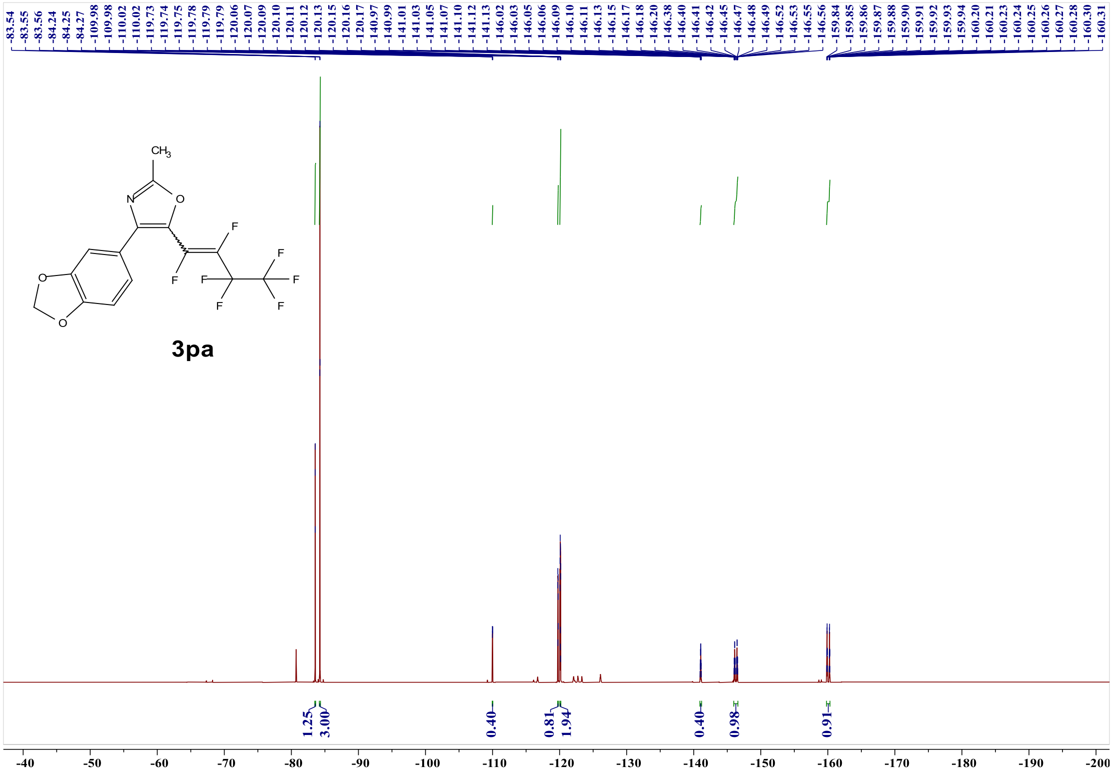

^{13}C NMR spectra of the product **3pa** (100 MHz, $CDCl_3$)

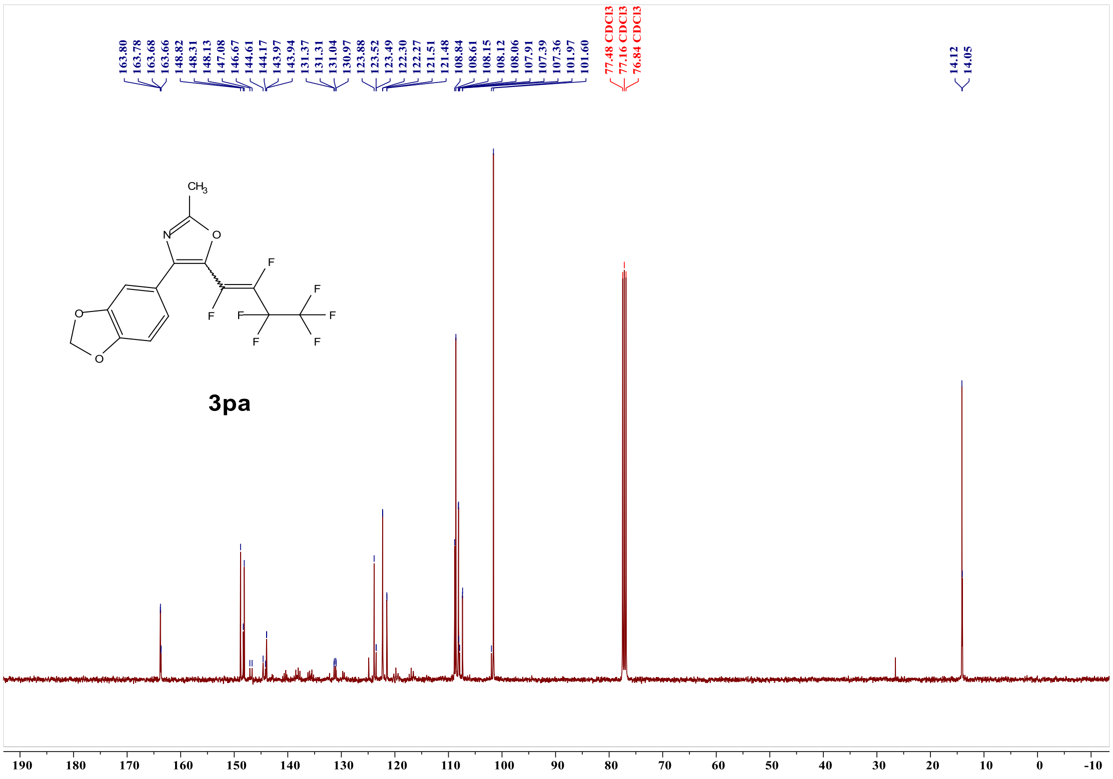

^{1}H NMR spectra of the product **3qa** (400 MHz, $CDCl_3$)

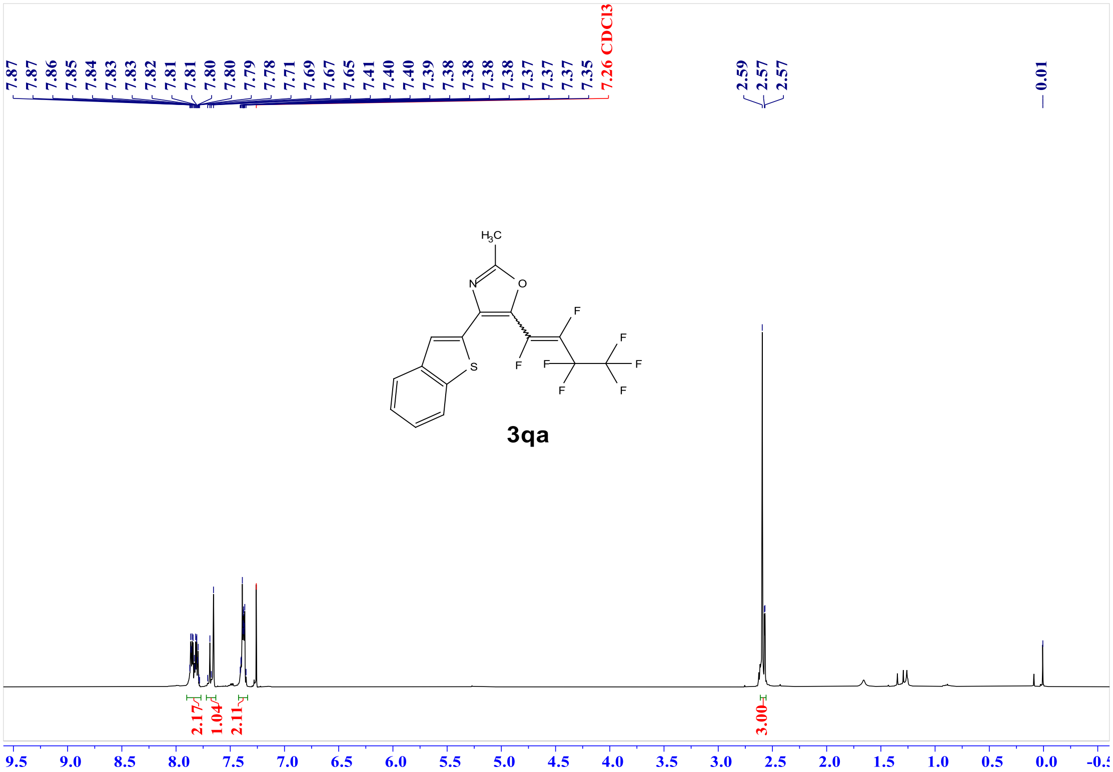

^{19}F NMR spectra of the product **3qa** (376 MHz, $CDCl_3$)

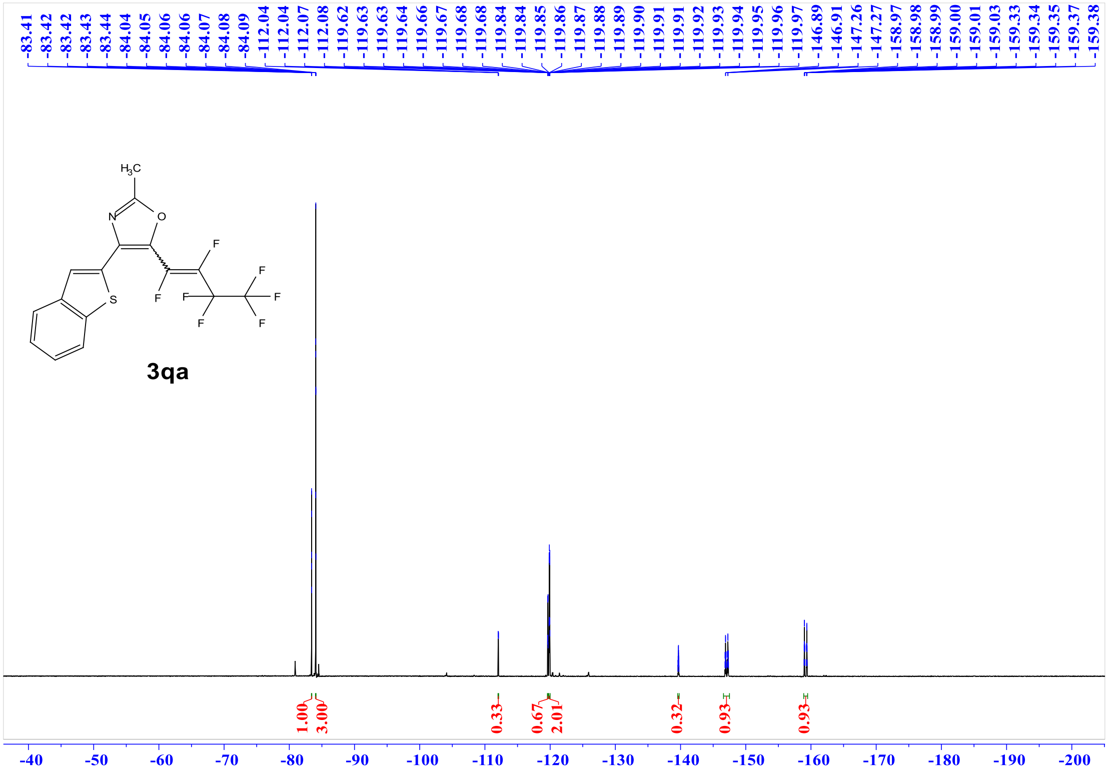

^{13}C NMR spectra of the product **3qa** (100 MHz, $CDCl_3$)

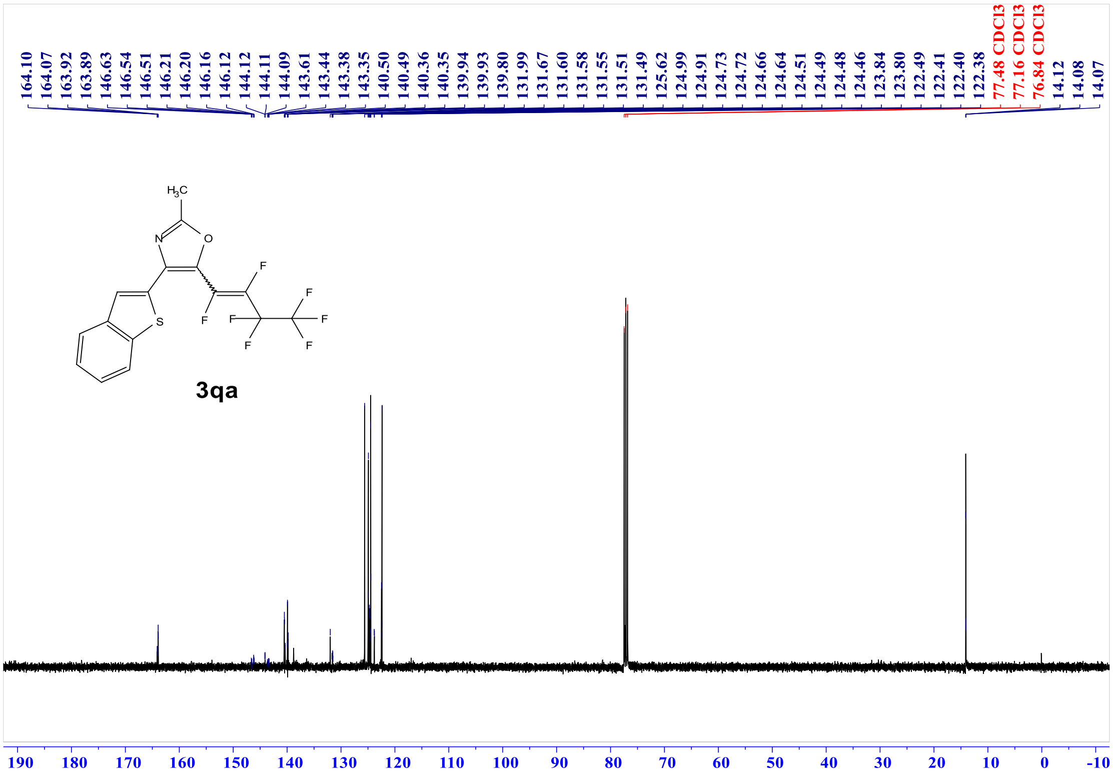

^{1}H NMR spectra of the product **3ra** (400 MHz, $CDCl_3$)

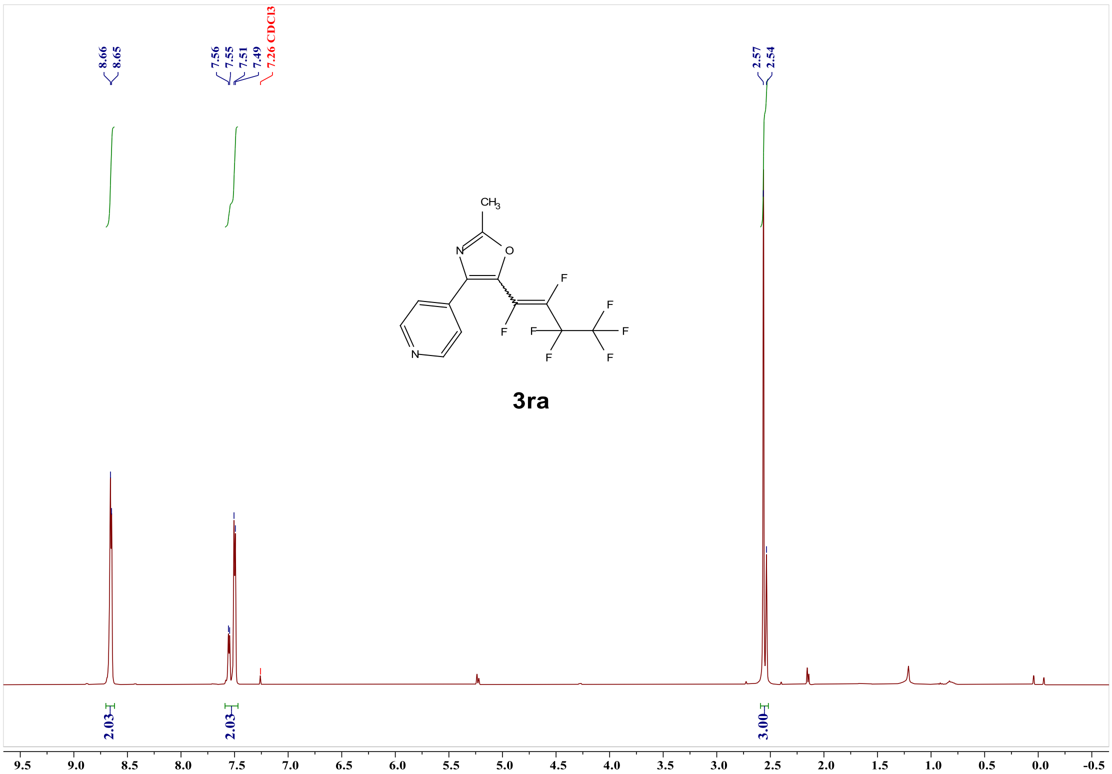

^{19}F NMR spectra of the product **3ra** (376 MHz, $CDCl_3$)

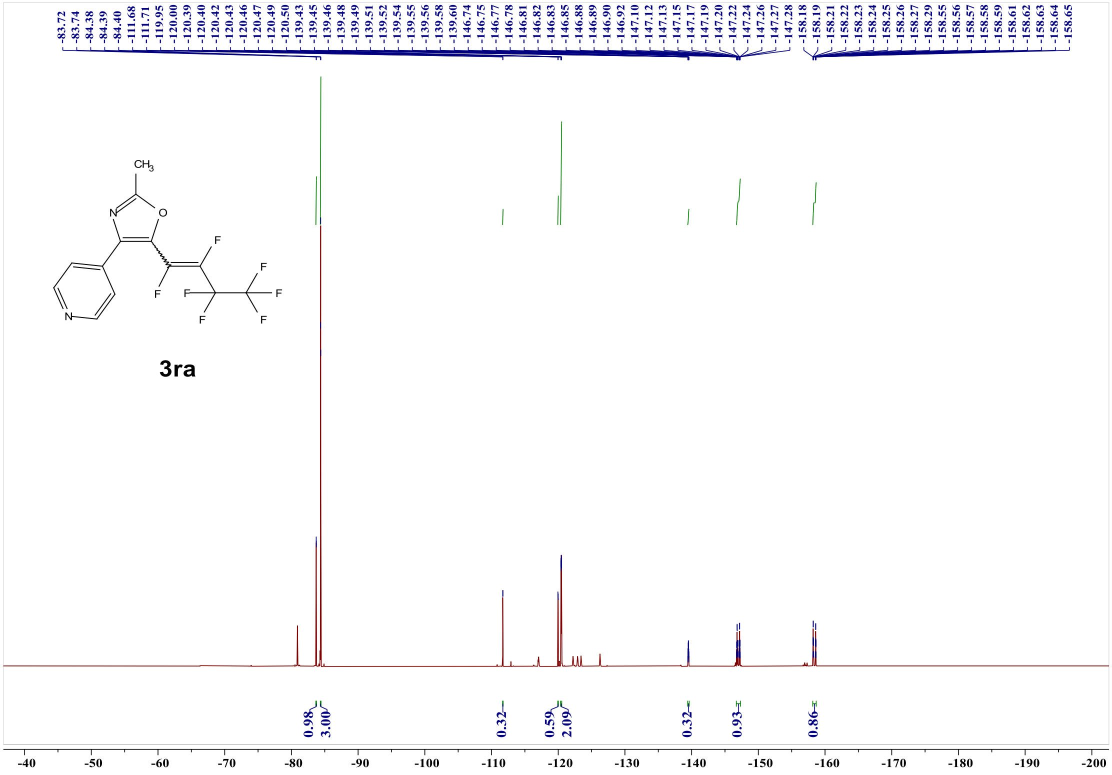

^{13}C NMR spectra of the product **3ra** (100 MHz, $CDCl_3$)

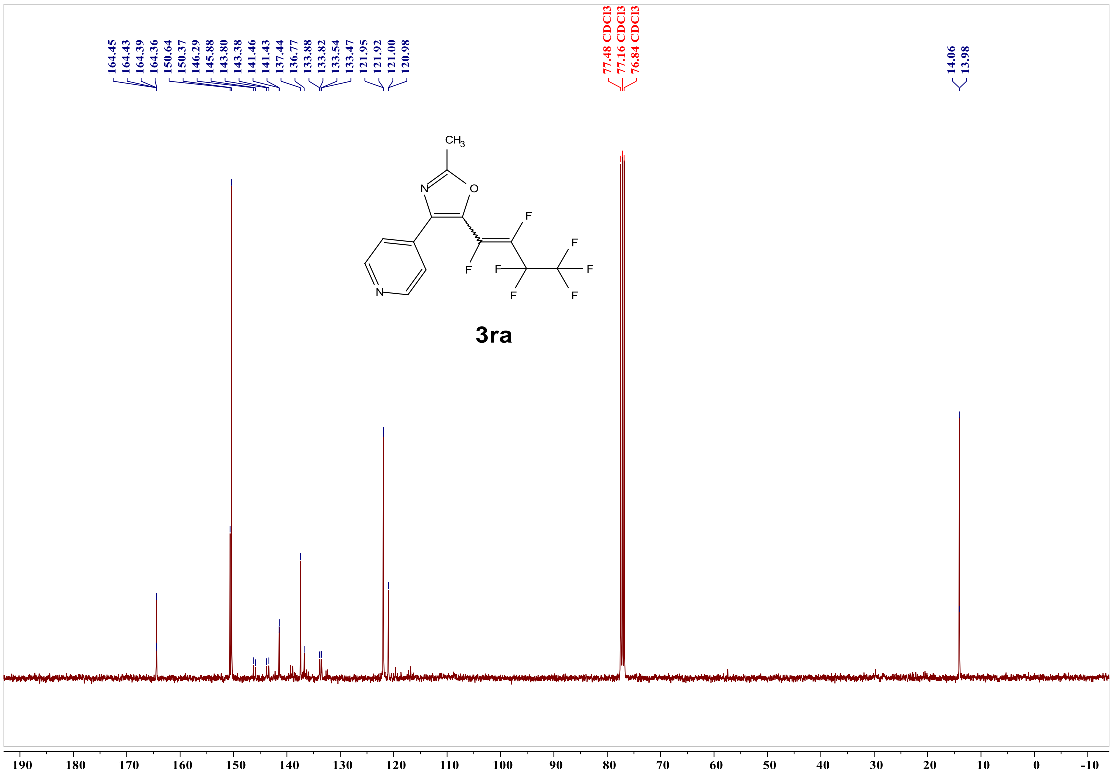

^{1}H NMR spectra of the product **3sa** (400 MHz, $CDCl_3$)

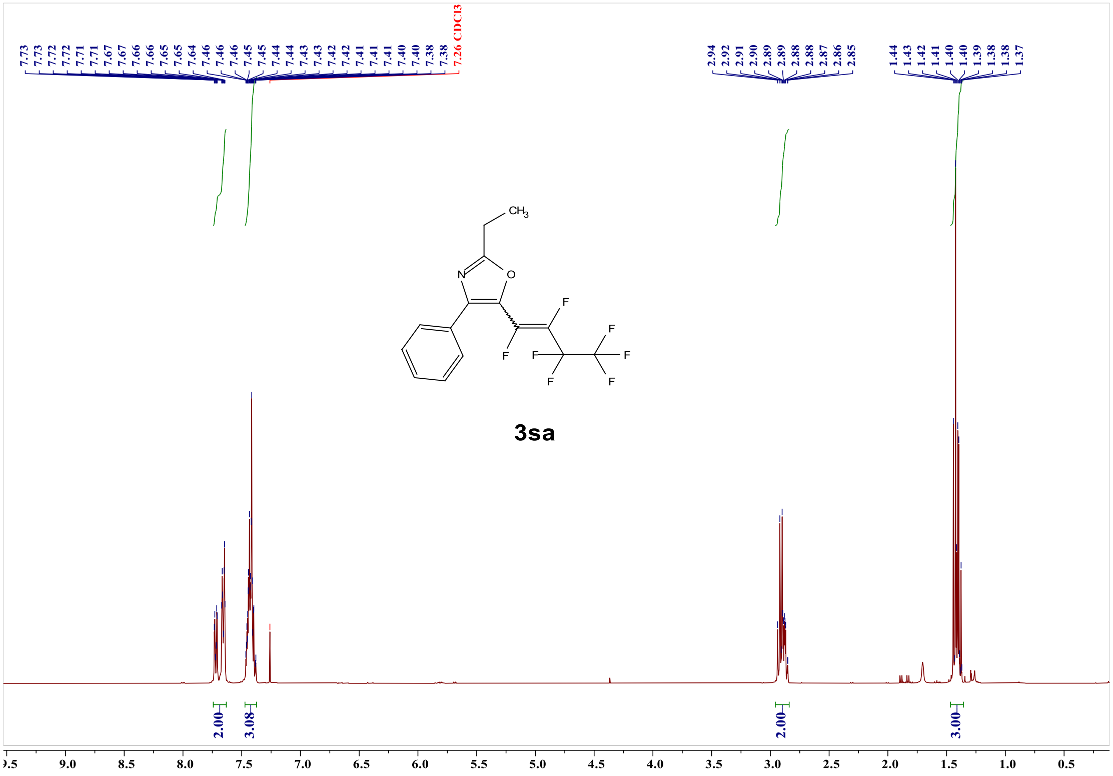

^{19}F NMR spectra of the product **3sa** (376 MHz, $CDCl_3$)

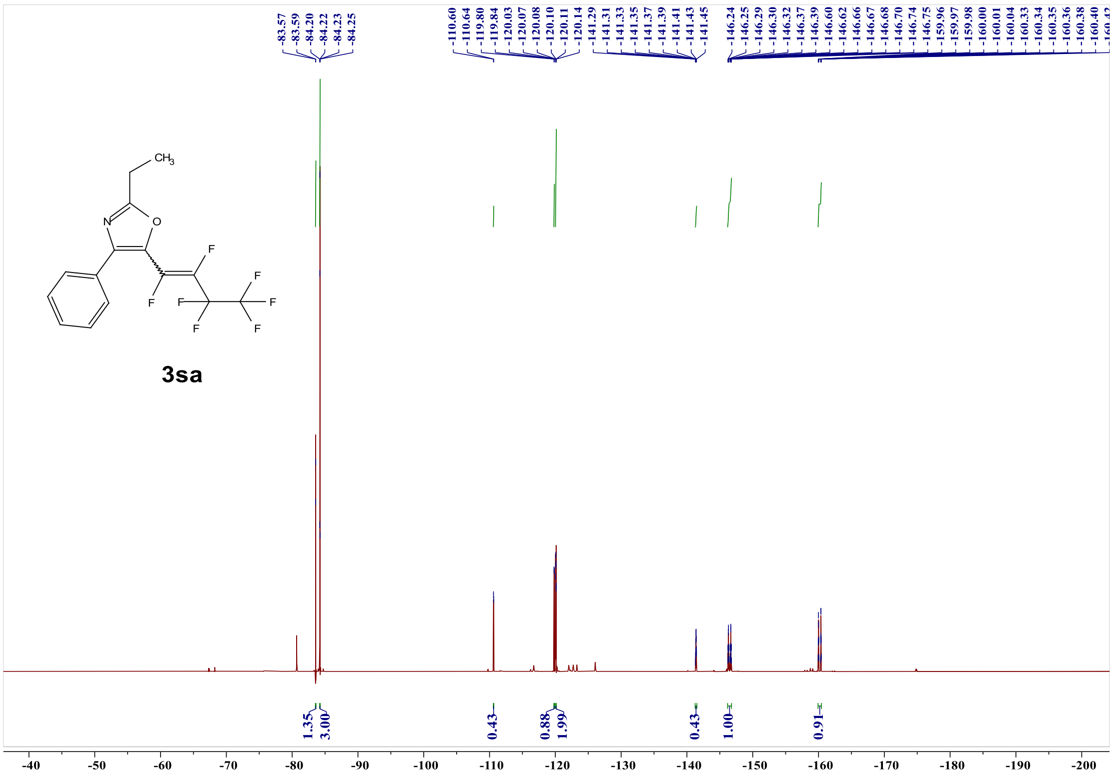

^{13}C NMR spectra of the product **3sa** (100 MHz, $CDCl_3$)

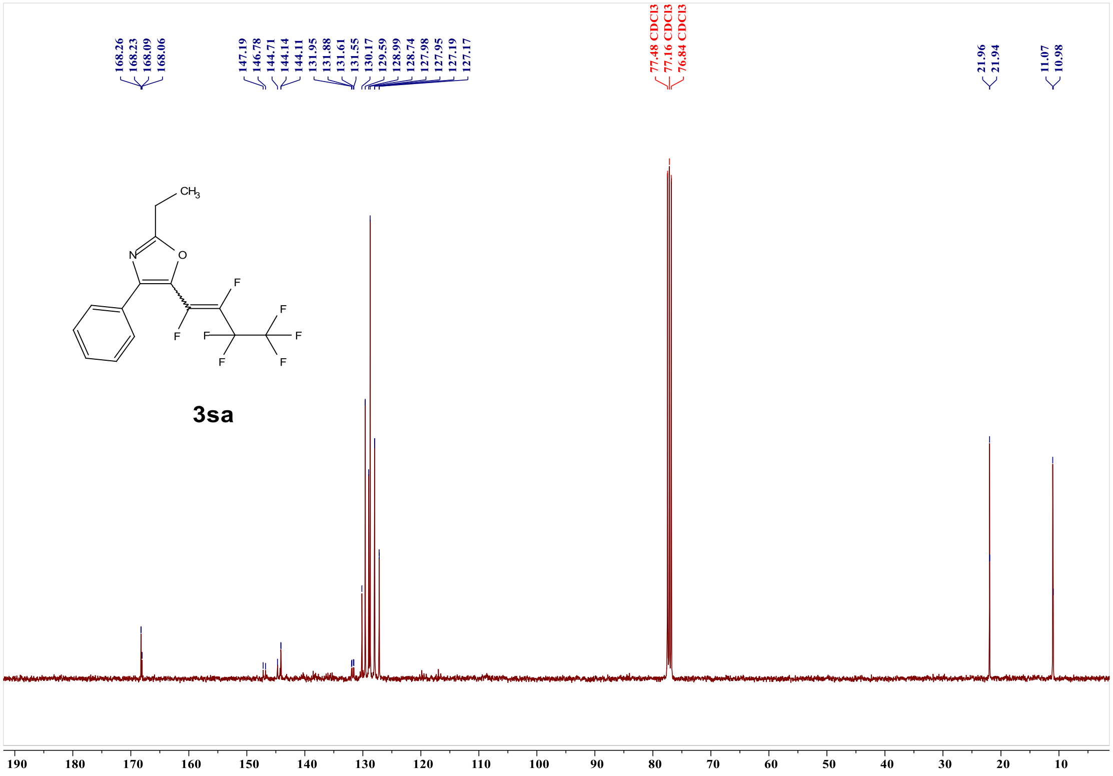

^{1}H NMR spectra of the product **3ta** (400 MHz, $CDCl_3$)

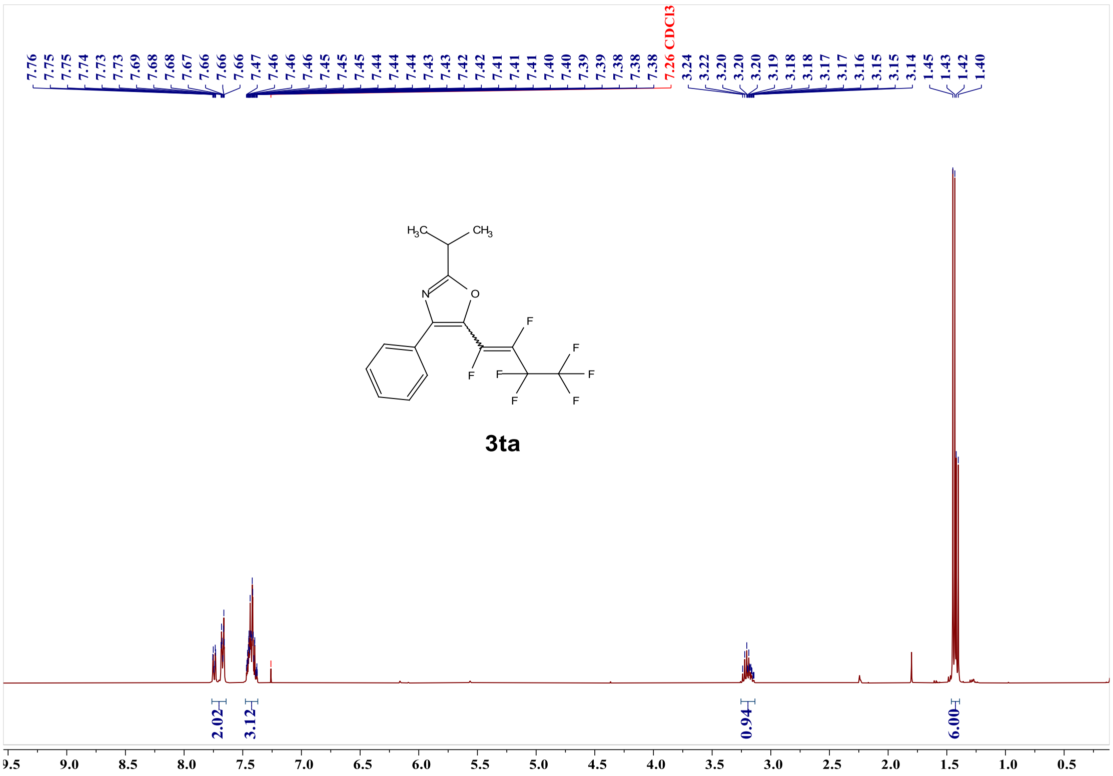

^{19}F NMR spectra of the product **3ta** (376 MHz, $CDCl_3$)

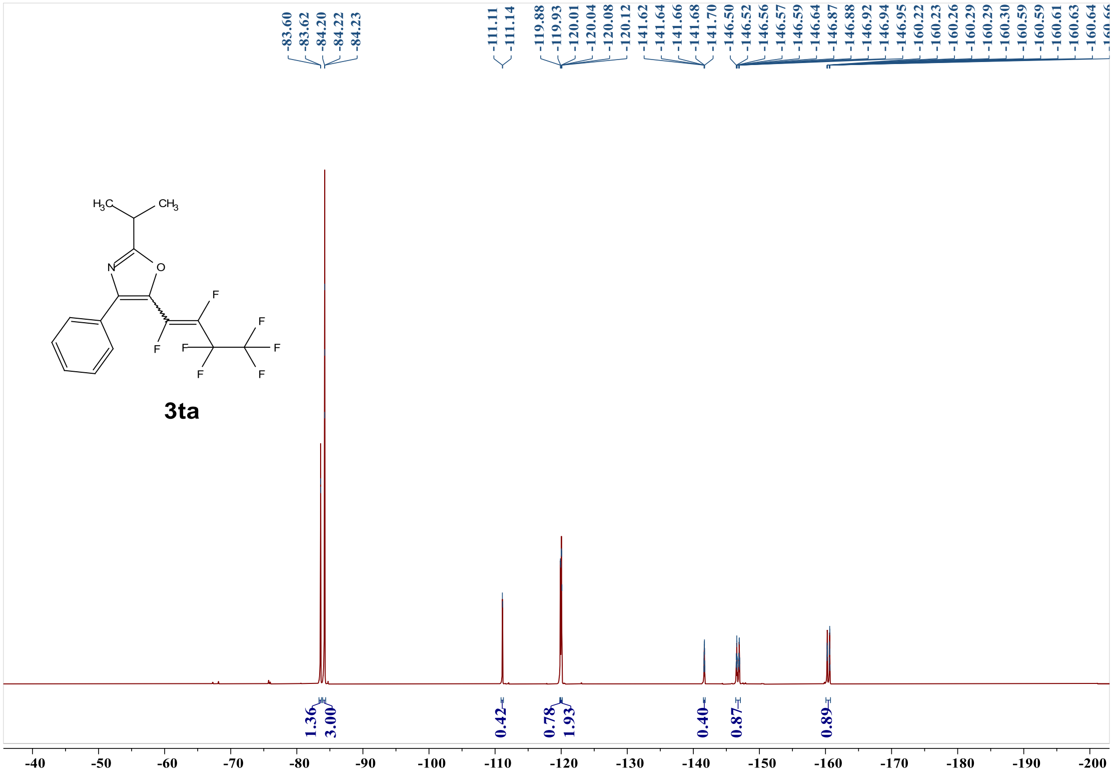

^{13}C NMR spectra of the product **3ta** (100 MHz, $CDCl_3$)

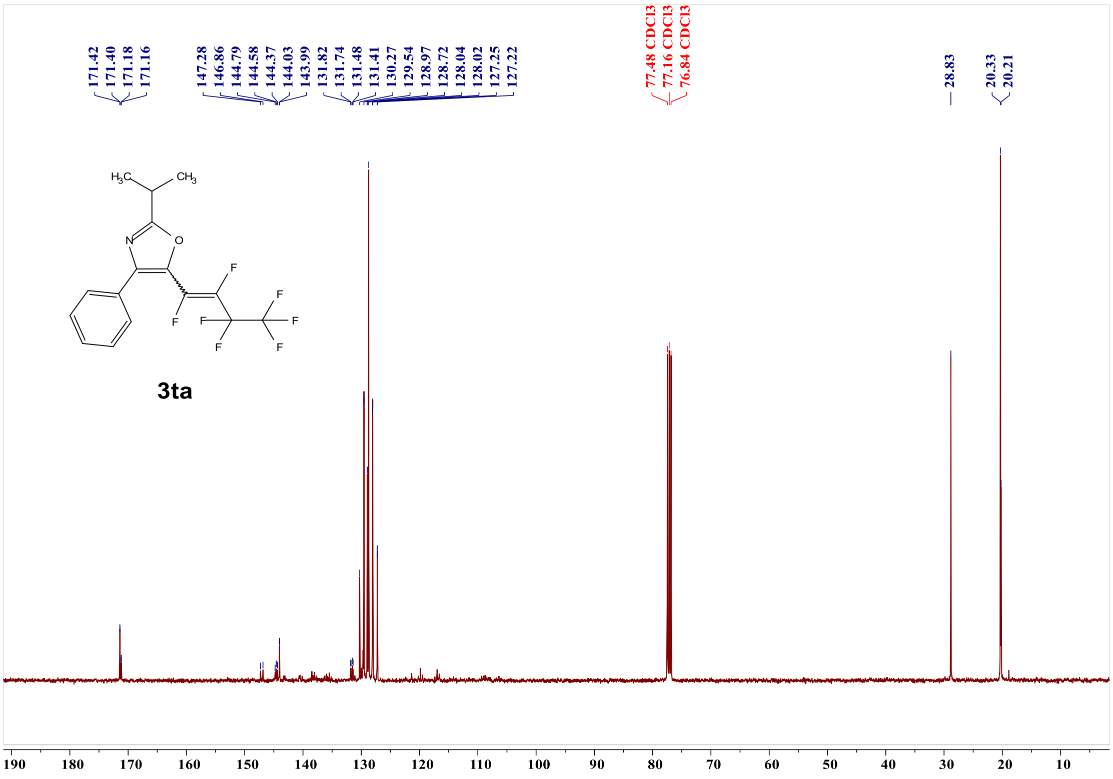

^{1}H NMR spectra of the product **3ua** (400 MHz, $CDCl_3$)

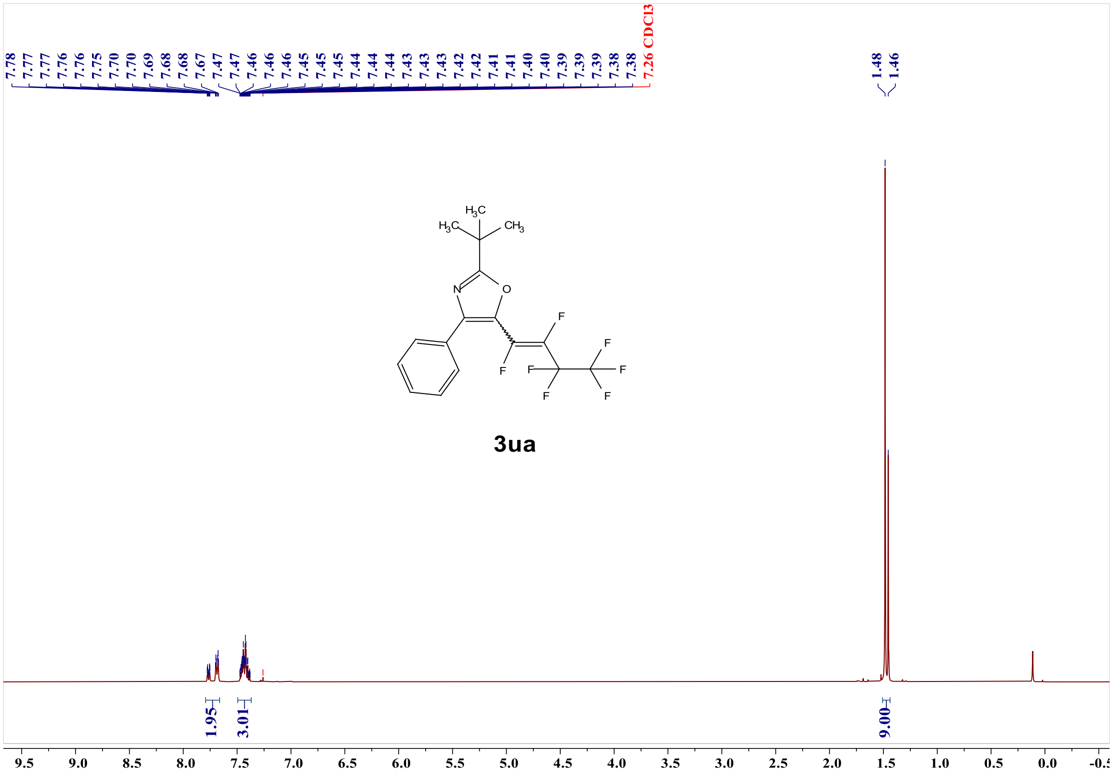

^{19}F NMR spectra of the product **3ua** (376 MHz, $CDCl_3$)

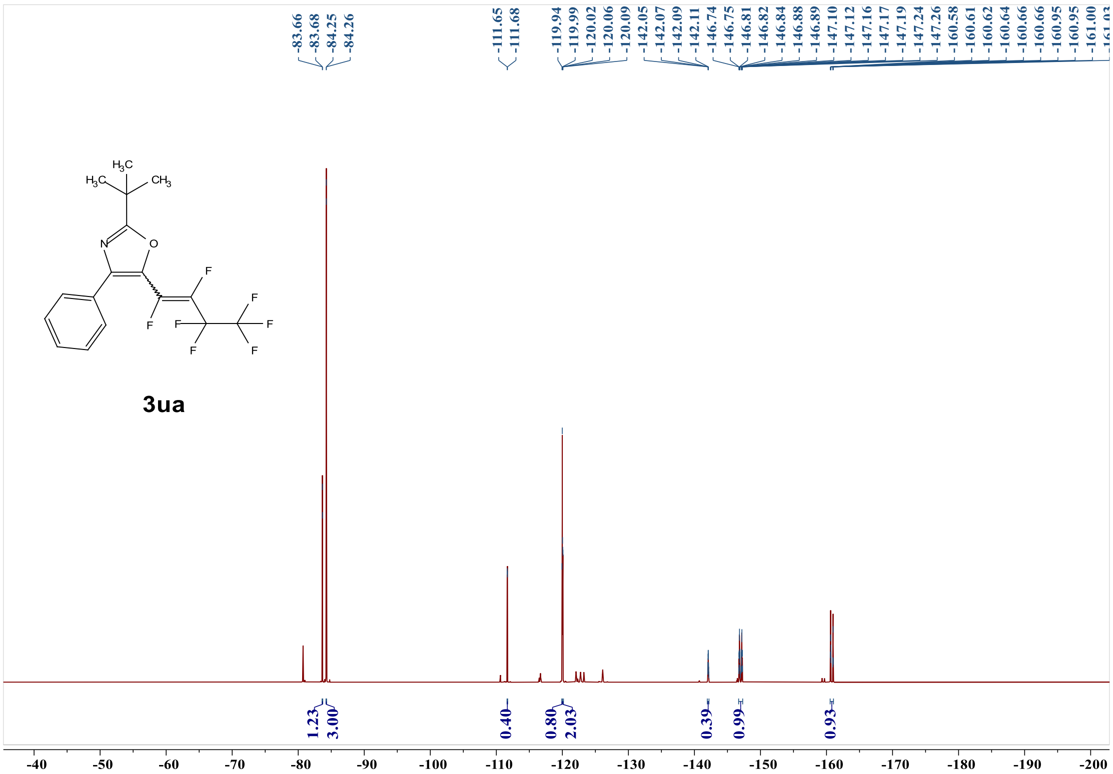

^{13}C NMR spectra of the product **3ua** (100 MHz, $CDCl_3$)

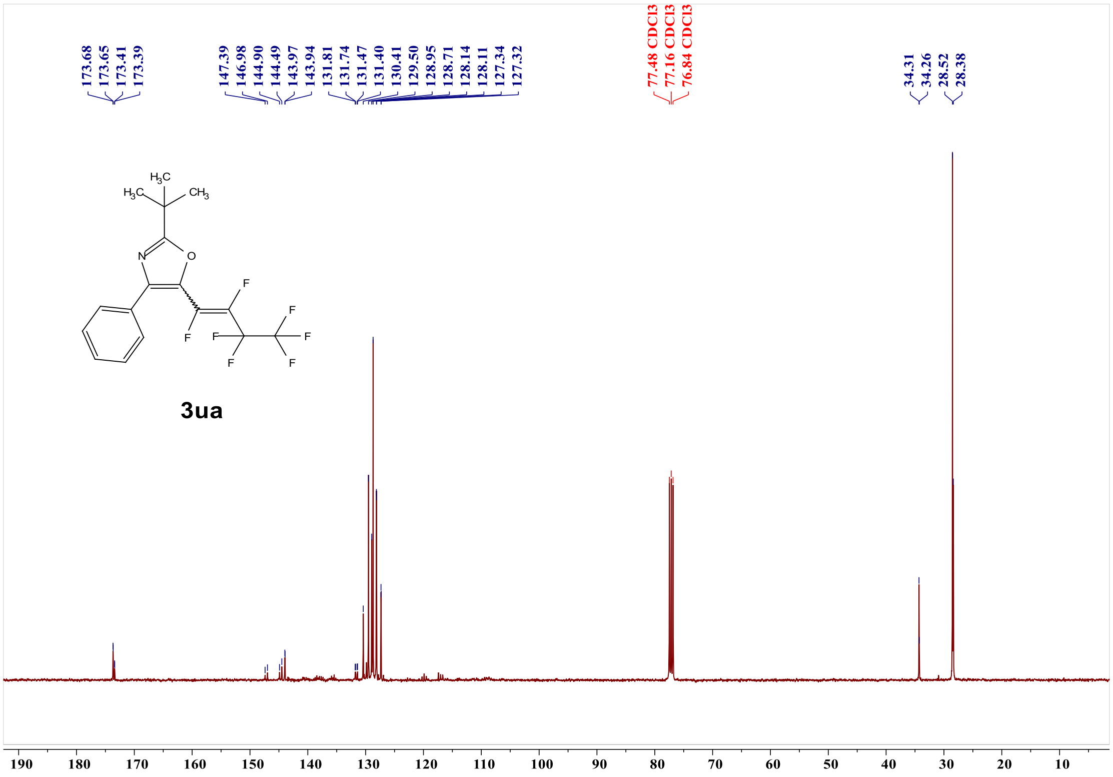

^{1}H NMR spectra of the product **3va** (400 MHz, $CDCl_3$)

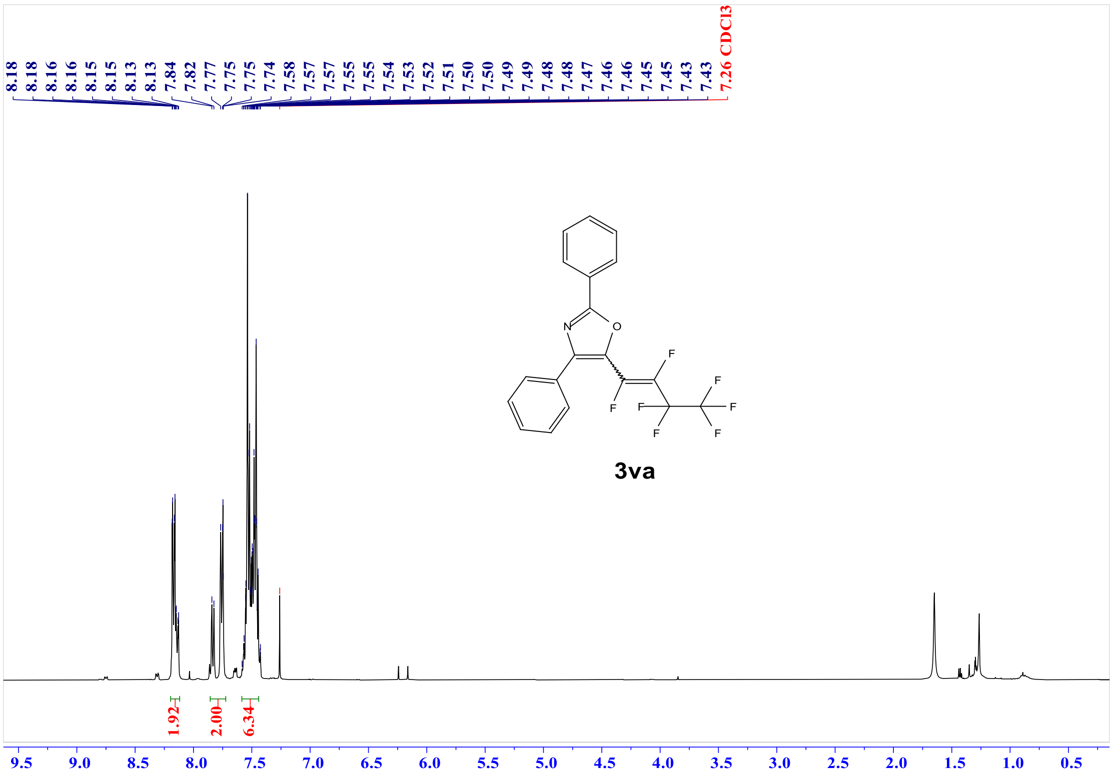

^{19}F NMR spectra of the product **3va** (376 MHz, $CDCl_3$)

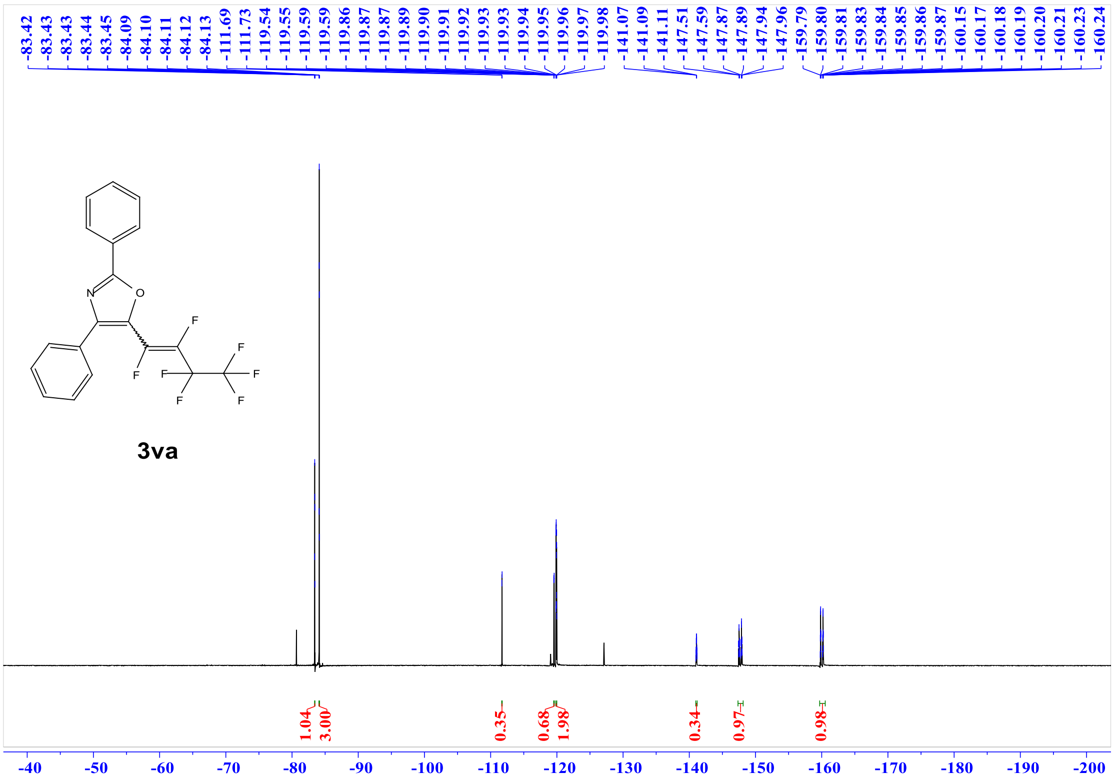

^{13}C NMR spectra of the product **3va** (100 MHz, $CDCl_3$)

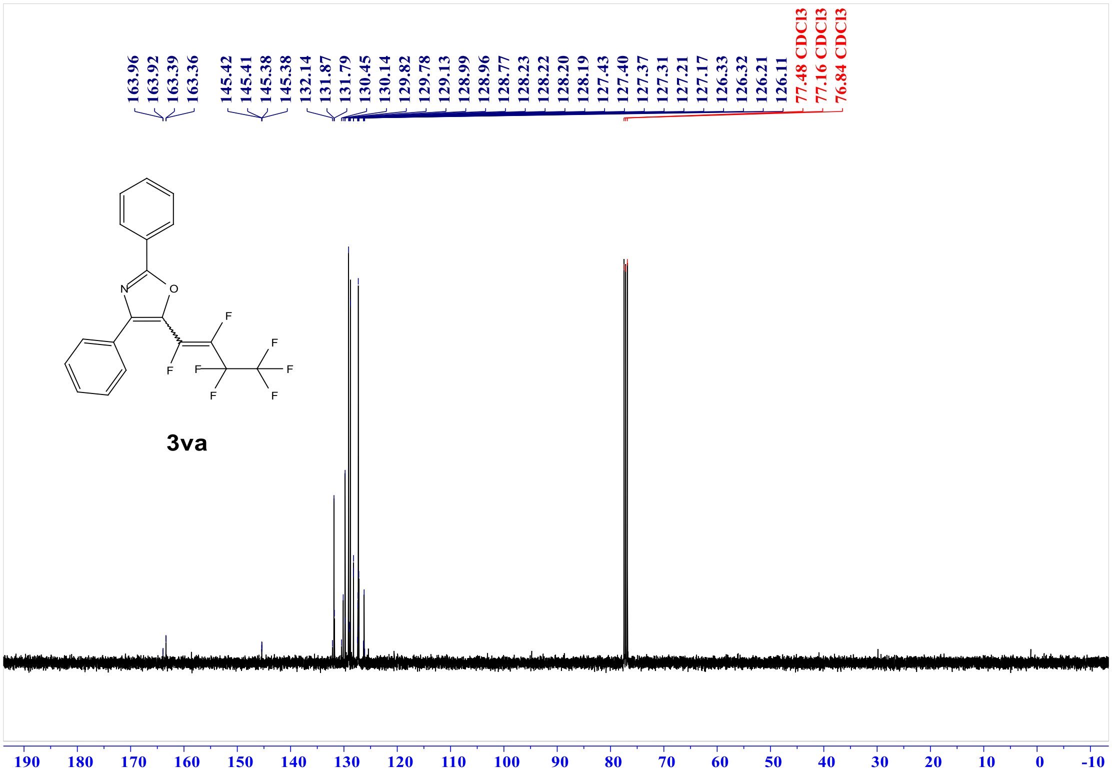

^{1}H NMR spectra of the product **3we** (400 MHz, $CDCl_3$)

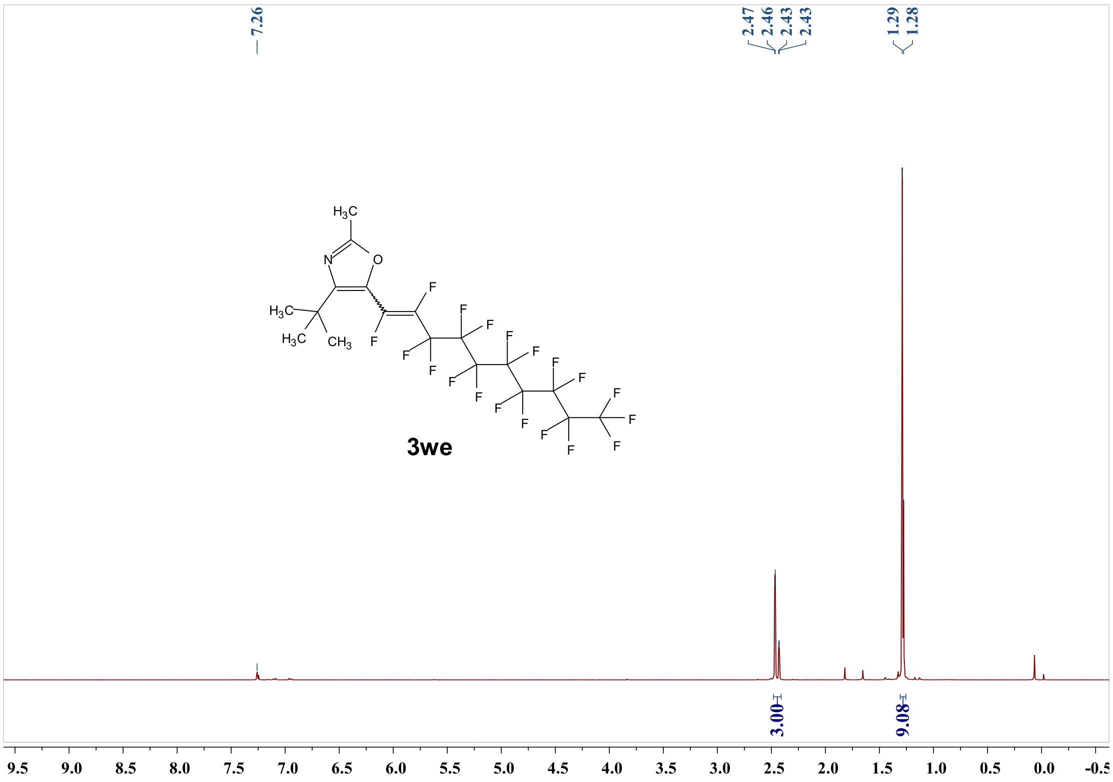

^{19}F NMR spectra of the product **3we** (376 MHz, $CDCl_3$)

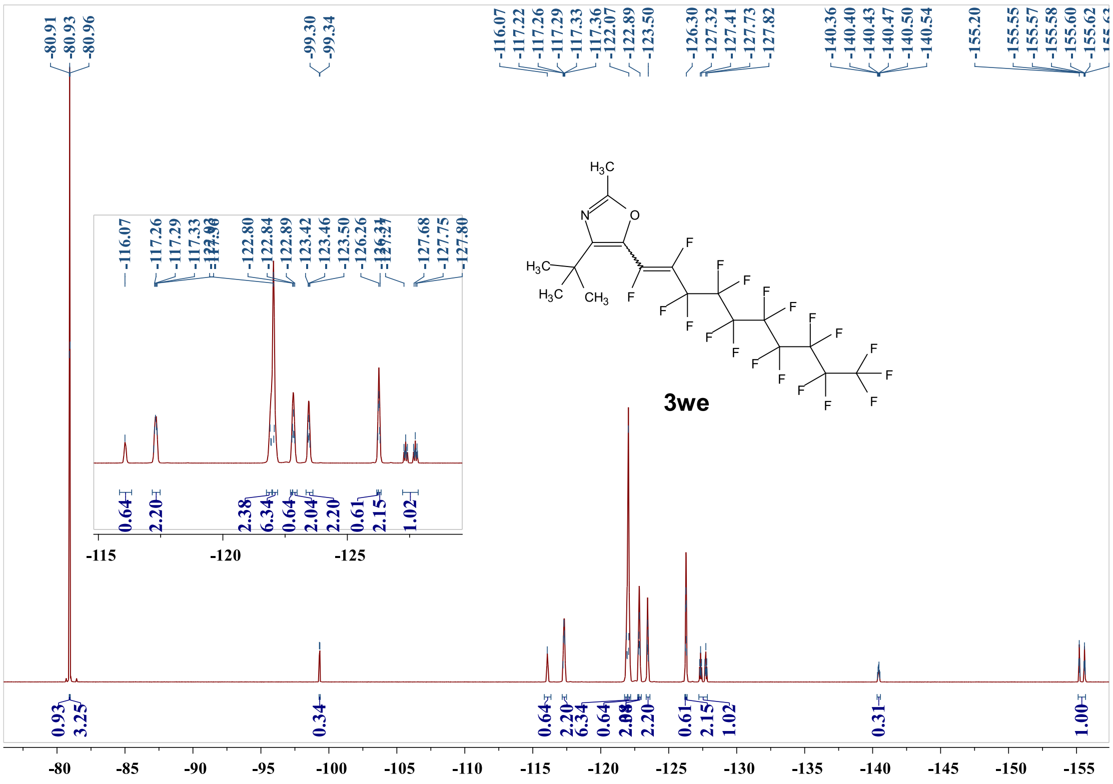

^{13}C NMR spectra of the product **3we** (100 MHz, $CDCl_3$)

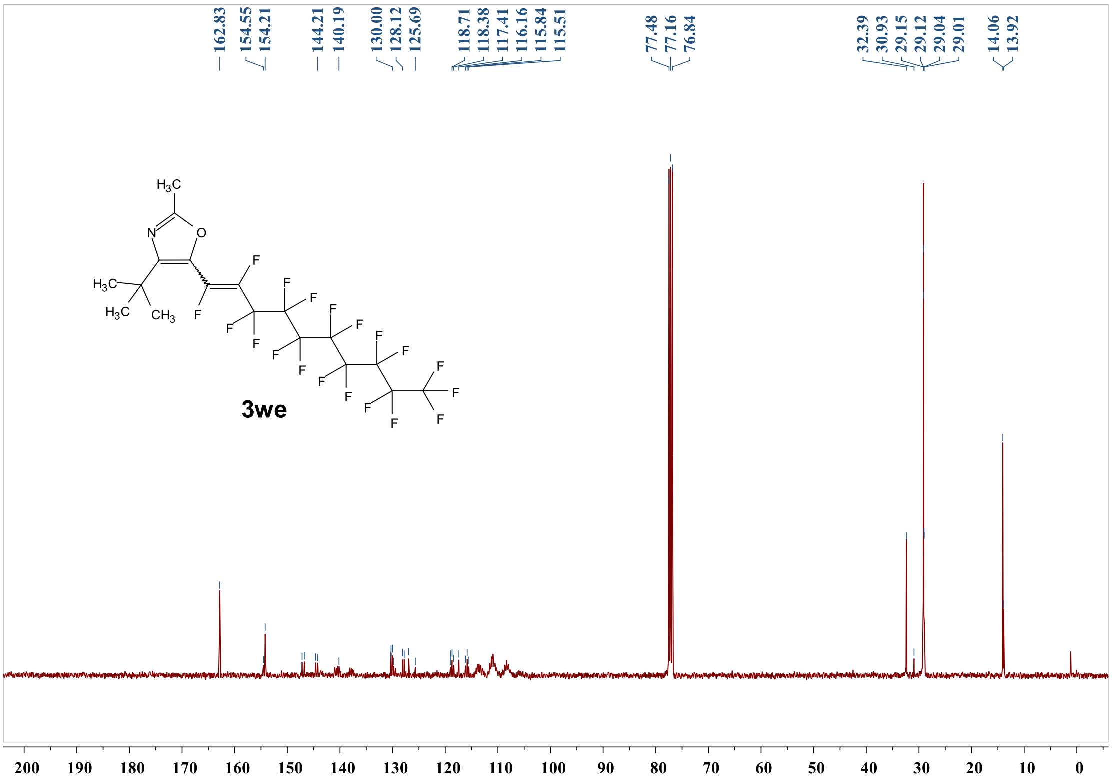

^{1}H NMR spectra of the product **3xa** (400 MHz, $CDCl_3$)

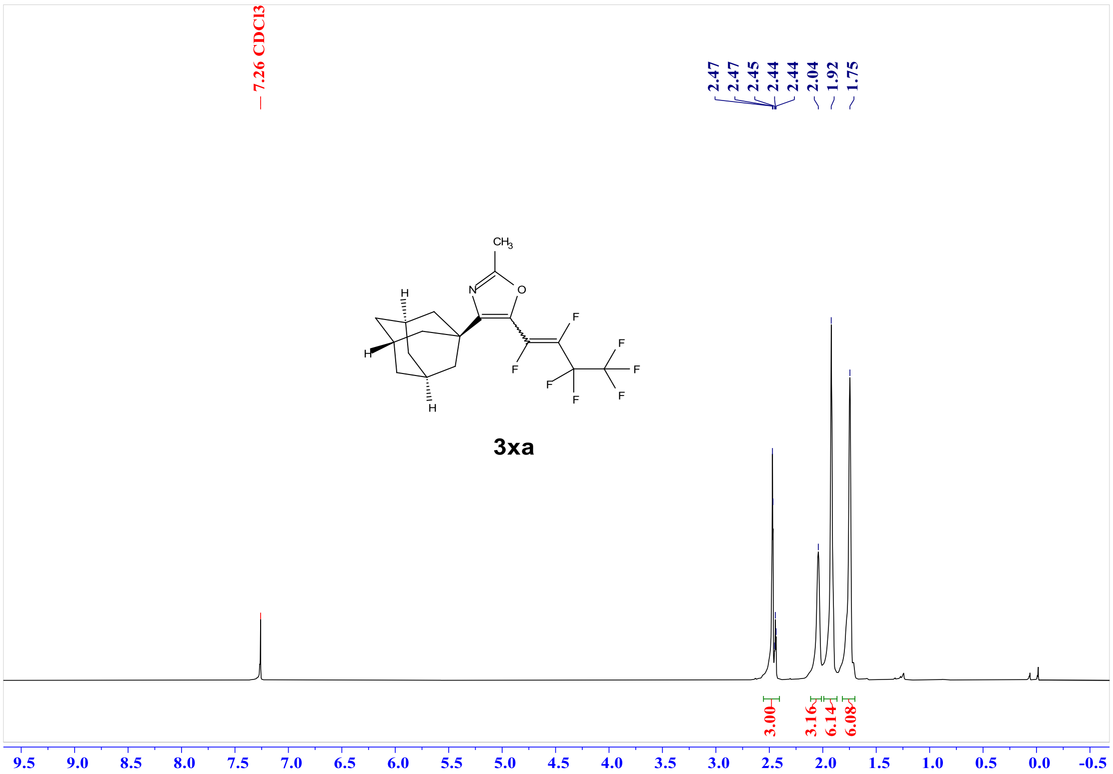

^{19}F NMR spectra of the product **3xa** (376 MHz, $CDCl_3$)

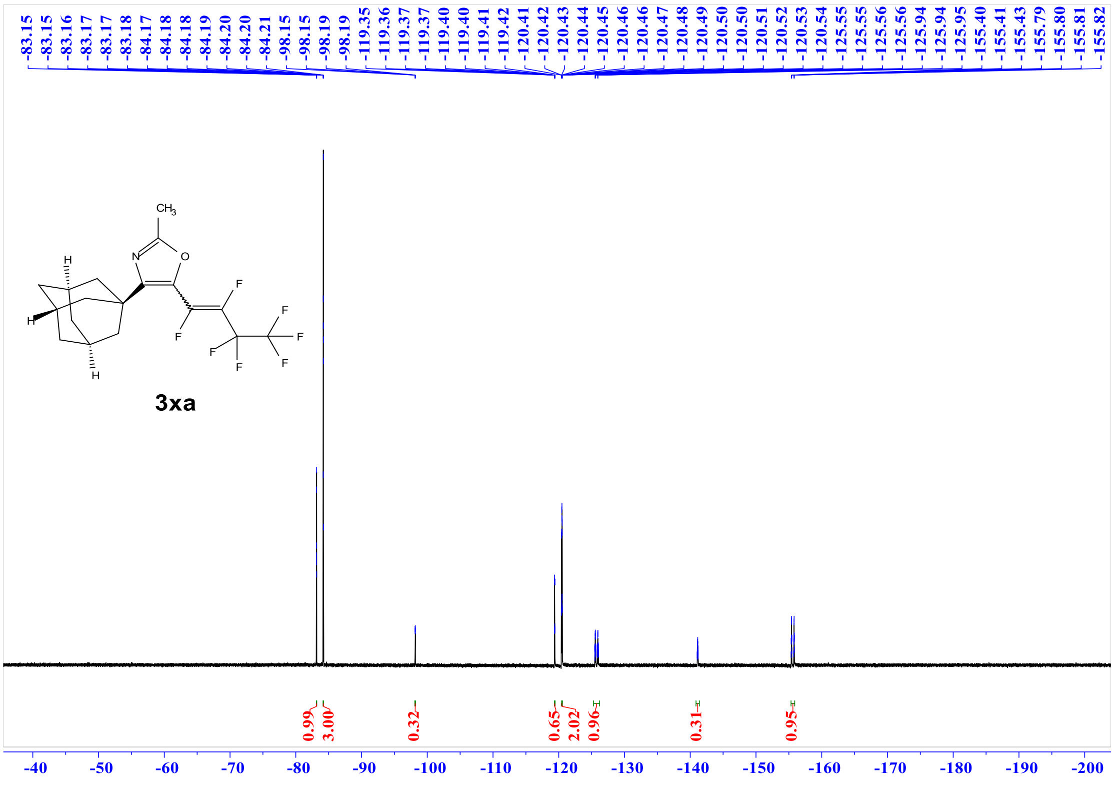

^{13}C NMR spectra of the product **3xa** (100 MHz, $CDCl_3$)

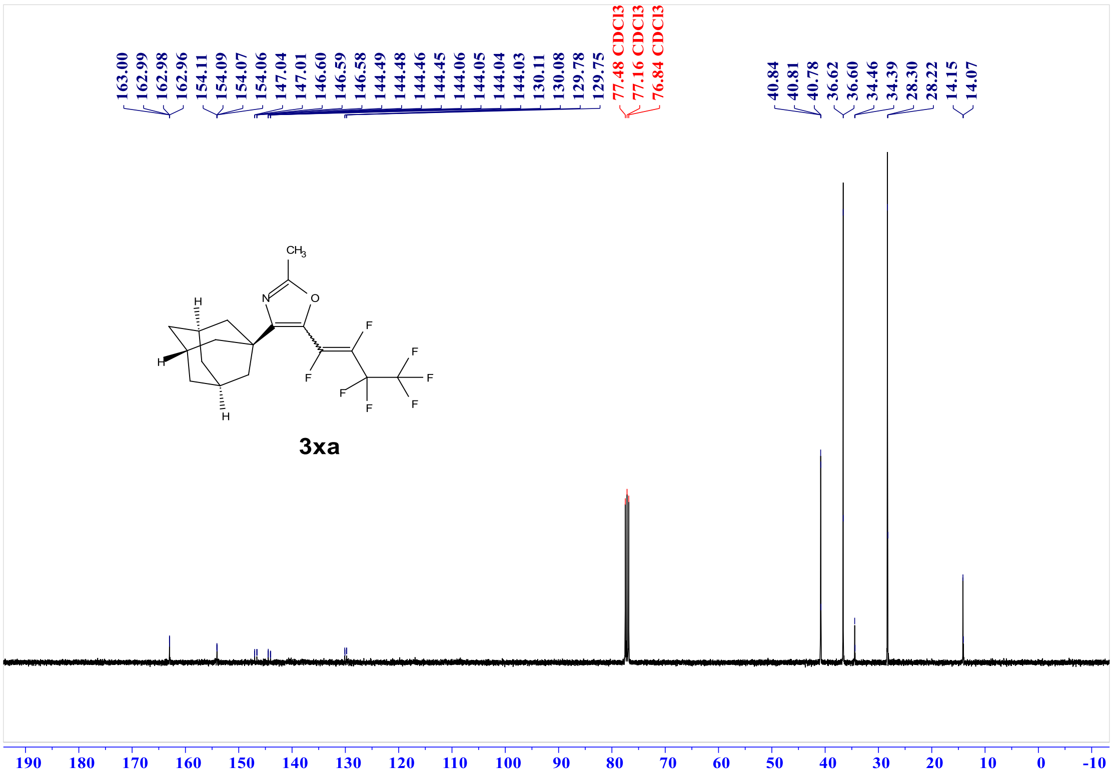

^{1}H NMR spectra of the product **3ye** (400 MHz, $CDCl_3$)

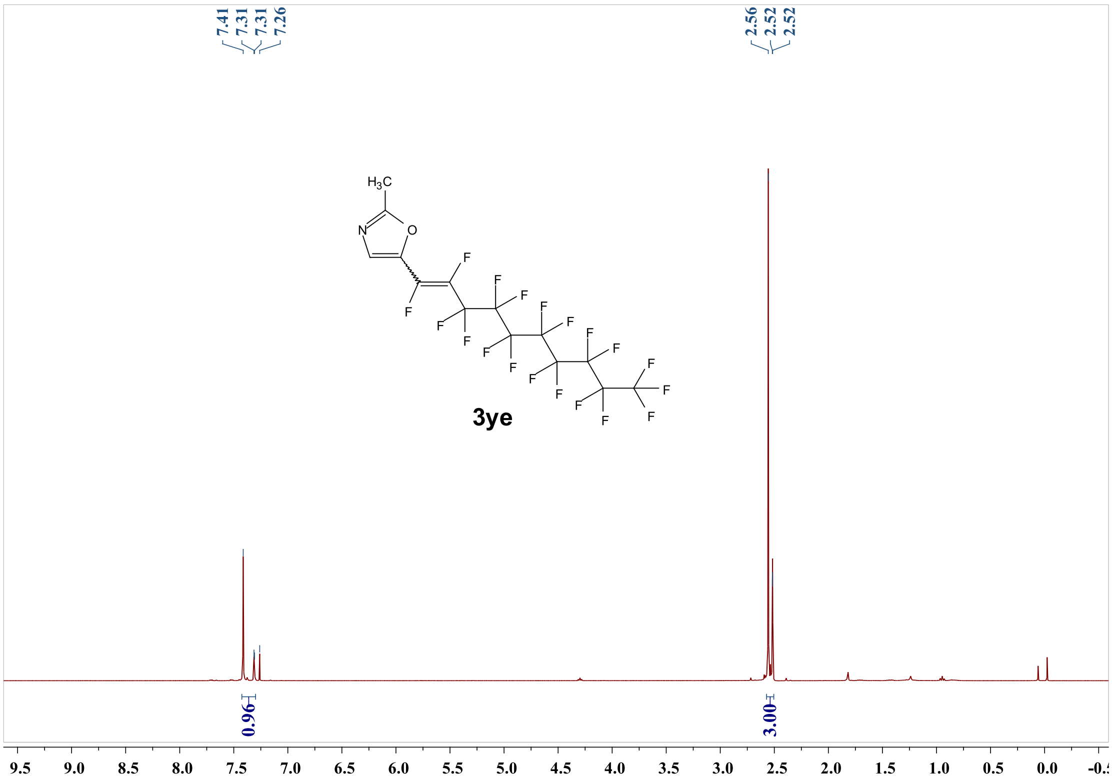

^{19}F NMR spectra of the product **3ye** (376 MHz, $CDCl_3$)

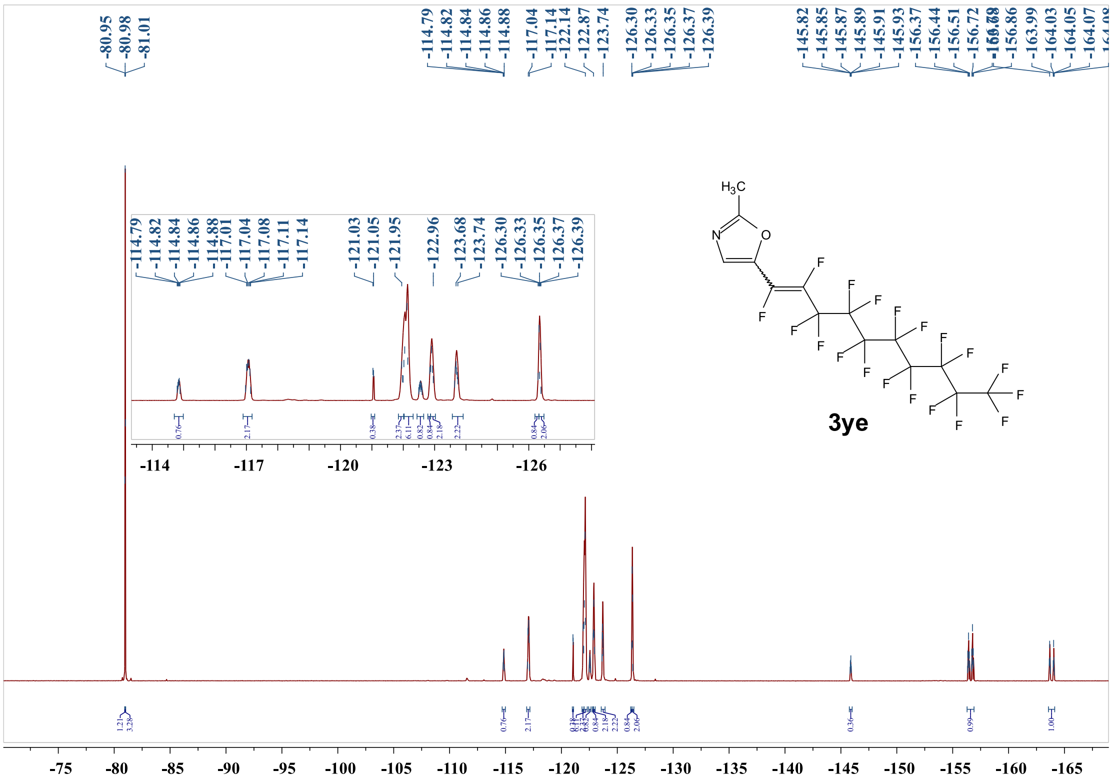

^{13}C NMR spectra of the product **3ye** (100 MHz, $CDCl_3$)

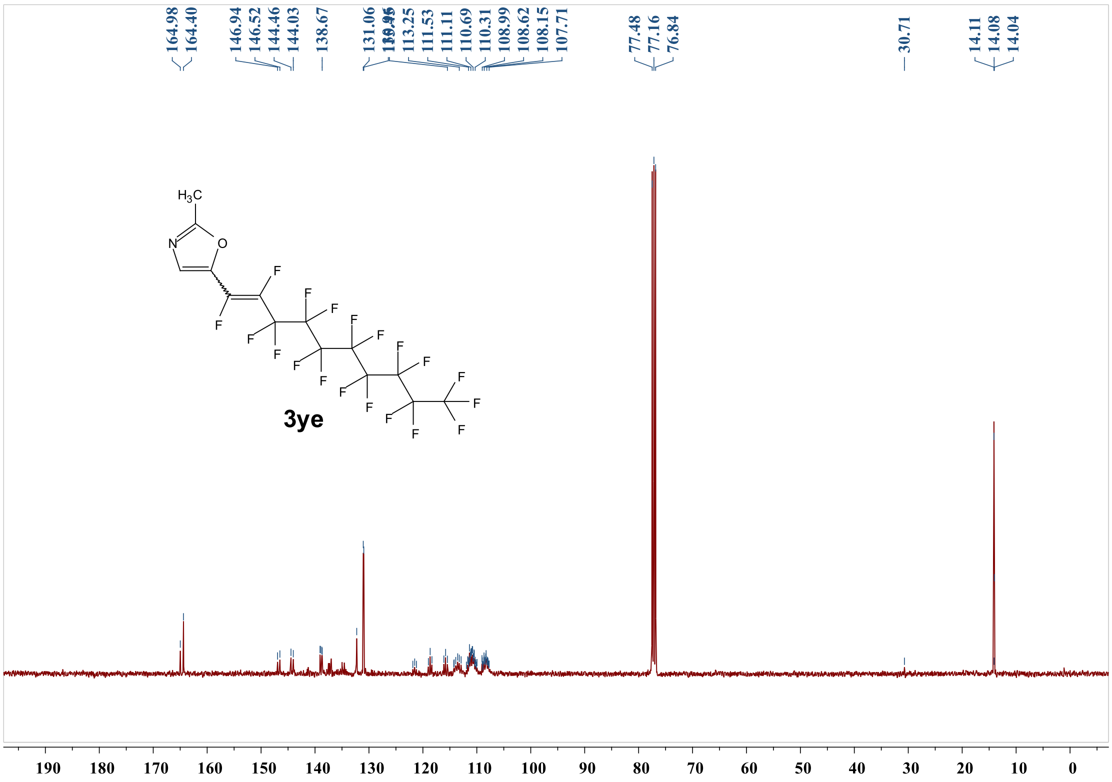

^1H NMR spectra of the product **3ab** (400 MHz, $CDCl_3$)

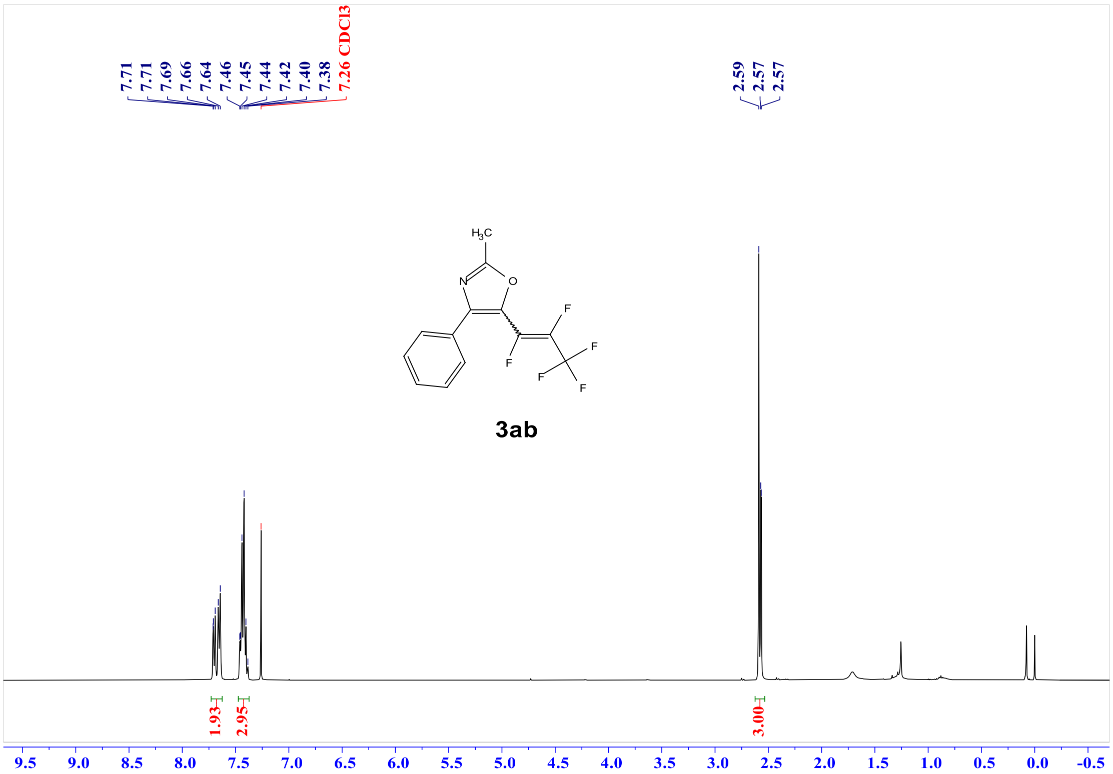

^{19}F NMR spectra of the product **3ab** (376 MHz, $CDCl_3$)

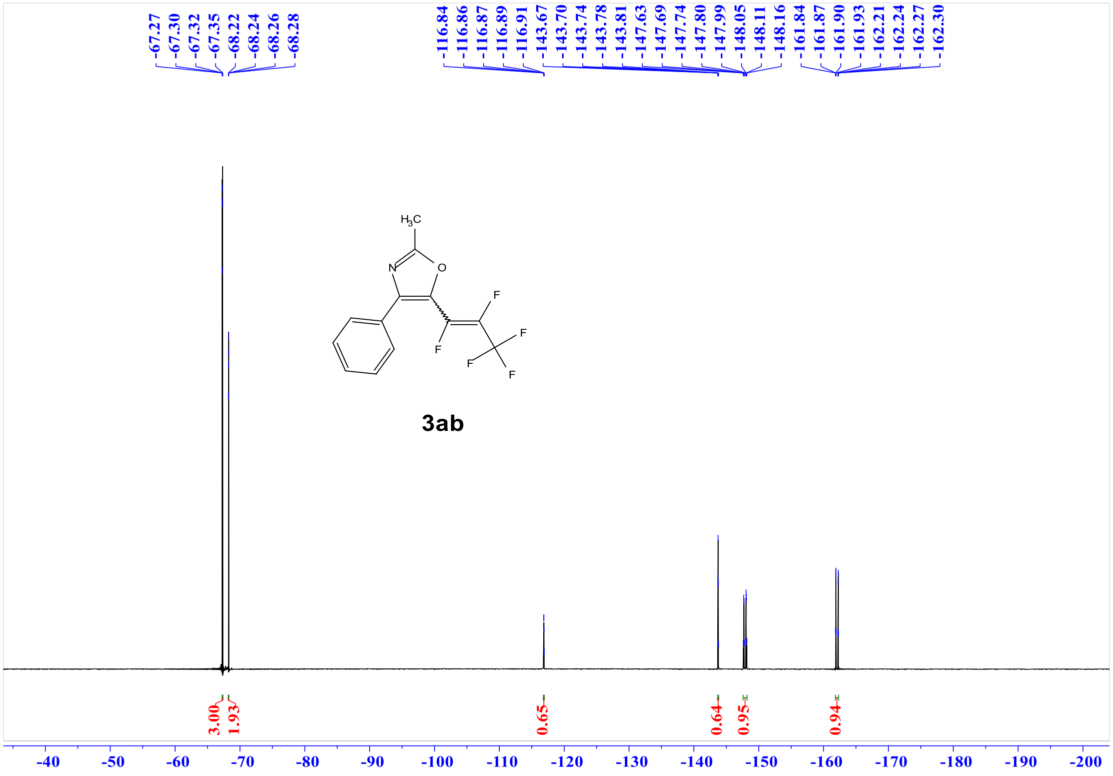

^{13}C NMR spectra of the product **3ab** (100 MHz, $CDCl_3$)

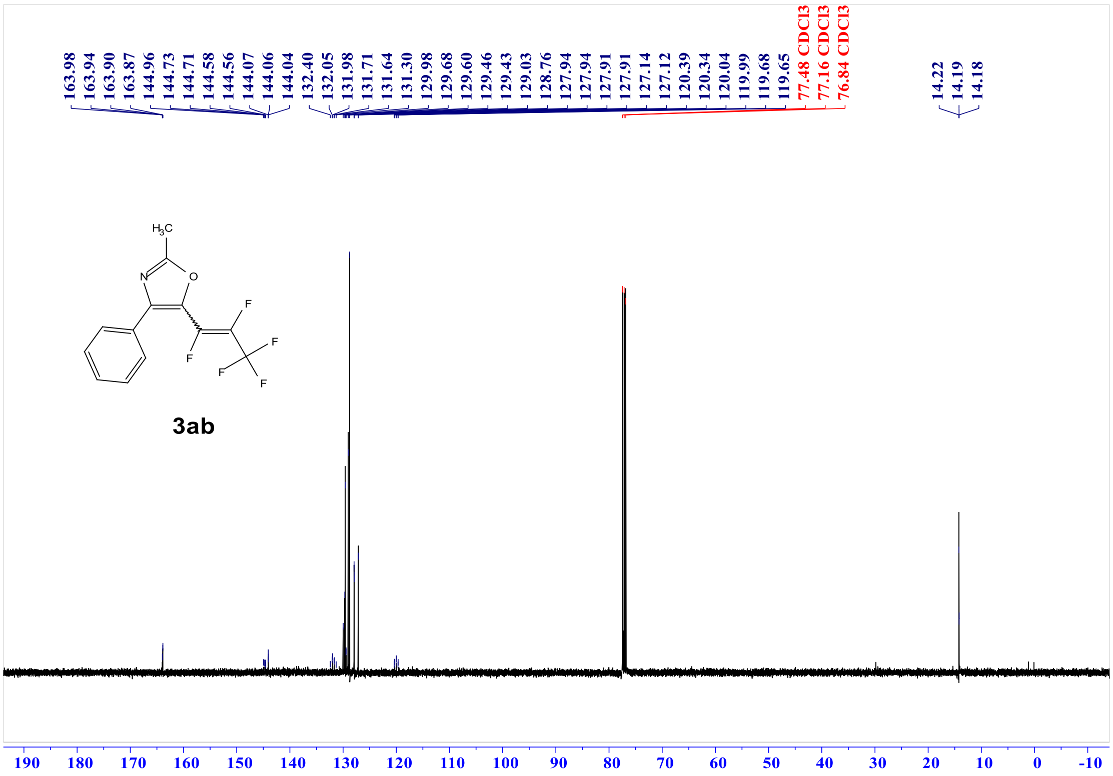

^{1}H NMR spectra of the product **3ac** (400 MHz, CDCl$_3$)

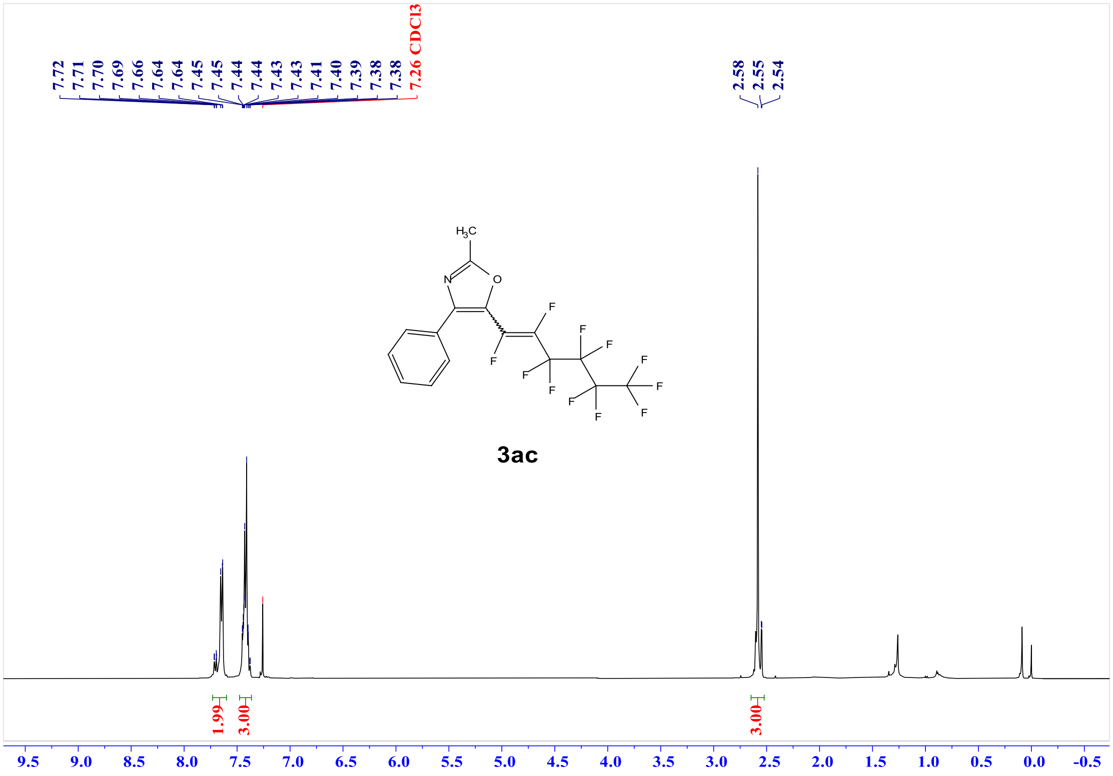

^{19}F NMR spectra of the product **3ac** (376 MHz, CDCl$_3$)

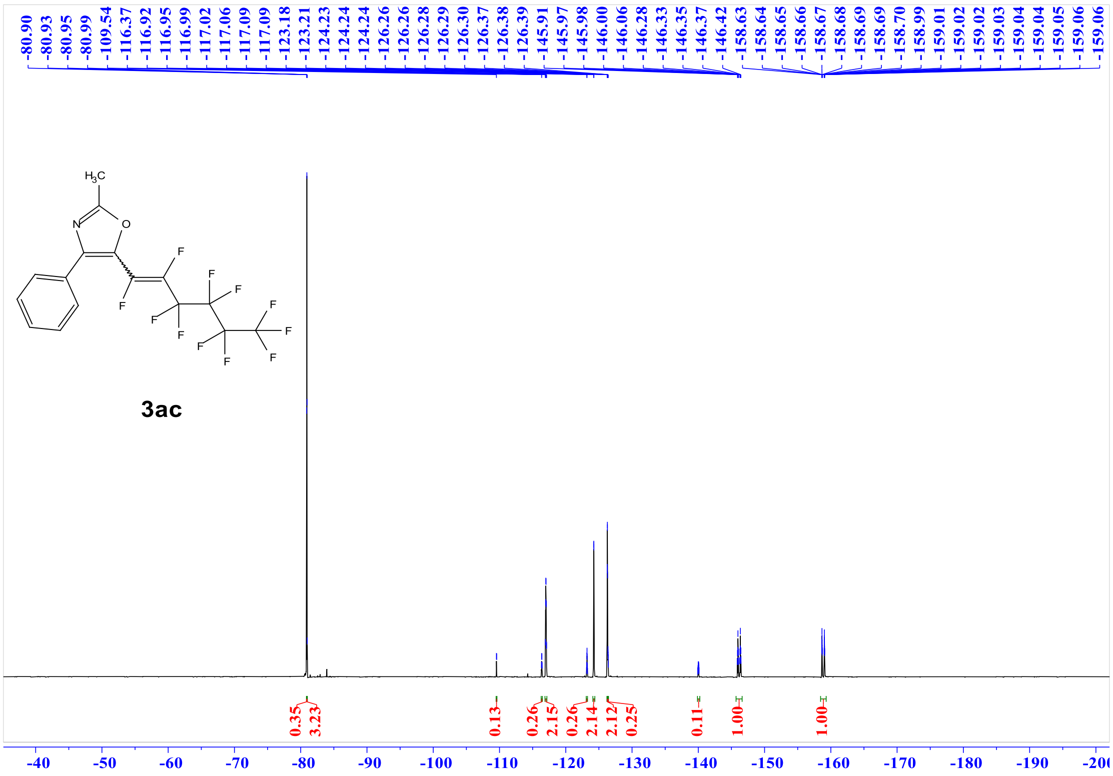

^{13}C NMR spectra of the product **3ac** (100 MHz, CDCl$_3$)

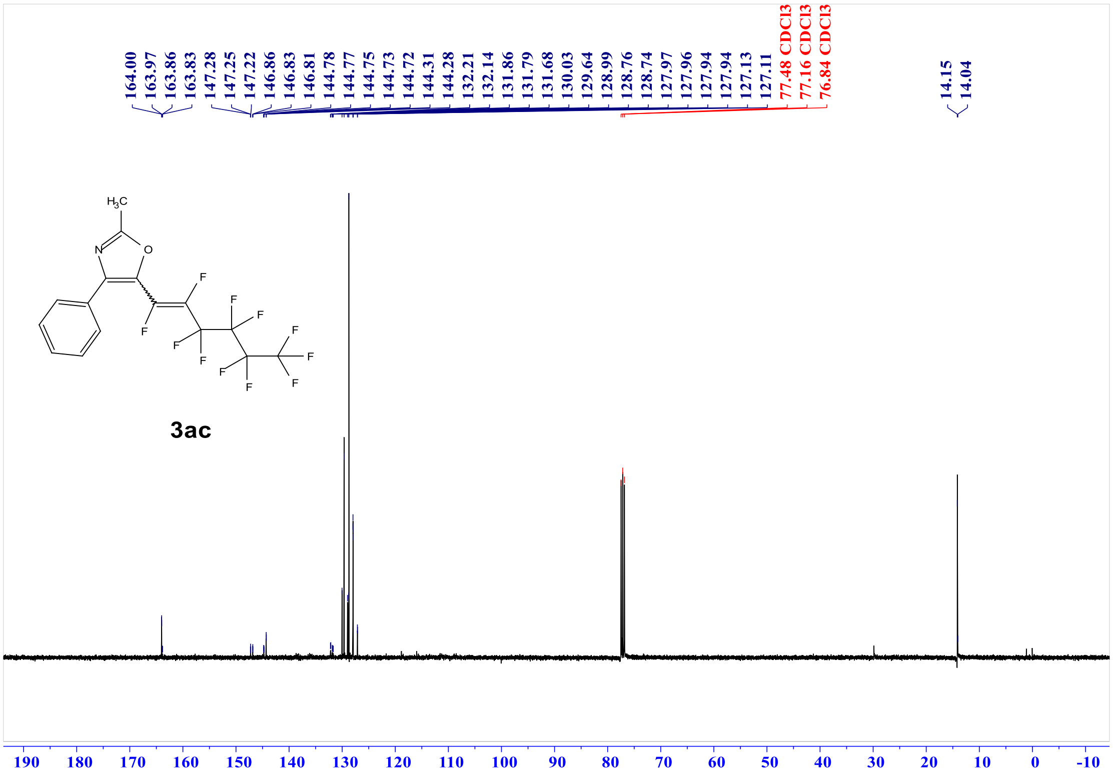

^{1}H NMR spectra of the product **3ad** (400 MHz, CDCl$_3$)

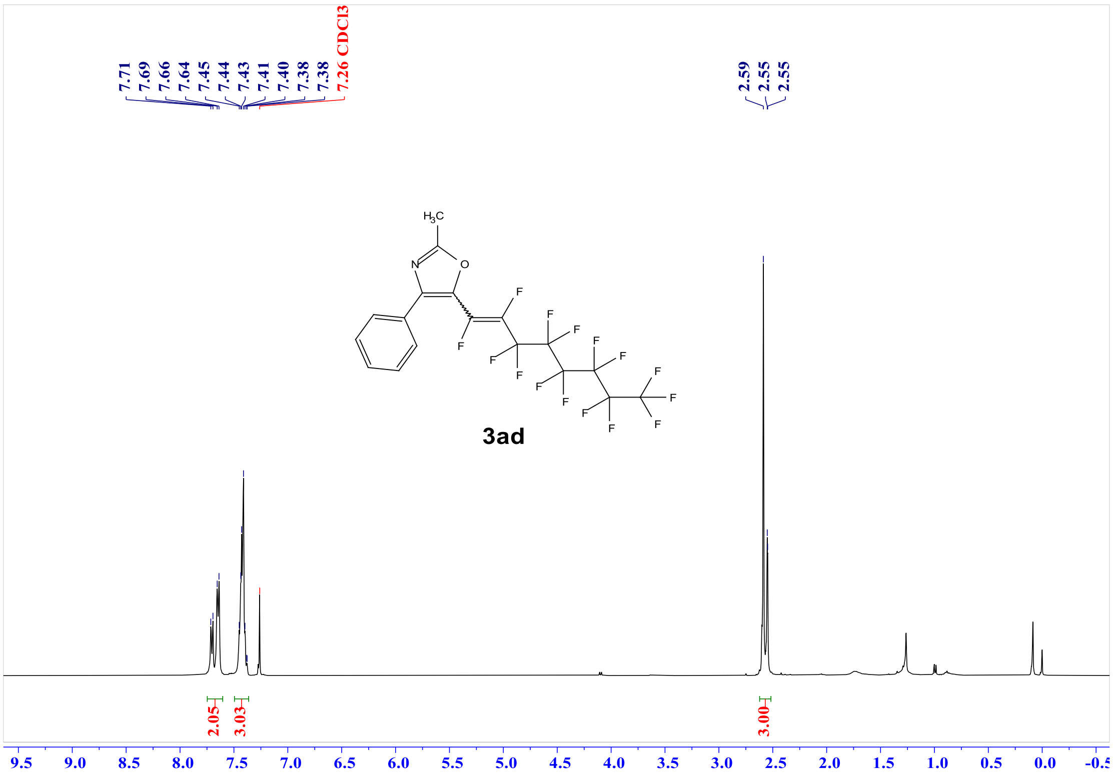

^{19}F NMR spectra of the product **3ad** (376 MHz, $CDCl_3$)

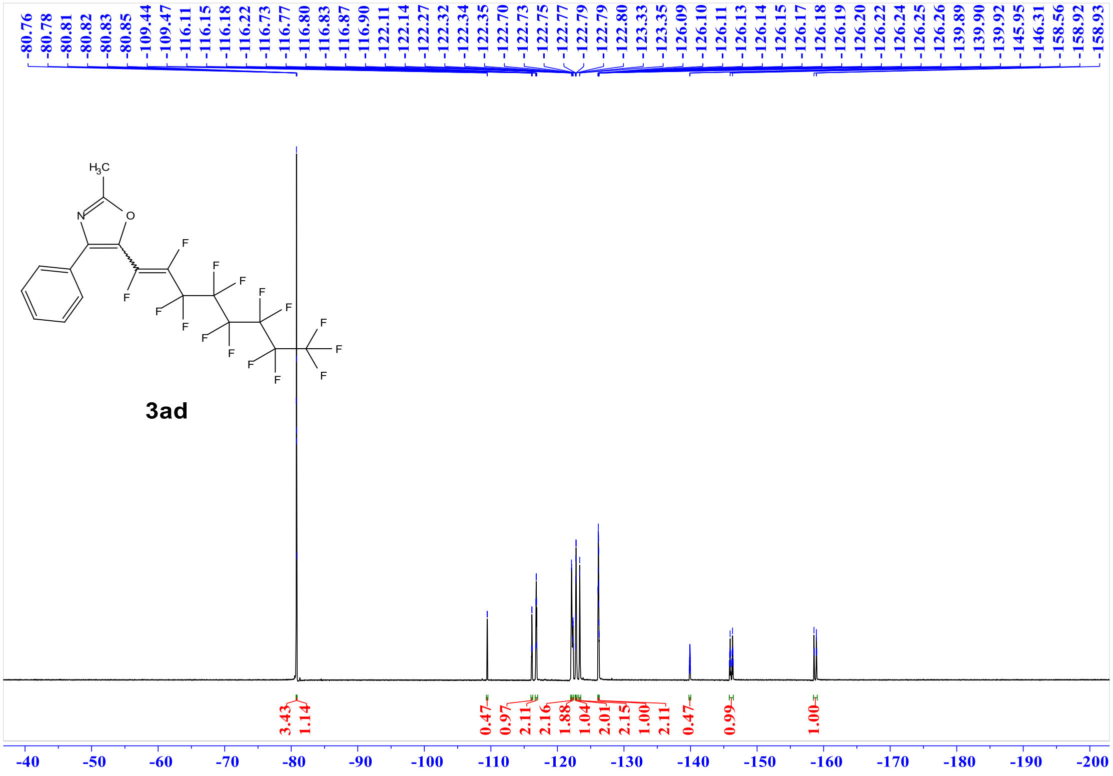

^{13}C NMR spectra of the product **3ad** (100 MHz, $CDCl_3$)

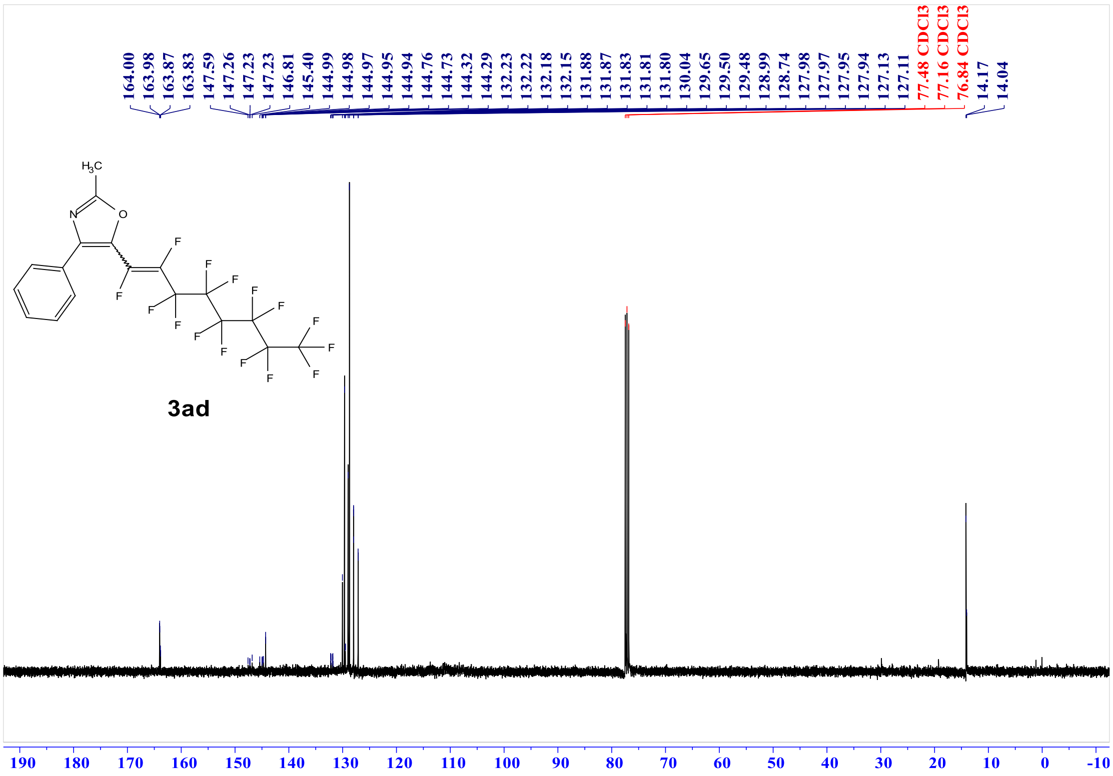

^{1}H NMR spectra of the product **3ae** (400 MHz, $CDCl_3$)

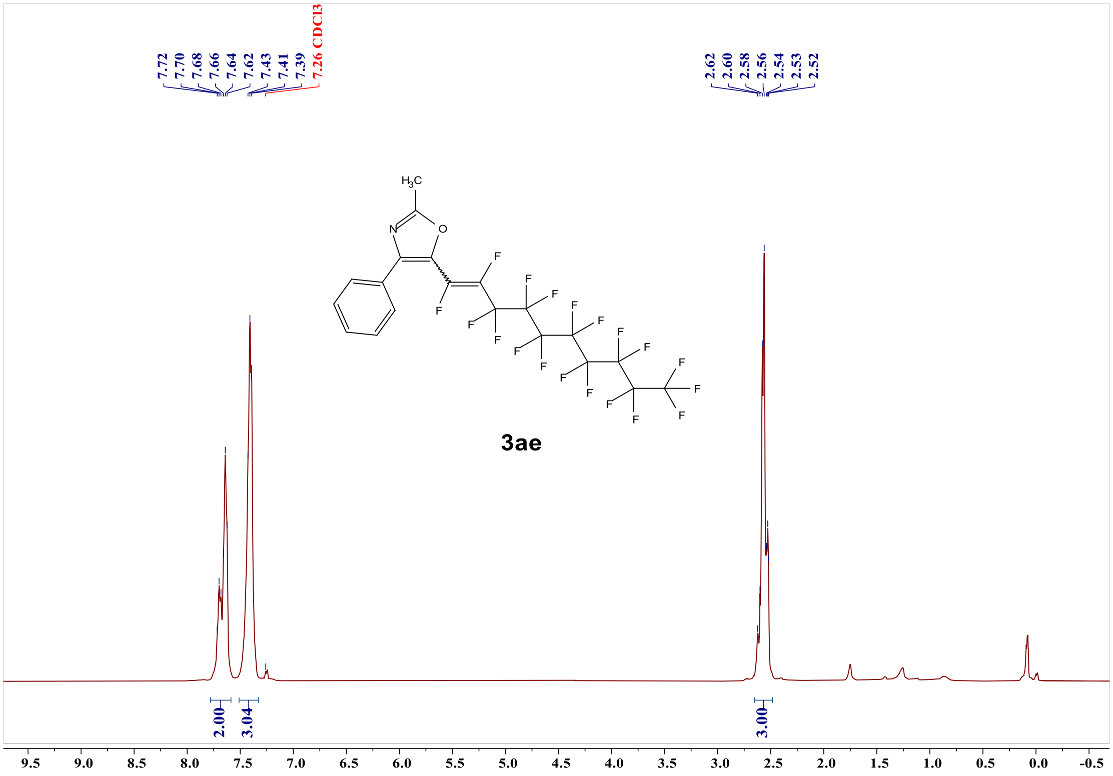

^{19}F NMR spectra of the product **3ae** (376 MHz, $CDCl_3$)

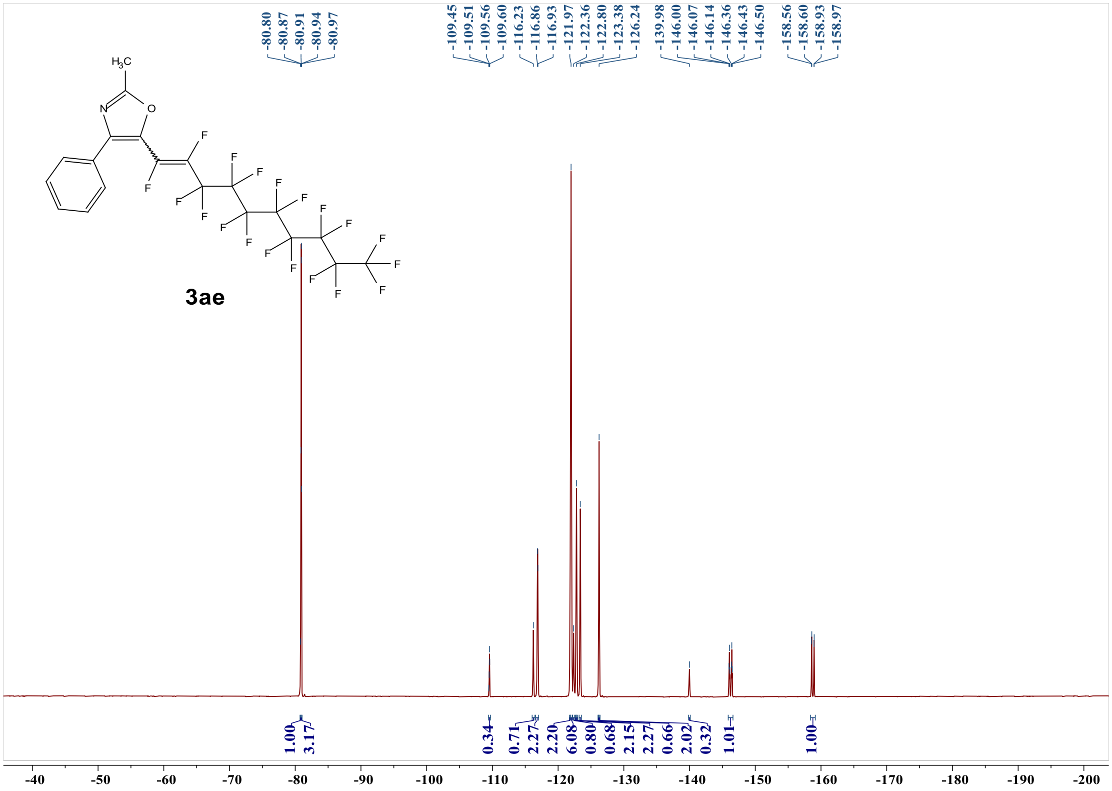

^{13}C NMR spectra of the product **3ae** (100 MHz, $CDCl_3$)

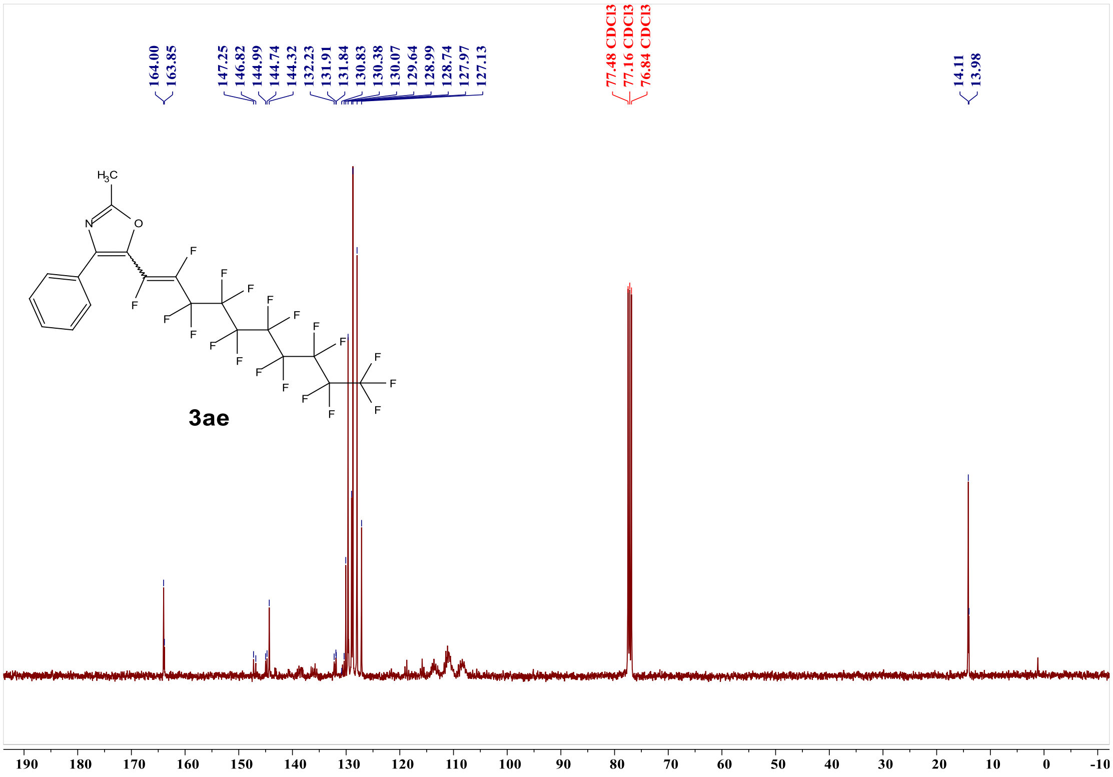

^{1}H NMR spectra of the product **3af** (400 MHz, $CDCl_3$)

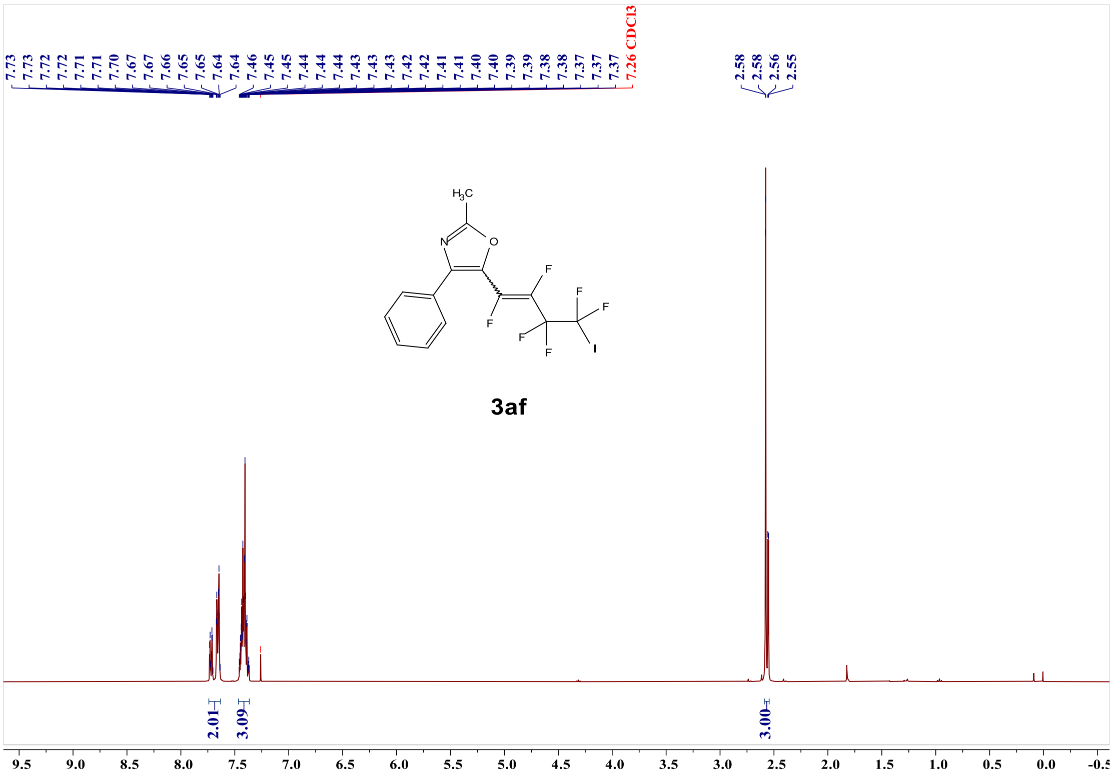

^{19}F NMR spectra of the product **3af** (376 MHz, CDCl3)

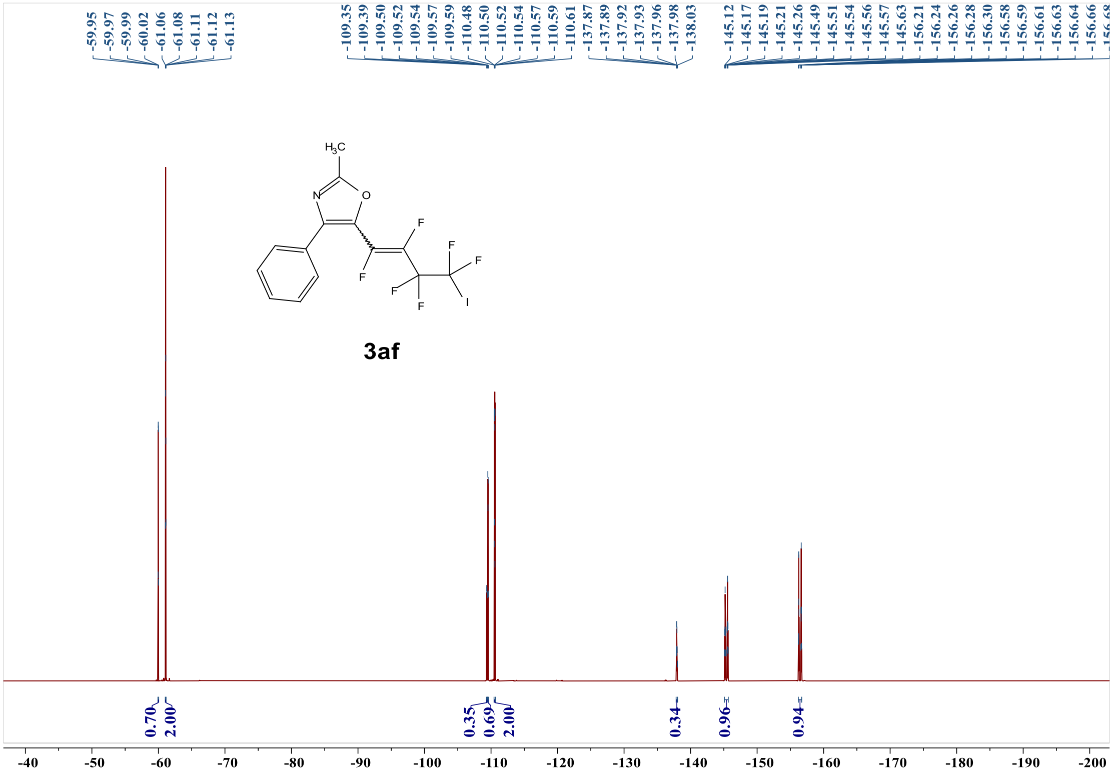

^{13}C NMR spectra of the product **3af** (100 MHz, CDCl3)

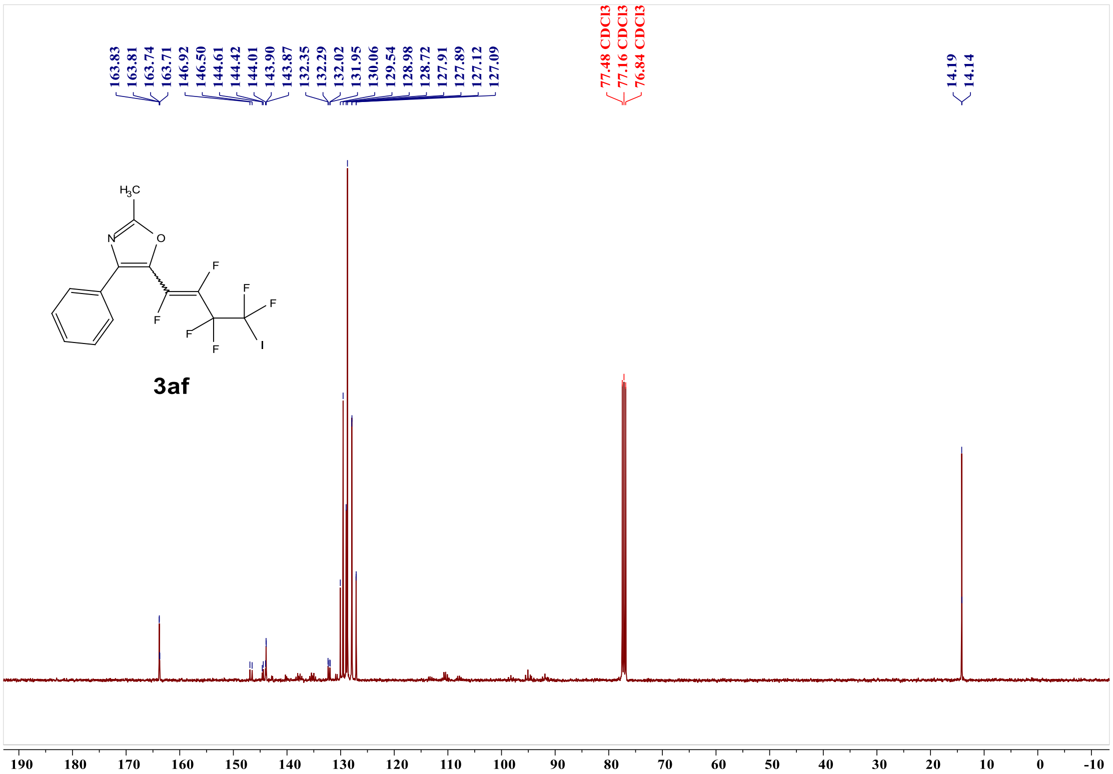

^{1}H NMR spectra of the product **3ag** (400 MHz, $CDCl_3$)

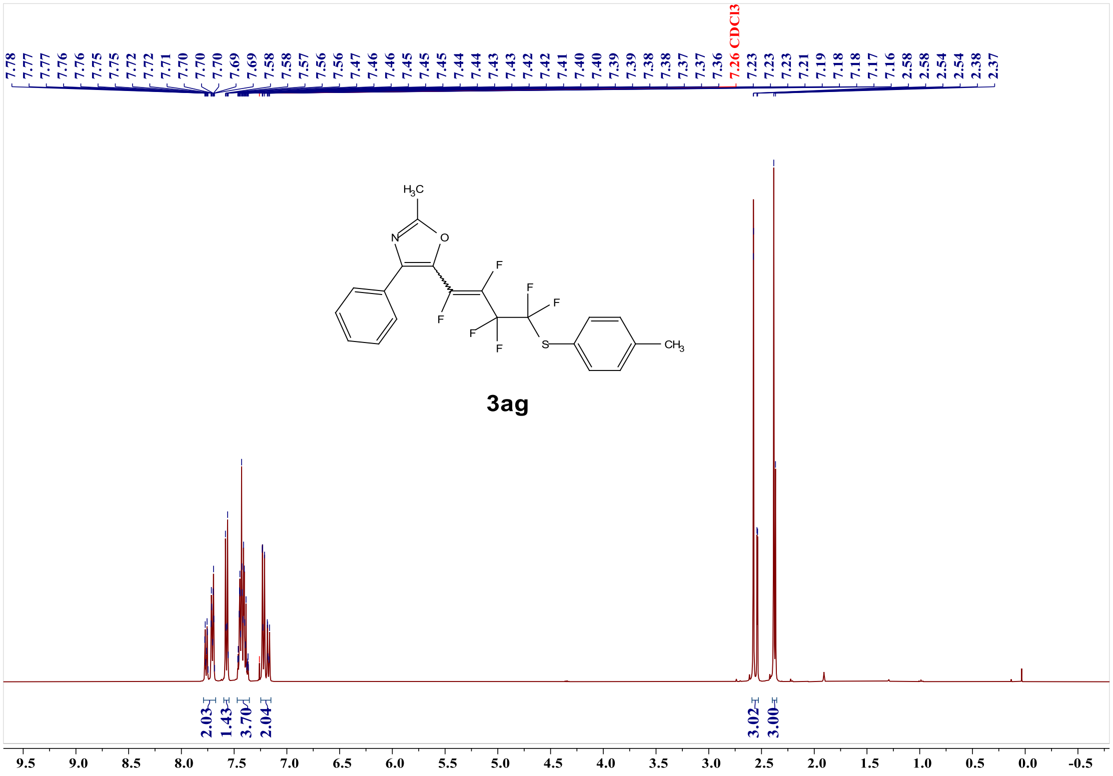

^{19}F NMR spectra of the product **3ag** (376 MHz, $CDCl_3$)

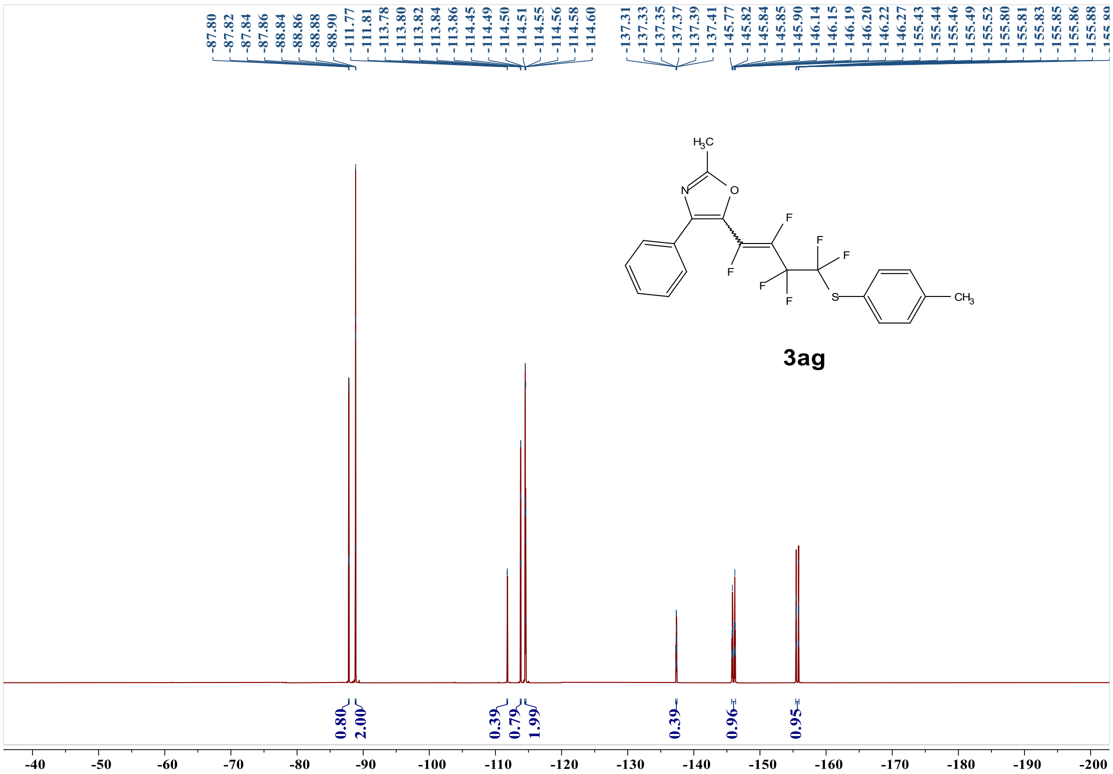

^{13}C NMR spectra of the product **3ag** (100 MHz, $CDCl_3$)

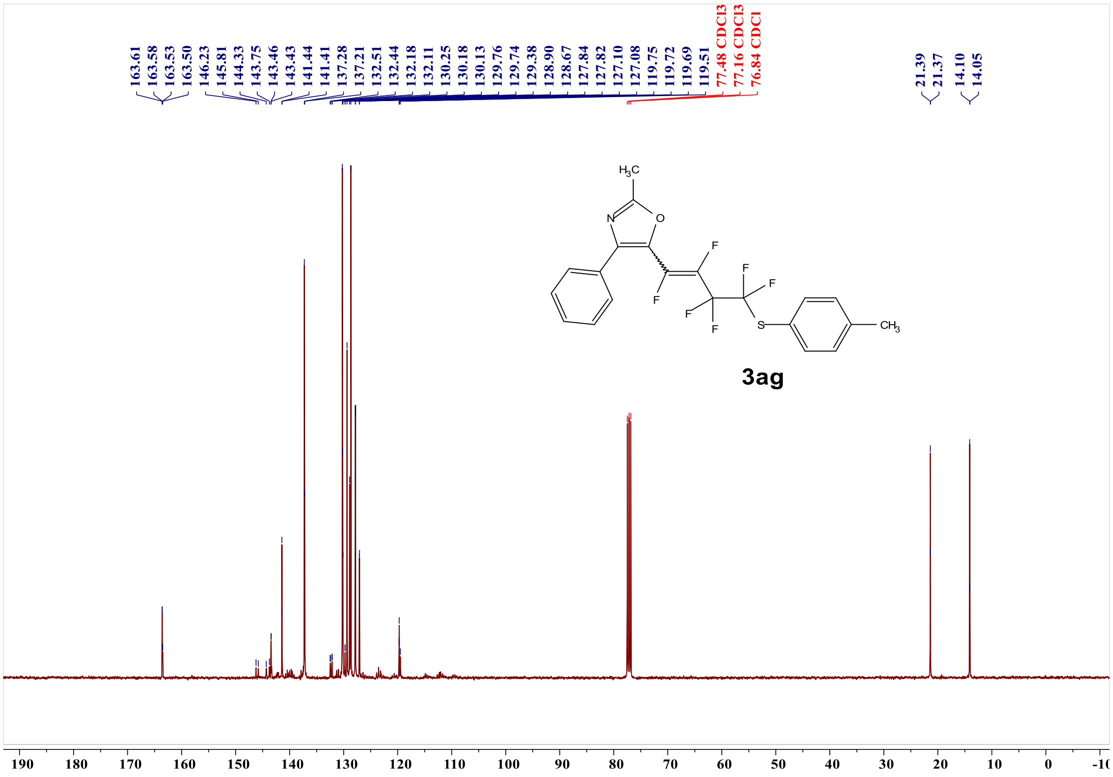

^{1}H NMR spectra of the product **3ah** (400 MHz, $CDCl_3$)

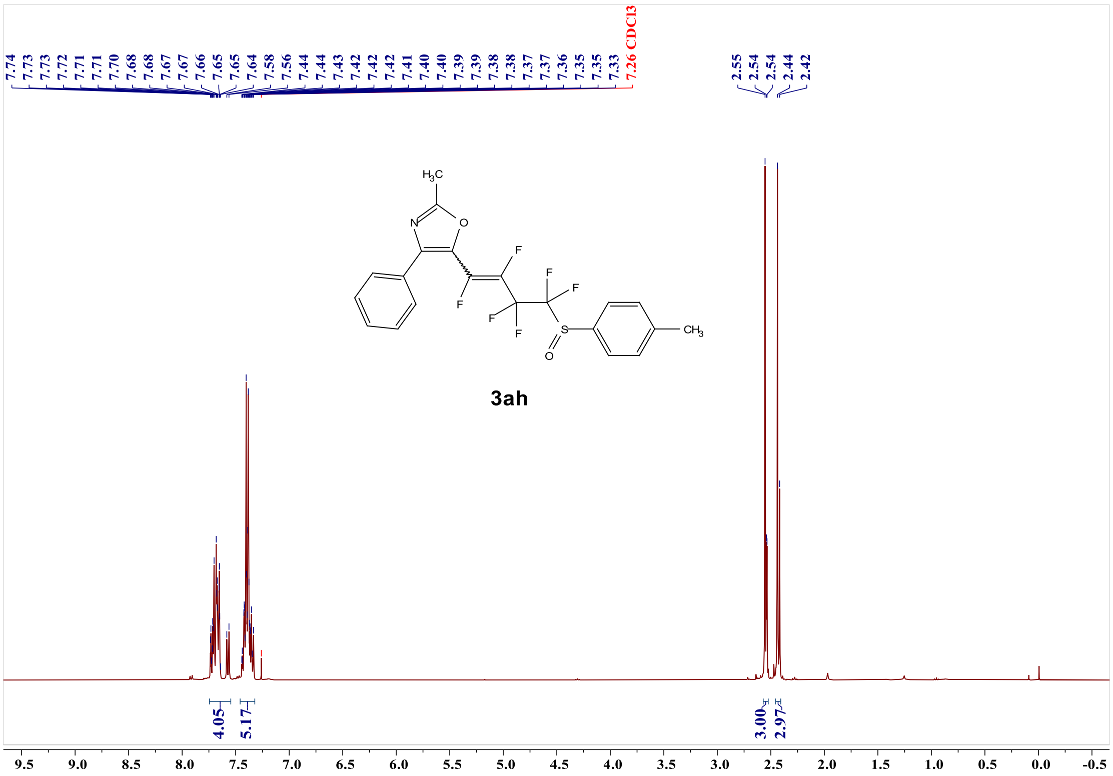

^{19}F NMR spectra of the product **3ah** (376 MHz, $CDCl_3$)

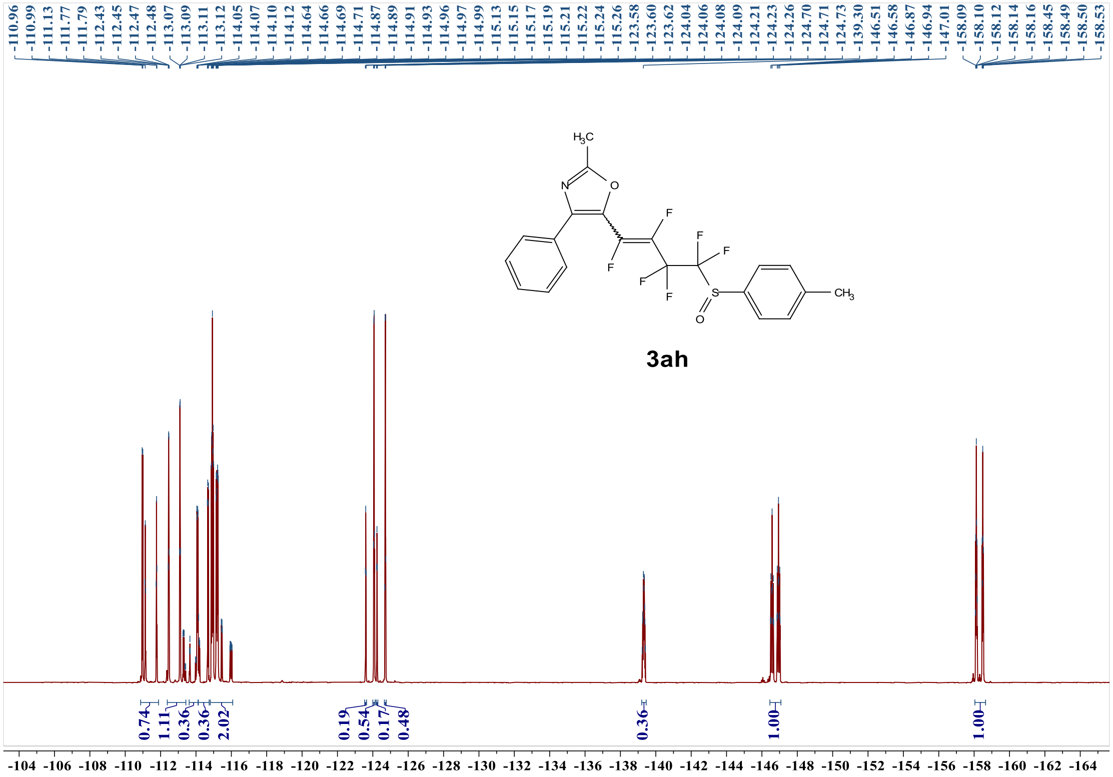

^{13}C NMR spectra of the product **3ah** (100 MHz, $CDCl_3$)

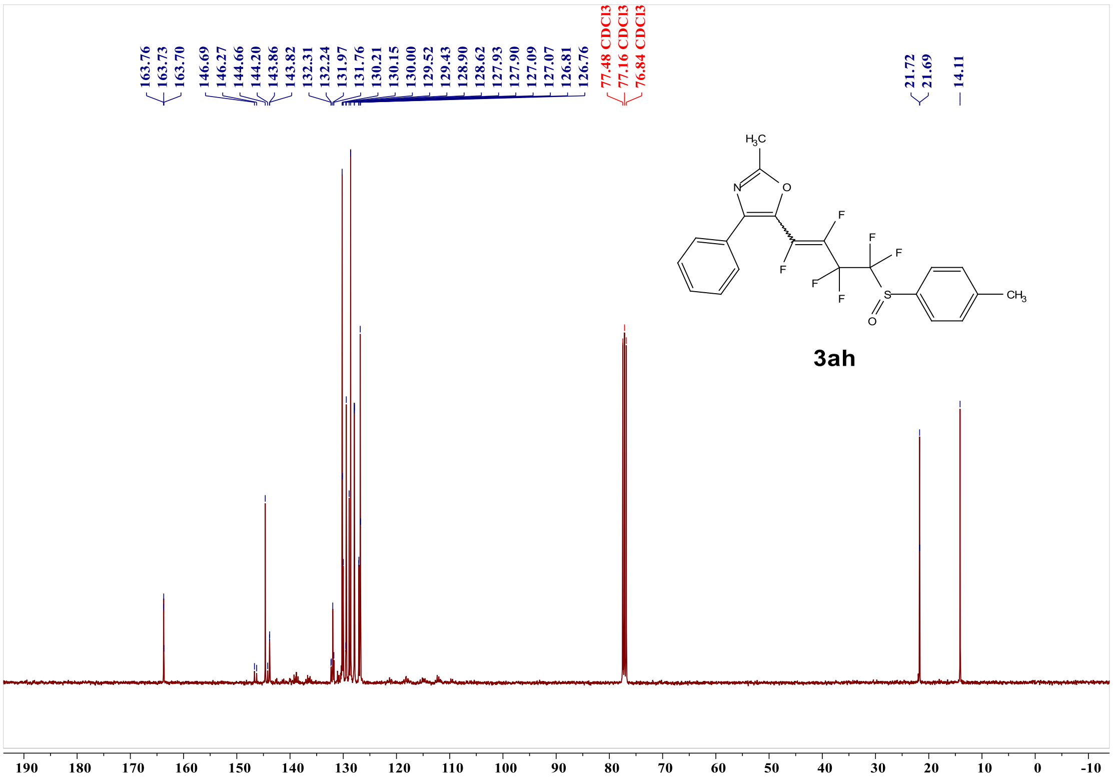

^{1}H NMR spectra of the product **3ai** (400 MHz, $CDCl_3$)

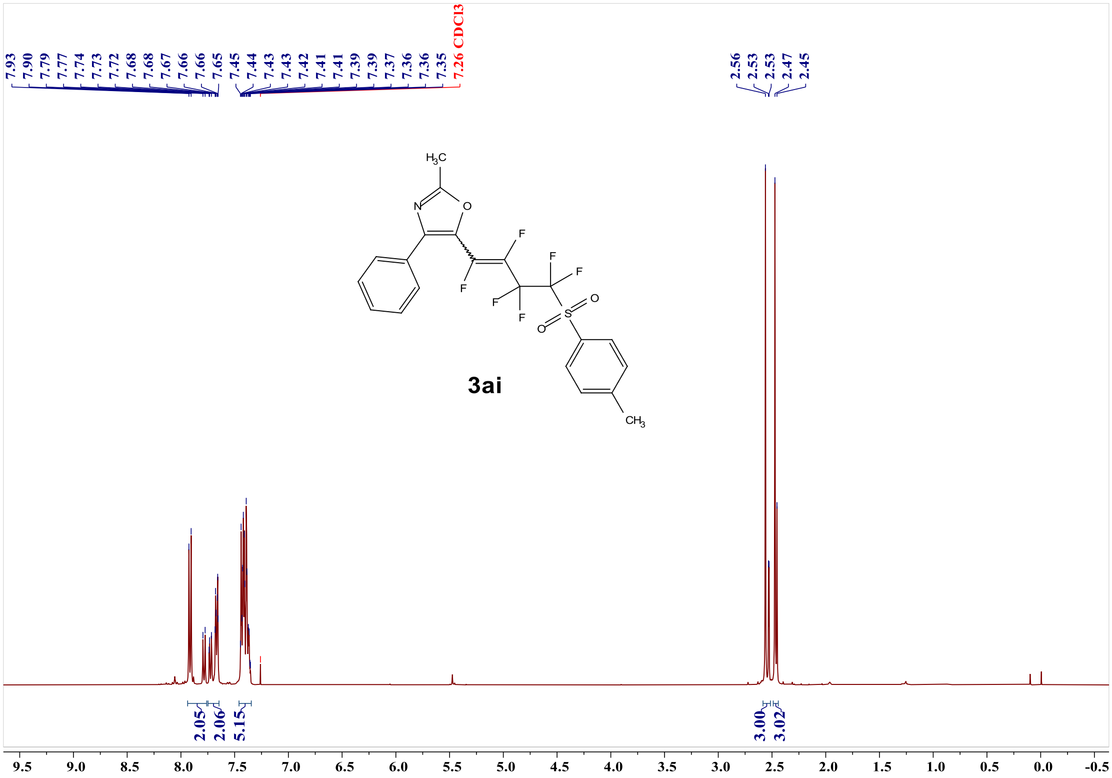

^{19}F NMR spectra of the product **3ai** (376 MHz, $CDCl_3$)

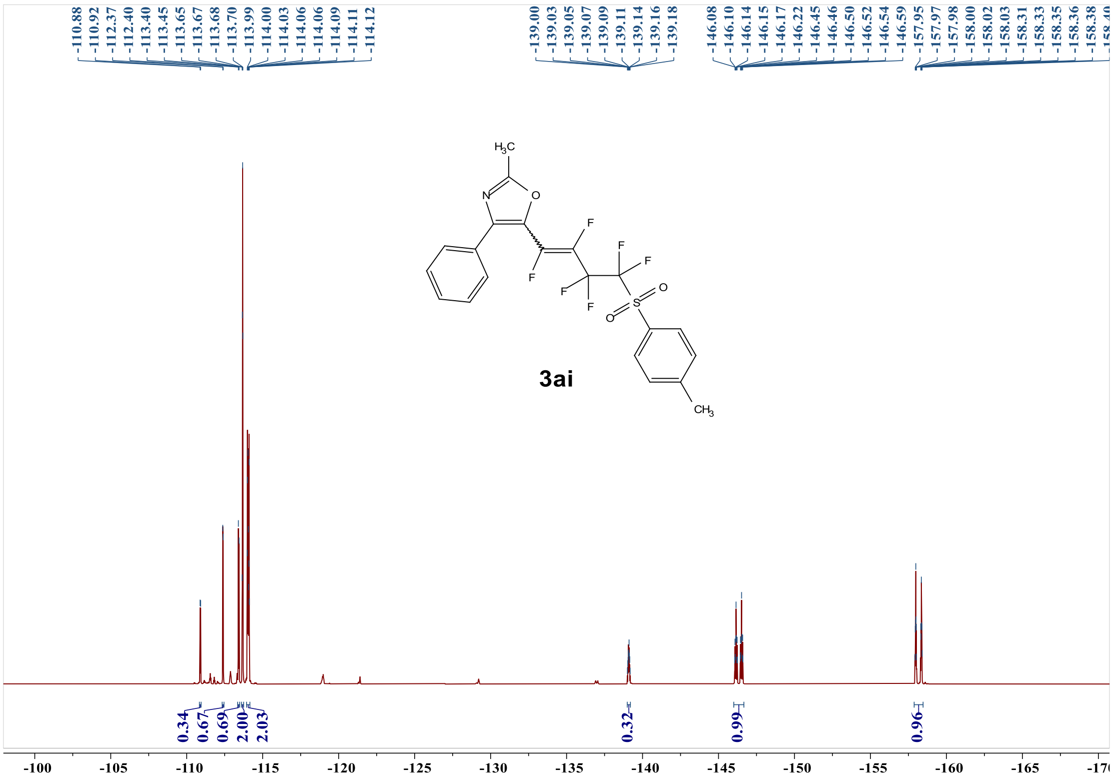

^{13}C NMR spectra of the product **3ai** (100 MHz, $CDCl_3$)

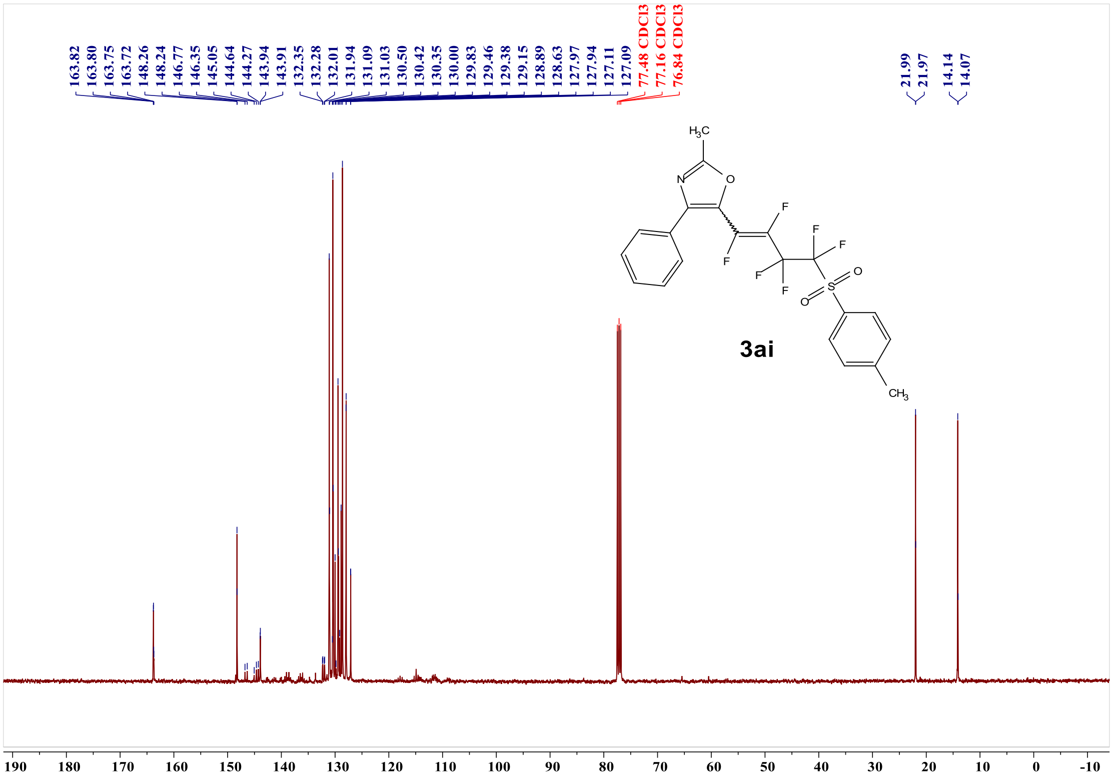

^{1}H NMR spectra of the product **3aj** (400 MHz, $CDCl_3$)

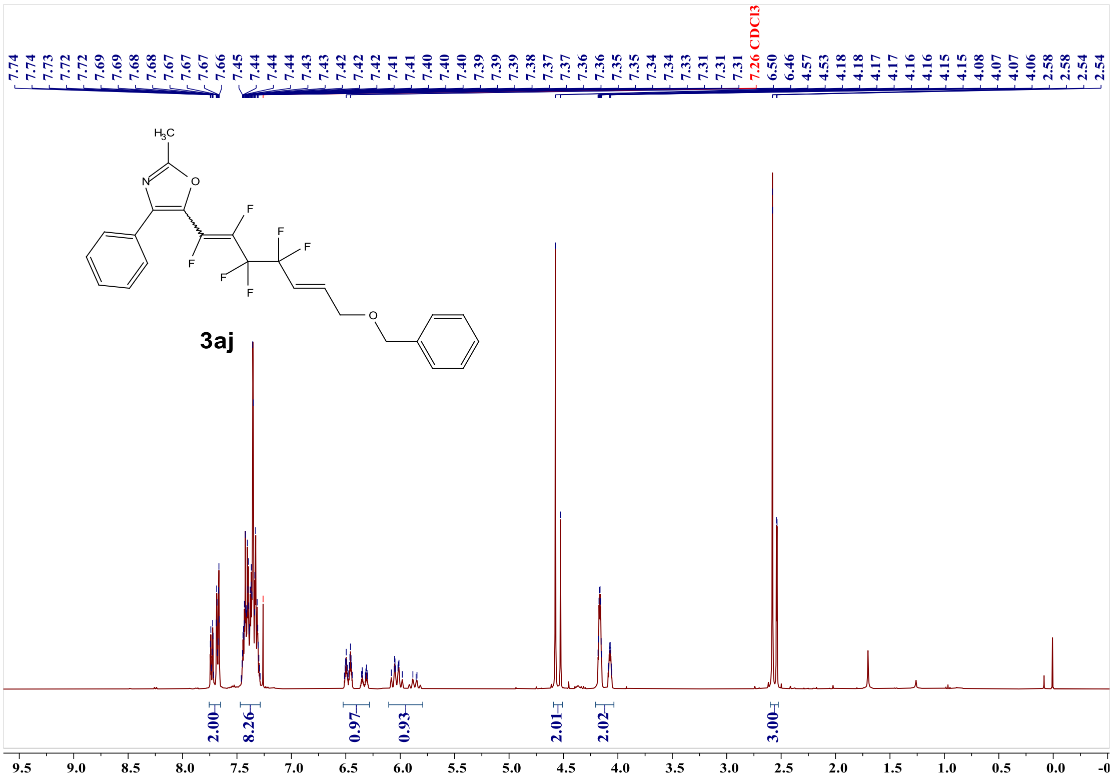

^{19}F NMR spectra of the product **3aj** (376 MHz, $CDCl_3$)

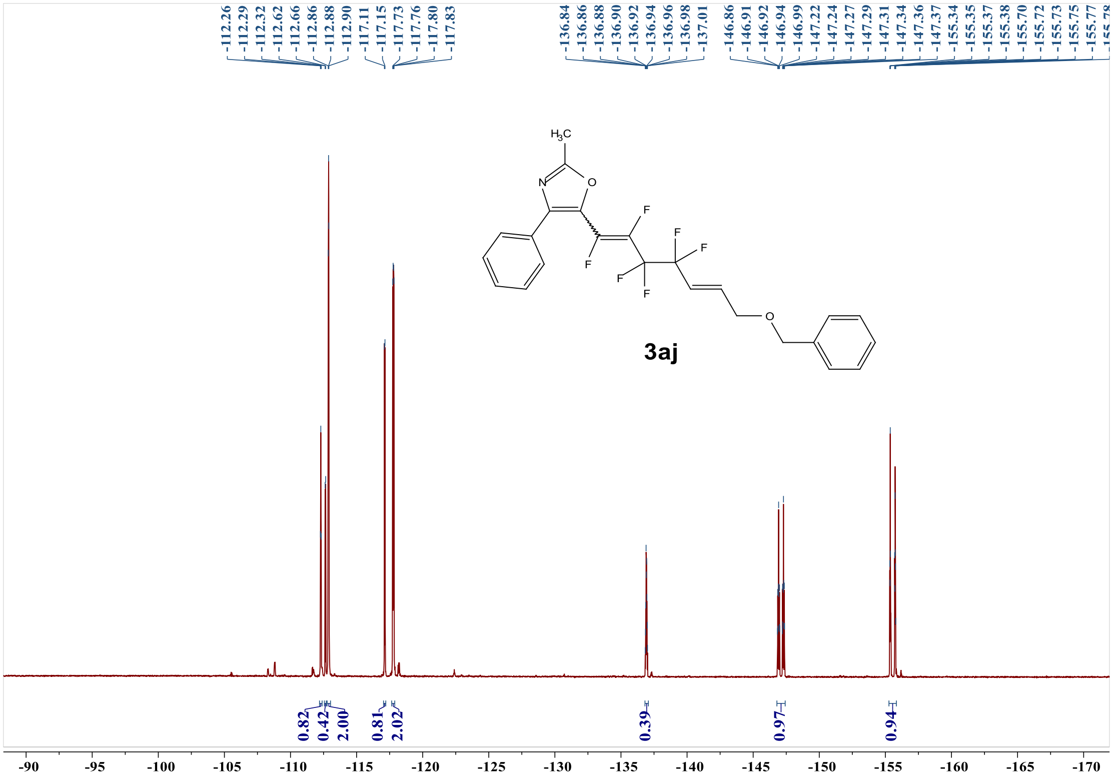

^{13}C NMR spectra of the product **3aj** (100 MHz, $CDCl_3$)

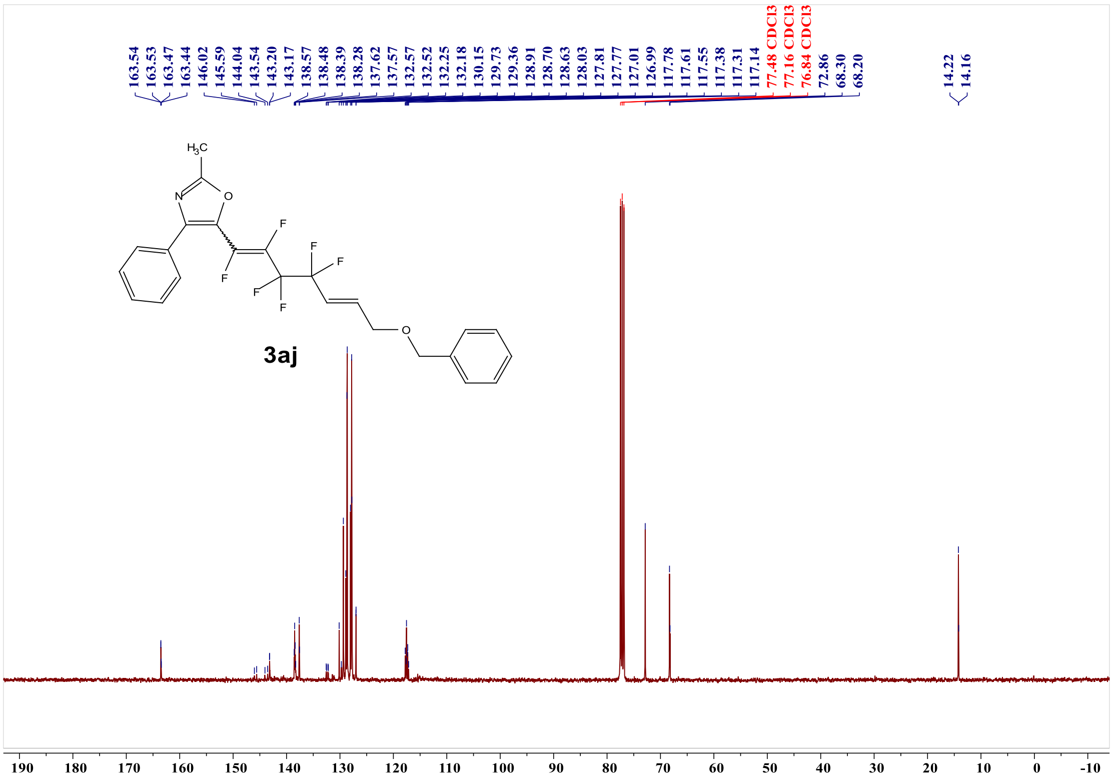

^{1}H NMR spectra of the product **3za** (400 MHz, $CDCl_3$)

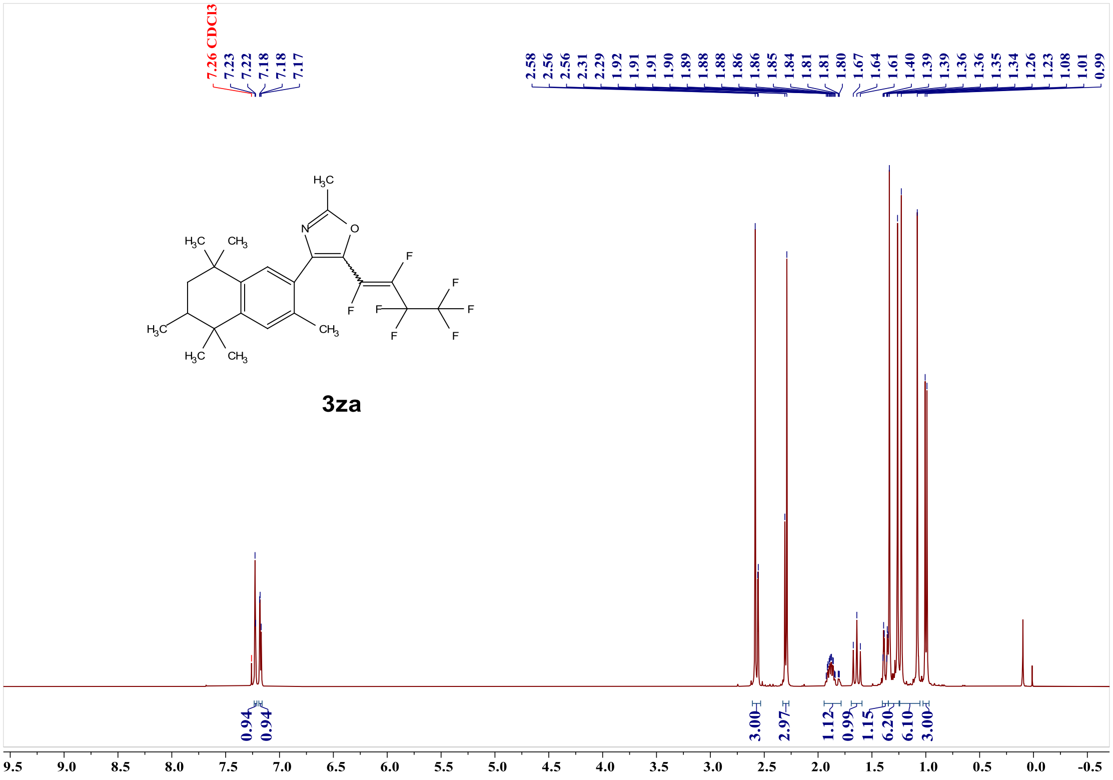

^{19}F NMR spectra of the product **3za** (376 MHz, $CDCl_3$)

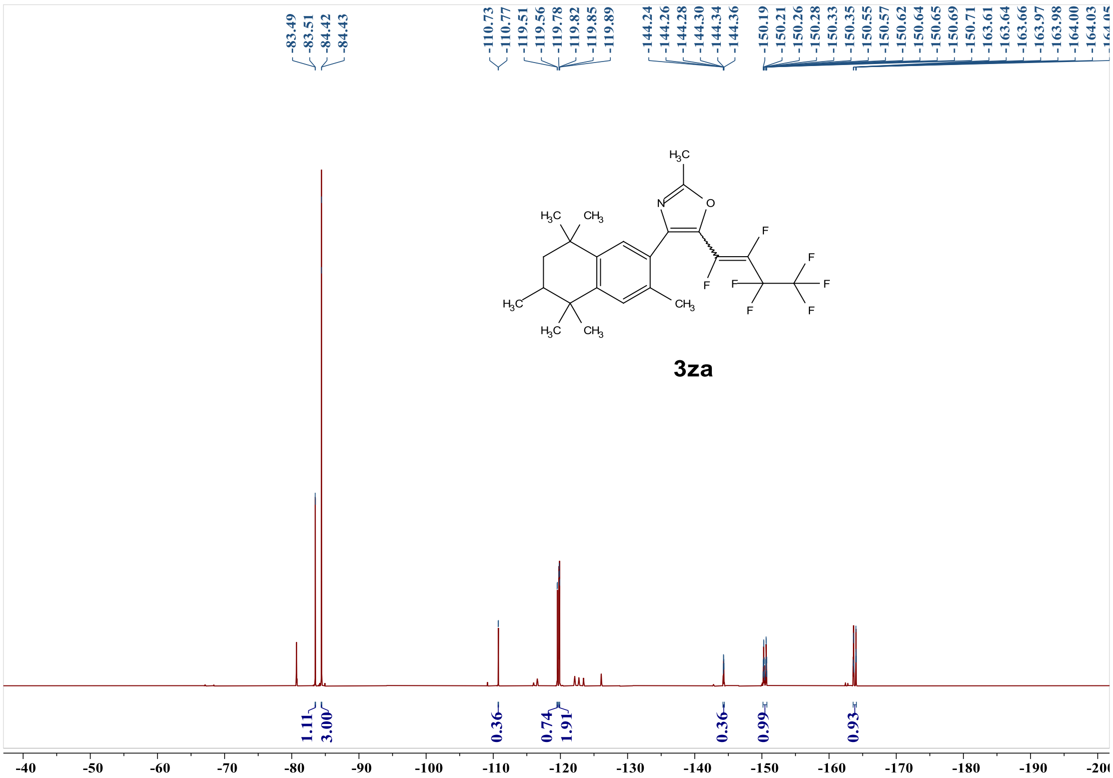

^{13}C NMR spectra of the product **3za** (100 MHz, $CDCl_3$)

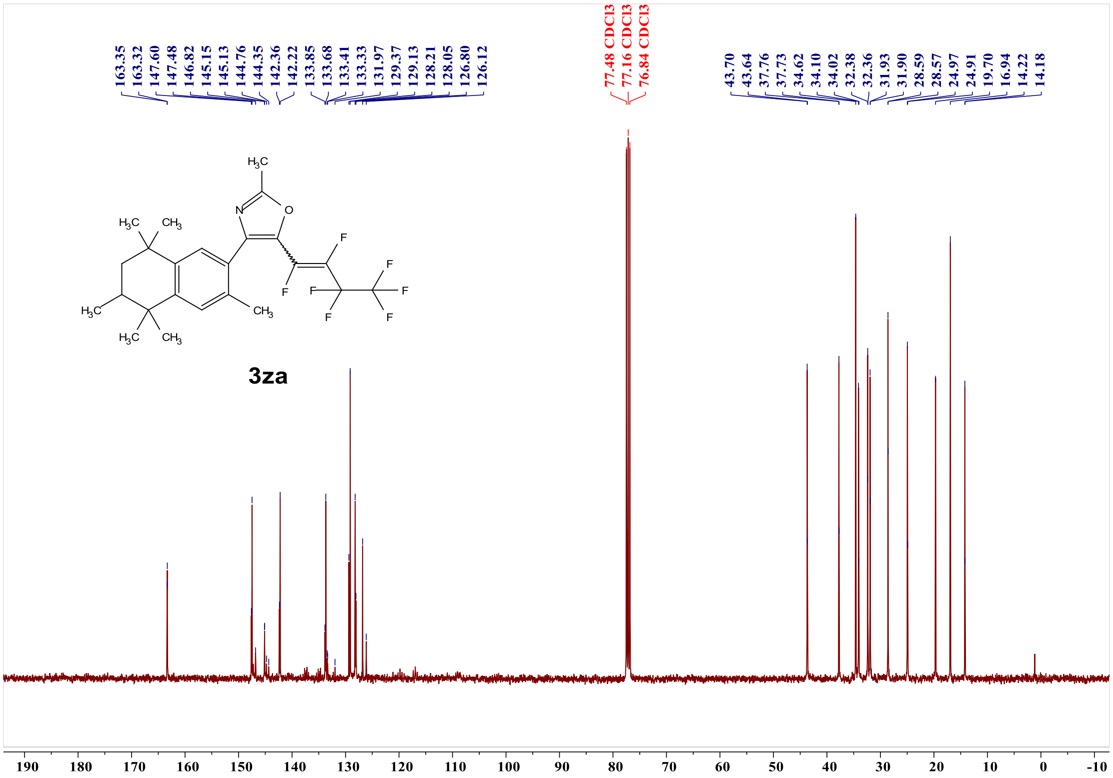

^{1}H NMR spectra of the product **3a’a** (400 MHz, $CDCl_3$)

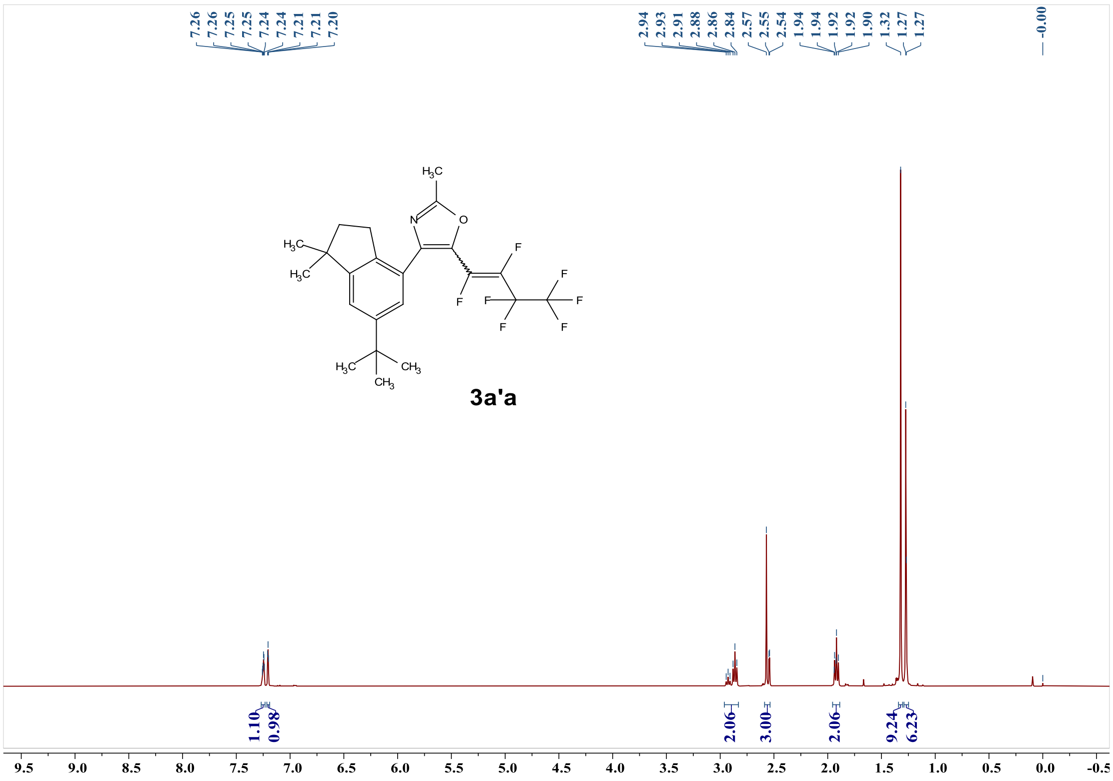

^{19}F NMR spectra of the product **3a'a** (376 MHz, $CDCl_3$)

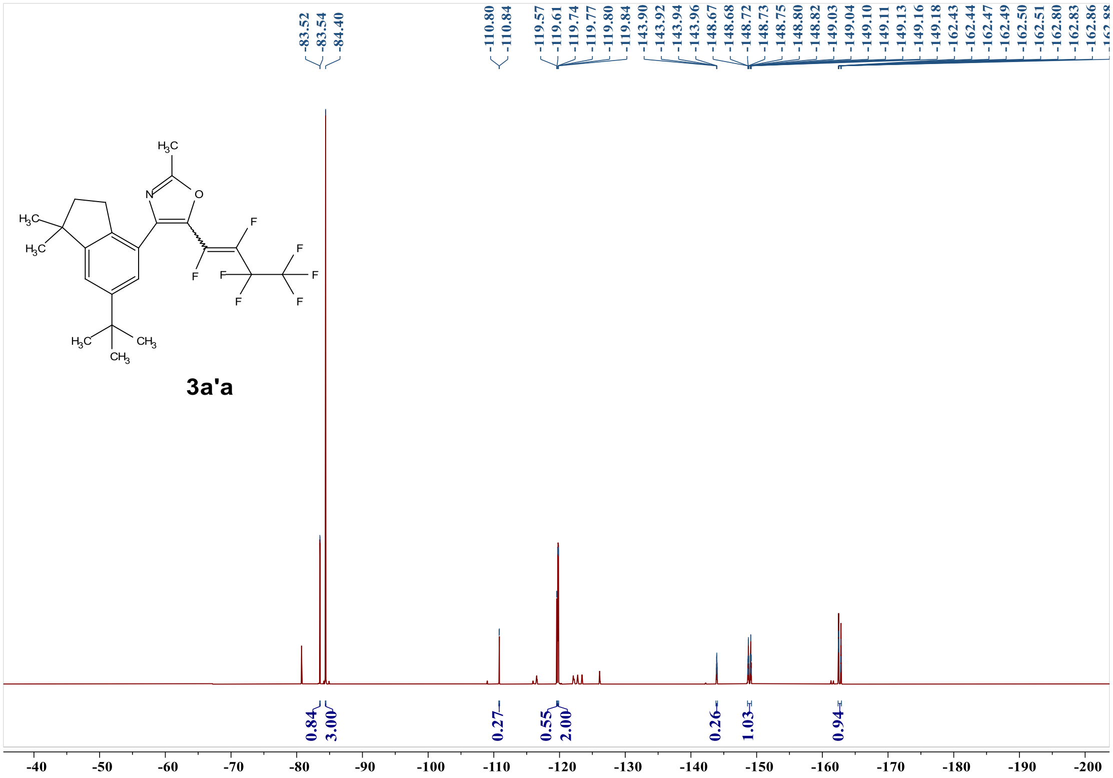

^{13}C NMR spectra of the product **3a'a** (100 MHz, $CDCl_3$)

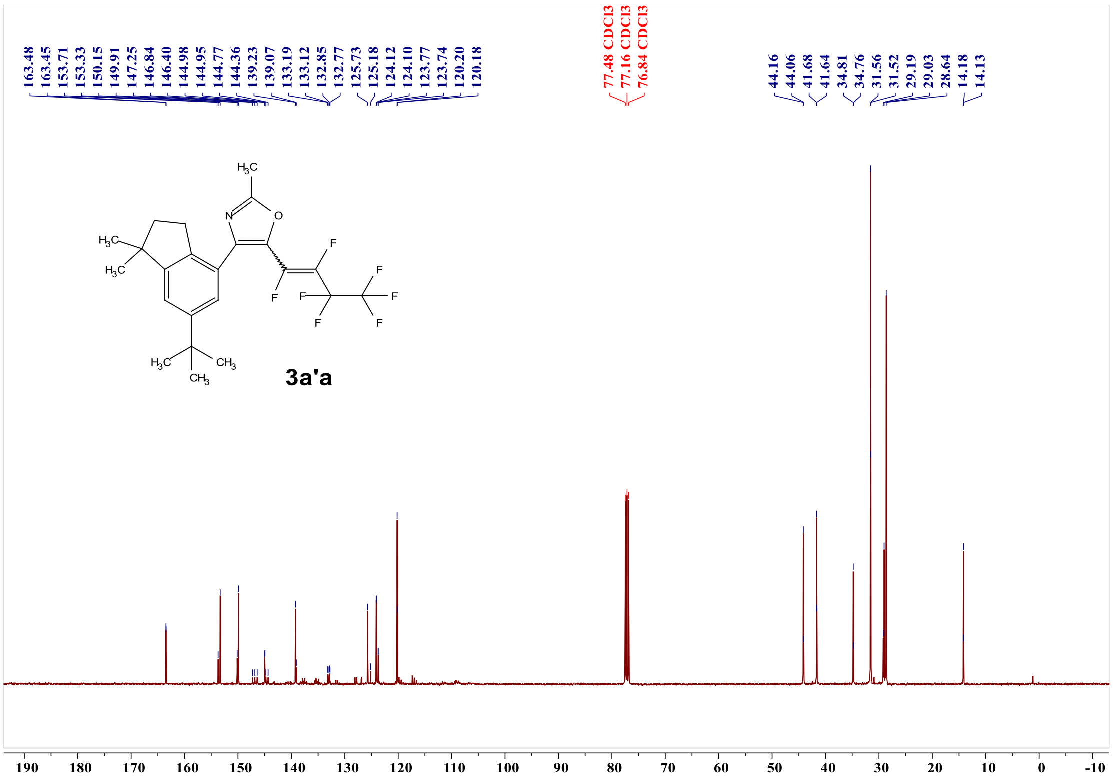

^{1}H NMR spectra of the product **3b’a** (400 MHz, $CDCl_3$)

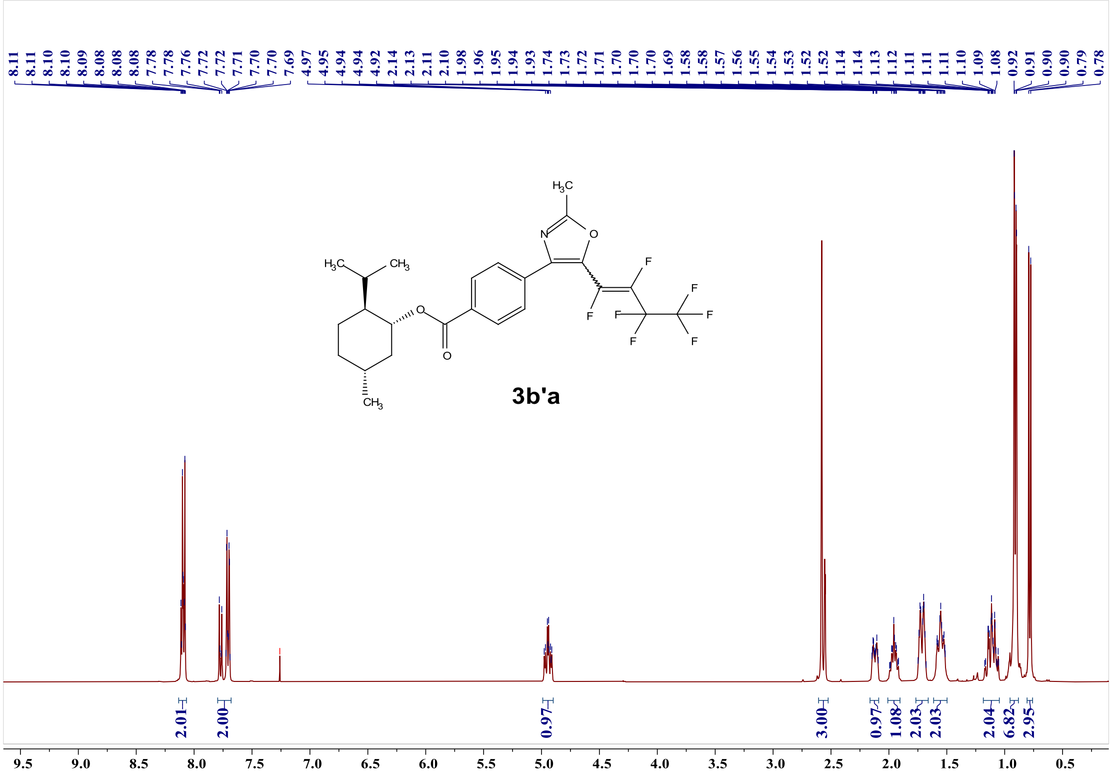

^{19}F NMR spectra of the product **3b’a** (376 MHz, $CDCl_3$)

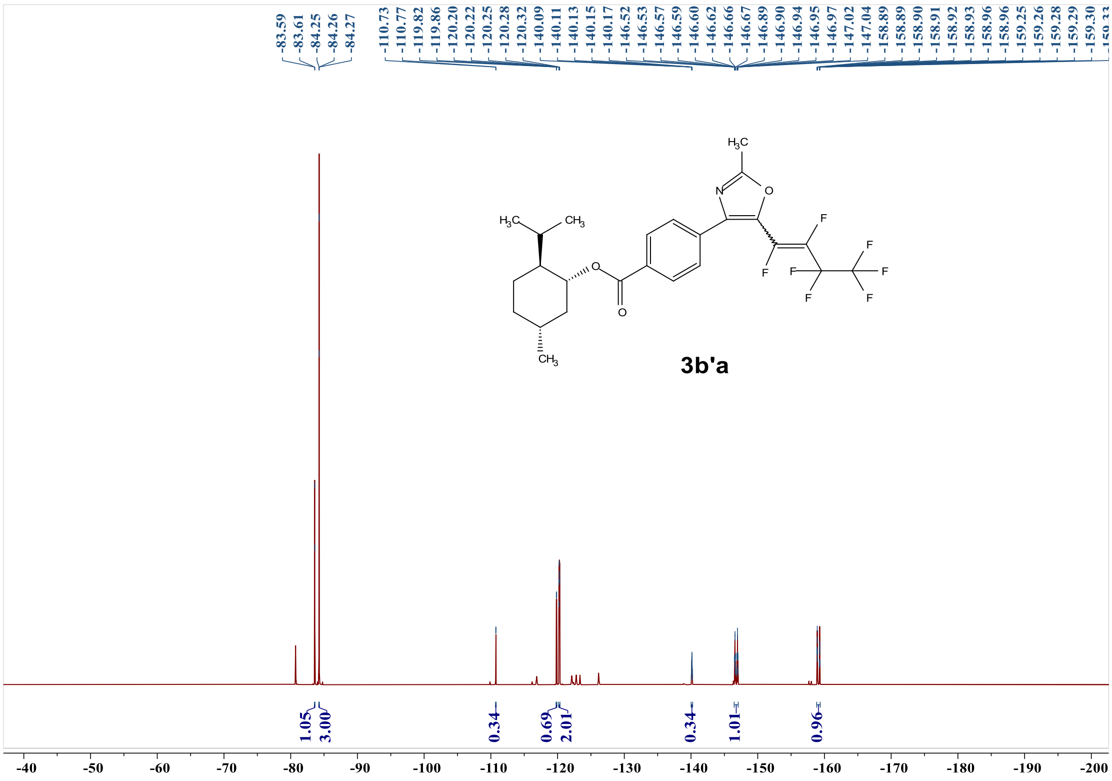

^{13}C NMR spectra of the product **3b'a** (100 MHz, $CDCl_3$)

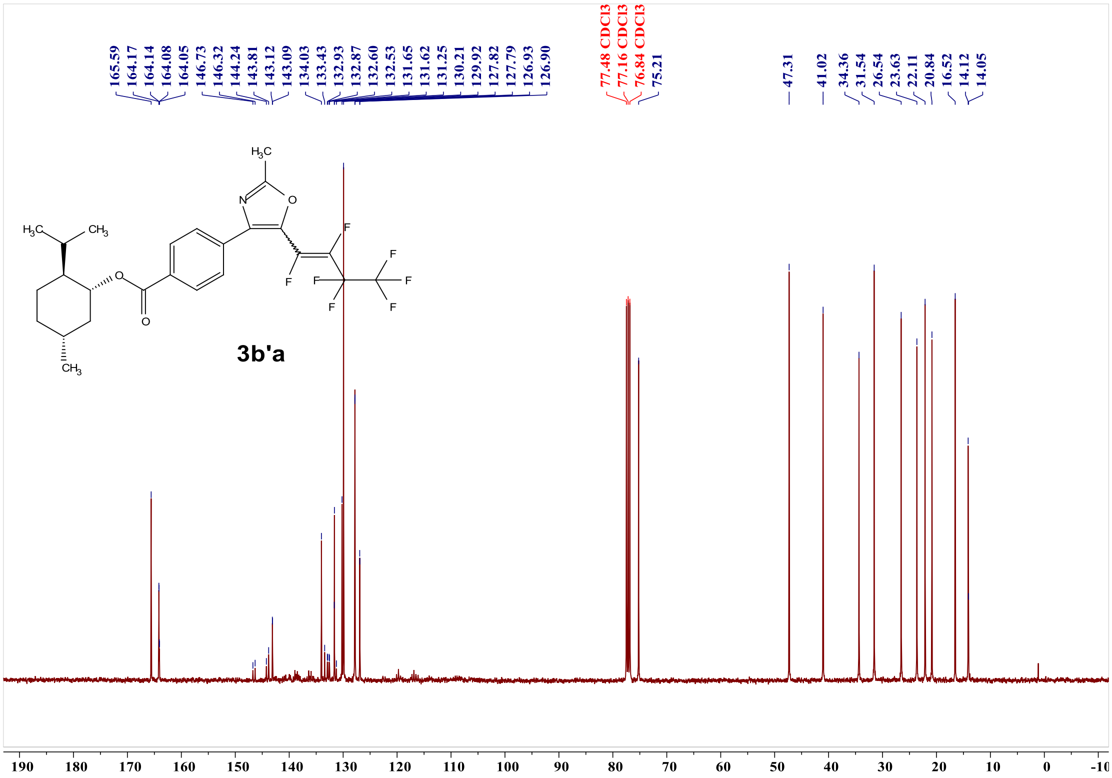

^{1}H NMR spectra of the product **3c'a** (400 MHz, $CDCl_3$)

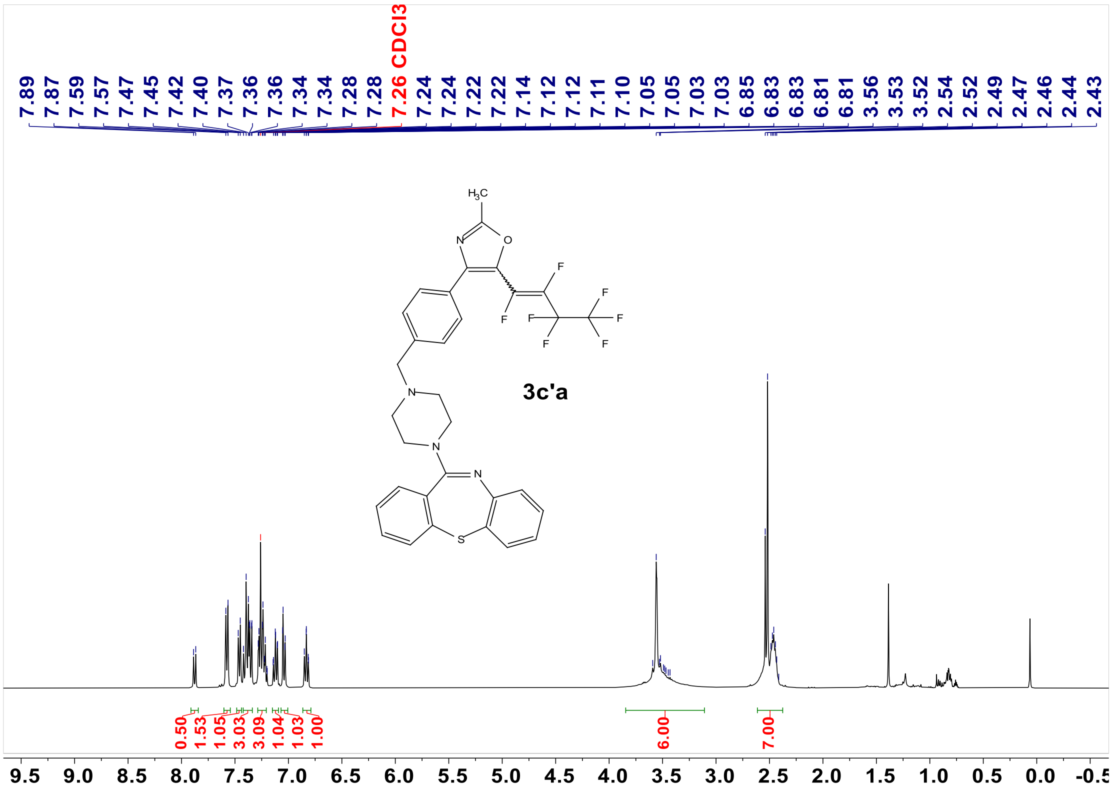

^{19}F NMR spectra of the product **3c’a** (376 MHz, $CDCl_3$)

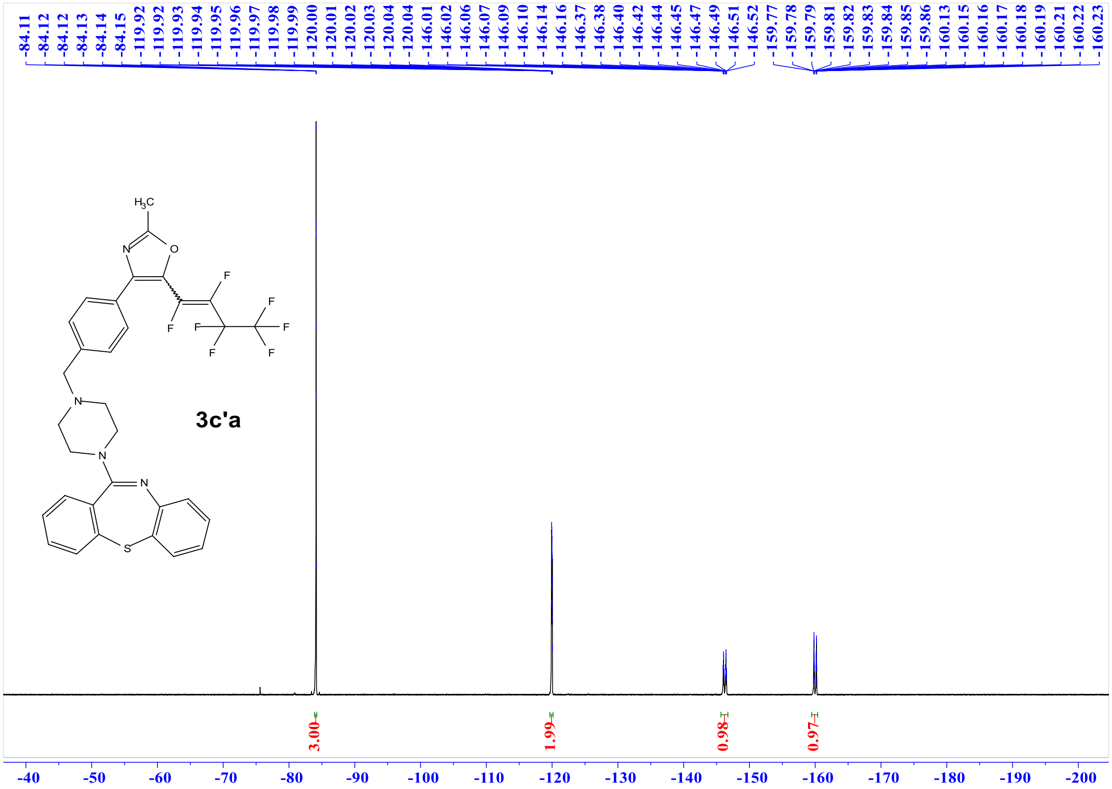

^{13}C NMR spectra of the product **3c’a** (100 MHz, $CDCl_3$)

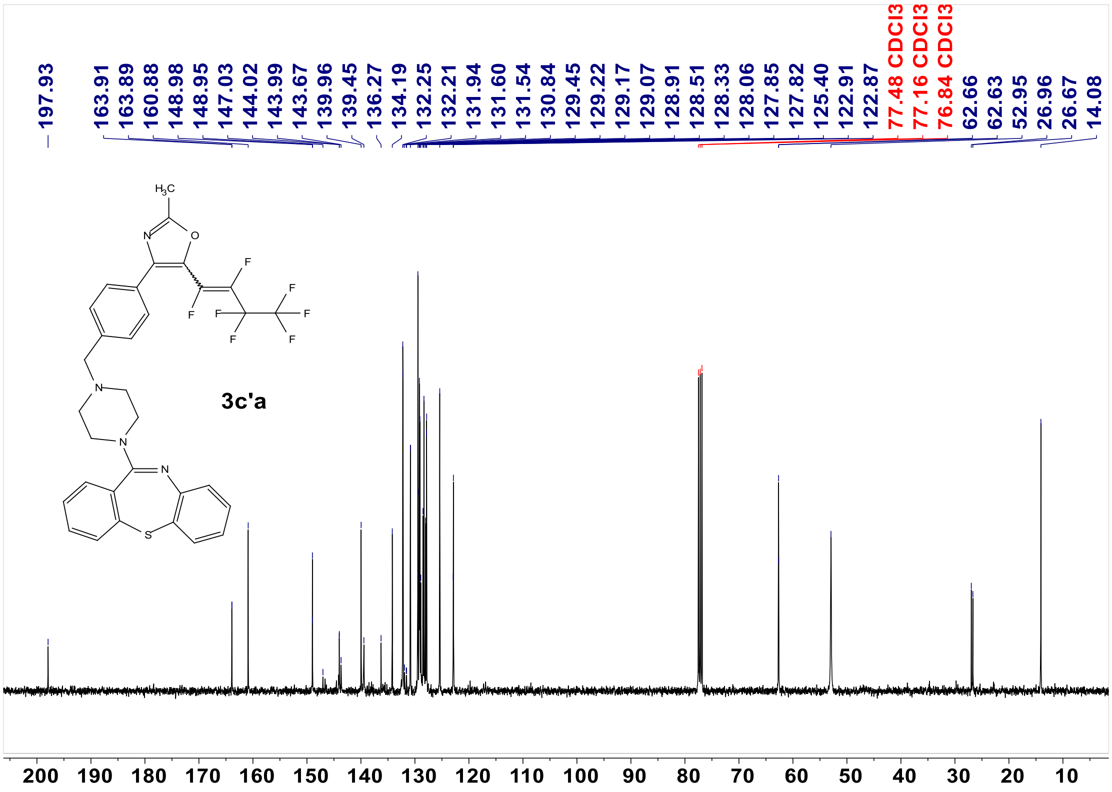

^{1}H NMR spectra of the product **3d'a** (400 MHz, $CDCl_3$)

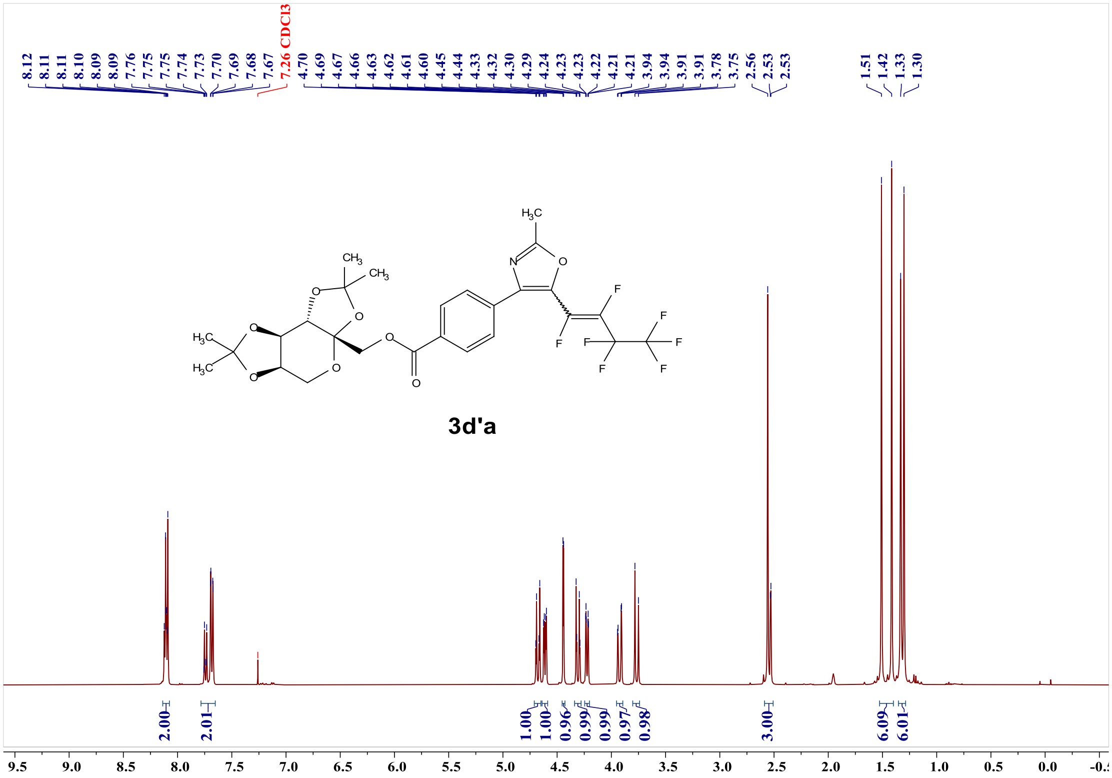

^{19}F NMR spectra of the product **3d'a** (376 MHz, $CDCl_3$)

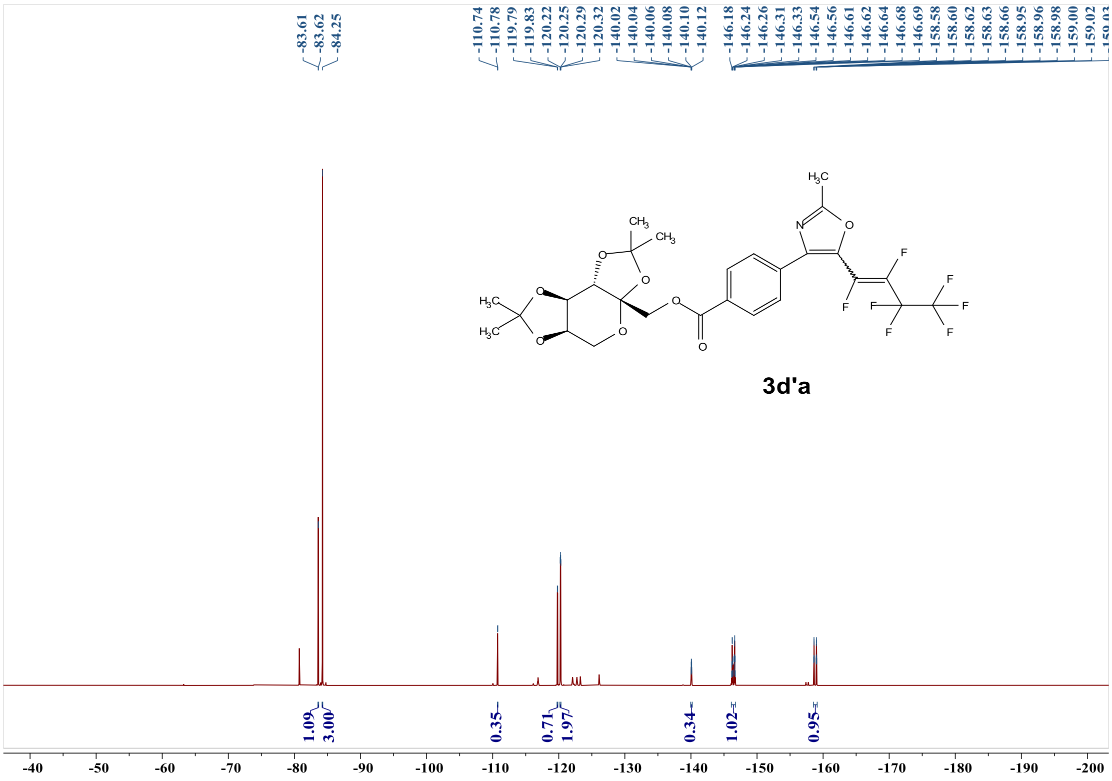

^{13}C NMR spectra of the product **3d'a** (100 MHz, $CDCl_3$)

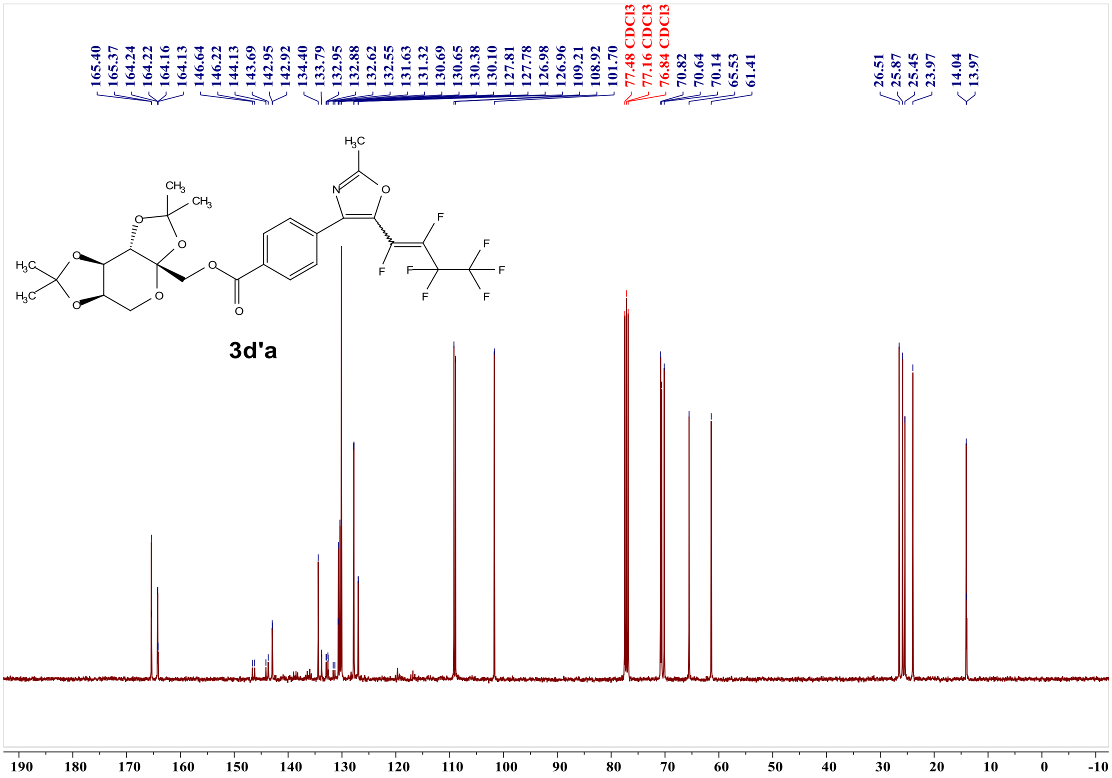

^{1}H NMR spectra of the product **3e'a** (400 MHz, $CDCl_3$)

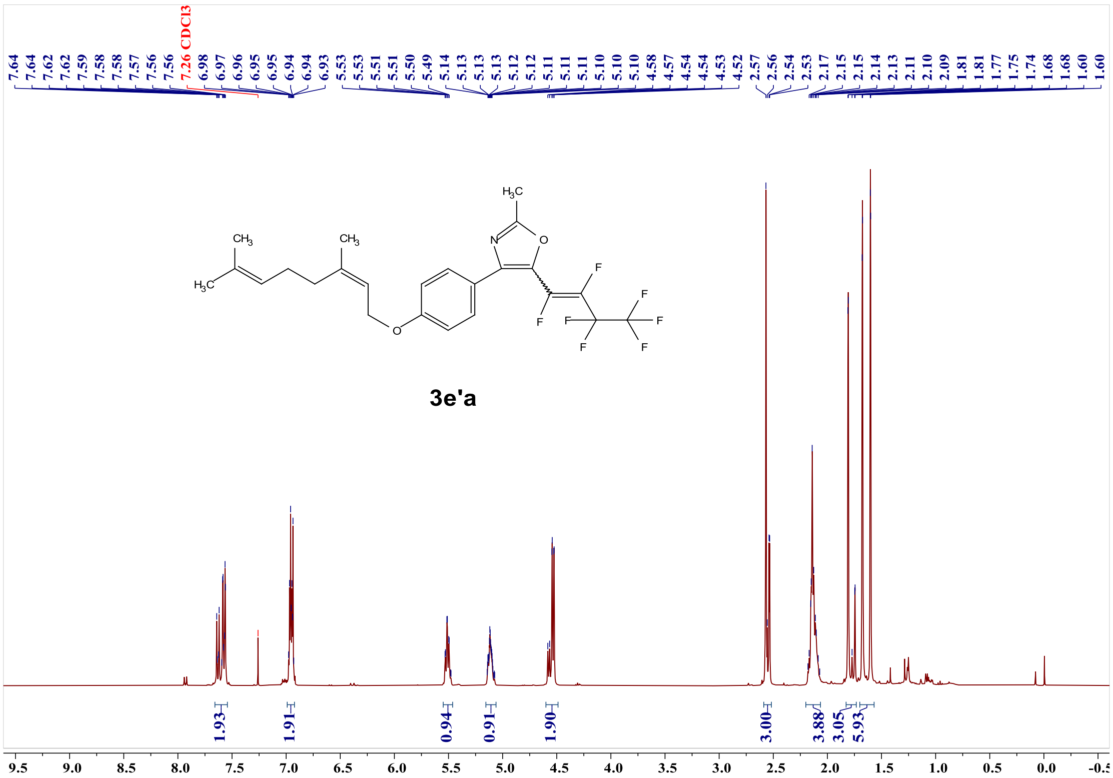

^{19}F NMR spectra of the product **3e’a** (376 MHz, $CDCl_3$)

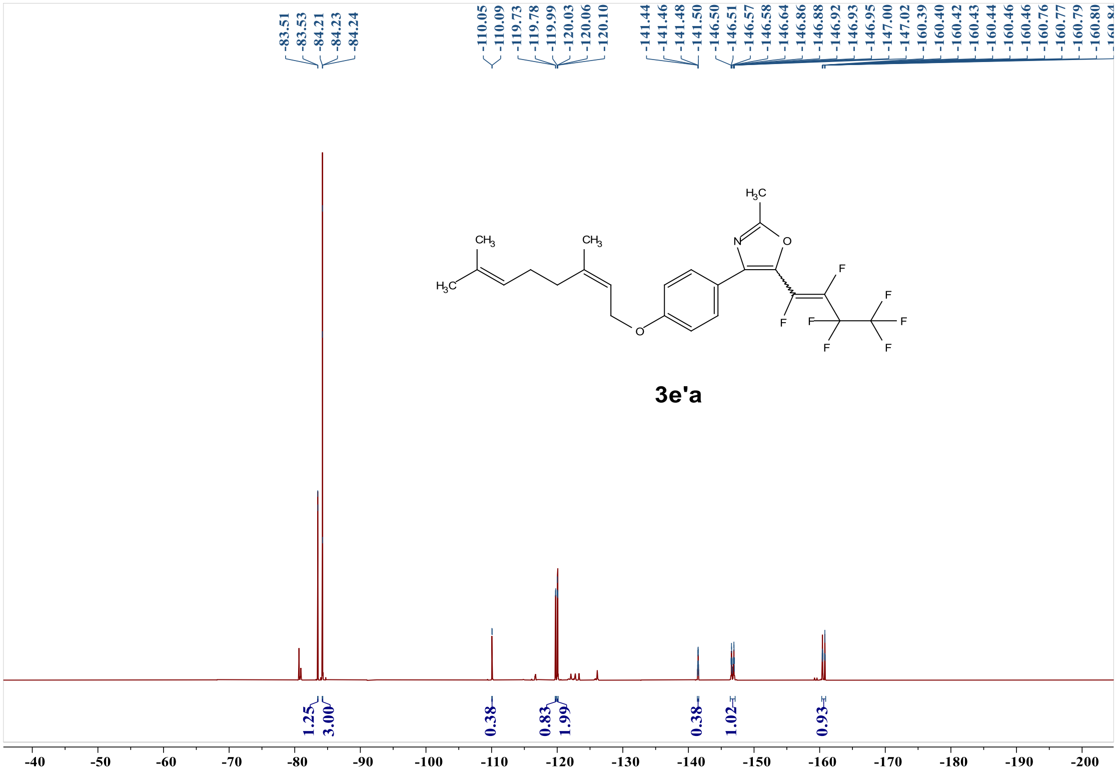

^{13}C NMR spectra of the product **3e’a** (100 MHz, $CDCl_3$)

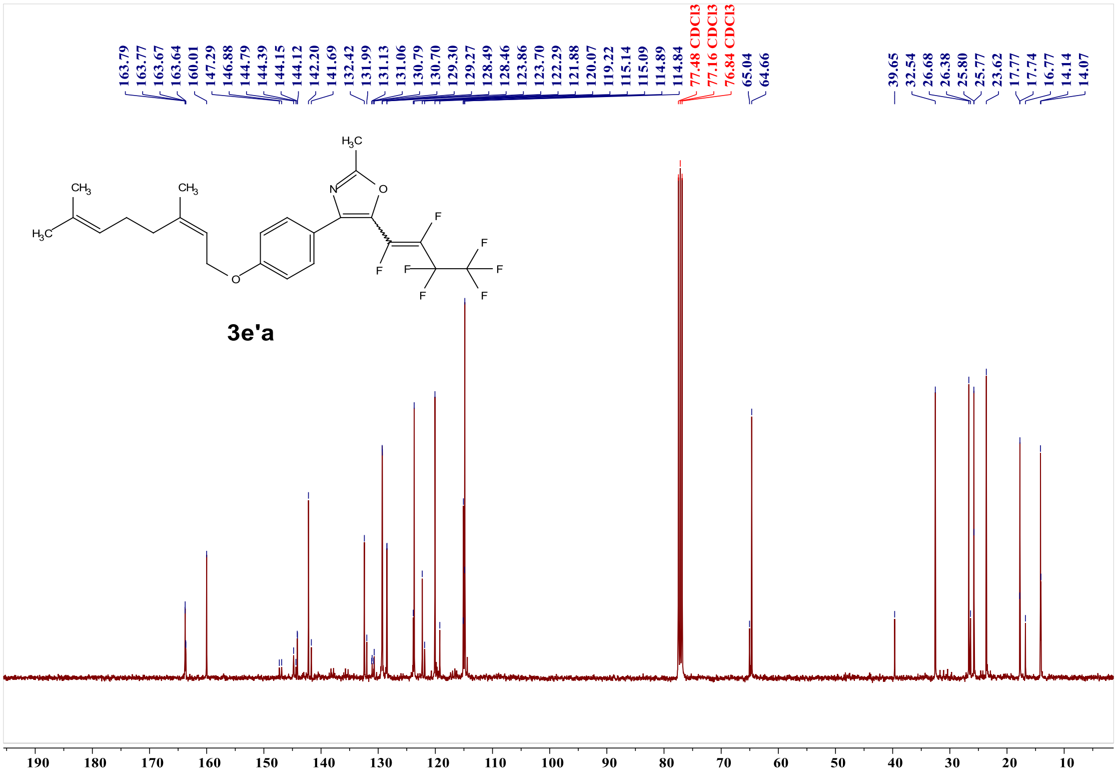

^{1}H NMR spectra of the product **3f'a** (400 MHz, $CDCl_3$)

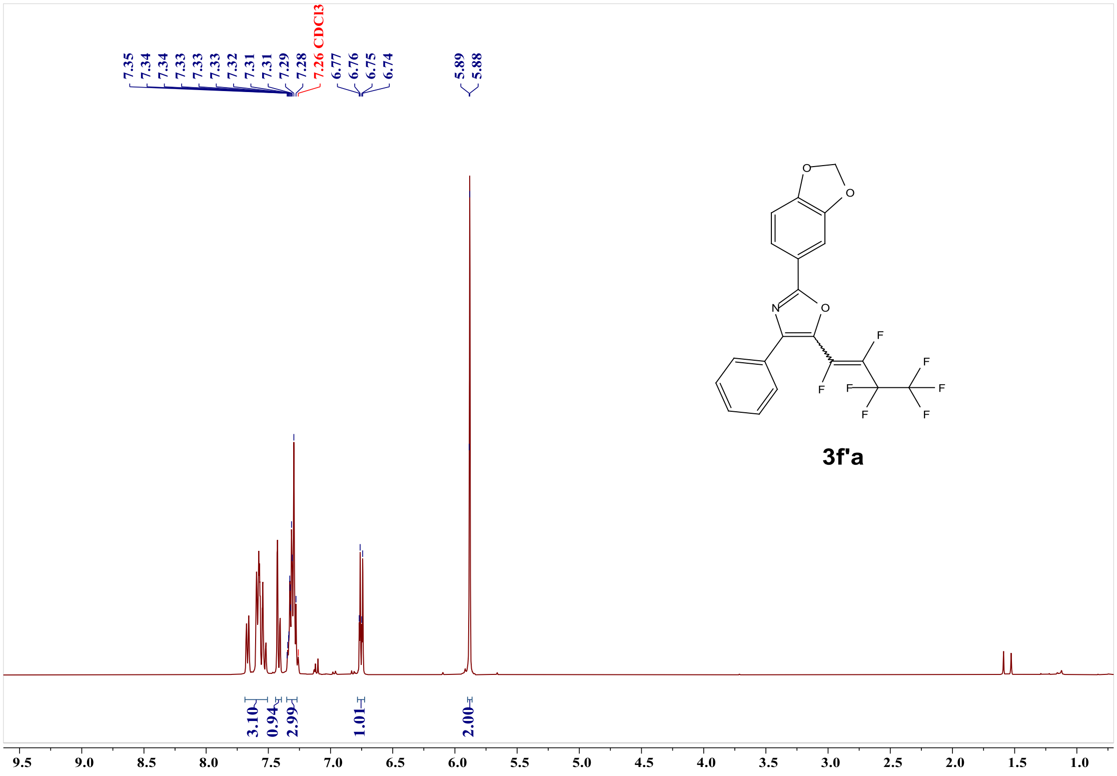

^{19}F NMR spectra of the product **3f'a** (376 MHz, $CDCl_3$)

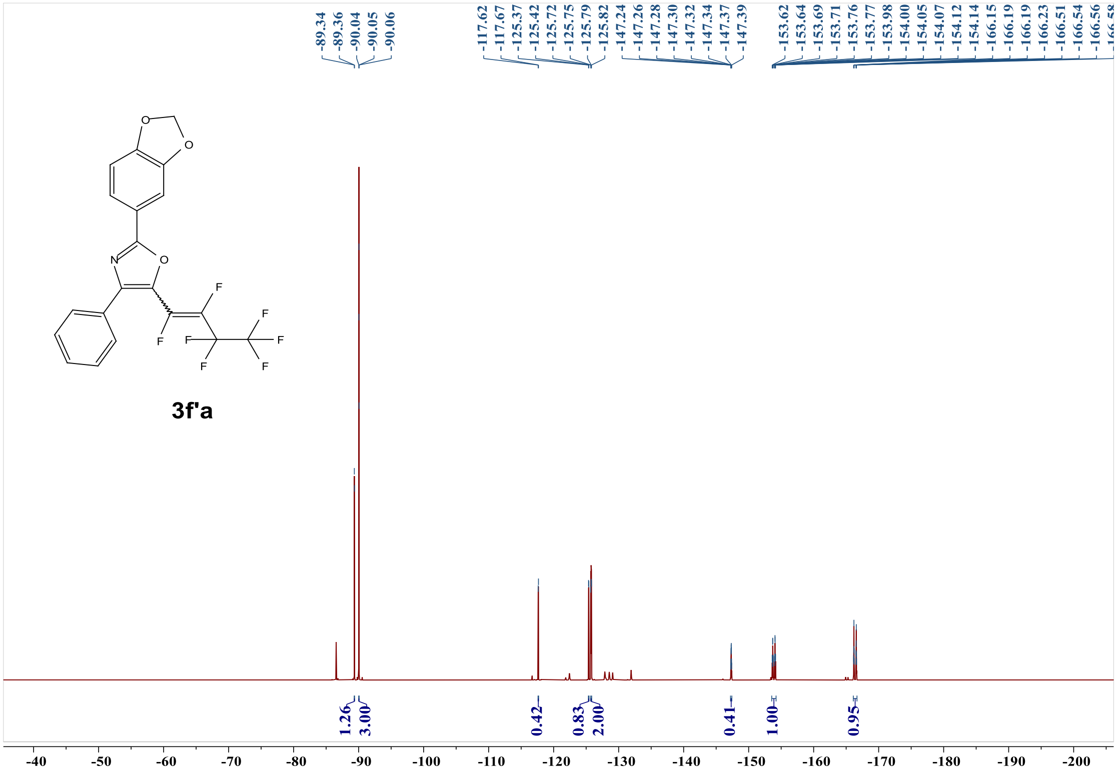

^{13}C NMR spectra of the product **3f'a** (100 MHz, $CDCl_3$)

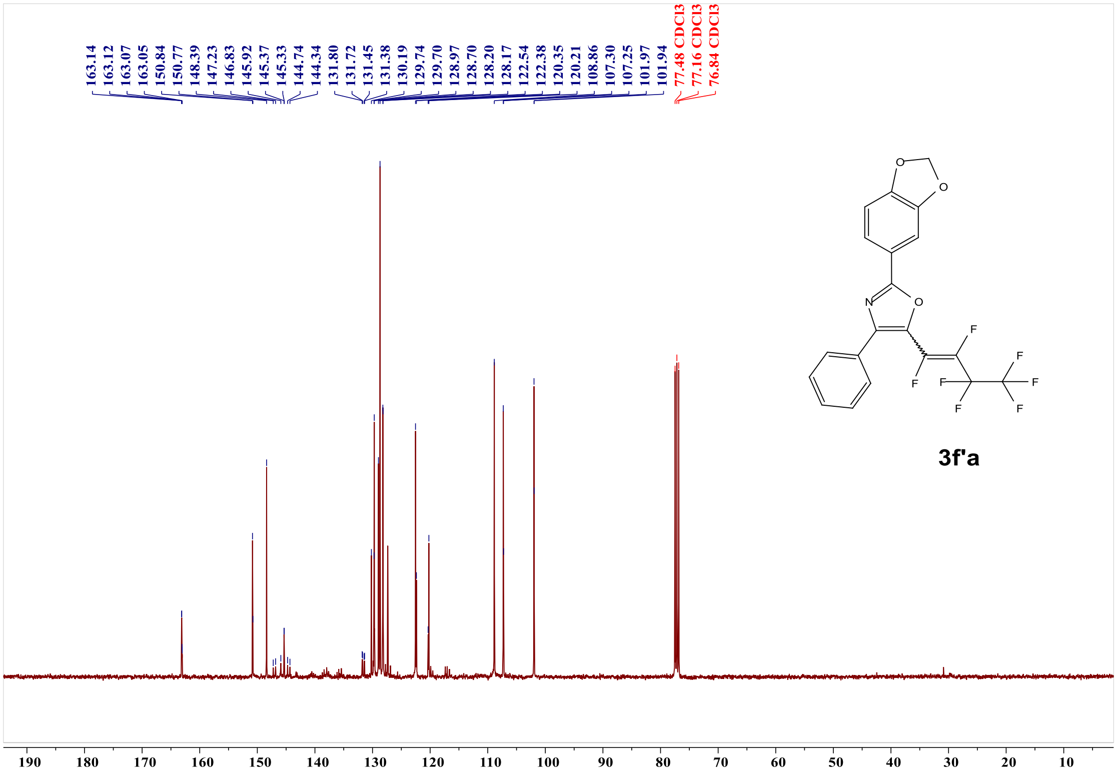

^{1}H NMR spectra of the product **3g'a** (400 MHz, $CDCl_3$)

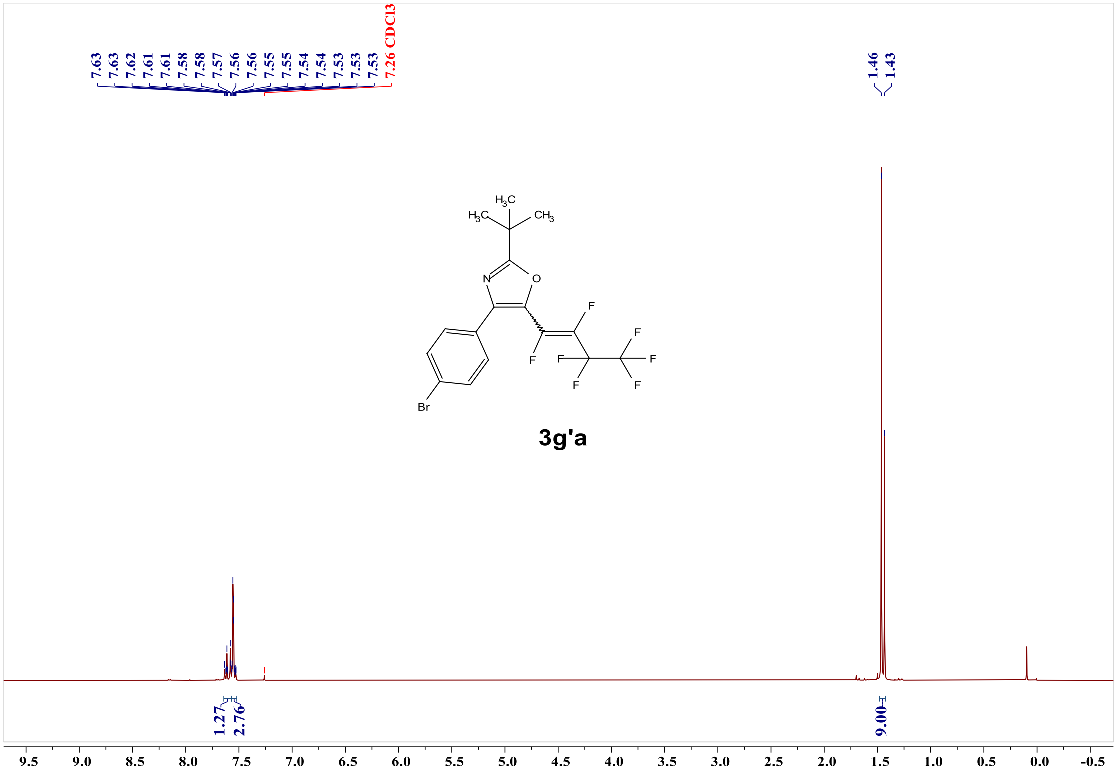

^{19}F NMR spectra of the product **3g'a** (376 MHz, $CDCl_3$)

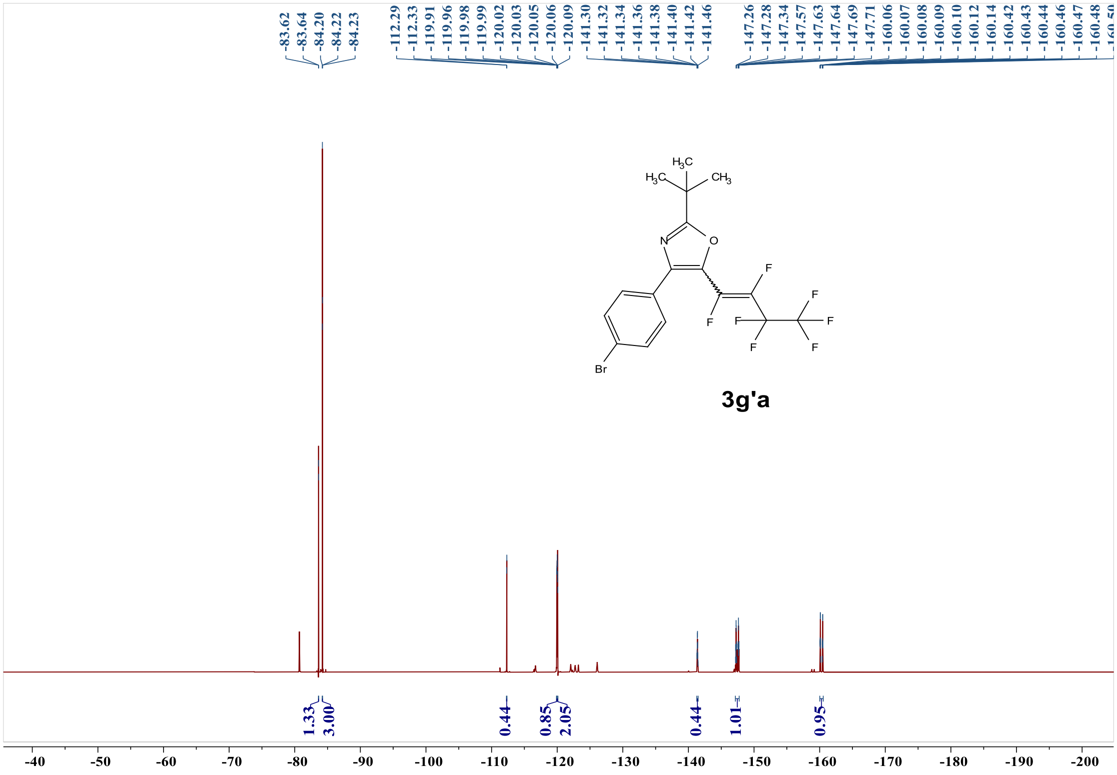

^{13}C NMR spectra of the product **3g'a** (100 MHz, $CDCl_3$)

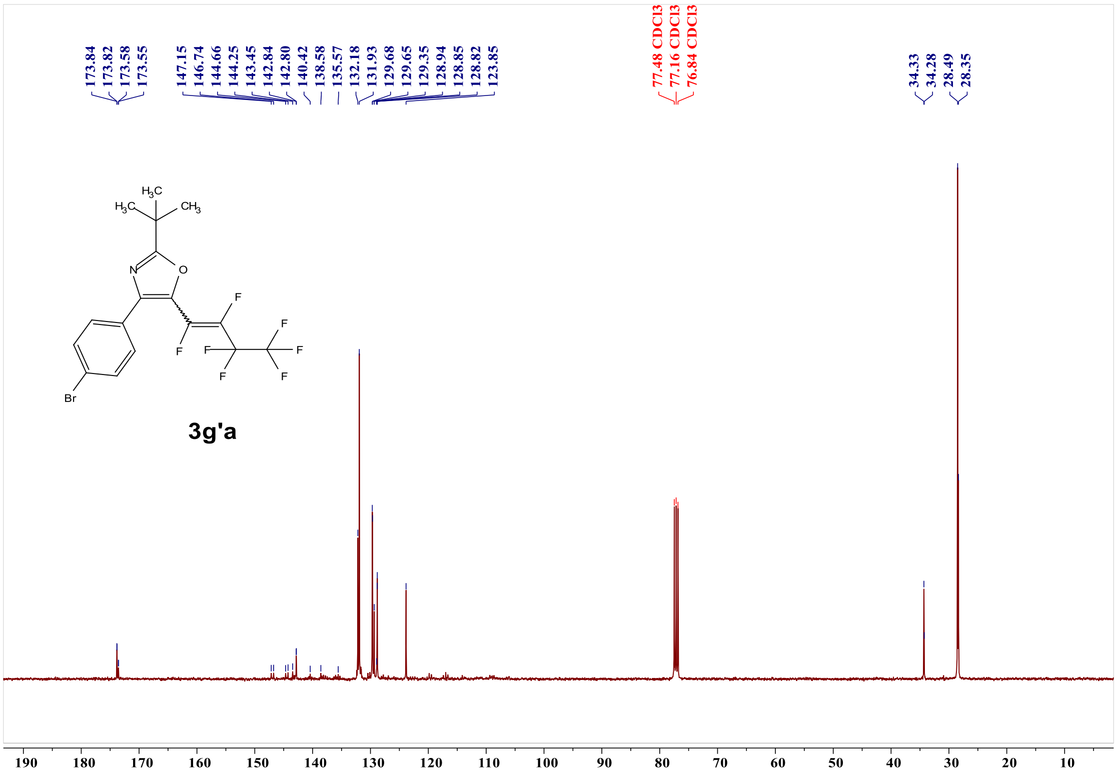

^{1}H NMR spectra of the product **3h'a** (400 MHz, $CDCl_3$)

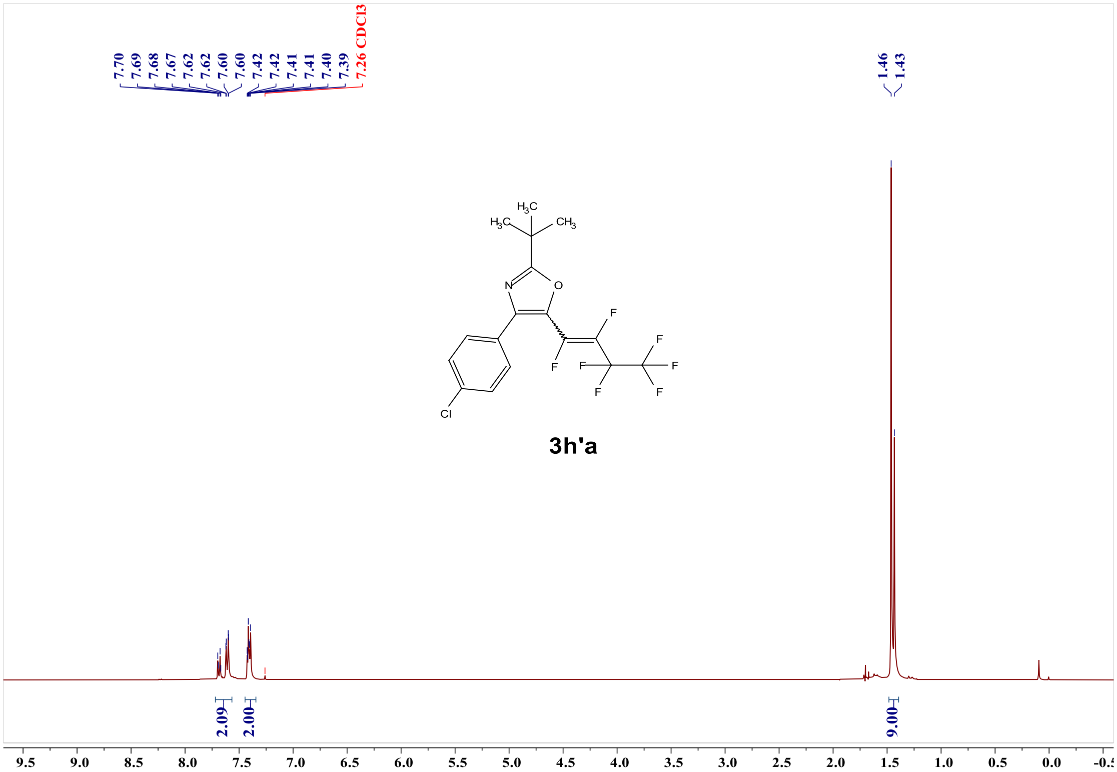

^{19}F NMR spectra of the product **3h'a** (376 MHz, $CDCl_3$)

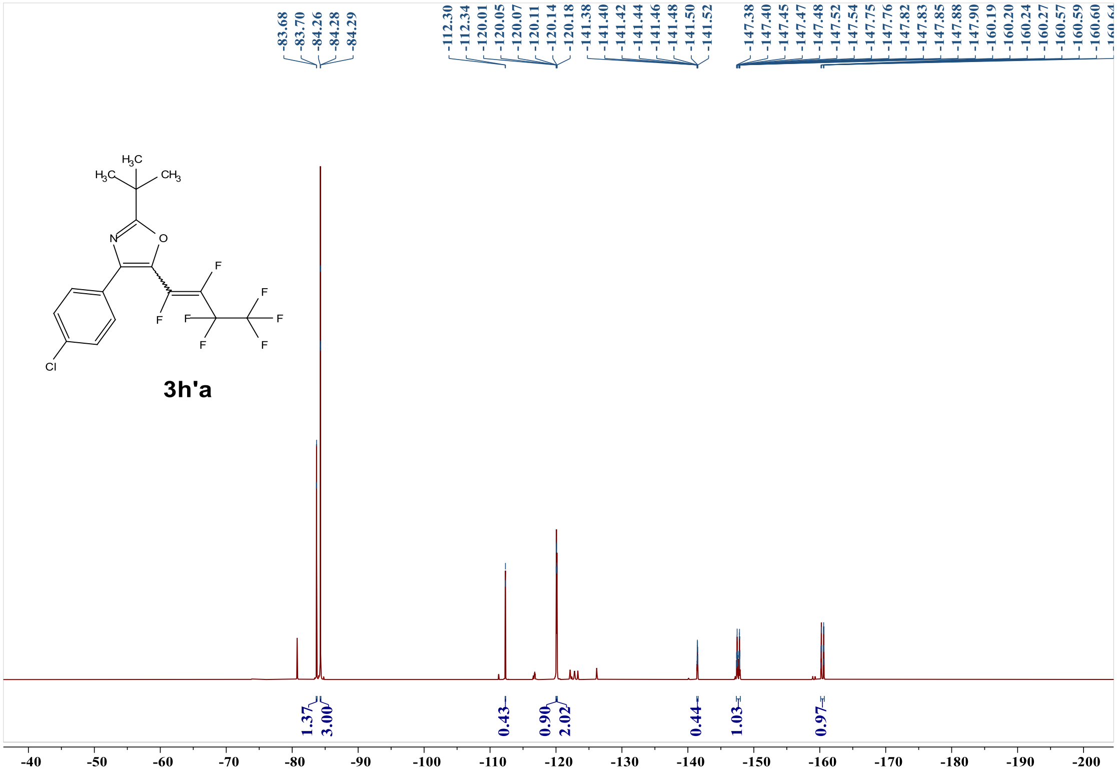

^{13}C NMR spectra of the product **3h'a** (100 MHz, CDCl$_3$)

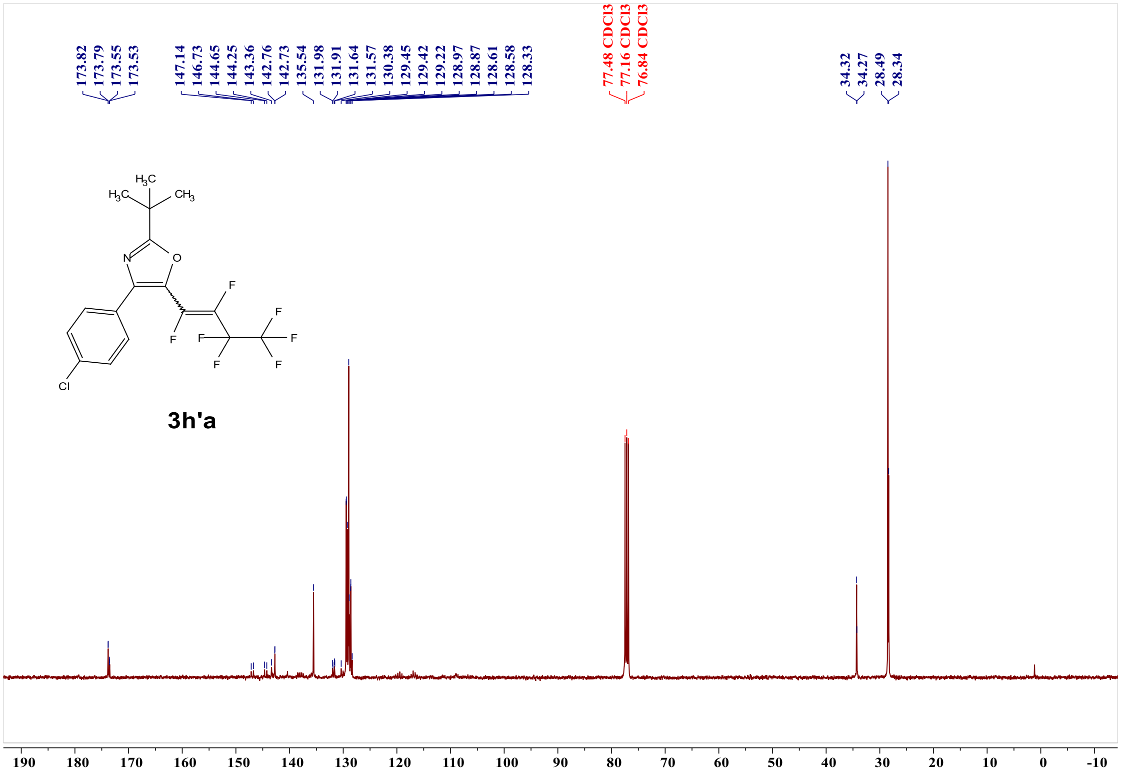

^{1}H NMR spectra of the product **5** (400 MHz, CDCl$_3$)

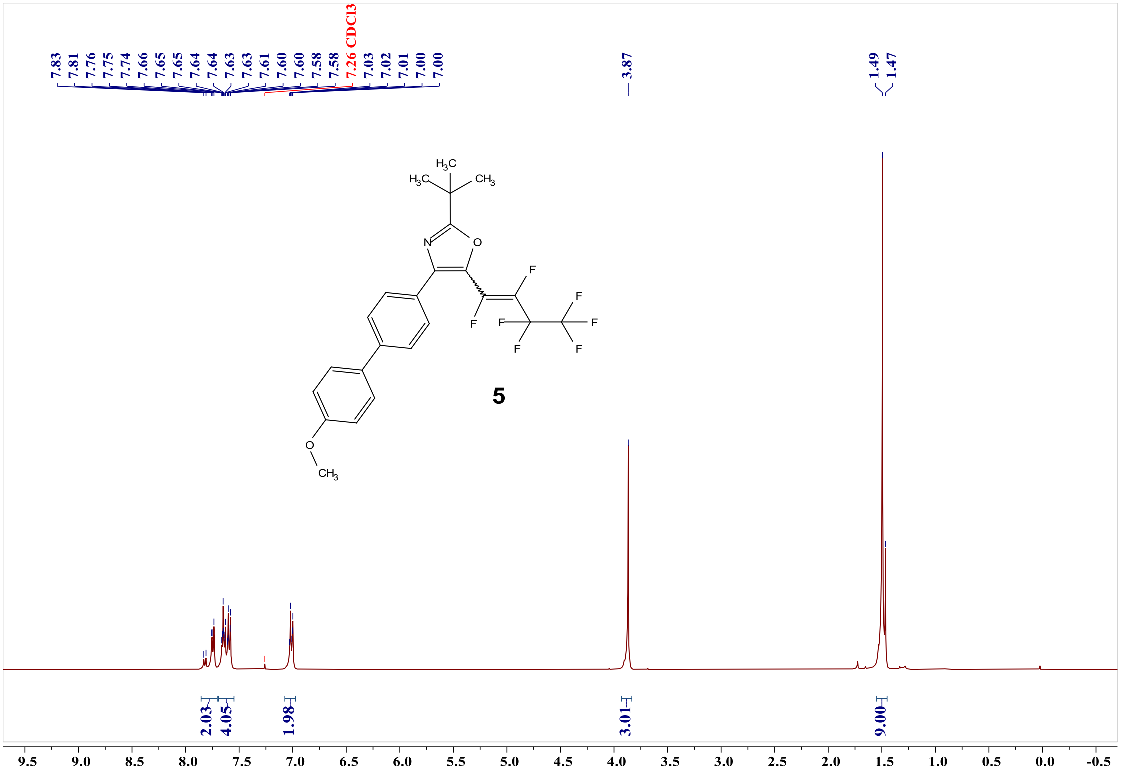

^{19}F NMR spectra of the product **5** (376 MHz, $CDCl_3$)

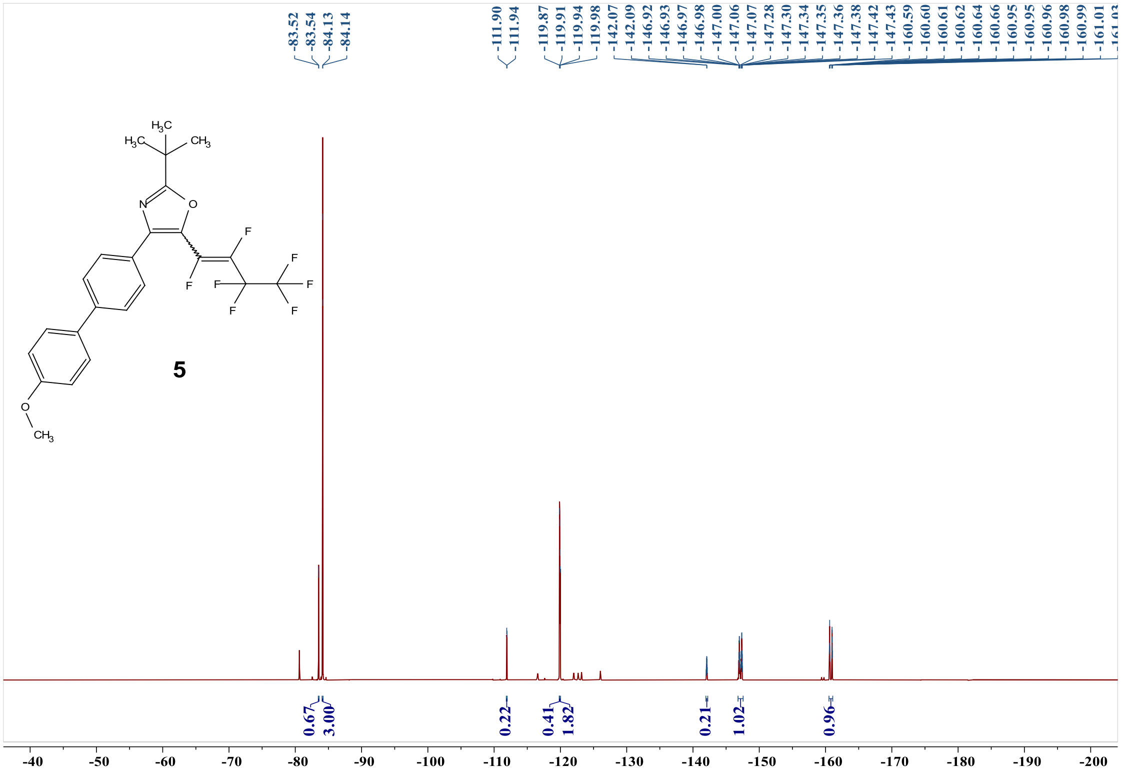

^{13}C NMR spectra of the product **5** (100 MHz, $CDCl_3$)

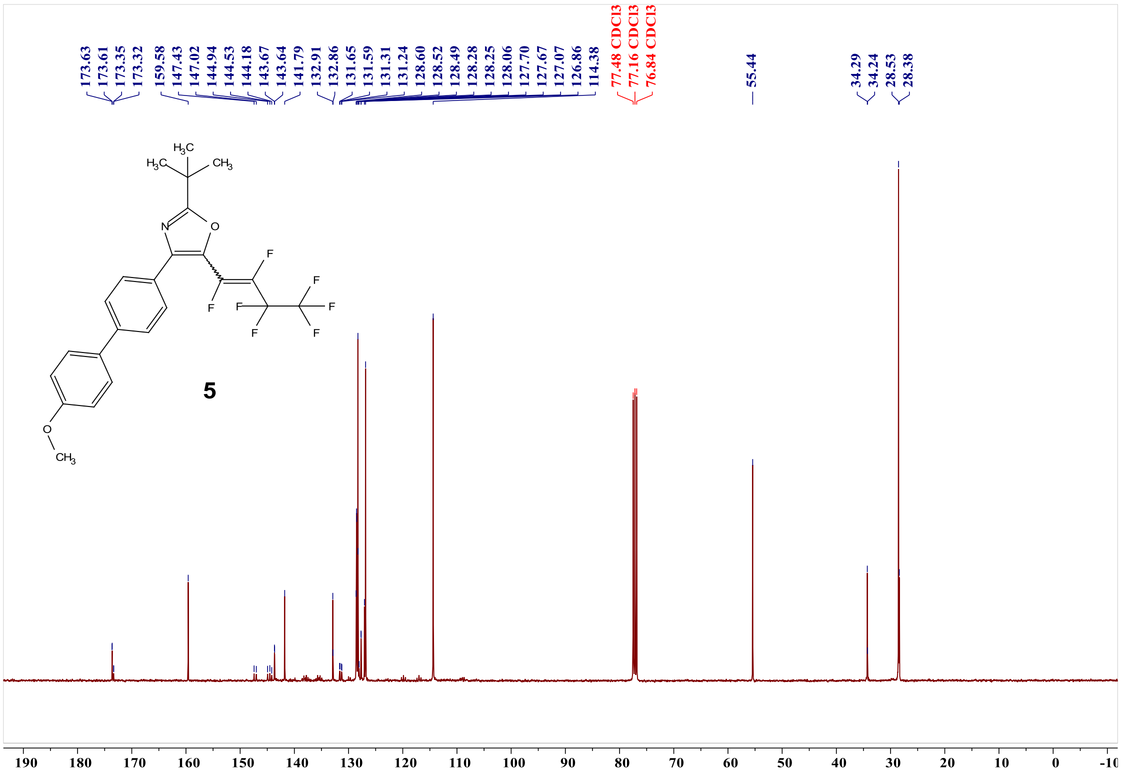

^{1}H NMR spectra of the product **7** (400 MHz, $CDCl_3$)

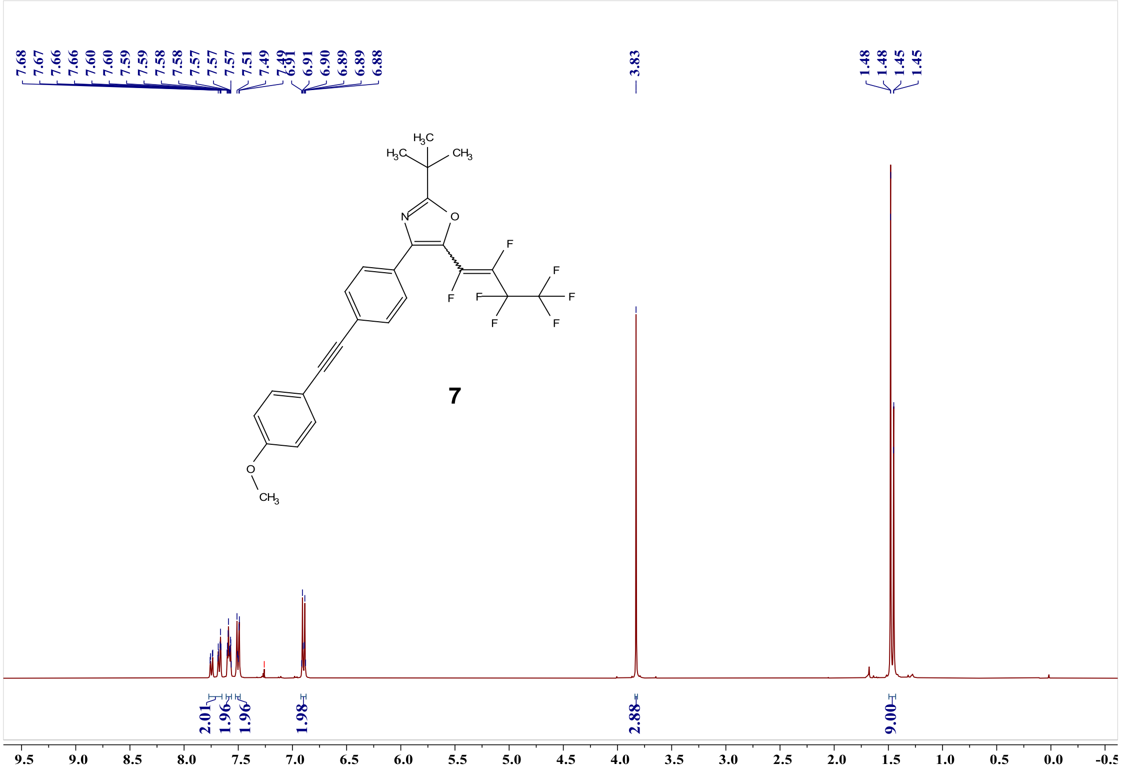

^{19}F NMR spectra of the product **7** (376 MHz, $CDCl_3$)

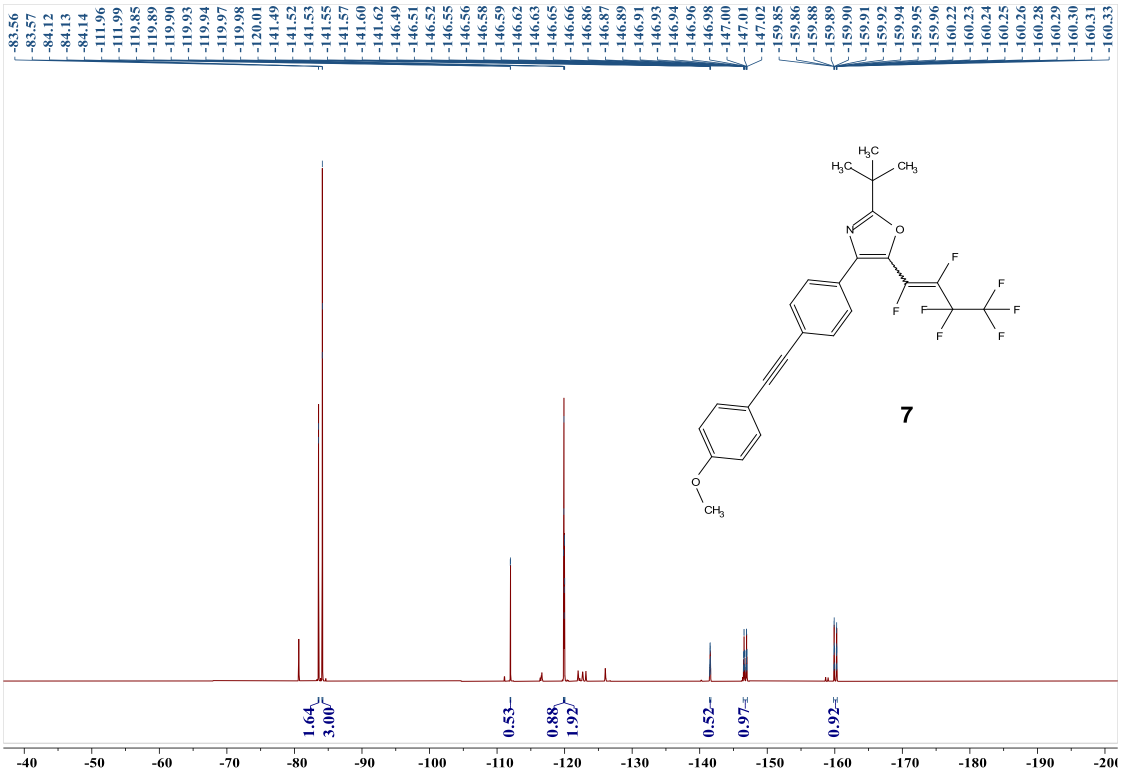

^{13}C NMR spectra of the product **7** (100 MHz, $CDCl_3$)

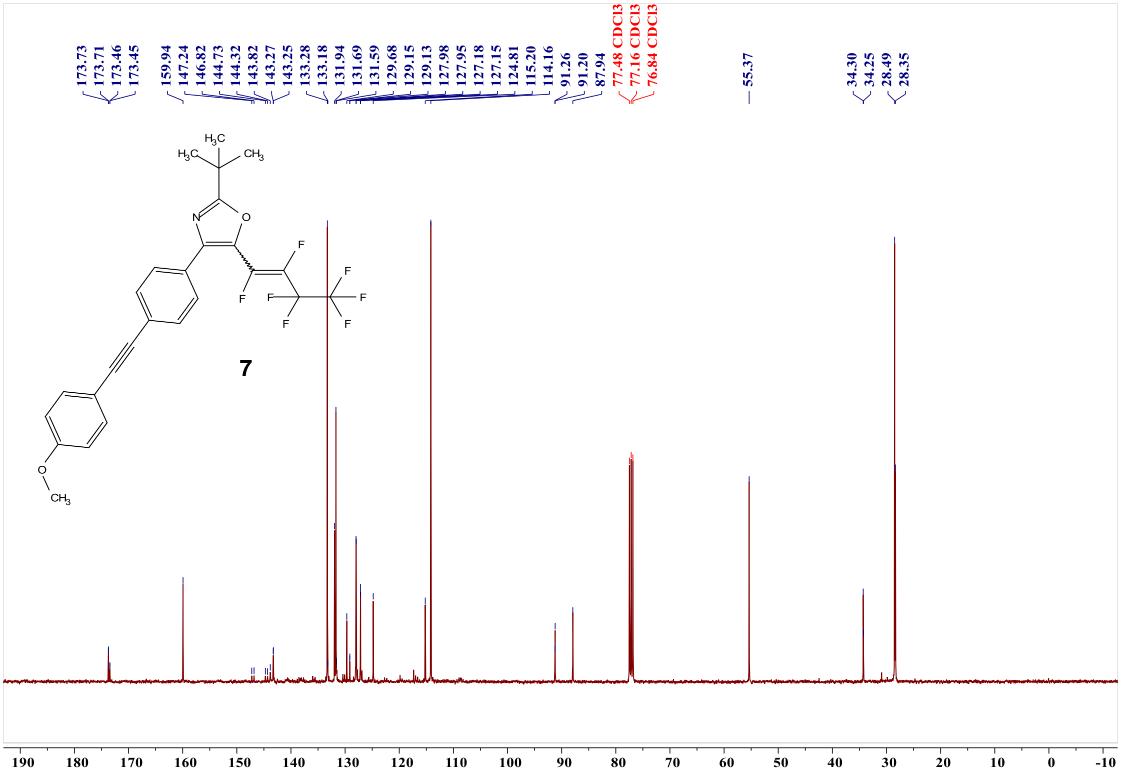

^{1}H NMR spectra of the product **9** (400 MHz, $CDCl_3$)

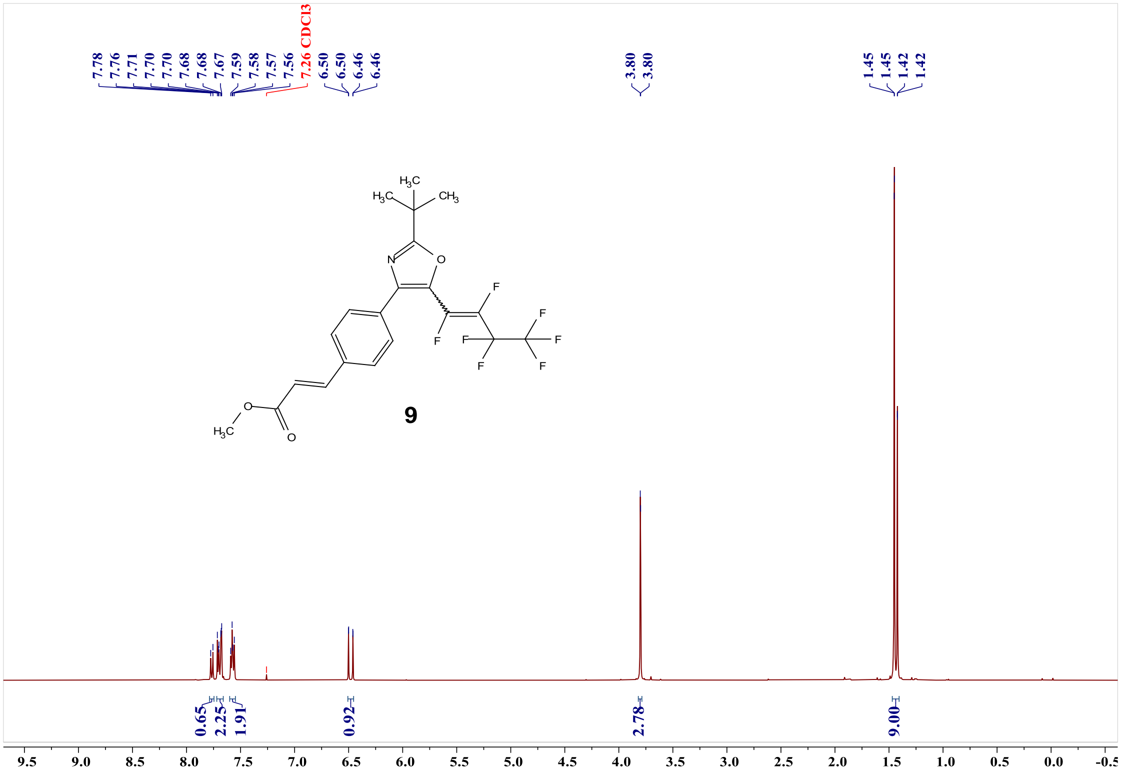

^{19}F NMR spectra of the product **9** (376 MHz, $CDCl_3$)

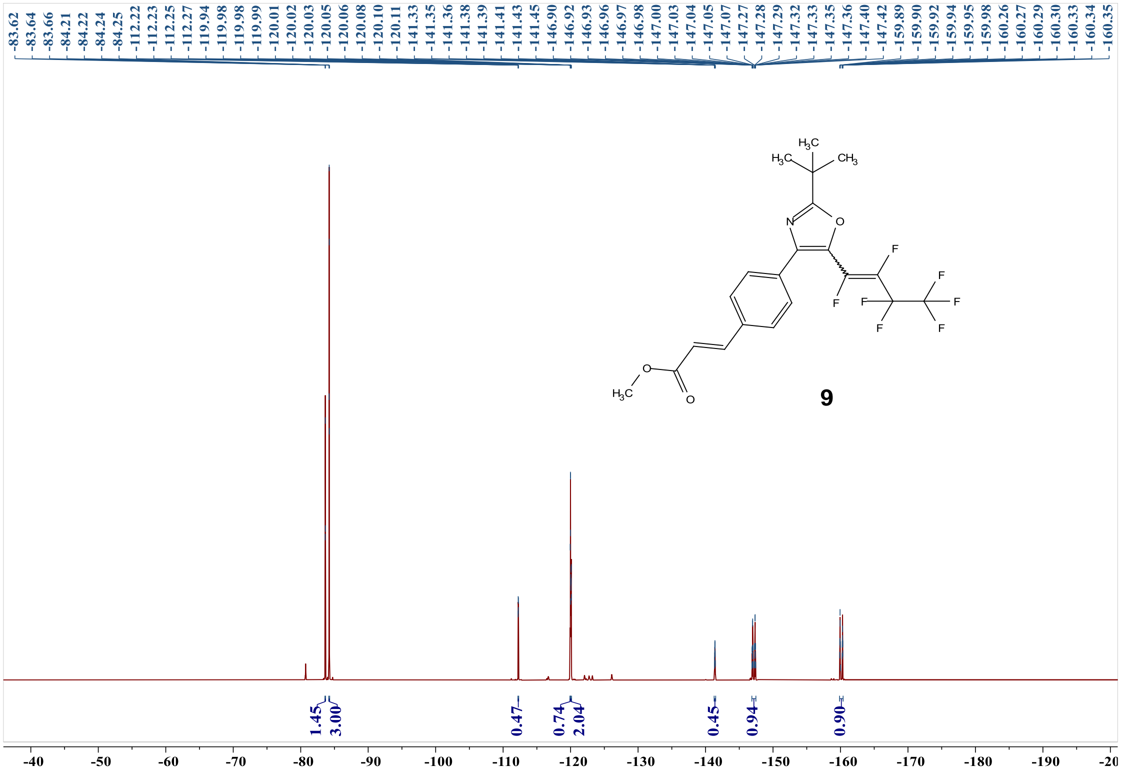

^{13}C NMR spectra of the product **9** (100 MHz, $CDCl_3$)

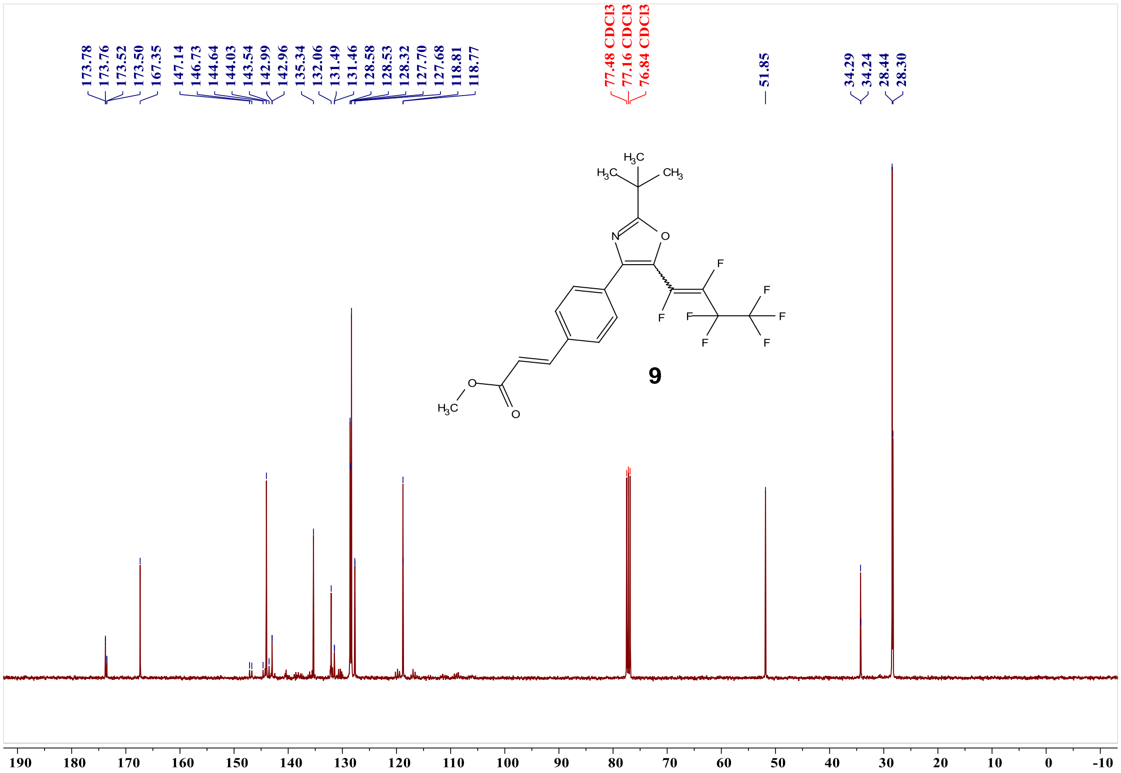

^{1}H NMR spectra of the product **11** (400 MHz, $CDCl_3$)

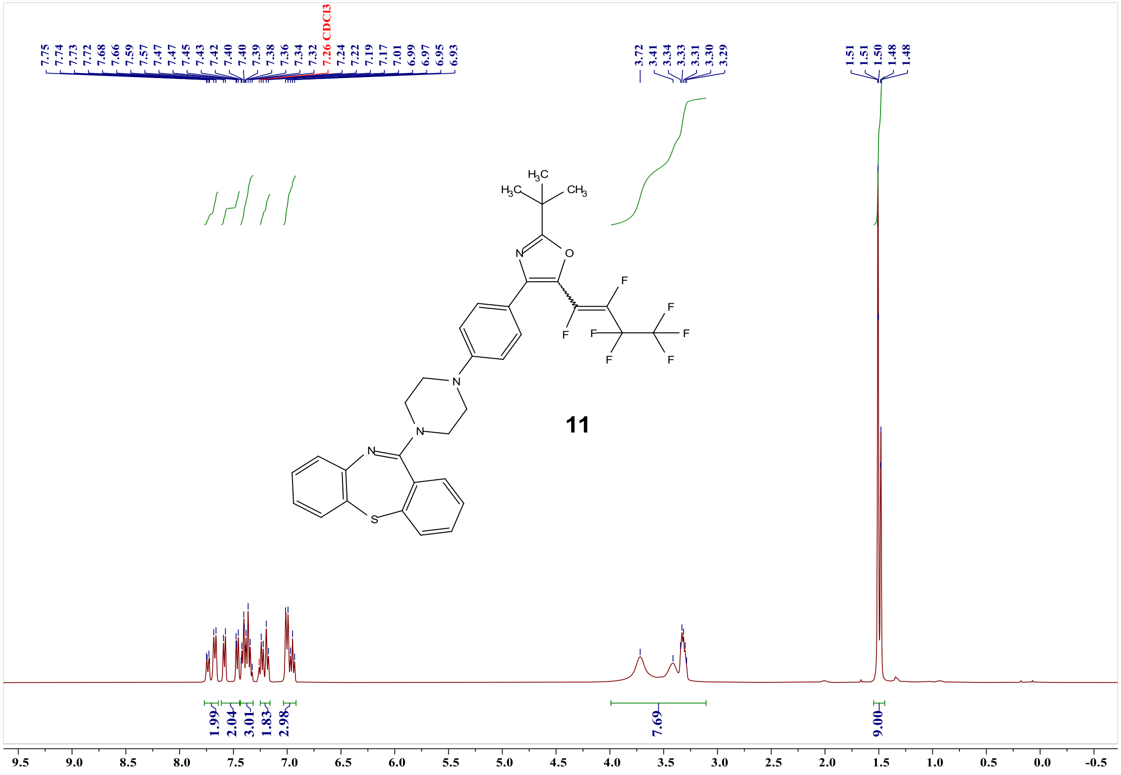

^{19}F NMR spectra of the product **11** (376 MHz, $CDCl_3$)

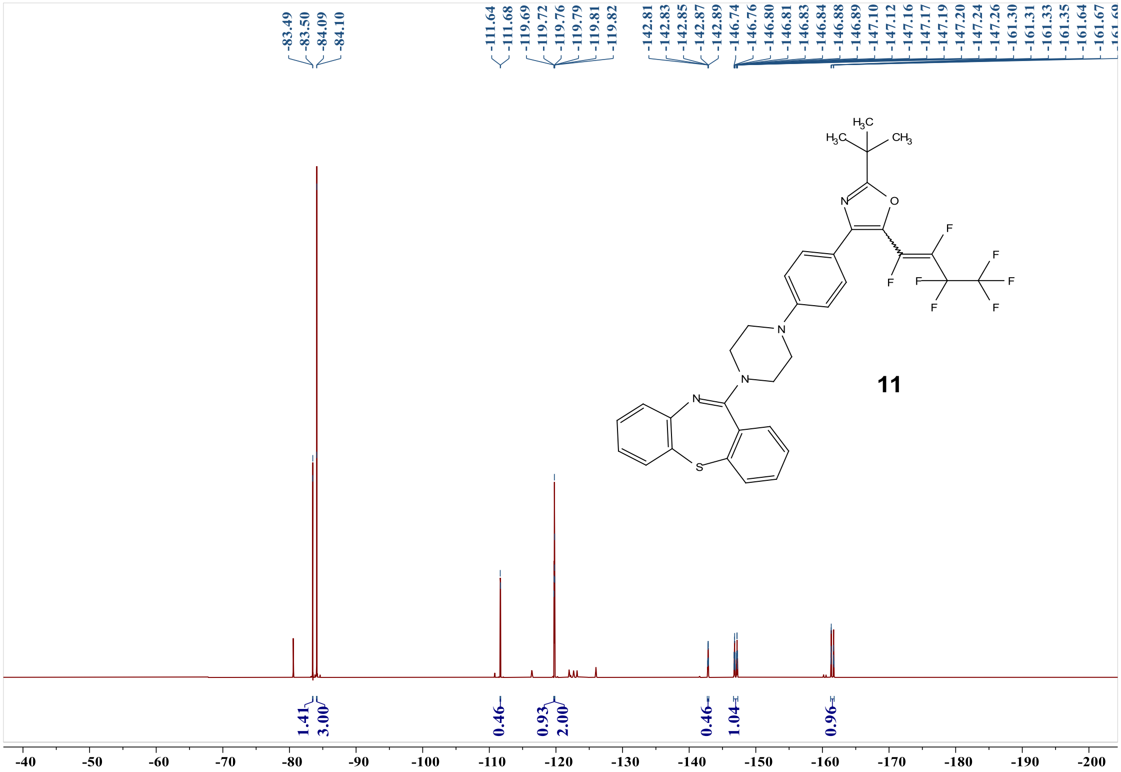

^{13}C NMR spectra of the product **11** (100 MHz, $CDCl_3$)

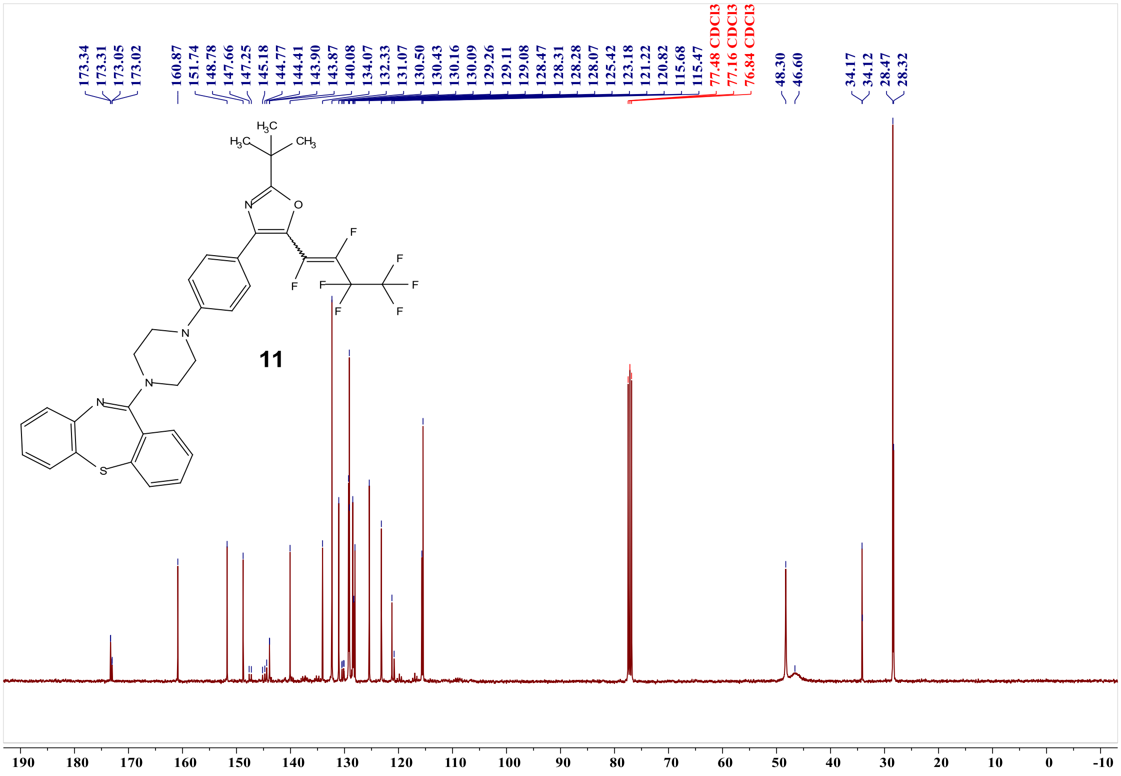

Supplement: Supplementary file 1 — Supporting Information [file ADVS-12-2404738-s001.pdf]
