# Supplementary material for: Defluorinative Cyclization of Enamides with Fluoroalkyl Halides Through Two Vicinal C(sp3)─F Bonds Functionalization
Source: Adv Sci (Weinh). 2024 Dec 31;12(8):2404738. doi: 10.1002/advs.202404738 (PMC11848561; doi:10.1002/advs.202404738)

checkCIF/PLATON report

Structure factors have been supplied for datablock(s) 8625

THIS REPORT IS FOR GUIDANCE ONLY. IF USED AS PART OF A REVIEW PROCEDURE FOR PUBLICATION, IT SHOULD NOT REPLACE THE EXPERTISE OF AN EXPERIENCED CRYSTALLOGRAPHIC REFEREE.

No syntax errors found. CIF dictionary Interpreting this report

Datablock: 8625

```
Bond precision:   C-C = 0.0057 A           Wavelength=1.54184

Cell:           a=10.1707(12)      b=10.8048(13)     c=16.5700(18)
                alpha=79.087(10)   beta=76.566(10)   gamma=85.923(10)
Temperature:    293 K

                 Calculated                    Reported
Volume           1738.4(4)                     1738.4(4)
Space group      P -1                          P -1
Hall group       -P 1                          -P 1
Moiety formula   C19 H10 F7 N O                C19 H10 F7 N O
Sum formula      C19 H10 F7 N O                C19 H10 F7 N O
Mr               401.28                        401.28
Dx,g cm-3        1.533                         1.533
Z                4                             4
Mu (mm-1)        1.306                         1.306
F000             808.0                         808.0
F000'            811.63
h,k,lmax         12,12,19                      12,12,19
Nref             6138                          5899
Tmin,Tmax        0.829,0.878                   0.514,1.000
Tmin'            0.811

Correction method= # Reported T Limits: Tmin=0.514 Tmax=1.000
AbsCorr = MULTI-SCAN

Data completeness= 0.961           Theta(max)= 66.591

R(reflections)= 0.0772( 2925)                          wR2(reflections)=
                                                       0.2902( 5899)

S = 0.977                   Npar= 505
```

The following ALERTS were generated. Each ALERT has the format
test-name_ALERT_alert-type_alert-level.
Click on the hyperlinks for more details of the test.

Alert level C

```
PLAT029_ALERT_3_C _diffrn_measured_fraction_theta_full value Low .      0.961 Why?
PLAT084_ALERT_3_C High wR2 Value (i.e. > 0.25) ...................       0.29 Report
PLAT112_ALERT_2_C ADDSYM Detects New (Pseudo) Symm. Elem       a/2         85 %Fit
PLAT230_ALERT_2_C Hirshfeld Test Diff for    C12      --C13      .        5.8 s.u.
PLAT230_ALERT_2_C Hirshfeld Test Diff for    F13      --C38      .        6.8 s.u.
PLAT241_ALERT_2_C High   'MainMol' Ueq as Compared to Neighbors of        C33 Check
PLAT242_ALERT_2_C Low    'MainMol' Ueq as Compared to Neighbors of        C18 Check
PLAT242_ALERT_2_C Low    'MainMol' Ueq as Compared to Neighbors of        C29 Check
PLAT242_ALERT_2_C Low    'MainMol' Ueq as Compared to Neighbors of        C32 Check
PLAT242_ALERT_2_C Low    'MainMol' Ueq as Compared to Neighbors of        C37 Check
PLAT331_ALERT_2_C Small Aver Phenyl C-C Dist C29      --C34      .       1.36 Ang.
PLAT340_ALERT_3_C Low Bond Precision on  C-C Bonds ...............    0.00568 Ang.
PLAT905_ALERT_3_C Negative K value in the Analysis of Variance ...     -2.297 Report
PLAT911_ALERT_3_C Missing FCF Refl Between Thmin & STh/L=    0.595        239 Report
```

Alert level G

```
PLAT072_ALERT_2_G SHELXL First  Parameter in WGHT  Unusually Large       0.17 Report
PLAT154_ALERT_1_G The s.u.'s on the Cell Angles are Equal ..(Note)       0.01 Degree
PLAT199_ALERT_1_G Reported _cell_measurement_temperature ..... (K)        293 Check
PLAT200_ALERT_1_G Reported   _diffrn_ambient_temperature ..... (K)        293 Check
PLAT242_ALERT_2_G Low    'MainMol' Ueq as Compared to Neighbors of        C19 Check
PLAT242_ALERT_2_G Low    'MainMol' Ueq as Compared to Neighbors of        C38 Check
PLAT398_ALERT_2_G Deviating  C-O-C   Angle From 120 for O1       .      104.1 Degree
PLAT398_ALERT_2_G Deviating  C-O-C   Angle From 120 for O2       .      104.3 Degree
PLAT941_ALERT_3_G Average HKL Measurement Multiplicity ...........        2.3 Low
PLAT978_ALERT_2_G Number C-C Bonds with Positive Residual Density.          0 Info
```

0 **ALERT level A** = Most likely a serious problem - resolve or explain
0 **ALERT level B** = A potentially serious problem, consider carefully
14 **ALERT level C** = Check. Ensure it is not caused by an omission or oversight
10 **ALERT level G** = General information/check it is not something unexpected

3 ALERT type 1 CIF construction/syntax error, inconsistent or missing data
15 ALERT type 2 Indicator that the structure model may be wrong or deficient
6 ALERT type 3 Indicator that the structure quality may be low
0 ALERT type 4 Improvement, methodology, query or suggestion
0 ALERT type 5 Informative message, check

It is advisable to attempt to resolve as many as possible of the alerts in all categories. Often the minor alerts point to easily fixed oversights, errors and omissions in your CIF or refinement strategy, so attention to these fine details can be worthwhile. In order to resolve some of the more serious problems it may be necessary to carry out additional measurements or structure refinements. However, the purpose of your study may justify the reported deviations and the more serious of these should normally be commented upon in the discussion or experimental section of a paper or in the "special_details" fields of the CIF. checkCIF was carefully designed to identify outliers and unusual parameters, but every test has its limitations and alerts that are not important in a particular case may appear. Conversely, the absence of alerts does not guarantee there are no aspects of the results needing attention. It is up to the individual to critically assess their own results and, if necessary, seek expert advice.

Publication of your CIF in IUCr journals

A basic structural check has been run on your CIF. These basic checks will be run on all CIFs submitted for publication in IUCr journals (*Acta Crystallographica*, *Journal of Applied Crystallography*, *Journal of Synchrotron Radiation*); however, if you intend to submit to *Acta Crystallographica Section C* or *E* or *IUCrData*, you should make sure that full publication checks are run on the final version of your CIF prior to submission.

Publication of your CIF in other journals

Please refer to the *Notes for Authors* of the relevant journal for any special instructions relating to CIF submission.

PLATON version of 19/02/2022; check.def file version of 19/02/2022

Datablock 8625 - ellipsoid plot

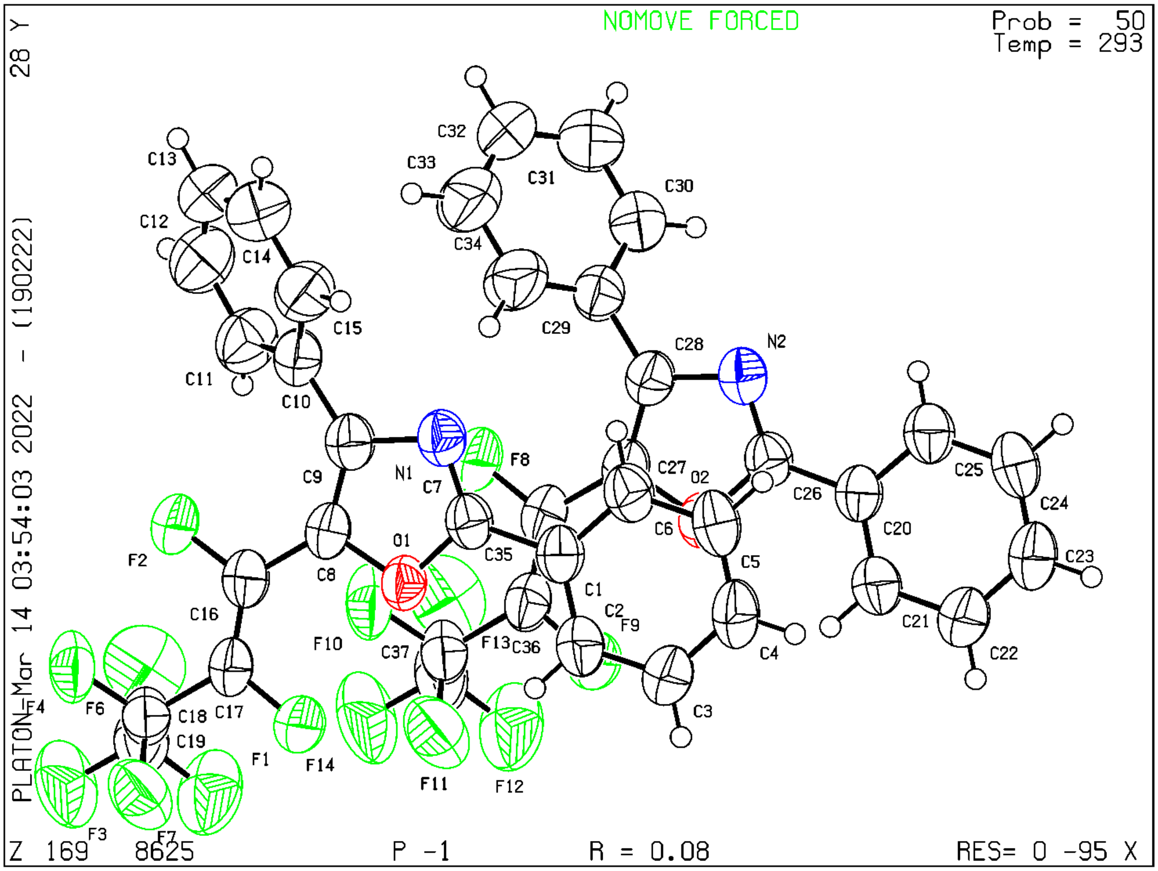

Supplement: Supplementary file 2 — Supporting cif [file ADVS-12-2404738-s002.zip › (E)-3va checkcif CCDC 2158255.pdf]
